# A novel method to determine perineal artery occlusion among male bicyclists

**DOI:** 10.7717/peerj.1477

**Published:** 2015-12-21

**Authors:** Sujeeth Parthiban, James M. Hotaling, Martin Kathrins, Amit P. Baftiri, Sally Freels, Craig S. Niederberger

**Affiliations:** 1 Department of Bioengineering, University of Illinois at Chicago, Chicago, IL, United States; 2 Department of Urology, University of Illinois at Chicago, Chicago, IL, United States; 3 Department of Radiology, University of Illinois at Chicago, Chicago, IL, United States; 4 Epidemiology and Biostatistics division, University of Illinois at Chicago, Chicago, IL, United States

**Keywords:** Erectile dysfunction, Bicycling, Impotence

## Abstract

**Background.** Perineal pressure due to bicycle riding has been associated with erectile dysfunction. We developed a novel method to measure the occlusive force exerted over the perineal arteries and determined perineal artery occlusion by a variety of seat designs.

**Methods.** Doppler ultrasonography facilitated perineal artery localization and determination of the force required for perineal artery occlusion in 20 healthy men. Flexiforce^®^ sensors were affixed over the proximal and distal aspects of the perineal arteries bilaterally. Individuals completed bicycle rides in the road- and stationary-settings with six distinct seat designs, including those with and without an anterior “nose.”

**Results.** The occlusion time proportion of the total ride time was calculated for each trial. The overall occlusion time proportion was 0.59 (95% CI [0.45–0.73]) across all seats and settings. The “no-nose” bicycle seat and the stationary-setting demonstrated significantly lower occlusion proportion times than the traditional nose bicycle seat and road-setting, respectively. However, all bicycle seats yielded an occlusion time proportion of 0.41 or greater.

**Discussion.** Our method of real-time, non-invasive force measurement localized to the perineal arteries may be used to validate future bicycle seat design. It also underscores the significant risk of perineal artery insufficiency in men who are avid bicyclists. This risk may be minimized by using newer “no-nose” bicycle seats.

## Introduction

Bicycling is an increasingly popular means of transportation and sport. A US National Survey estimated that, in the summer of 2002, 43% of the driving age public rode a bicycle, accounting to 2.5 billion bicycling trips (Royal & Miller-Steiger, 2008). Active commuting, such as bicycling, has been associated with reduced risk for the development of cardiovascular disease and obesity (Gordon-Larsen et al., 2009). It has been reported that 21% of sport cyclists complained genital numbness after a bicycle race and 13% reported impotence (Andersen & Bovim, 1997). Multiple studies have correlated bicycle riding with the development of erectile dysfunction (ED) in longitudinal epidemiologic cohorts and controlled experiments. (Dettori et al., 2004; Goldstein, Lurie & Lubisich, 2007; Marceau et al., 2001; Schrader et al., 2002; Schwarzer et al., 2002; Sommer, Goldstein & Korda, 2010).

Sexual dysfunction induced by bicycle riding is a multifactorial process. Sommer et al. (2001) interviewed 100 avid male long-distance cyclists. They found genital numbness present in 61% of cyclists. 31% of cyclists complained of both penile and scrotal numbness. All of the interviewed cyclists with erectile dysfunction also complained of genital numbness (Sommer et al., 2001). Such genital numbness may be caused by a combination of pudendal nerve impingement and perineal arterial occlusion, the anatomic courses of which are in close proximity. It has been previously asserted that perineal artery occlusion may lead to insufficiency of the blood supply to the penile tissues (Sommer, Goldstein & Korda, 2010). Even in non erect penile tissues, the resulting hypoxemia may negatively affect the dynamic biochemical pathways necessary for normal erectile function (Sommer, Goldstein & Korda, 2010). However, prior studies have not collected data on the focal forces applied over the perineal arteries during a real-world road-setting, which represent the typical conditions for a male rider.

We created a novel device enabling *in vivo*, real-time, non-invasive measurement of the forces exerted on the perineal arteries during bicycling in both a stationary- and road-setting. We used Doppler ultrasound to identify perineal artery occlusion forces and to facilitate force sensor placement. Using both an outdoor road-setting and indoor stationary-setting, we tested multiple bicycle seat designs on 20 subjects for the proportion of time during the ride that the perineal arteries would be occluded.

## Materials and Methods

All procedures in our study were reviewed and approved by the University of Illinois at Chicago Institutional Review Board (Protocol # 2007 -0284). Written informed consent was obtained from all the subjects. Twenty healthy, avid male bicyclists were recruited from the community. Baseline biometric information was recorded. We developed a portable device to record force measurements from Flexiforce^®^ sensors (Tekscan Inc., Boston, MA, USA). The development of the device, calibration and validation was previously described (Parthiban et al., 2014). Flexiforce^®^ sensors were selected to measure force as they are thin, flexible and not inconvenient when applied to the perineum of the riders.

**Figure 1:**
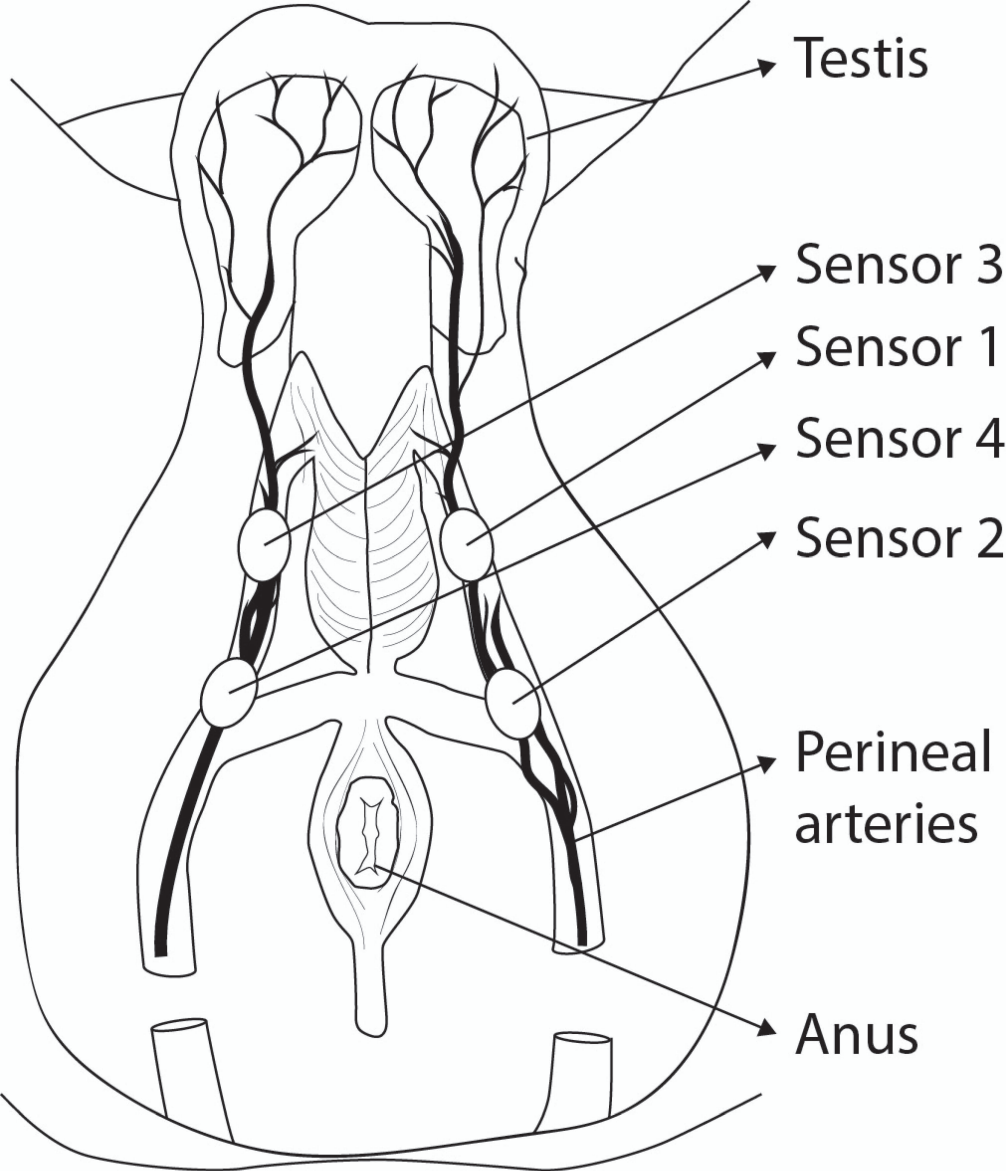
Position of sensors on the Perineum.

The subjects’ heart rates were increased to 120 through moderate exercise (stair climbing) in order to account for changes in blood pressure during exercise. The following measurements were completed prior to the resting heart rate returning to baseline. The force required to occlude the perineal artery was measured in a laboratory setting. The subjects were examined in the supine, hip-flexed, frog-legged position to allow access to the perineum. An experienced ultrasonographer used a GE LOGIQ^®^e 9L-RS (GE Healthcare, Milwaukee, WI, USA) Doppler ultrasound probe to identify the left and right perineal arteries in both proximal and distal positions. Increasing pressure on the perineum was applied using the Flexiforce^®^ force-sensing probe until cessation of blood flow in the perineal arteries bilaterally, as measured by Doppler. This was performed twice to account for variability and the mean arterial occlusive force was recorded. Flexiforce^®^ sensors were affixed at cutaneous positions bilaterally overlying the perineal arteries in both proximal and distal locations (Fig. 1) using Tegaderm™ (3M, St. Paul, MN, USA). The sensors were then attached to the custom-designed device located in a low-profile backpack and data collected at 10 Hz. The same kind of sensors and recording device were used for all measurements.

**Figure 2:**
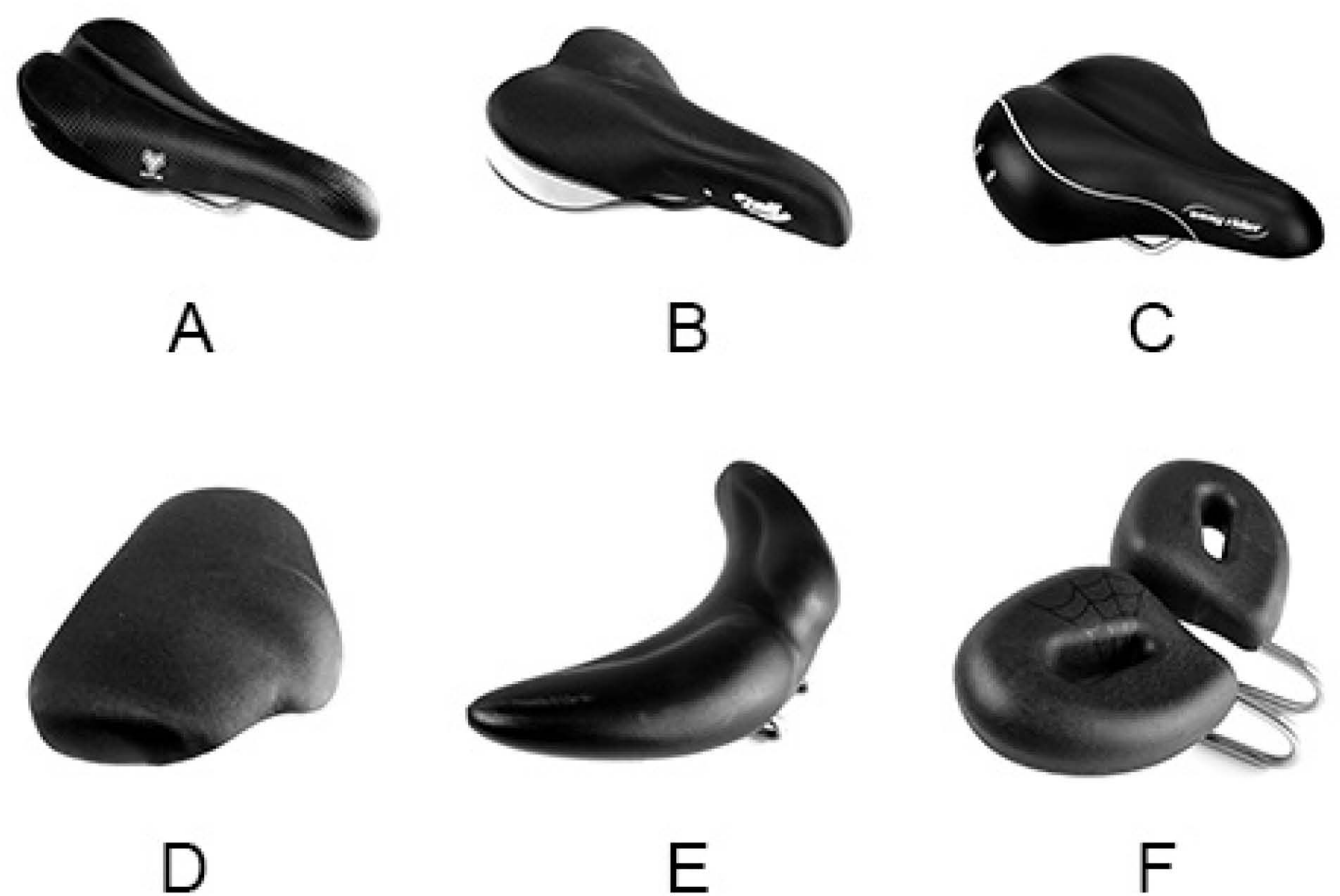
Test seats models.

The bicycle seat models varied in size, shape, and padding. Seats were kept at uniform angle parallel to the ground. Three had a conventional anterior “nose” (A, B, C,) and three had a newer “no-nose” design (D, E, F). Among the traditional “nose” seats, seats B and C had soft padding and grooved center channel. For the road setting, subjects bicycled on a standard flat, city course for 0.5 miles on each of six bicycle seats (Fig. 2). A hybrid 19.5″, bicycle frame (Trek FX™ ) was used by all the subjects. Only the saddle height was adjusted for each saddle, so that subjects are at a trunk angle where they feel comfortable. For the stationary setting, the protocol was repeated on the same bicycle frame mounted on a trainer. Subjects were asked to wear normal athletic shorts and refrained from using bicycle shorts, as it may interfere with the sensor recordings. Zero force recordings from all the sensors before and after bicycling ensured that none of the sensors were malfunctioning. Recorded perineal forces were compared to the subjects’ previously measured occlusion force. The order of testing was randomized across all test subjects and all subject trials were performed in one day. Bicycling experience was later collected from subjects through electronic communication.

Occlusion time proportion (OTP) was defined as the proportion of total ride time when any of the four unique perineal force sensors exceeded the respective perineal arterial occlusion force. Generalized estimating equation (GEE) models estimated OTP for each combination of seat and condition. These models are preferable to mixed effects models for estimating marginal distributions. The GEE model accounted for potential correlation among repeated sensor readings within individuals. GEE models were fit using PROC GENMOD in SAS version 9.2 (SAS, Cary, NC, USA) specifying binary outcome, identity link, and exchangeable correlation structure. The average sampling duration for a subject on a seat was approximately 5 min. A binary indicator was calculated for force above occlusion in any of the four sensors at each time point. The total sample size was 754,940 binary indicators of occlusion, which was the sum of all the discrete measurements from the 20 subjects. Standard errors of proportion estimates were similar in magnitude to standard errors treating the average proportion from each trial as a single continuous measurement and working with a sample size of 20. Specifically, there was a very accurate measure of proportion for each subject under each condition, but the inference was based on 20 subjects. The effect of road- versus stationary-condition was tested overall in a model without interaction between seat and condition, as well as contrasts for each type of seat in the full interaction model. Tests were done to compare seats (B, C, D, E, and F) against seat A within the road ride condition, as seat A has a prototypical bike saddle shape with no padding and can be considered as a standard seat. All statistical analyses were performed using GENMOD procedure on SAS version 9.2 (SAS, Cary, NC, USA).

## Results

**Figure 3:**
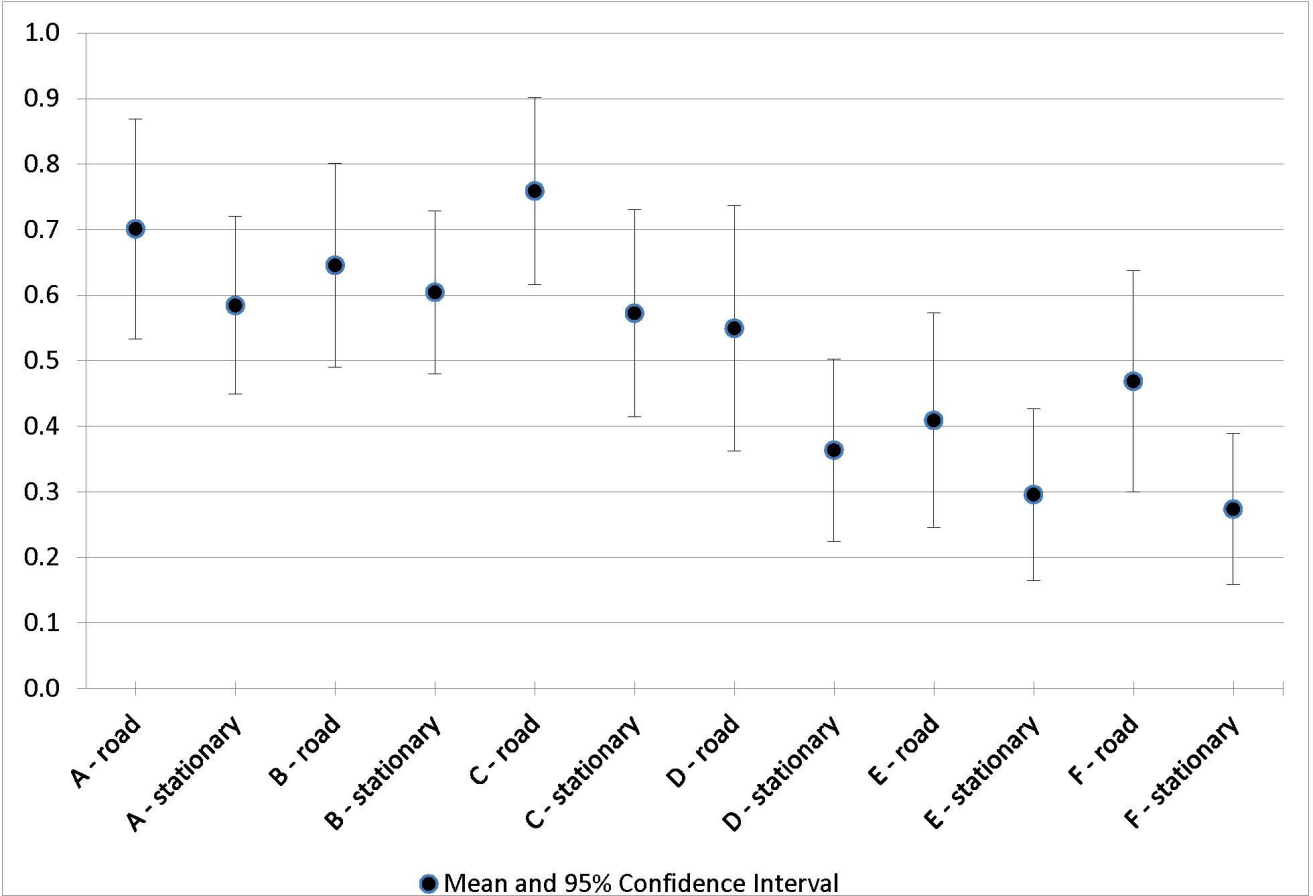
Proportion of time above occlusion force in any of four sensors.

Twenty riders completed the study (mean age: 36.35 ± 12.5 years; BMI: 23.70 ± 2.6) with a mean occlusion force of 10.03 ± 1.29 N on the right perineal artery and 10.03 ± 1.5 N on the left perineal artery. For all seats and conditions, the OTPs were significant (Table 1 and Fig. 3). The overall OTP was 0.59 (95% CI [0.45–0.73]) across all seats and settings. Road-setting trials produced a significantly higher OTP compared to stationary-setting trials (Table 2). This effect was observed for all seat models. As an average across all seat models, the increase in OTP associated with the road setting approached, but did not reach, statistical significance (*p* = .07). However, the increase in OTP in the road-setting compared to the stationary-setting was significant for seats C (*p* = .04), D (*p* = .03), and F (*p* = .01). The comparison of newer seat models (B, C, D, E, and F) against seat A, the prototypical seat model, is shown in Table 3. Seats D, E, and F were associated with significantly lower OTP compared to A (*p* < .001, *p* = .002, and *p* = .04, respectively). In a model grouping “nose” seats (A, B, and C) versus “no-nose” seats (D, E, and F), “nose” seats had significantly higher OTP (*p* < .001). Table 4 lists the demographic information and cycling experience for all the subjects.

**Table 1:** Proportion of time above occlusion force in any of four sensors: point estimates and standard errors from GEE model^a^.

Seat	Condition	Estimate	SE
A	Road ride	.70	.09
A	Stationary ride	.58	.07
B	Road ride	.65	.08
B	Stationary ride	.60	.06
C	Road ride	.76	.07
C	Stationary ride	.57	.08
D	Road ride	.55	.10
D	Stationary ride	.36	.07
E	Road ride	.41	.08
E	Stationary ride	.30	.07
F	Road ride	.47	.09
F	Stationary ride	.27	.06

**Notes.**

a 20 subjects; 23,369–45,356 observations per subject; 754,940 total observations.

Binary outcome, identity link, and exchangeable correlation structure specified.

Exchangeable working correlation is *ρ* = .2097.

**Table 2:** Effect of condition on occlusion time proportion (road-setting versus stationary-setting).

		Estimate	SE	*p*-value
Average across seats:^a^		+.13	.07	.07
Conditional on seat:^b^	A	+.12	.10	.24
	B	+.04	.10	.67
	C	+.19	.09	.04
	D	+.19	.09	.03
	E	+.11	.07	.10
	F	+.20	.08	.01

**Notes.**

a From GEE model without interaction terms (exchangeable working correlation is *ρ* = .3575).

b Estimates for conditional effects are from full interaction model as in Table 1.

**Table 3:** Effect of seat type on occlusion time proportion conditional on road ride.

	Estimate	SE	*p*-value
B vs. A^a^	−.06	.06	.37
C vs. A^a^	+.06	.06	.29
D vs. A^a^	−.15	.07	.04
E vs. A^a^	−.29	.07	<.001
F vs. A^a^	−.23	.07	.002
Effect of nosed seat^b^ (A, B, or C vs. D, E, or F)	+.23	.06	<.001

**Notes.**

a From full interaction model as in Table 1.

b From GEE model with indicator for “nose” seat, condition (road-setting versus stationary-setting) and the interaction between “nose” seat and condition.

**Table 4:** Demographic information of the individual subjects participated in the study.

Subject	Age (years)	Height (cm)	Weight (kg)	BMI	Bicycling experience (miles/week)
1	63	177.8	84.1	26.60	120
2	25	170.2	64.5	22.27	125
3	23	175.3	68	22.13	80
4	44	177.8	70.5	22.30	200
5	28	167.6	75	26.70	^a^
6	23	175.3	79.5	25.87	300
7	27	175.3	65.9	21.44	120
8	27	172.7	79.5	26.66	150
9	31	176.1	69.1	22.28	100
10	57	175.3	88.6	28.83	200
11	26	175.1	68.2	22.24	75
12	35	172.7	63.6	21.32	100
13	36	165.1	75	27.51	100
14	33	176.1	82	26.44	^a^
15	31	188	72.3	20.46	300
16	62	185	77.2	22.56	125
17	46	179	69.2	21.60	^a^
18	44	174	63.2	20.87	75
19	29	171	62.7	21.44	180
20	37	189	87.7	24.55	150
Mean	36.35	175.92	73.29	23.70	147.06
S.D	12.50	6.01	8.17	2.62	69.58

**Notes.**

a Data unavailable.

## Discussion

The repetitive stress of bicycle riding has been associated with the development of ED. In an analysis of the Massachusetts Male Aging Study, men who participated in bicycling more than three hours per week were more likely to suffer from ED than men who participated less than three hours per week after controlling for age and co-morbidities (Marceau et al., 2001). A prospective study of 463 cyclists showed that 4.2% of the cyclists developed *de-novo* ED one week after completing a 320 km course (Dettori et al., 2004). This data indicates that bicycle related stress and trauma may adversely affect erectile function in both the short- and long-term.

There have been a number of previous studies that investigated the effect of bicycle seat design on perineal pressure through the use of pressure mats on bicycle seats. Lowe, Schrader & Breitenstein (2004) applied pressure mats to bicycle seats and measured pressure distribution underlying the perineal region during stationary bicycling and concluded that the “no-nose” design reduced the pressure exerted on the perineum without leading to an increase shift in pressure on the pedals or handlebars. Schrader, Breitenstein & Lowe (2008) in a prospective study of male bicyclists who changed from a traditional “nose” seat to a “no-nose” seat for six months, noted improvements in International Index of Erectile Function Questionnaire, urogenital numbness, and penile vibrotactile sensitivity threshold.

We present an investigative method to monitor the effects of bicycle riding and various bicycle seat designs on perineal arterial occlusion. In order to accomplish this, we devised a novel device to record pressures exerted directly on the perineum, rather than on the bicycle seat itself as has been performed in past studies. In these previous studies, it was asserted that no such technology existed to discriminate pressure exerted by the posterior regions of the thigh from the contact force affecting the soft tissues of the perineum in a real-world setting (Schrader, Breitenstein & Lowe 2008). By locating the perineal arteries with Doppler ultrasound probes and measuring the arterial occlusion pressure, we sought to translate the pressure exerted on the perineum during bicycle riding into a more clinically meaningful variable. Our approach better accounts for anatomic differences between participants and for real-world bicycling conditions, involving differences in rider position and weight distribution which have been associated with perineal artery occlusion based on cross-sectional pelvic imaging (Sommer, Goldstein & Korda, 2010). Overall, we found that all seats and riding conditions lead to OTP ranging from 27% in a “no-nose,” broad-based saddle on a stationary-setting to 70% on a traditional “nose” saddle in a road-setting.

The mean occlusion pressure of 20 Kpa (10.03 N) for the perineal arteries is well below the previously reported peak perineal pressure of 40 Kpa for the no-nose seats (Lowe, Schrader & Breitenstein, 2004). This indicates that arterial occlusion can occur even with the newer no-nose designs. Bressel et al. (2007) noted a significant compression of cavernosal spaces that houses the arteries and nerves at perineal pressures of 15 Kpa. It should be noted that significant compression of perineal structures observed in MRI imaging may not necessarily result in complete arterial occlusion. We used Doppler ultrasound to confirm complete arterial occlusion and recorded the corresponding occlusion pressure.

Our study is the first to provide evidence that traditional “nose” seats are more likely to exert focal forces to the area overlying the perineal arteries such that they may be occluded more often than “no-nose” seats. There was no statistically significant difference in the OTP across the various traditional “nose” seats (models A, B, C), indicating that the presence of padding and grooved center channel is less clinically meaningful than the presence of an anterior nose on the seat. In fact, each of the traditional “nose” seats yielded an OTP of at least 65% during road setting bicycling tests. Yet, even the newer “no-nose” bicycle seats produced an OTP of at least 41%, indicating that any choice of seat model may lead to significant instances of perineal artery occlusion.

Bressel & Parker, (2010) observed that a 60% reduction of typical seated pressure resulted in reduced perineal compression. Lowe, Schrader & Breitenstein (2004) showed that the “no-nose” saddles resulted in reduction of pressure in the anterior region by more than 40% in a stationary trial setting. Our results showed that “no-nose” seats resulted in a 33% and 47% reduced OTP compared to the “nose” seats in road setting and stationary setting trials respectively.

Model E yielded the lowest OTP of all bicycle seat designs. This design has the least anterior, midline material and likely provides the most support to the ischial tuberosities. Finally, among all models, there was a trend toward worsening OTP when measured in the road-setting as compared to the stationary-setting. This should be taken into consideration in future evaluations of new bicycle seat designs.

The position of the perineal artery may shift relative to the cutaneous force sensors during active bicycling. However, it has been demonstrated that the most important dynamic variable in terms of arterial pressure exertion is the angle of the bony pelvis relative to the bicycle seat, which is accounted for by our performance of pressure-monitoring in a real-world, road-setting (Gemery et al., 2007). We also accounted for changes in blood pressure during exercise by performing initial measurements at an elevated heart rate after moderate exercise.

There are several limitations in our current study. First, the sample size was small. Information regarding subjects’ baseline erectile function and co-morbidities was not gathered. While we accounted for occlusion in any one of the four sensors placed, it remains to be determined if unilateral occlusion of the perineal arteries may have clinical implications. Indeed, the presence of unilateral pudendal arterial disease may be causative is up to 15% of cases of arteriogenic ED (Rosen et al., 1990). Our technique also does not account for partial occlusion of the perineal artery.

We conclude that the “no-nose” bicycle seat design is associated with significantly less instances of perineal arterial occlusive pressure during bicycling. However, all seats studied achieved occlusive pressures for a minimum 41% of riding time. Thus, all bicycle seats may contribute to perineal arterial occlusion and possibly to erectile dysfunction and genital numbness. Our investigative system serves as a novel method to validate new bicycle seat designs for their effect on perineal vasculature.

## Supplemental Information

10.7717/peerj.1477/supp-1 Supplemental Information 1 Plots of raw data This pdf includes raw data from all the subjects. The values shown are actual sensor values and not converted into force in Newton. We had 20 subjects, each rode 6 different seats under 2 scenarios. So, we have in total 240 graphs representing each.

## Additional Information and Declarations

### Data Availability

The following information was supplied regarding data availability: Raw data is included in the Supplemental Information.
