# Supplementary figures and images for: A novel method to determine perineal artery occlusion among male bicyclists
Source: PeerJ. 2015 Dec 21;3:e1477. doi: 10.7717/peerj.1477 (PMC4690354; doi:10.7717/peerj.1477)

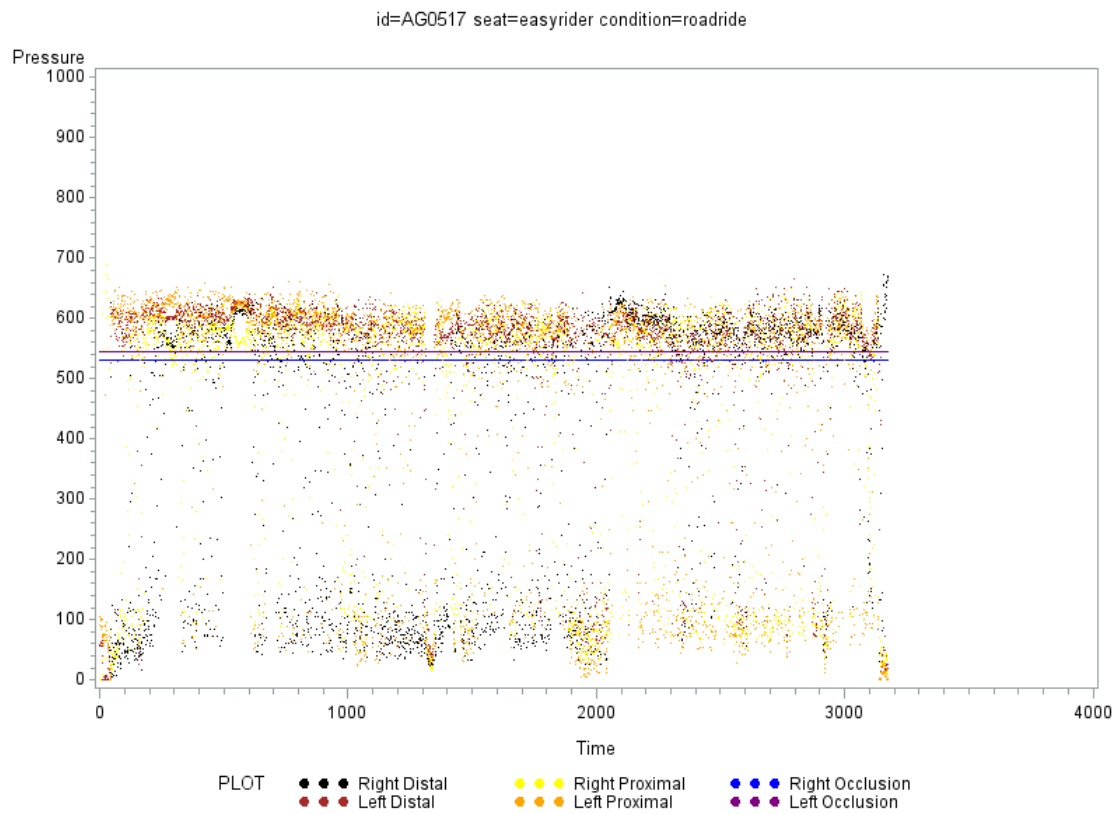

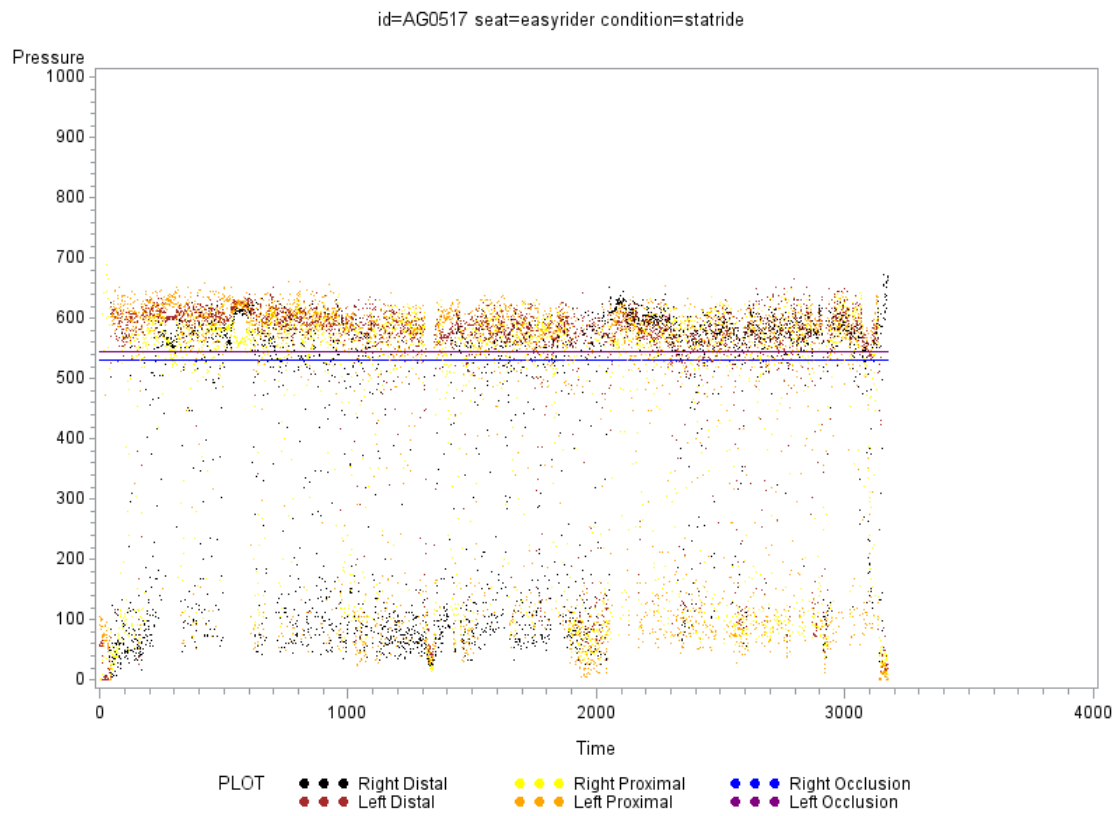

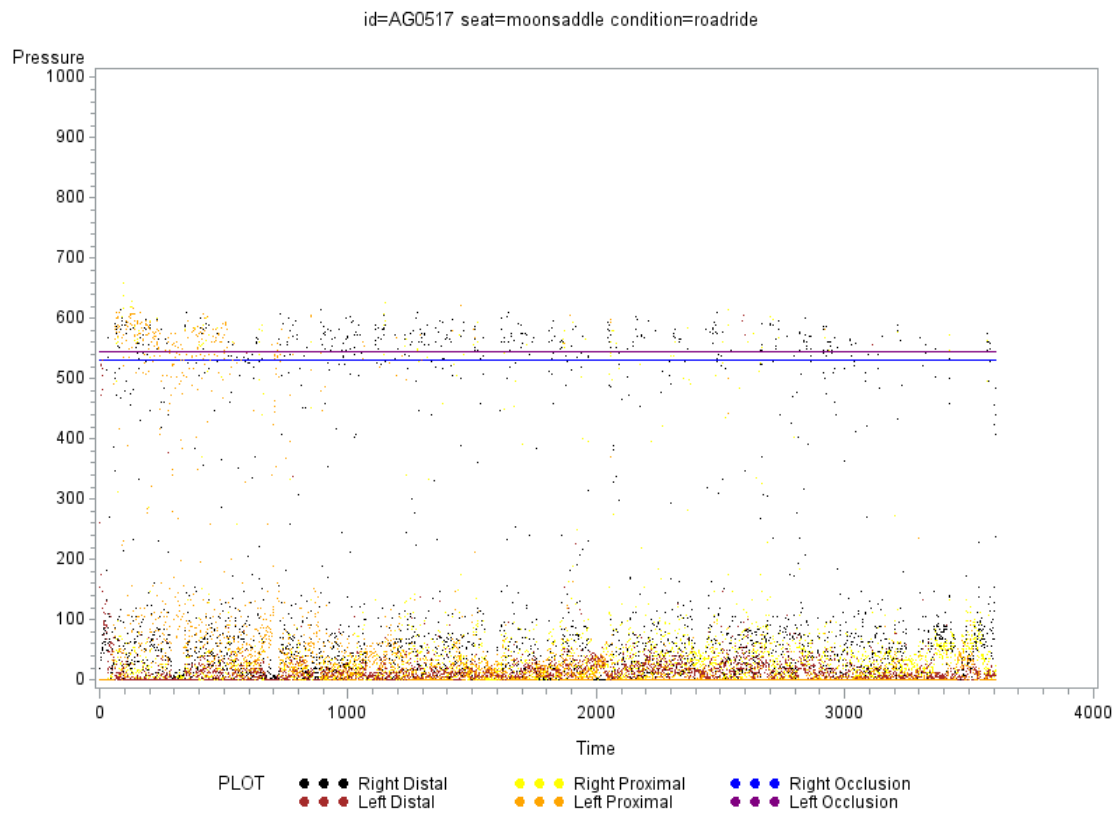

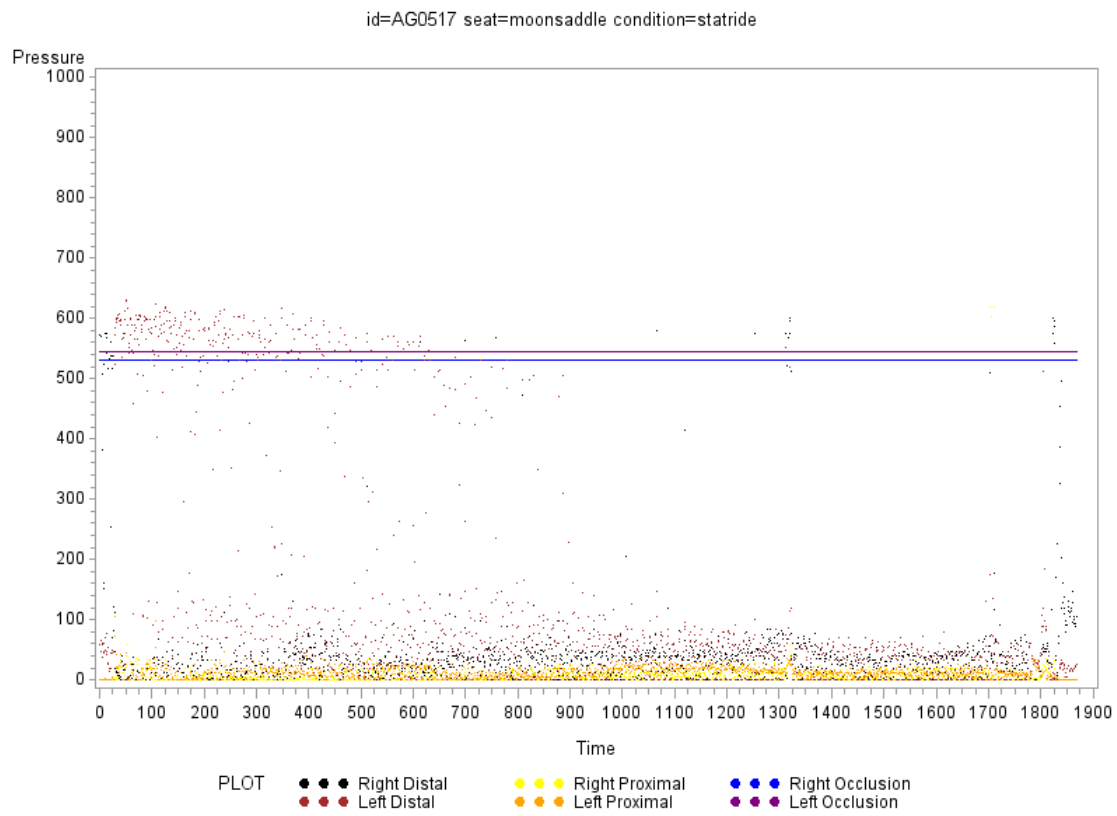

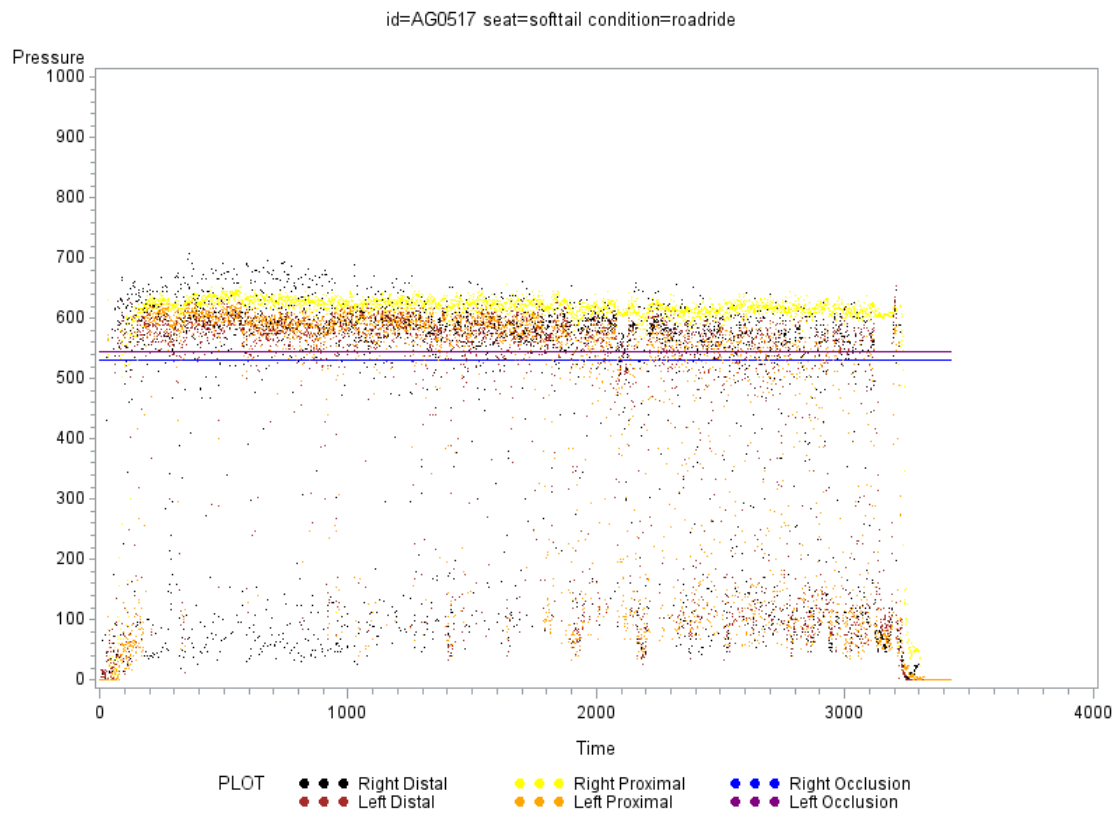

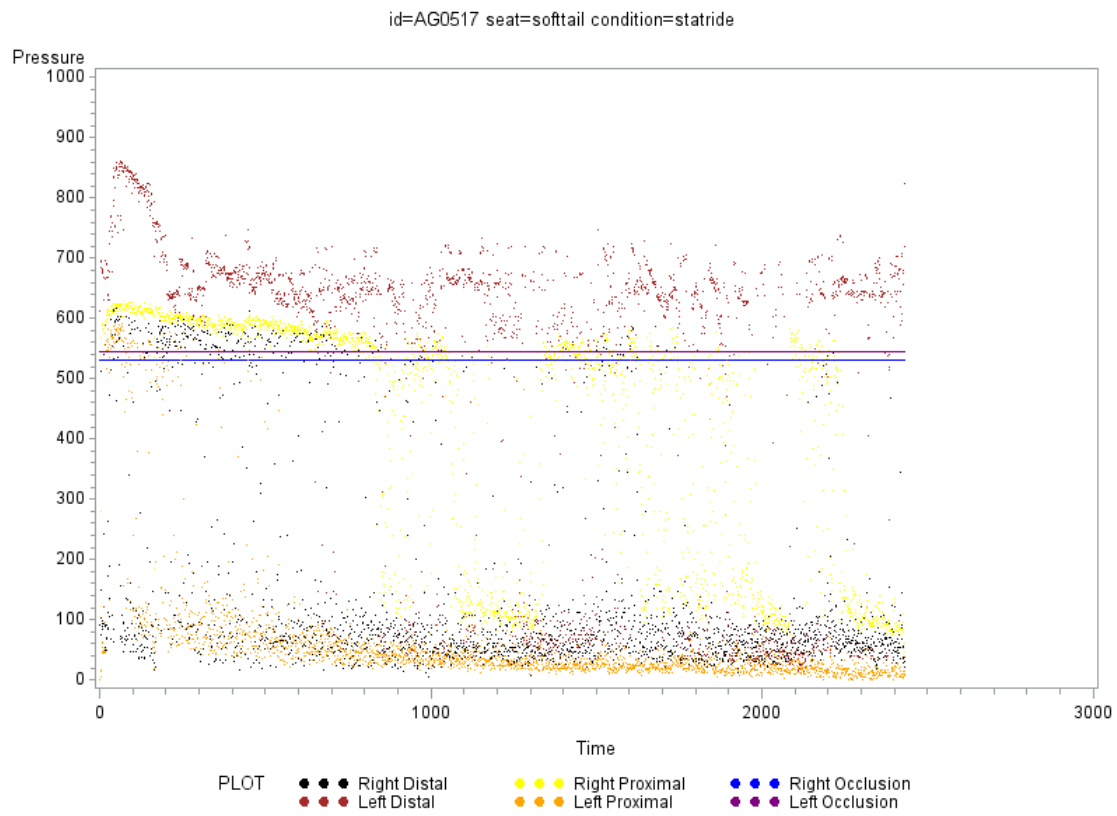

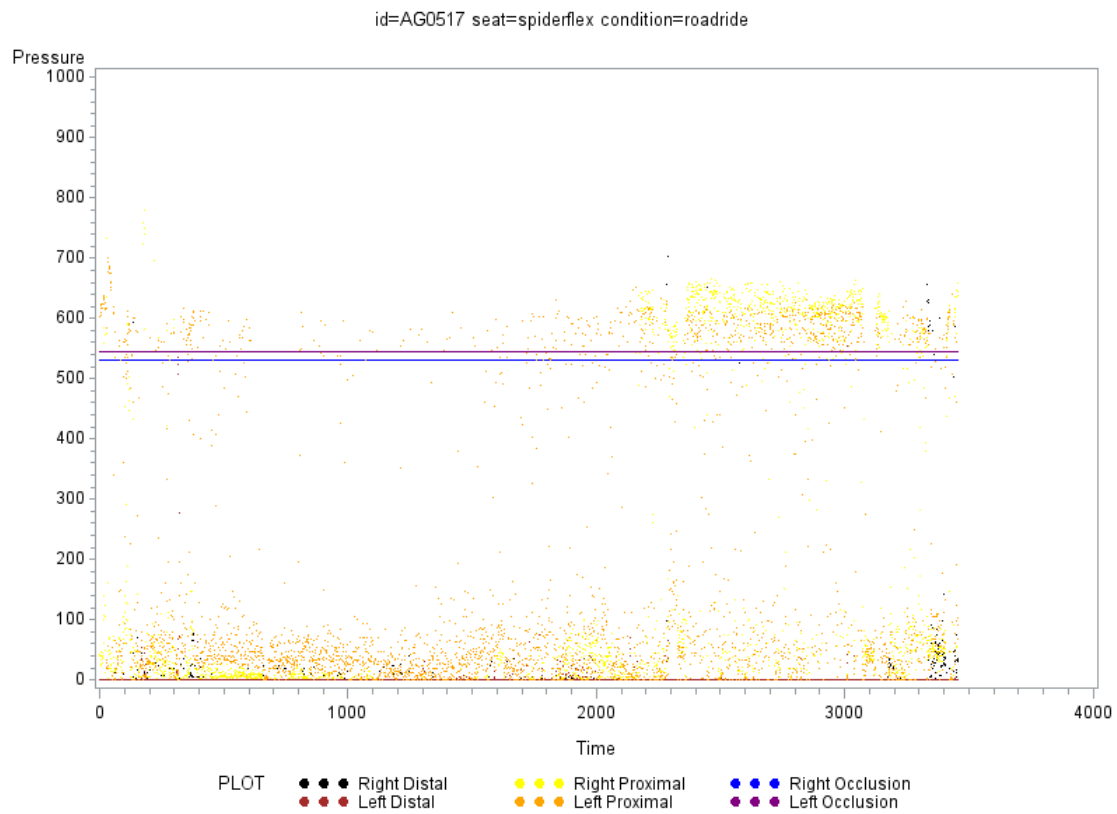

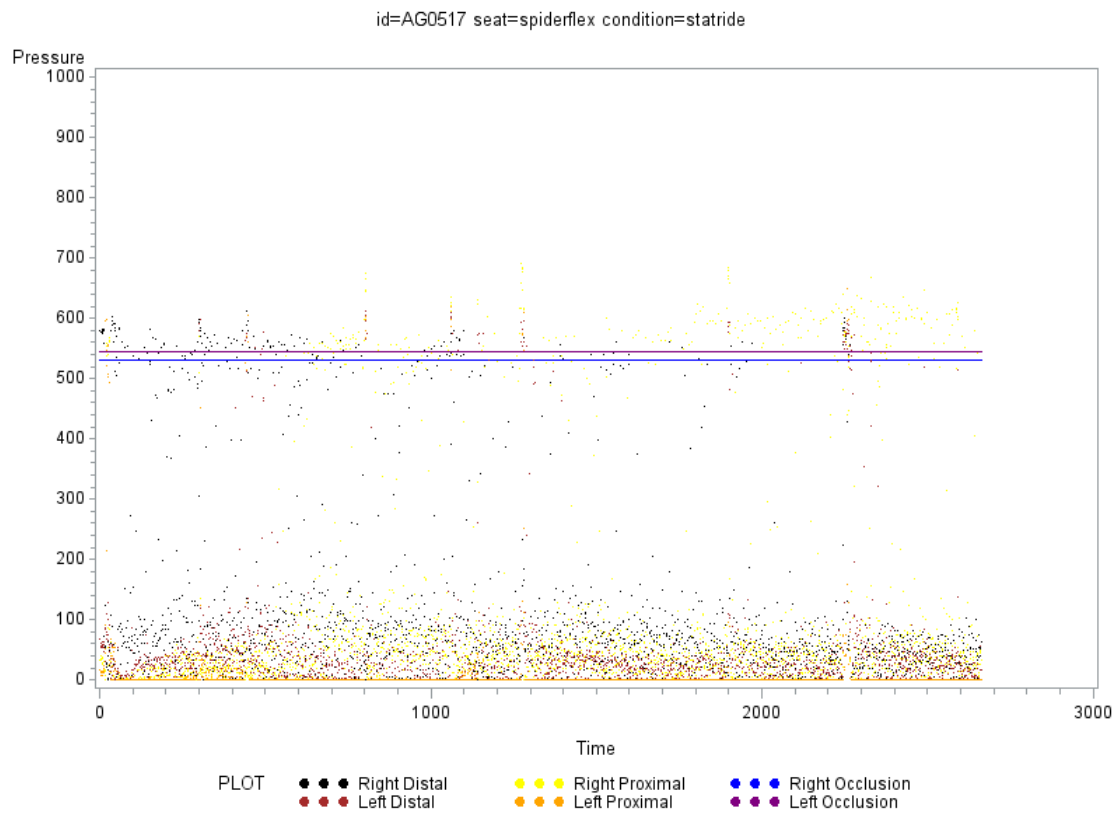

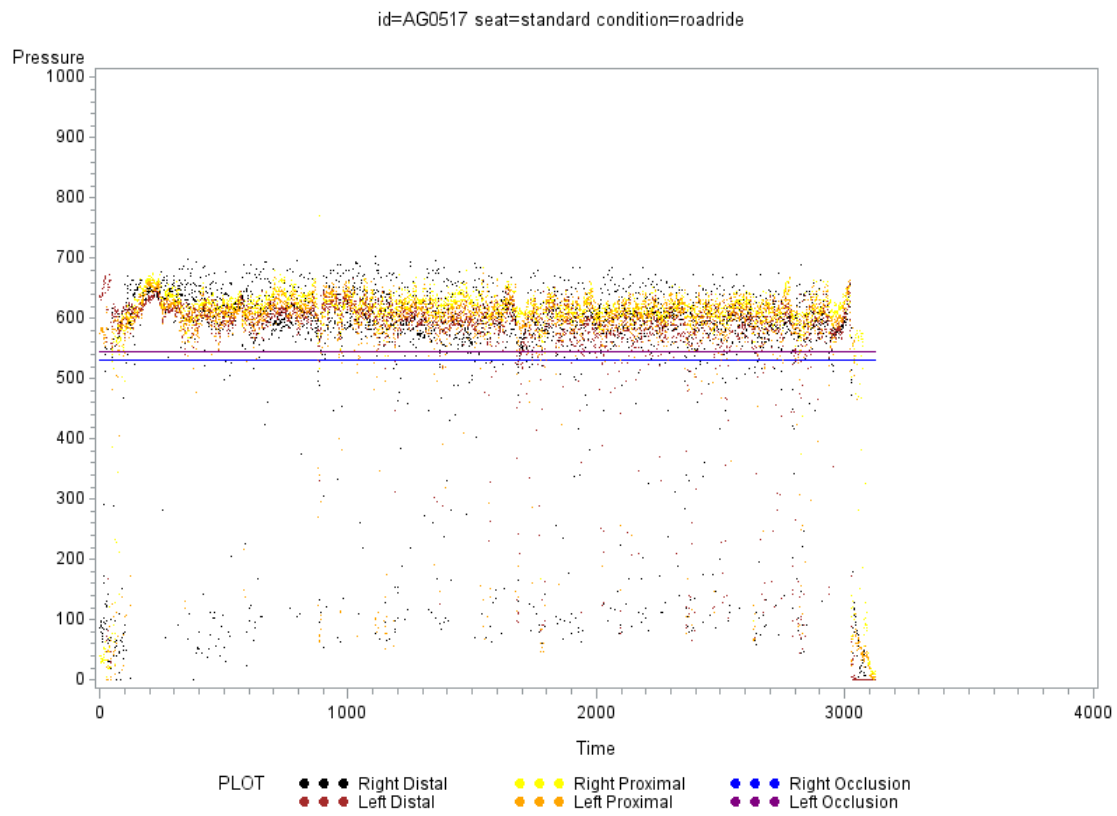

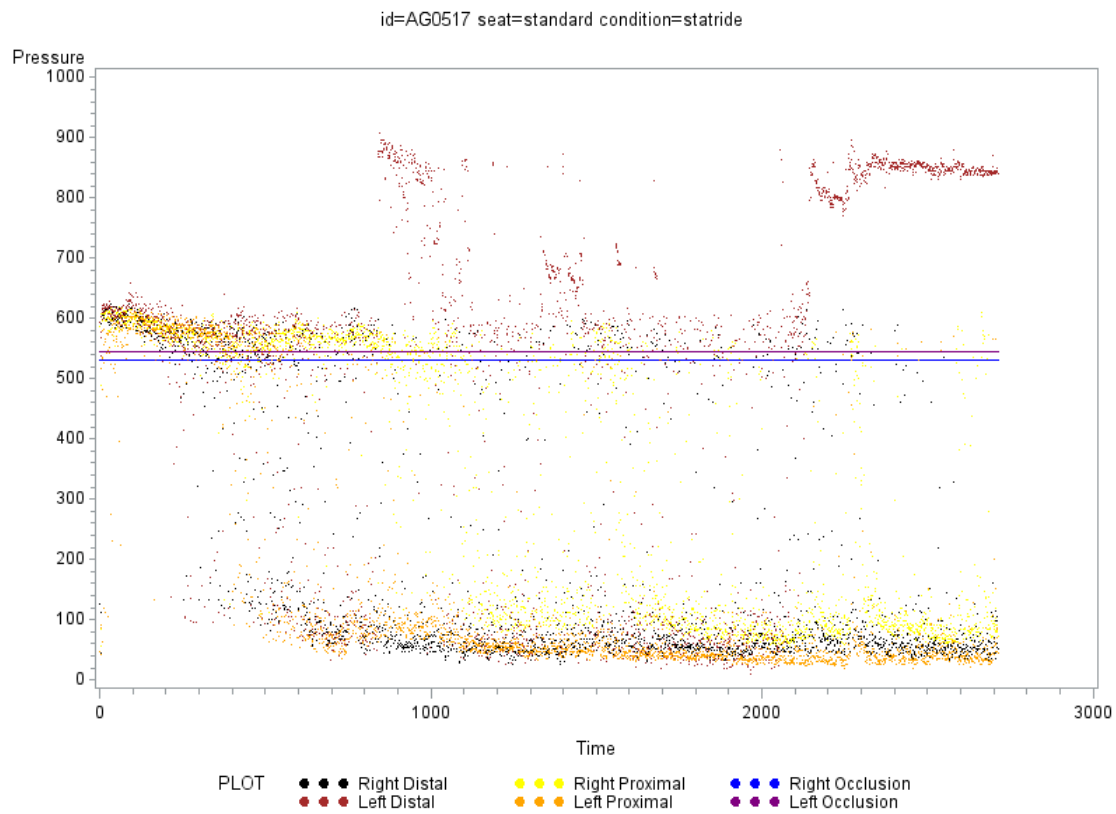

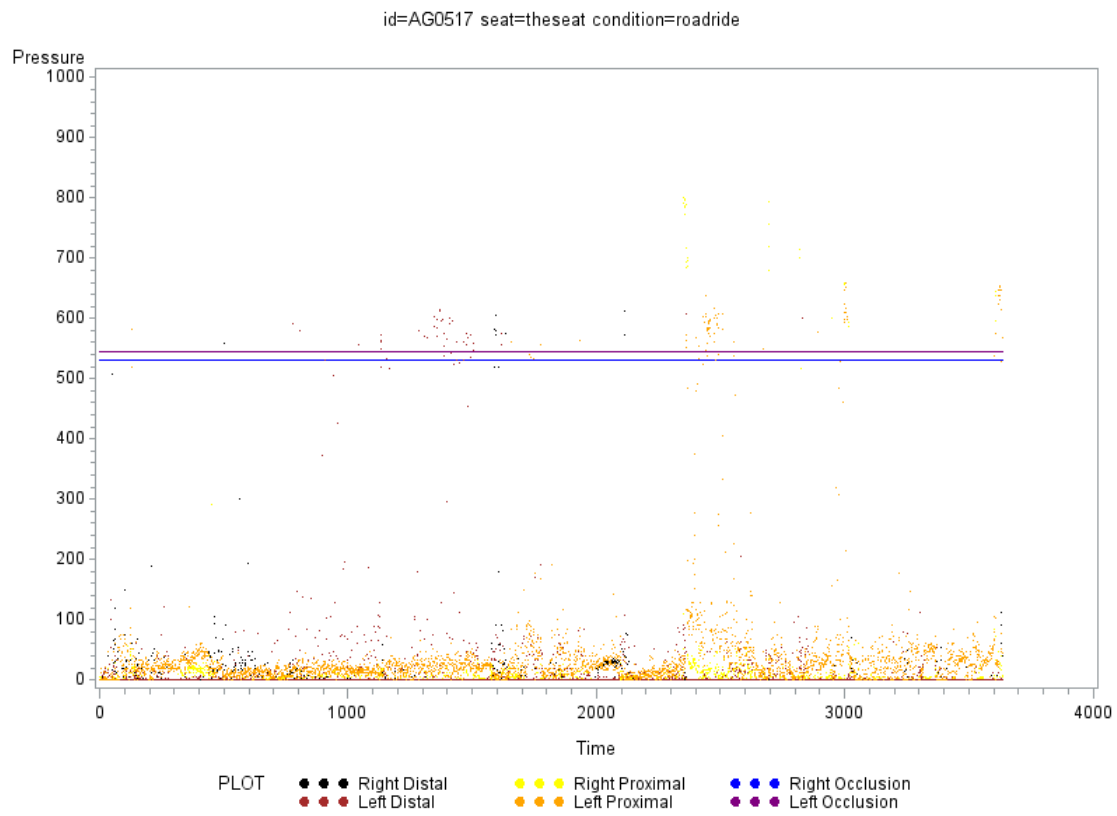

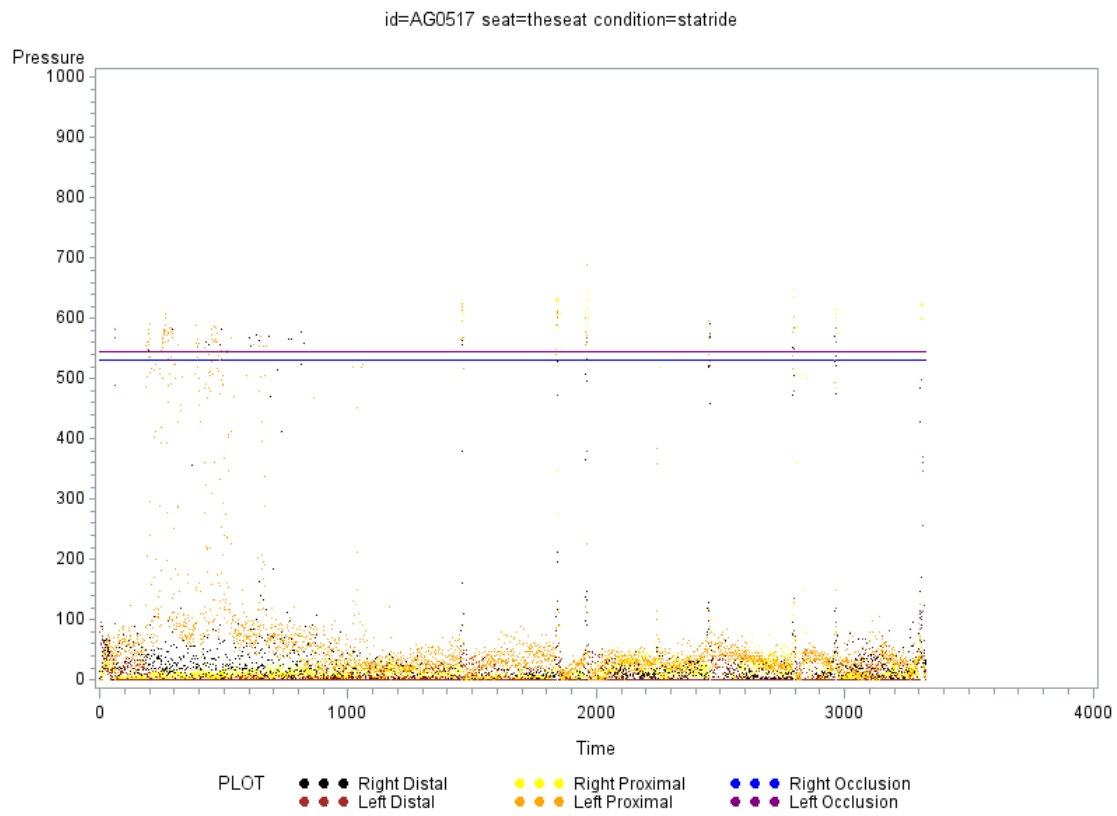

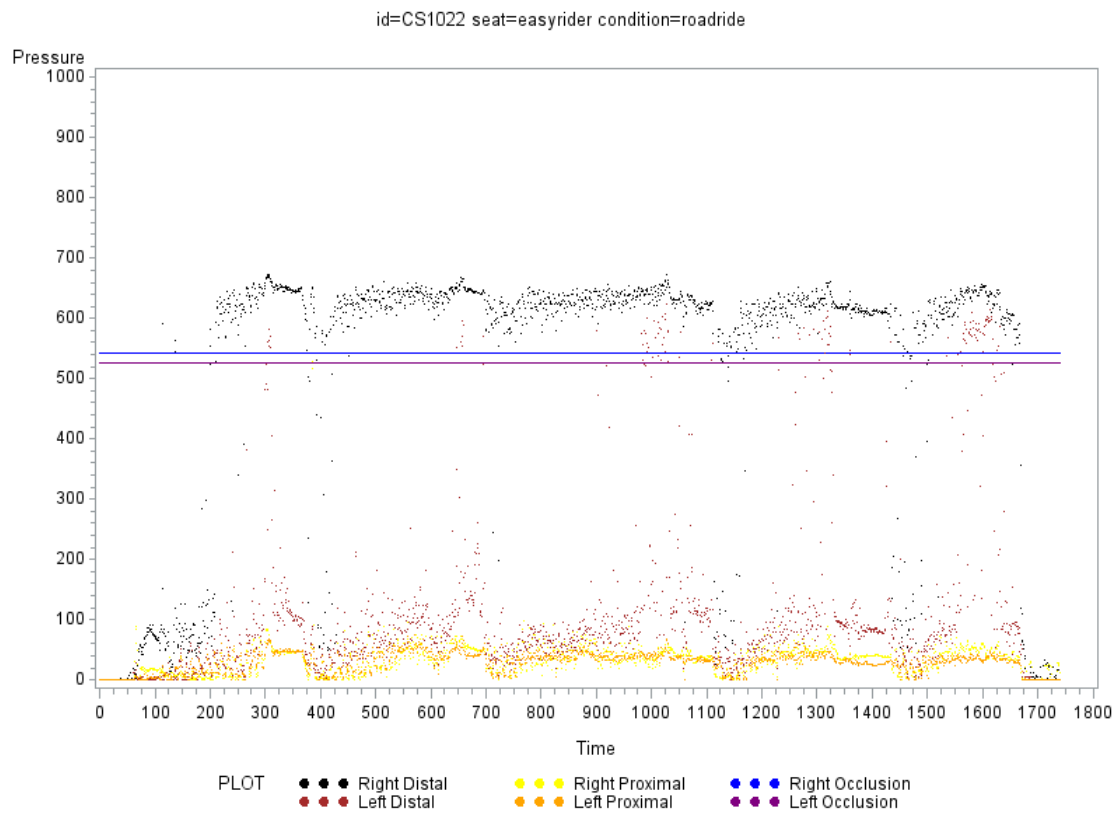

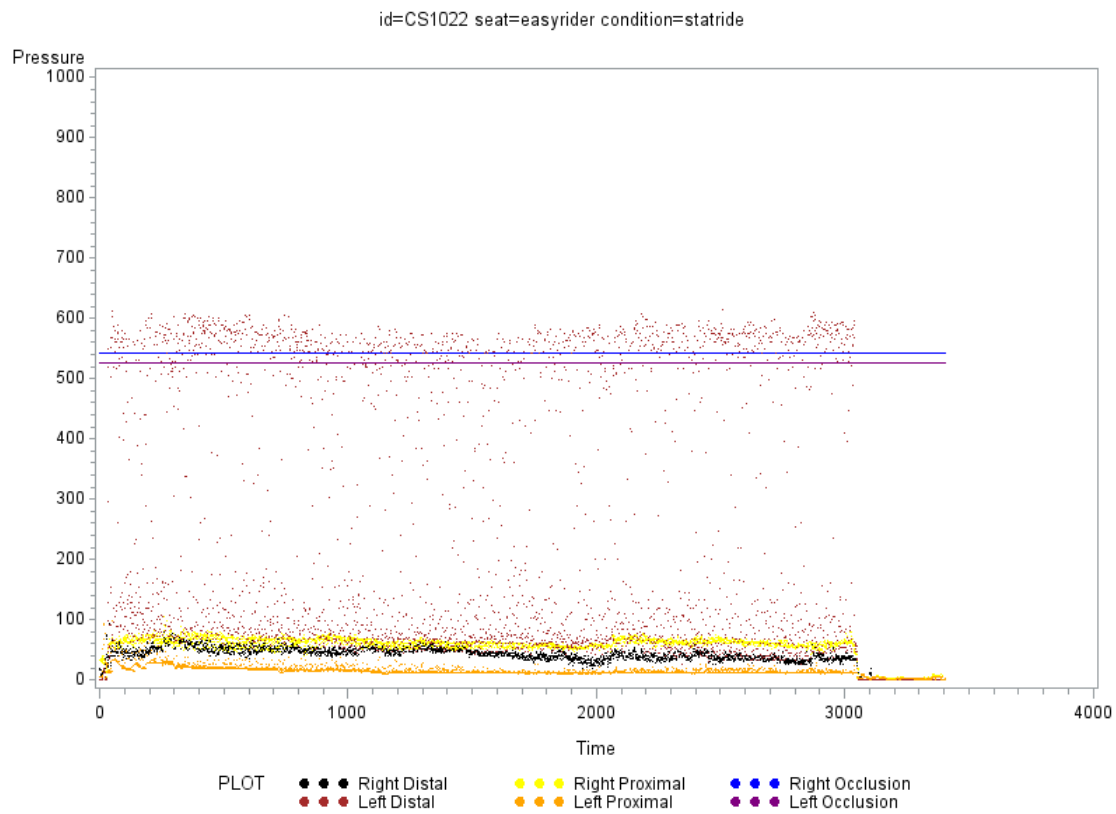

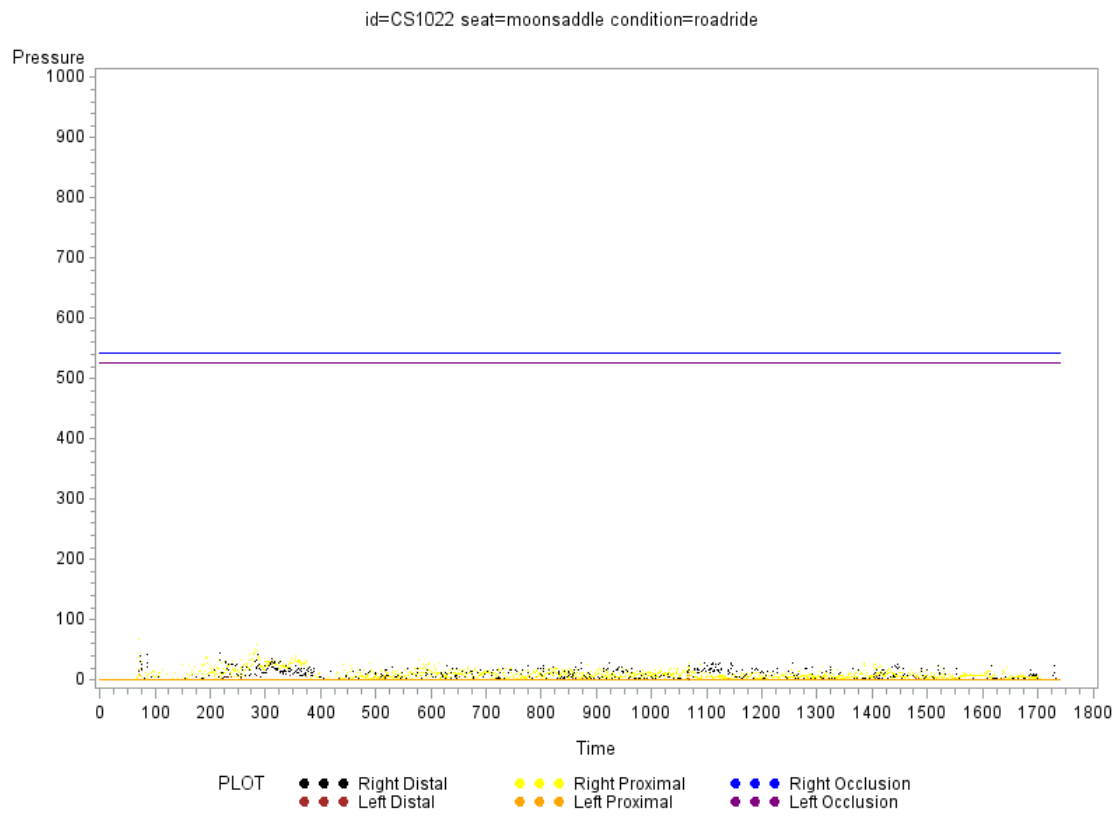

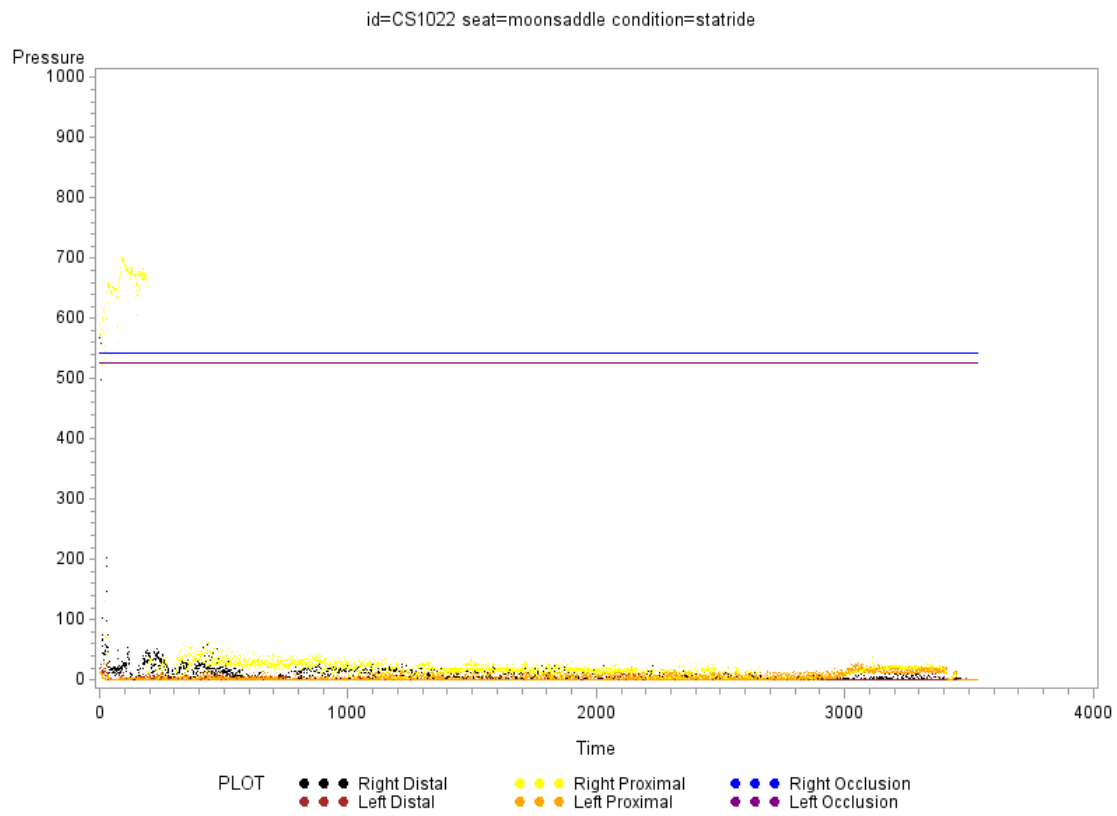

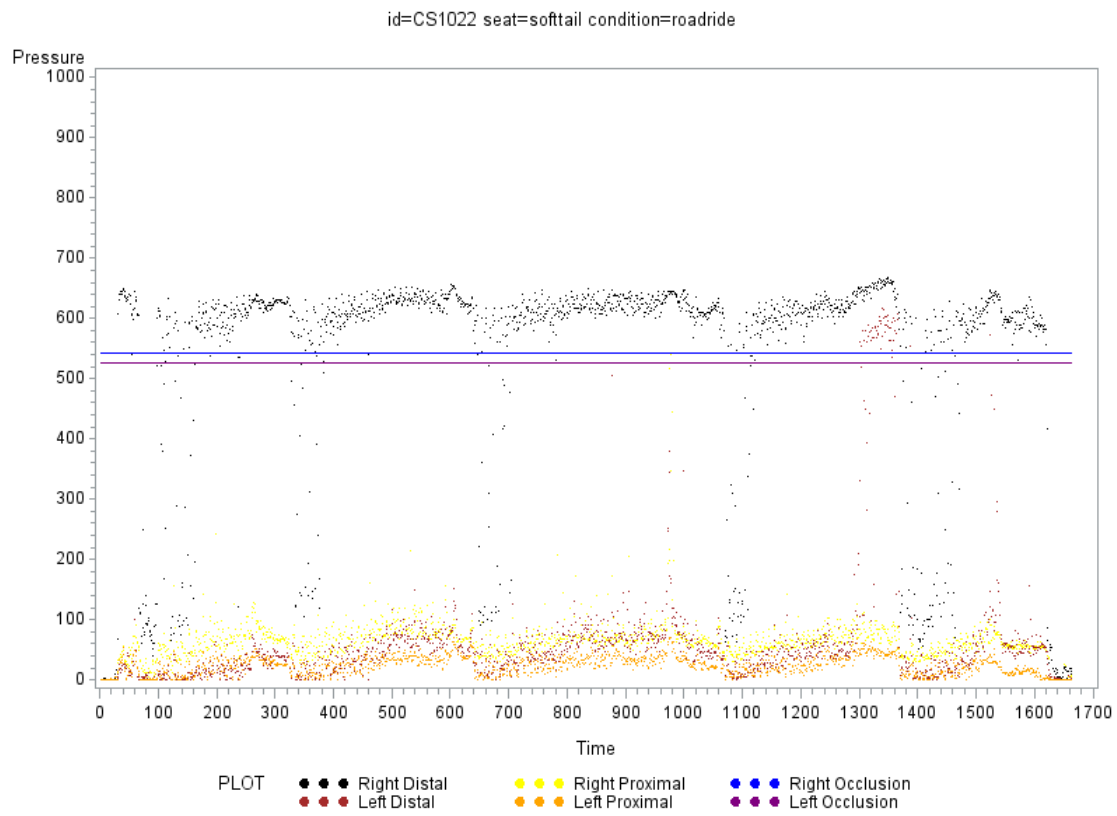

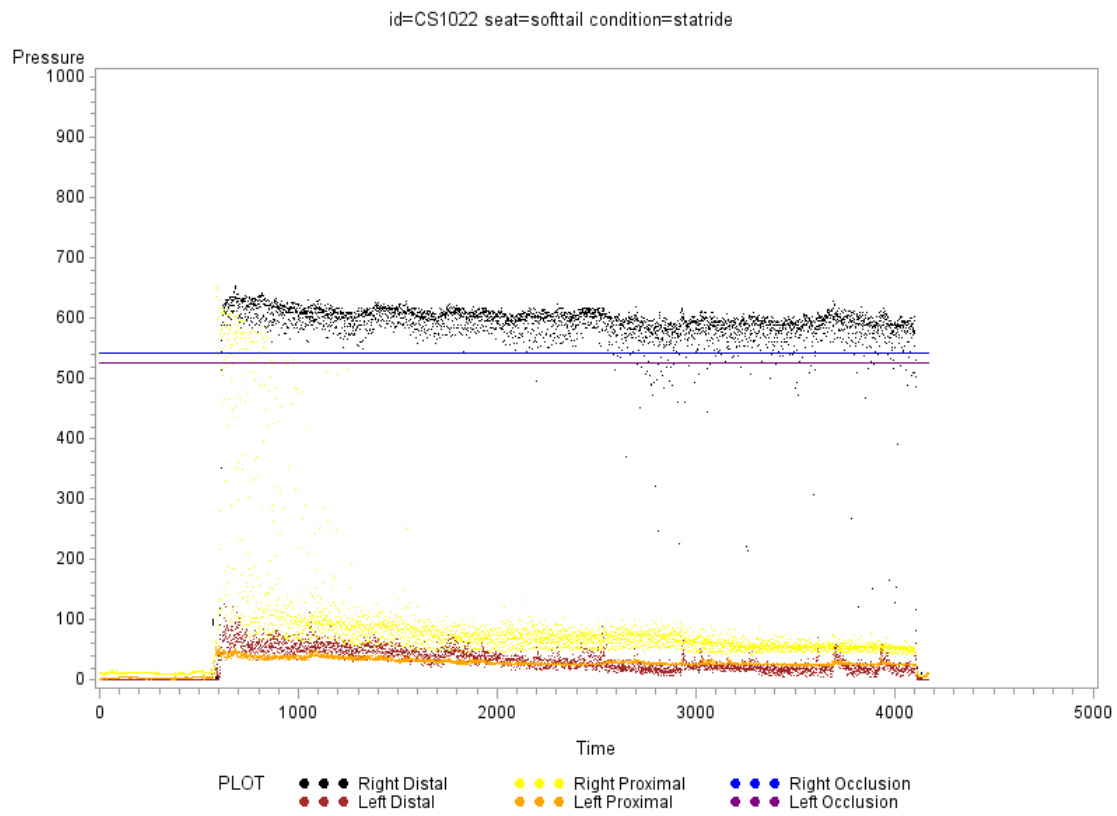

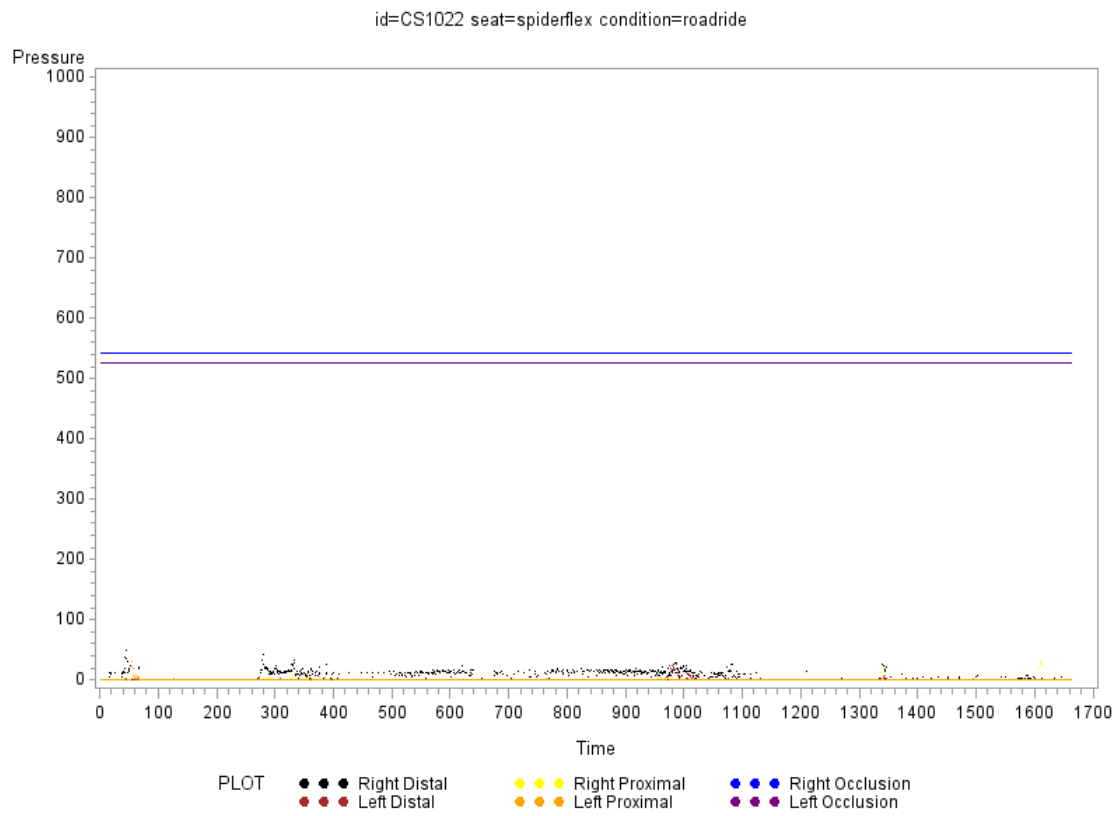

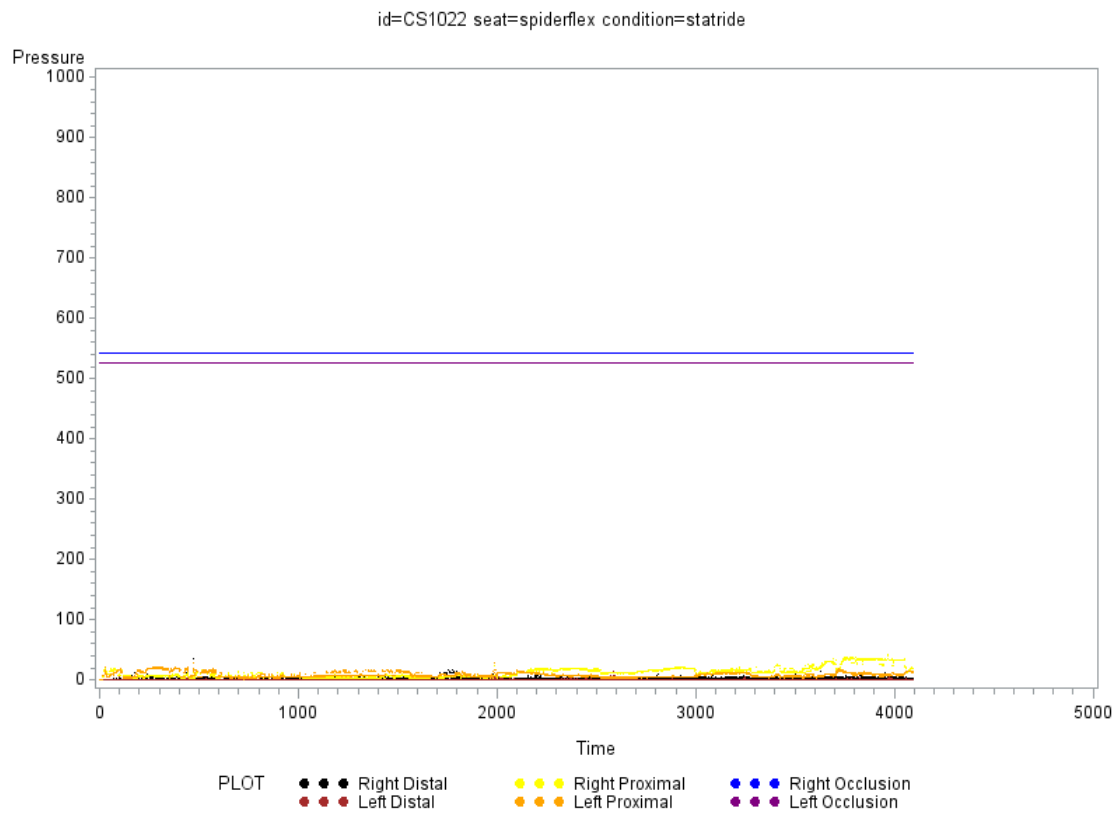

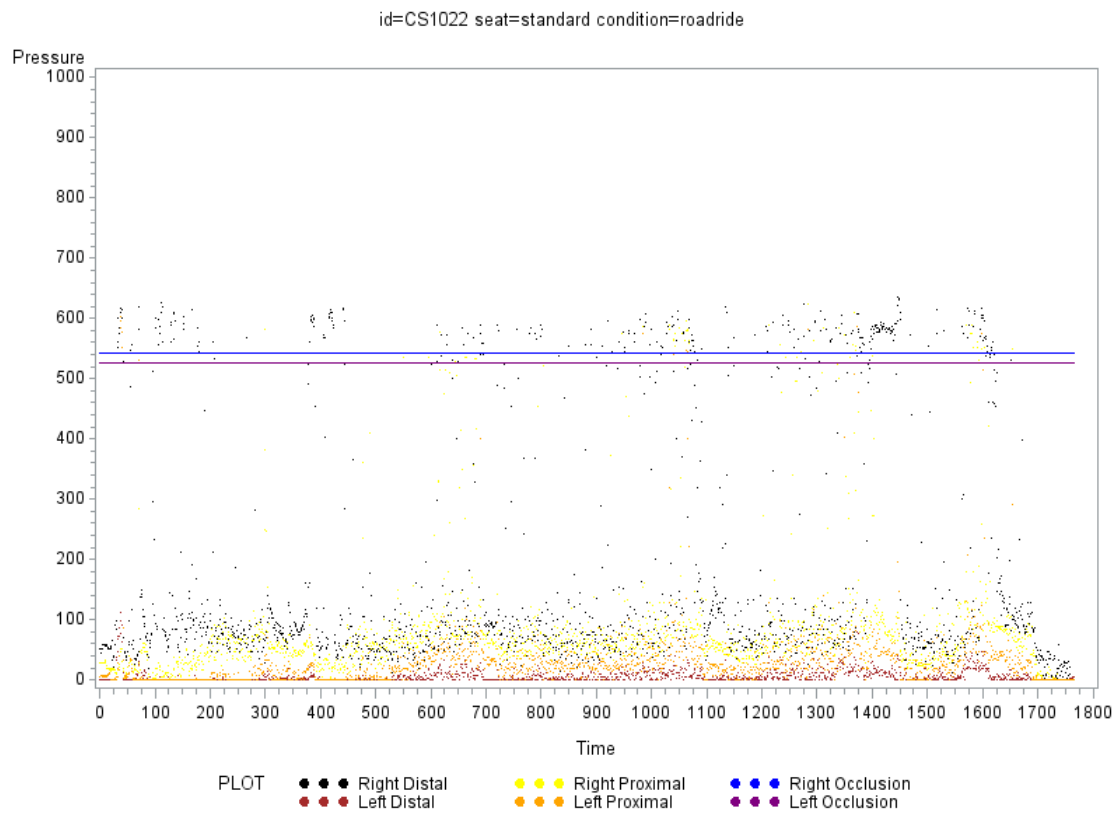

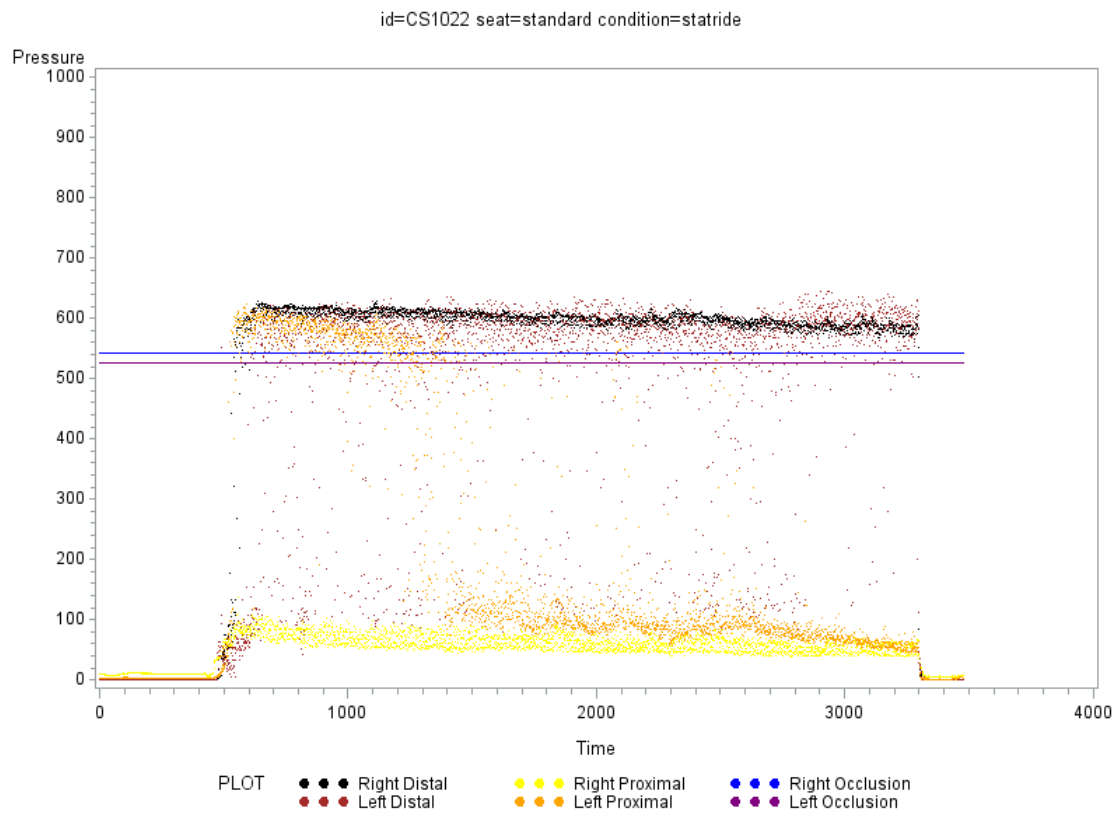

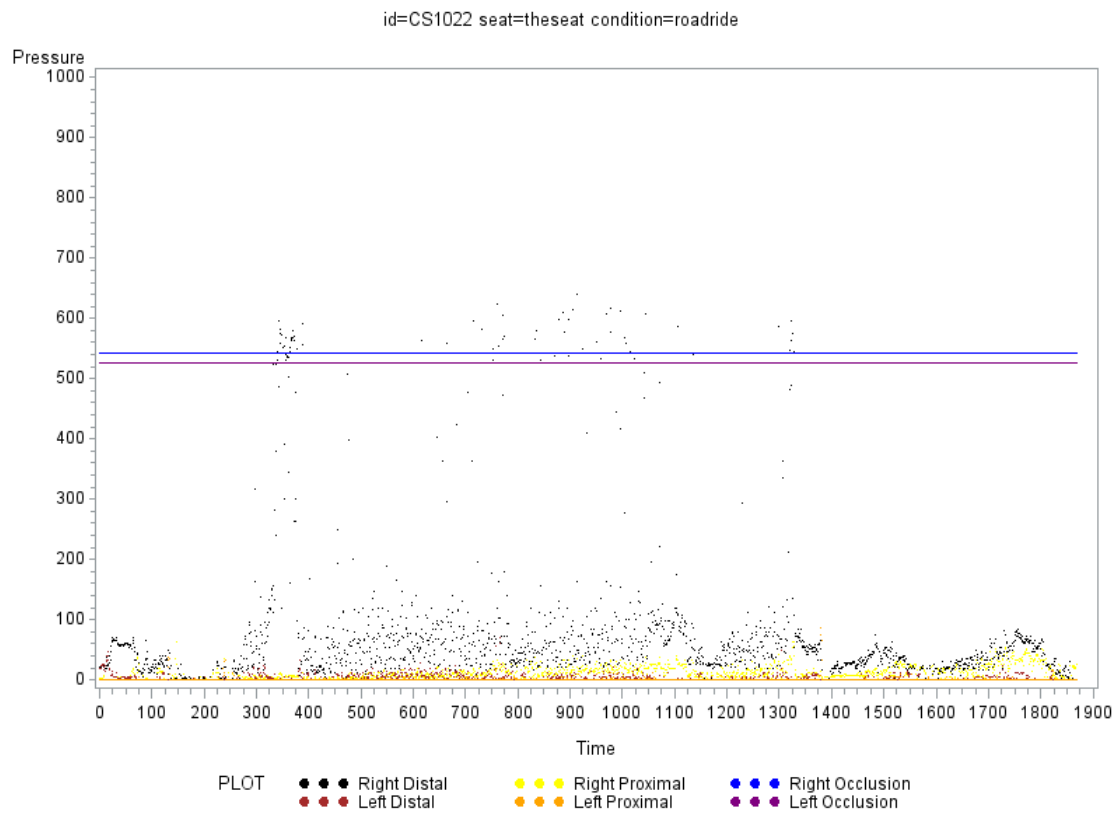

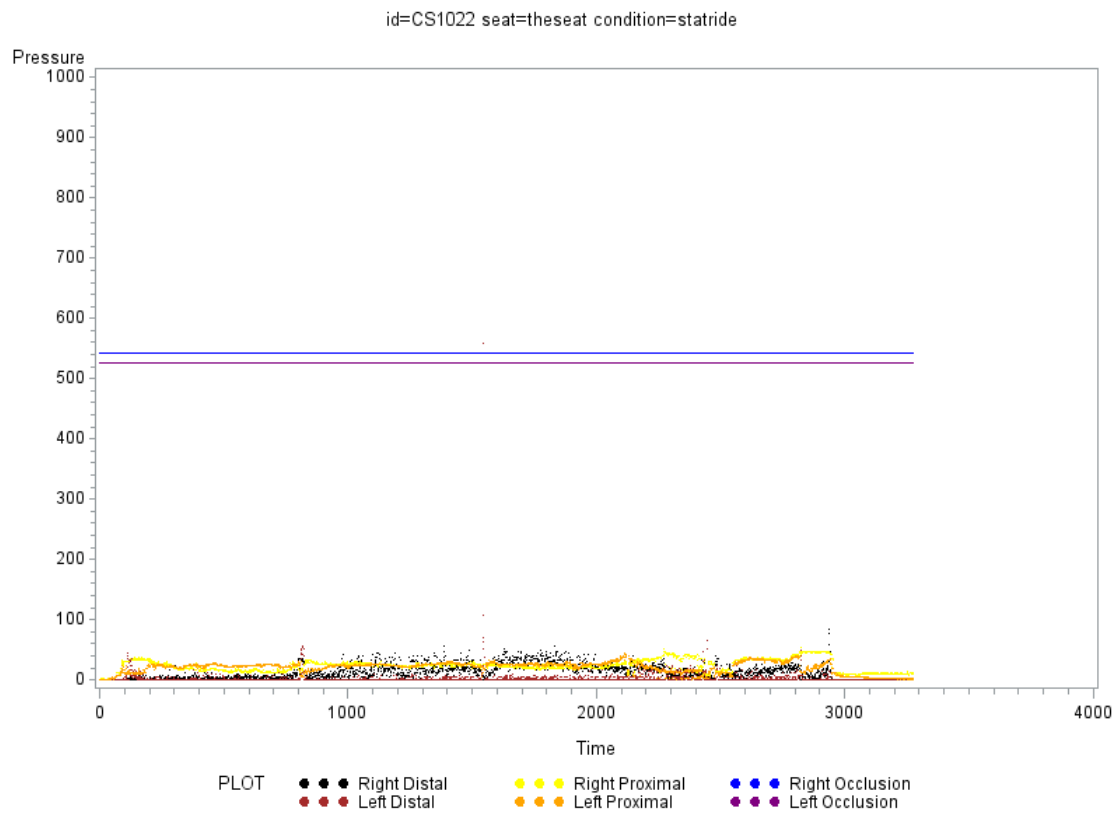

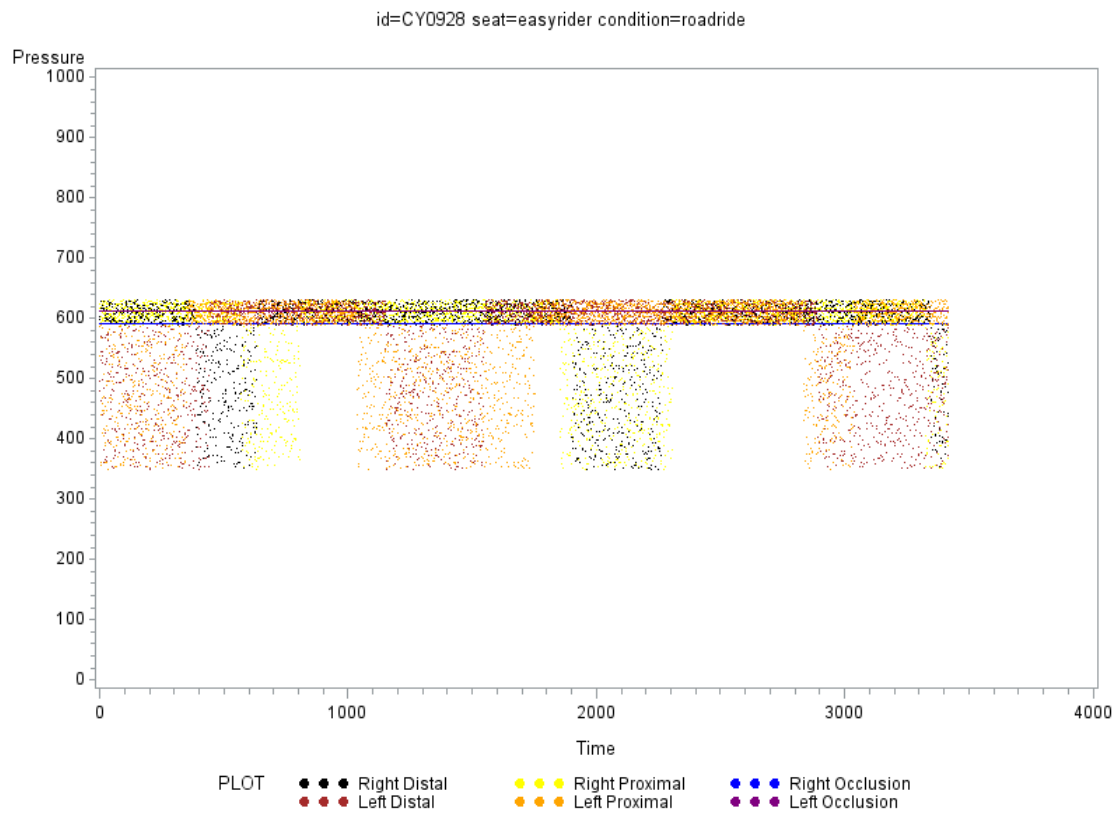

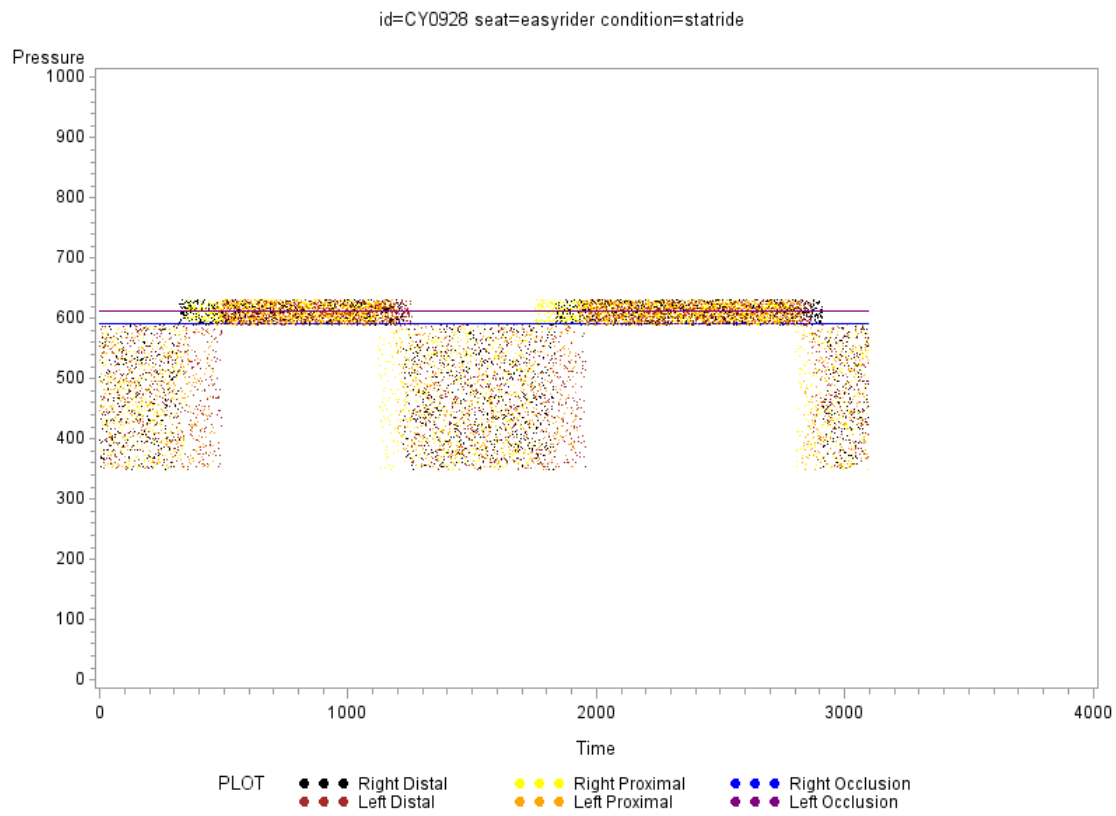

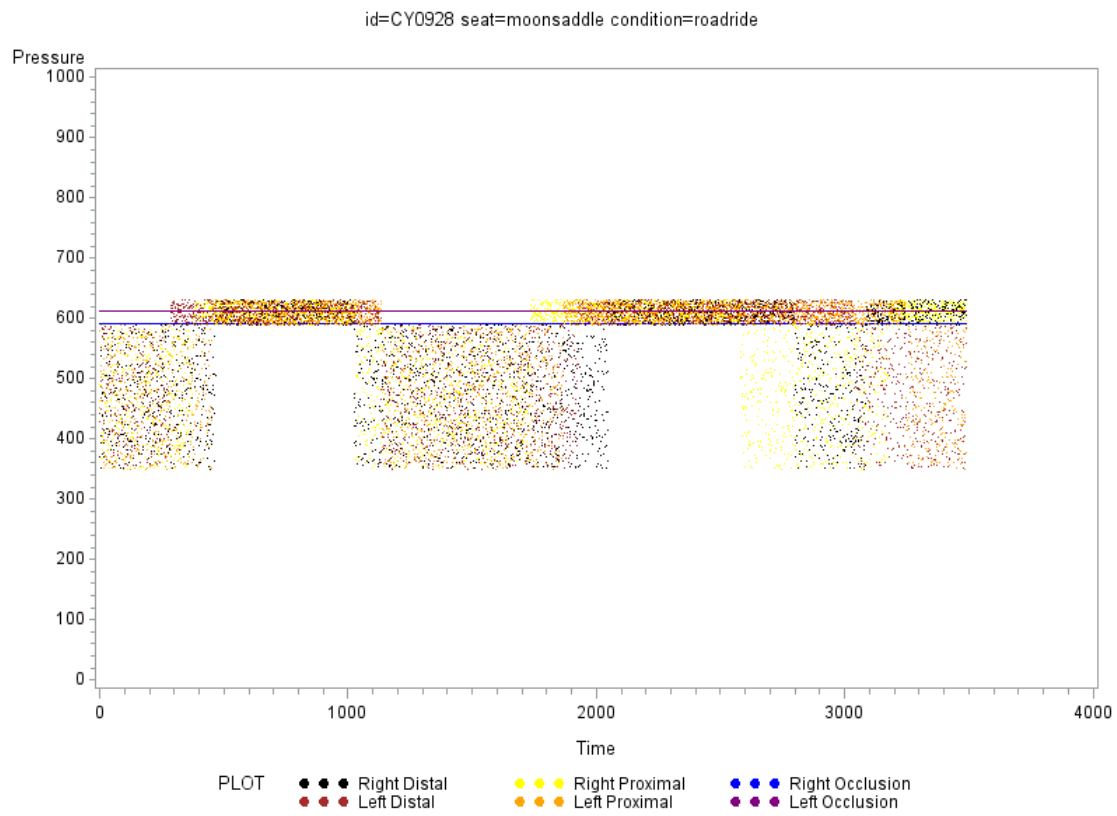

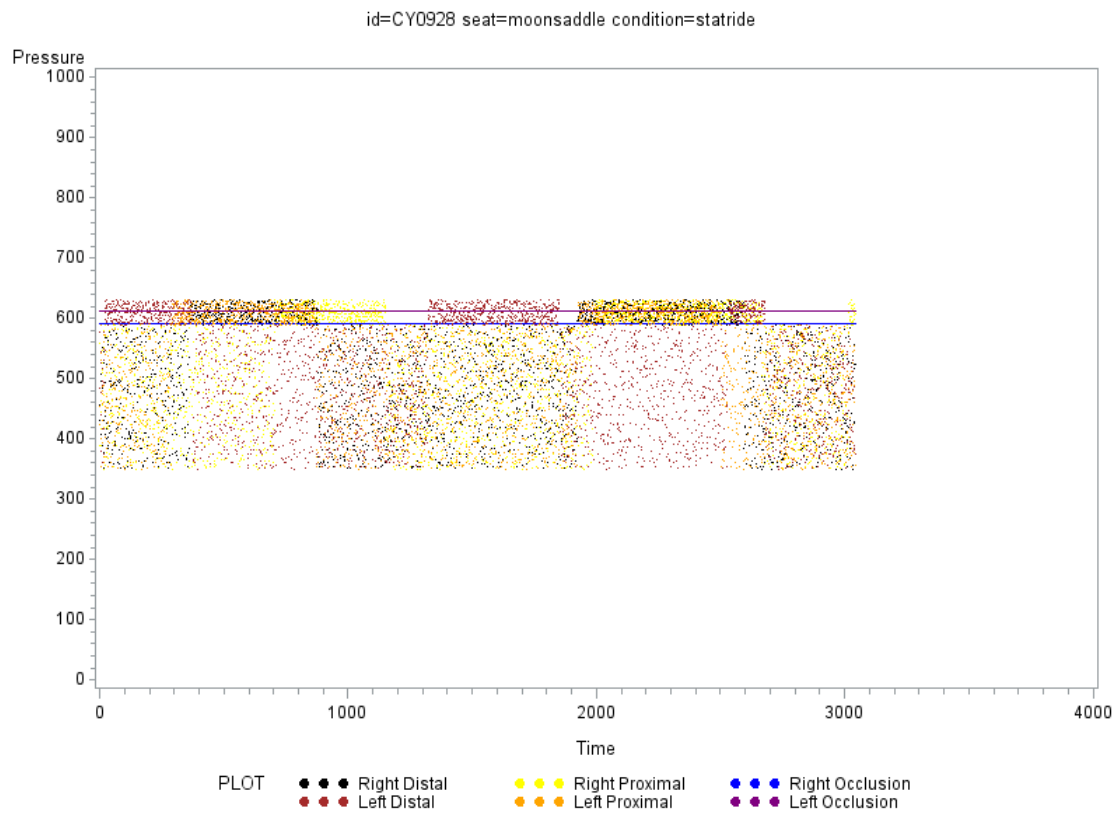

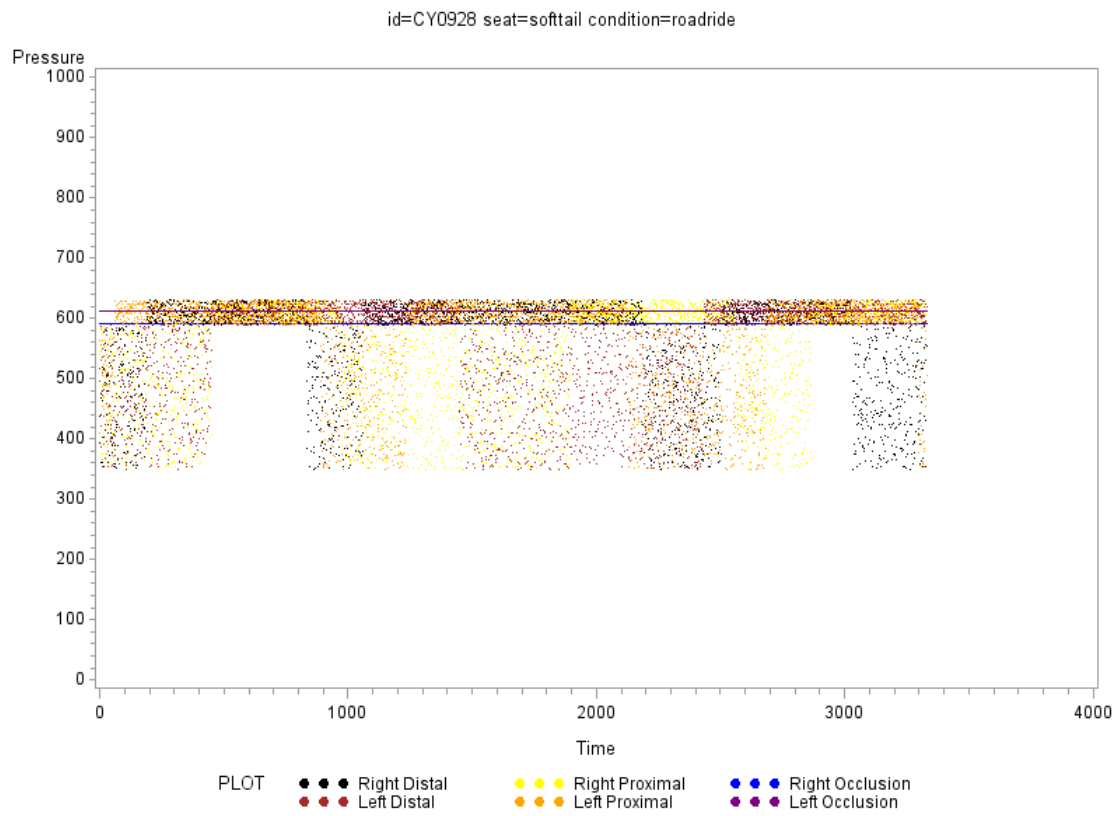

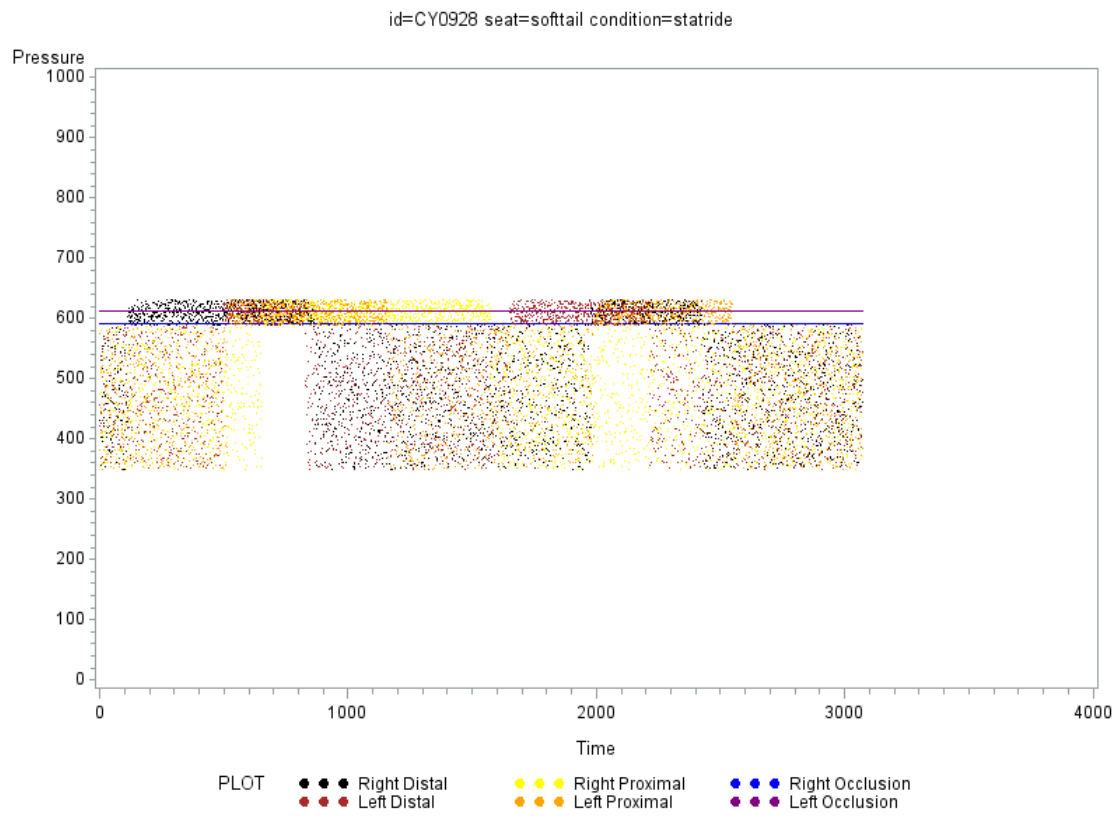

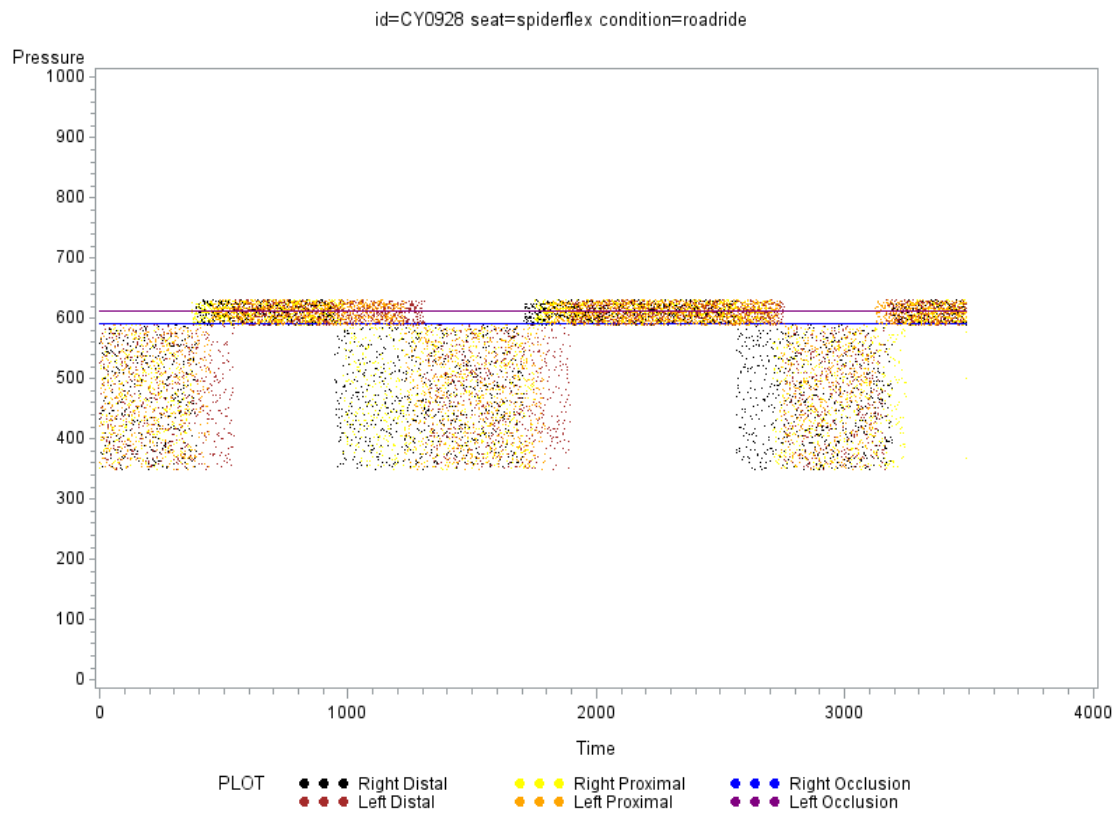

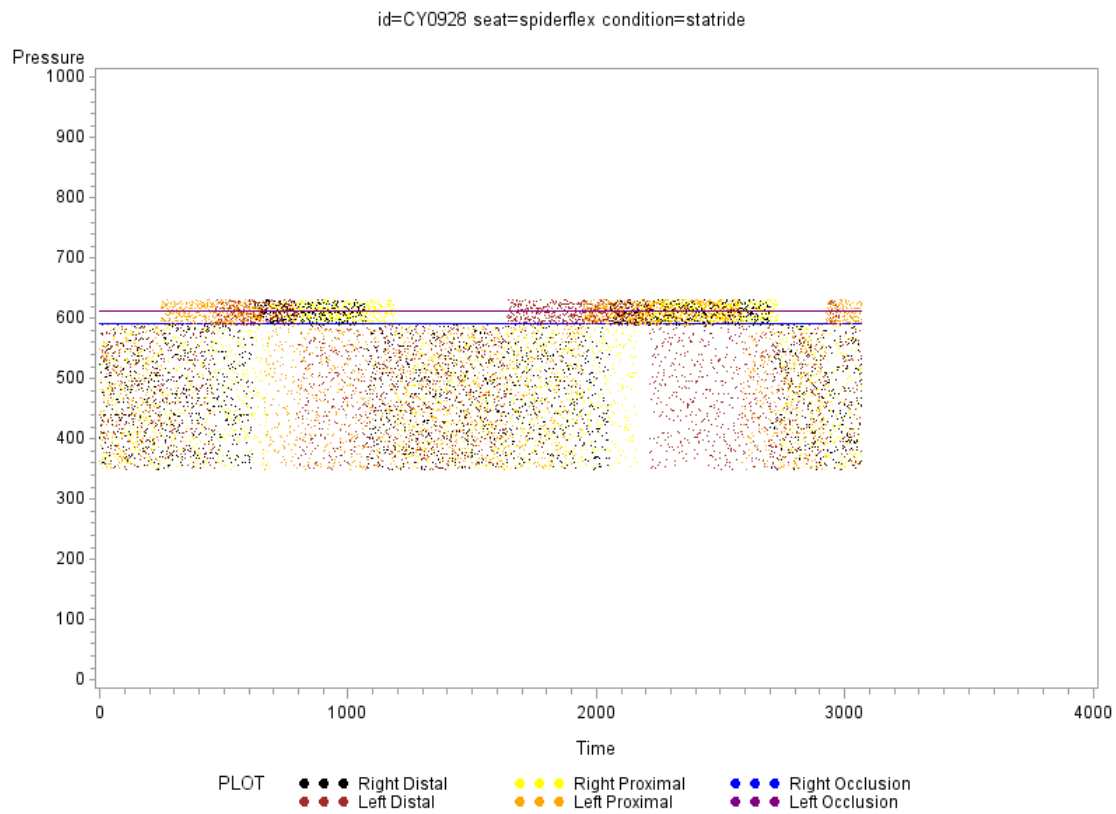

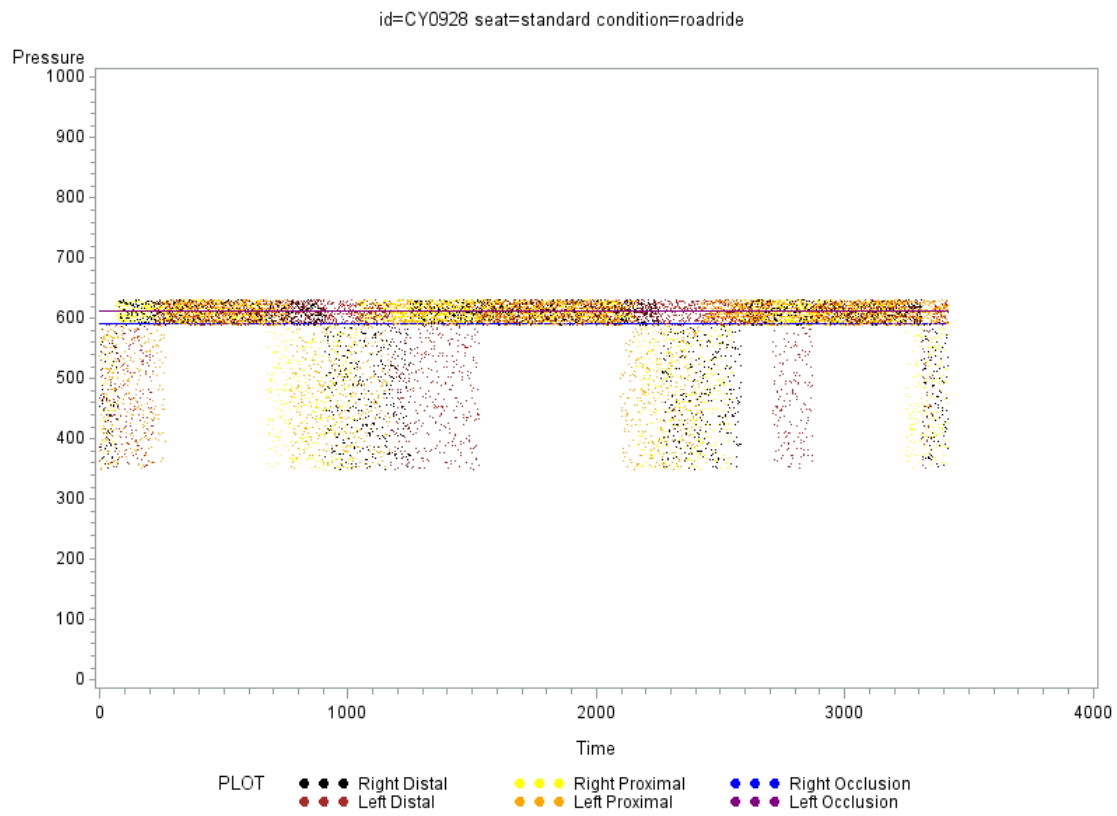

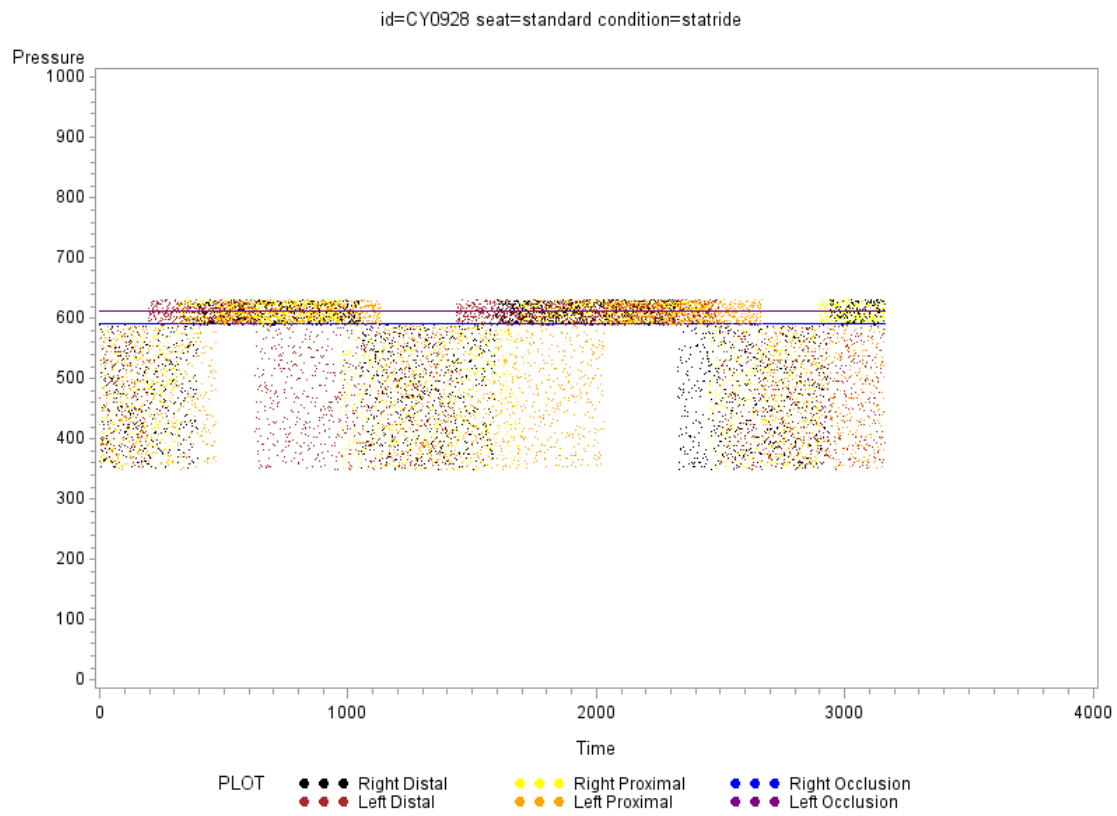

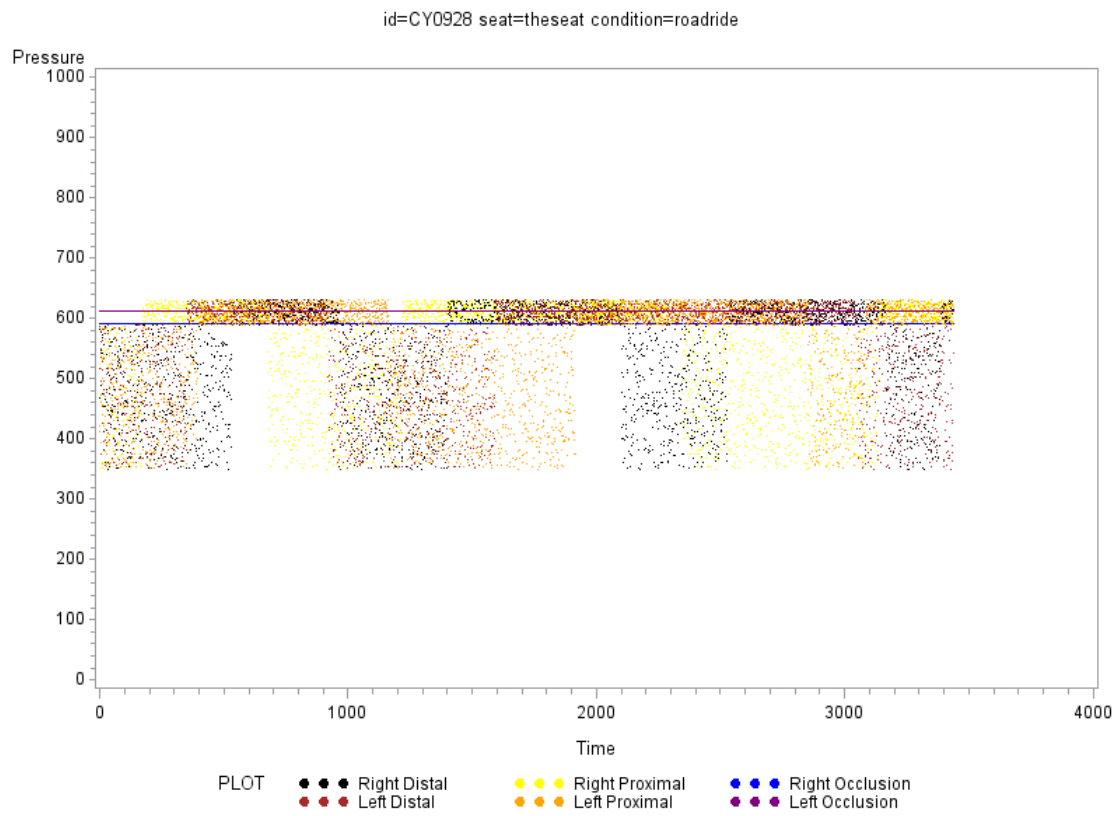

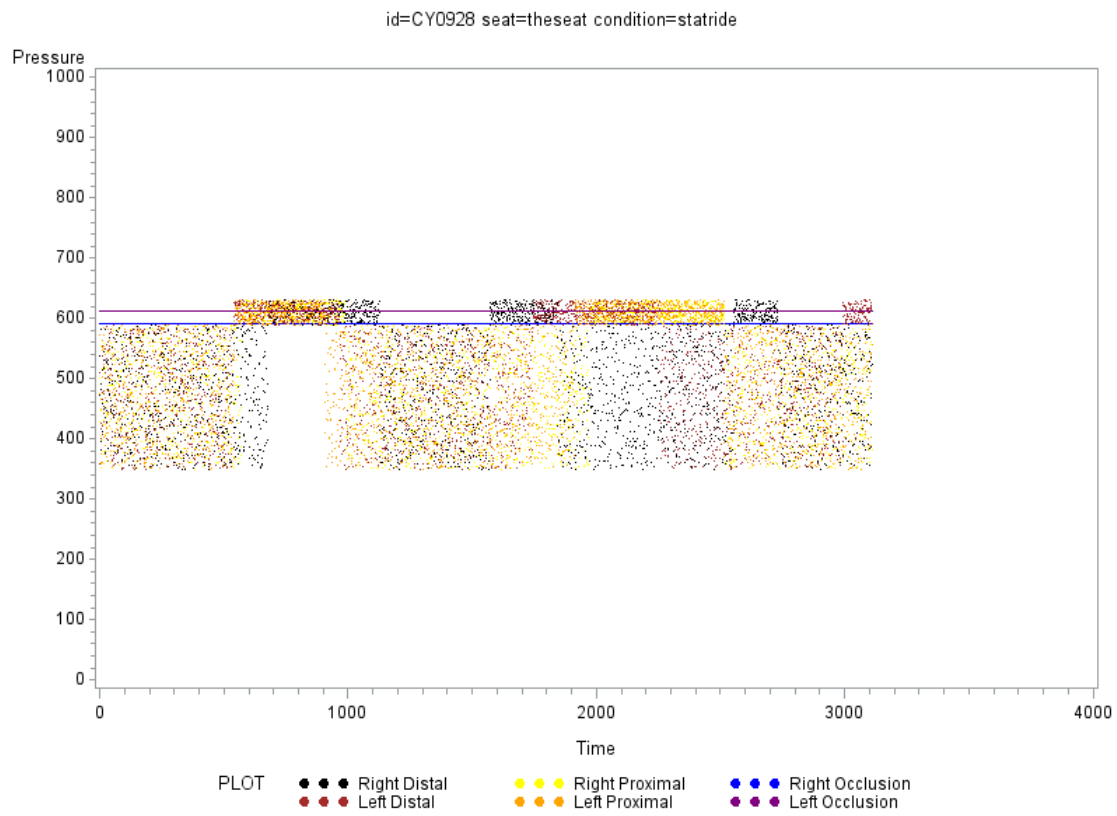

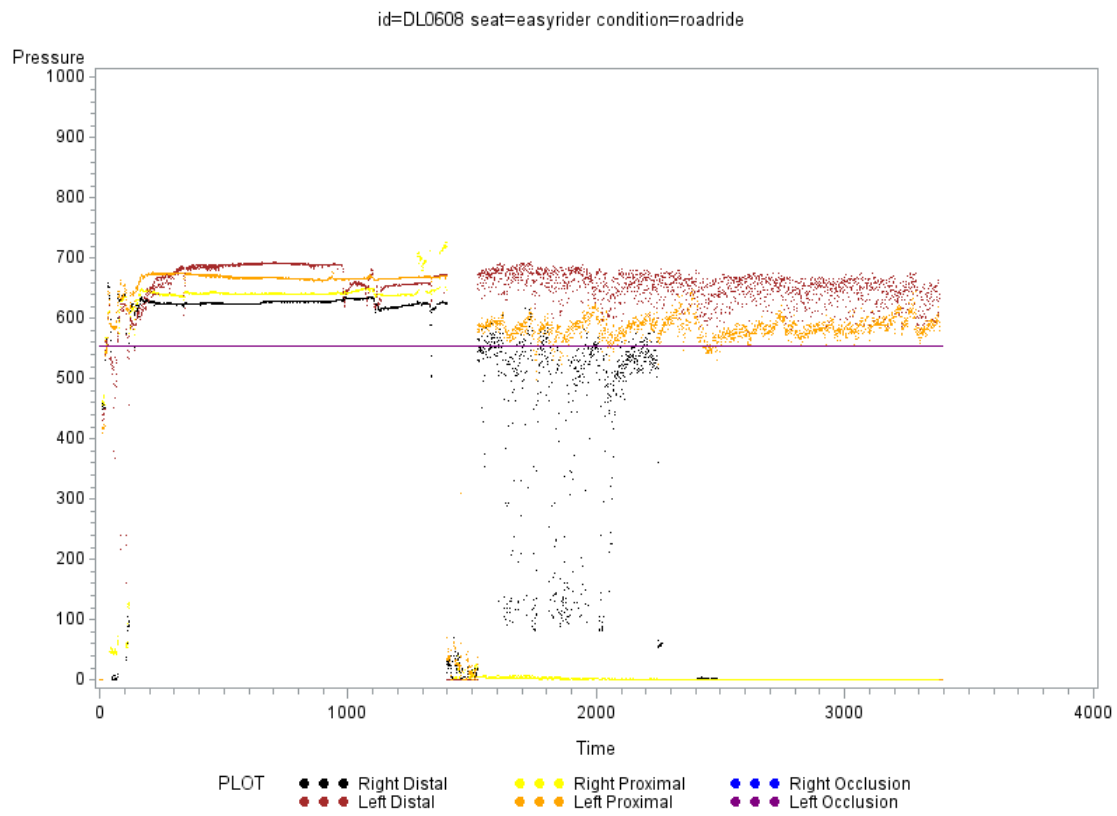

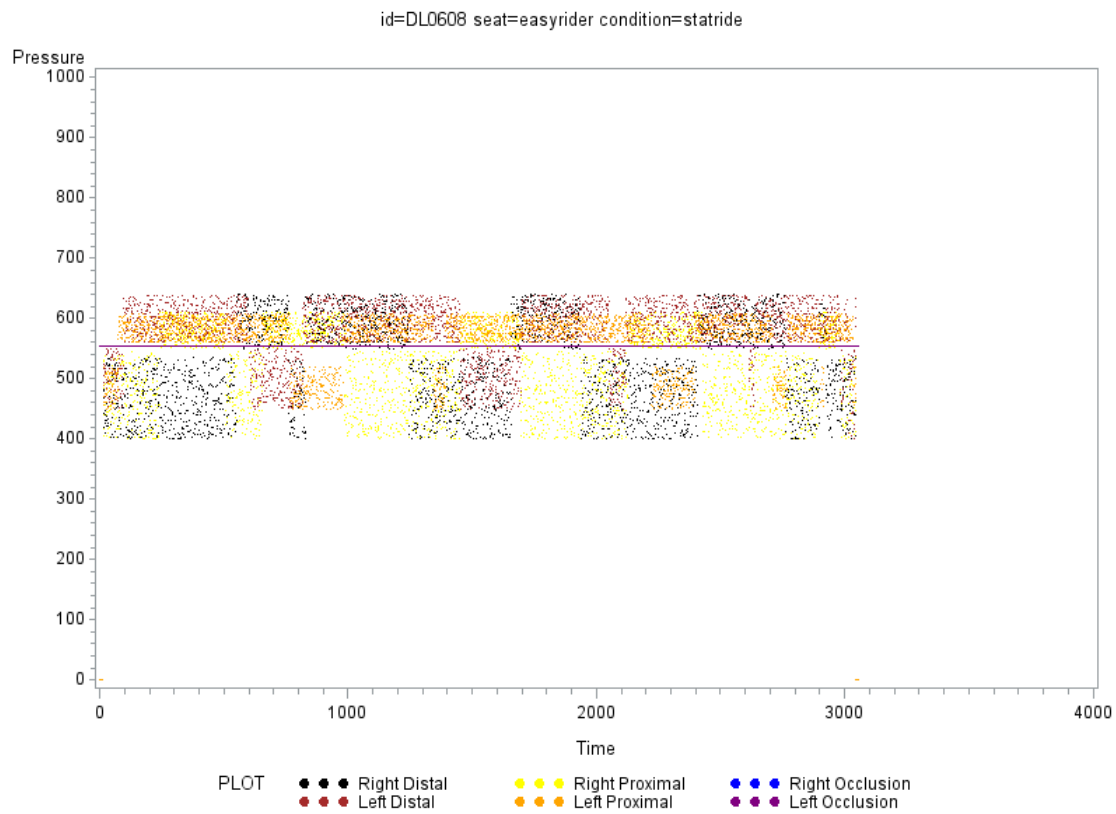

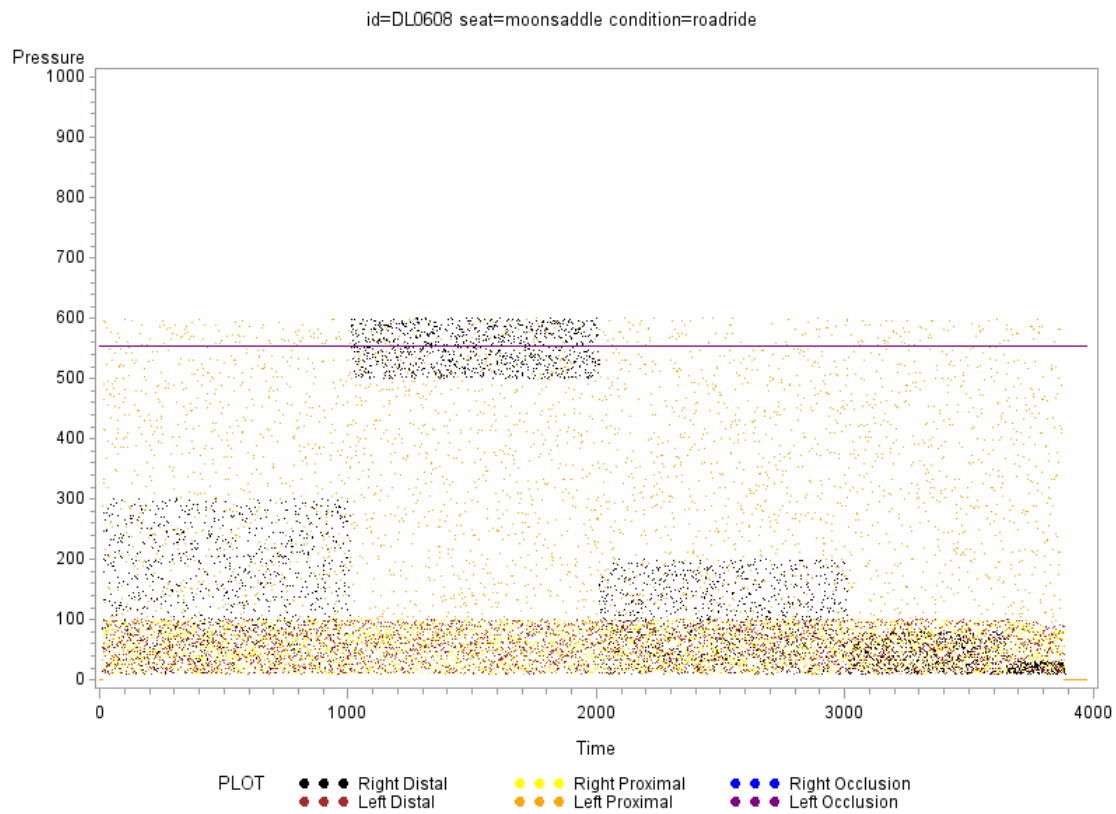

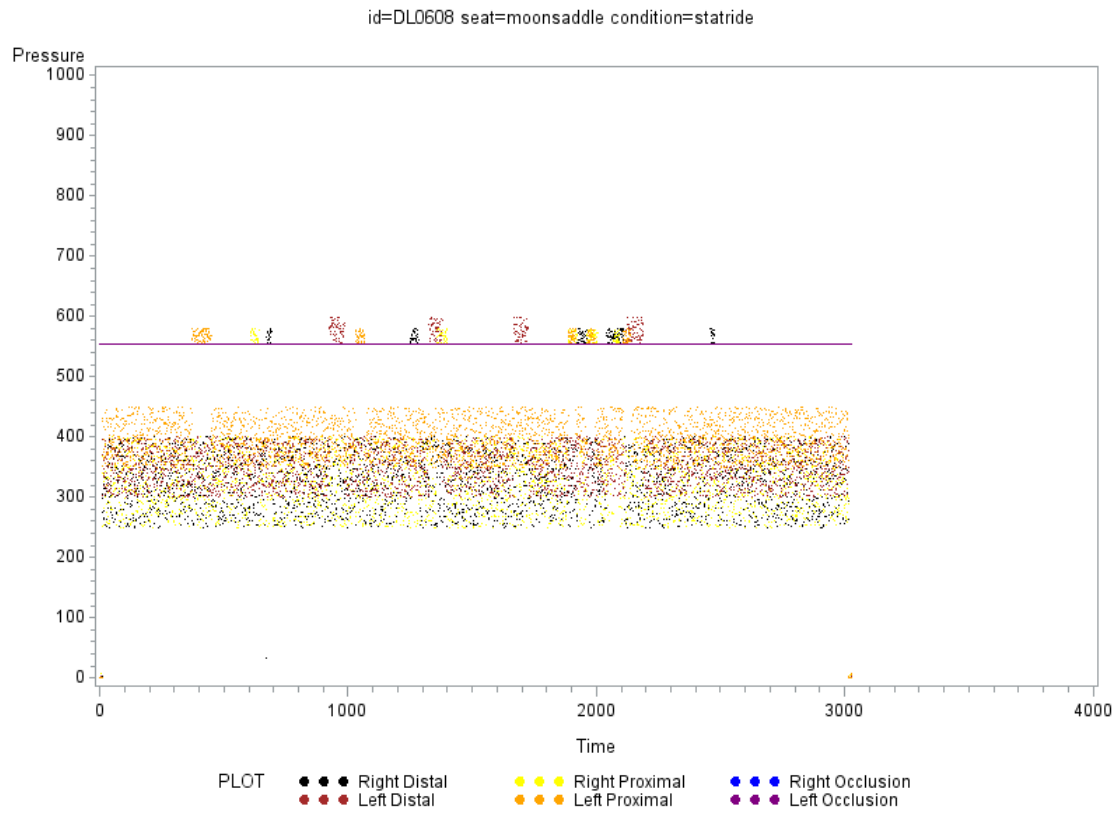

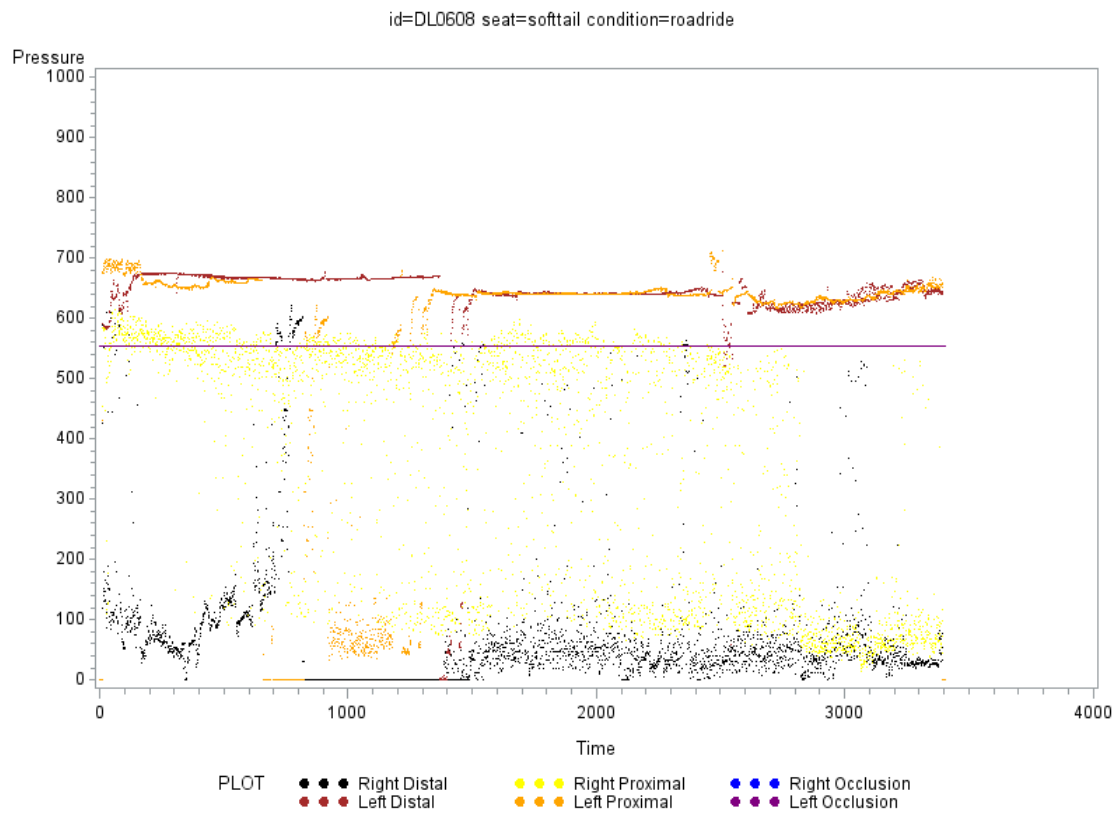

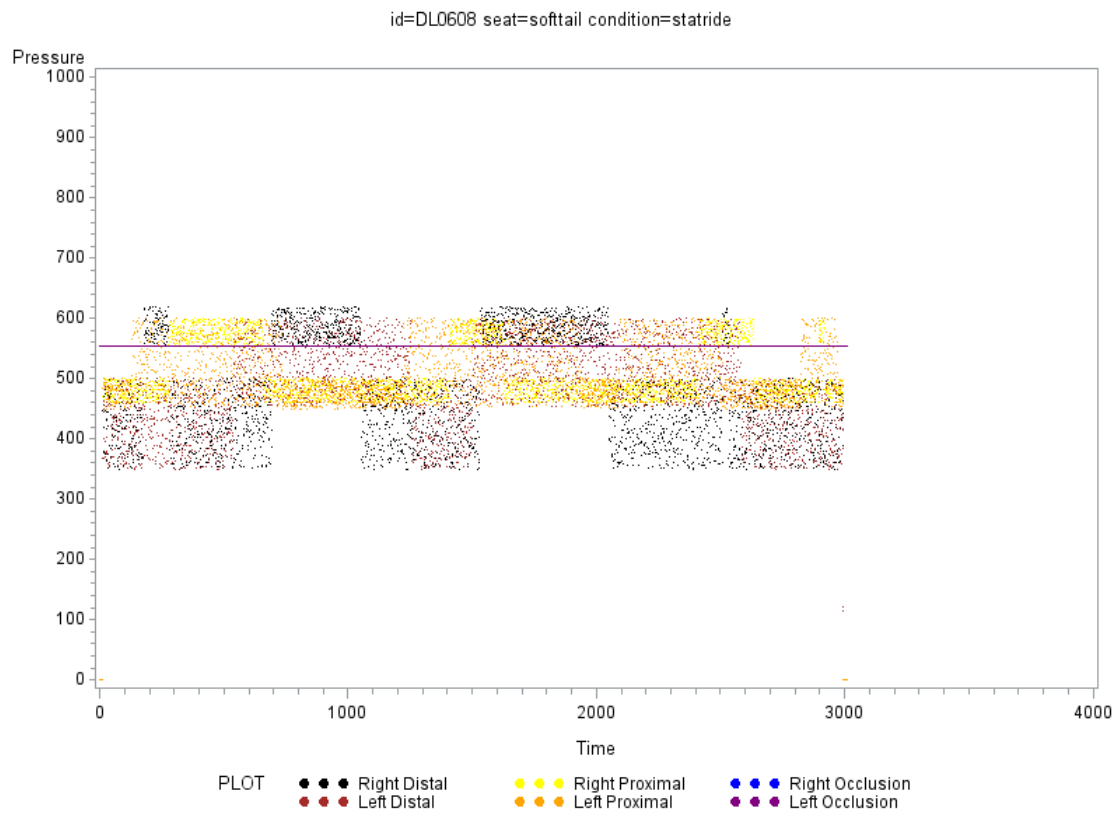

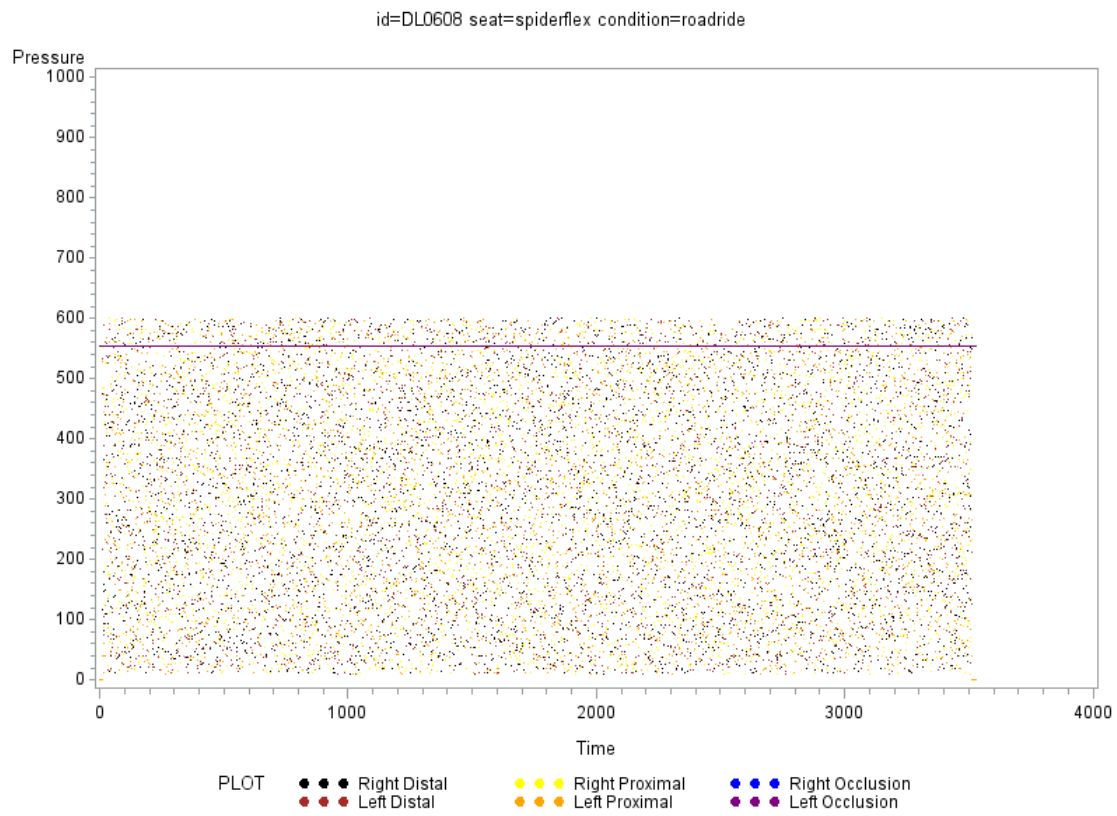

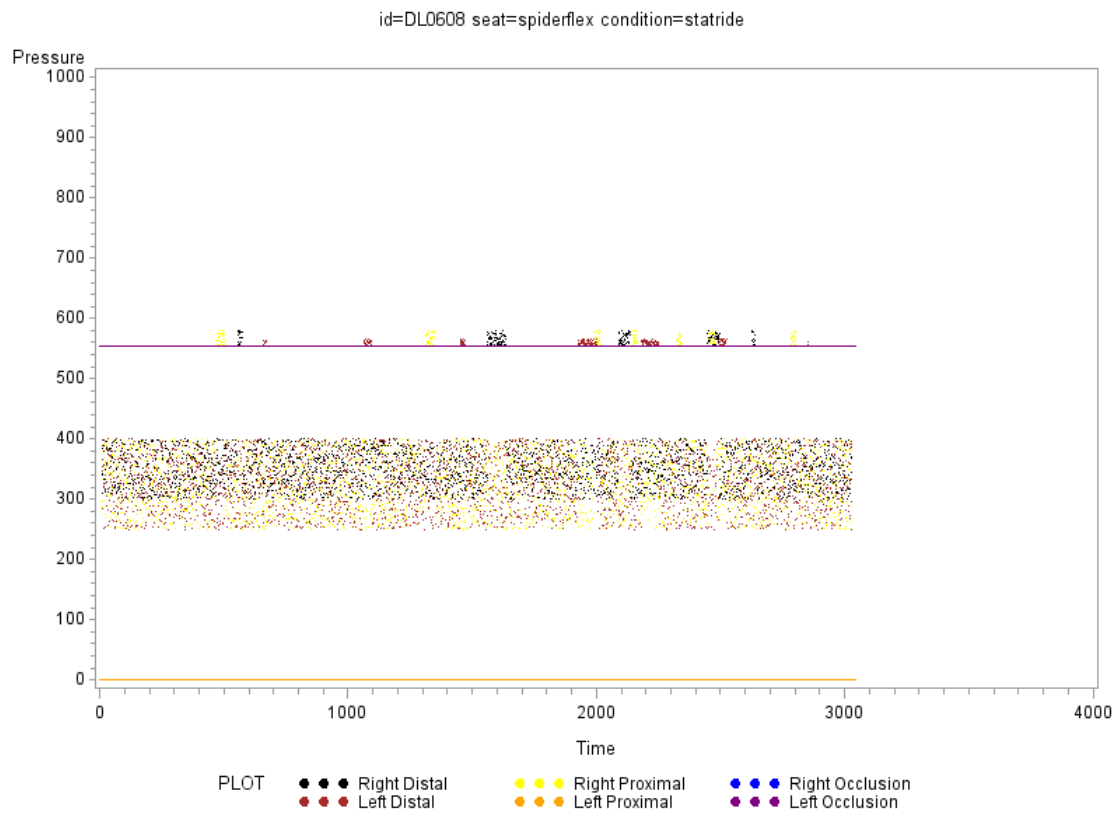

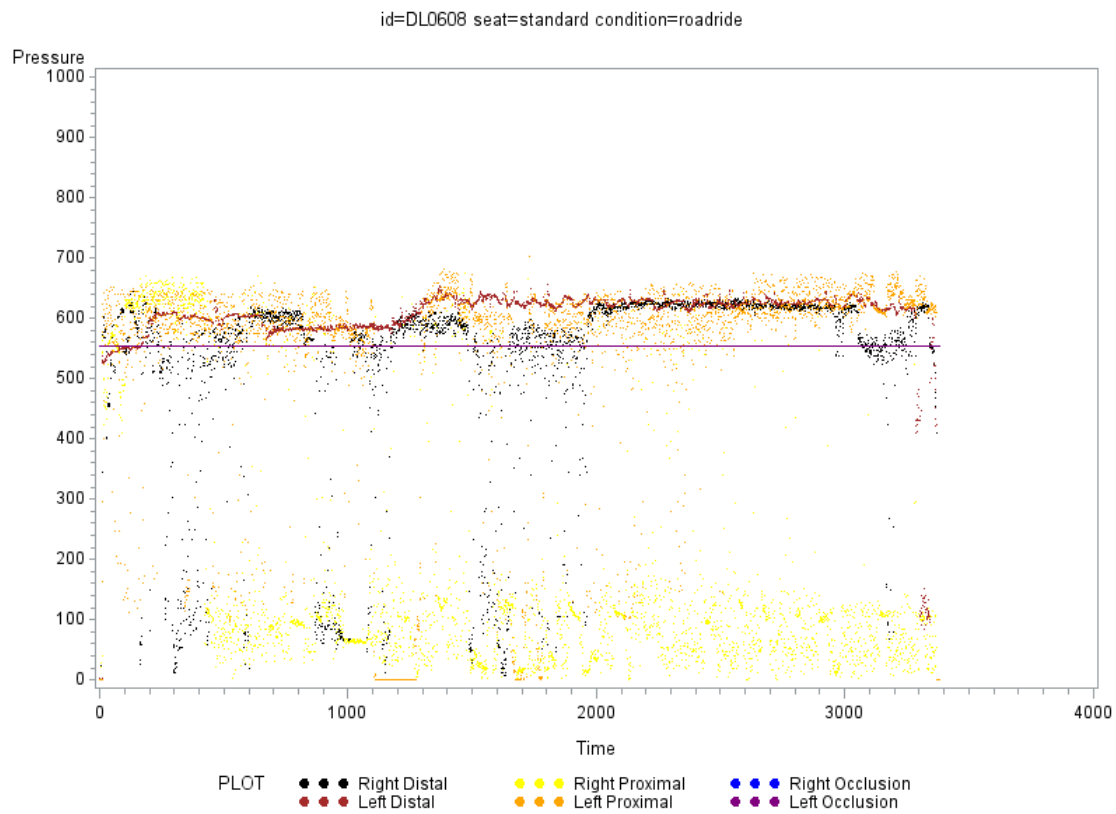

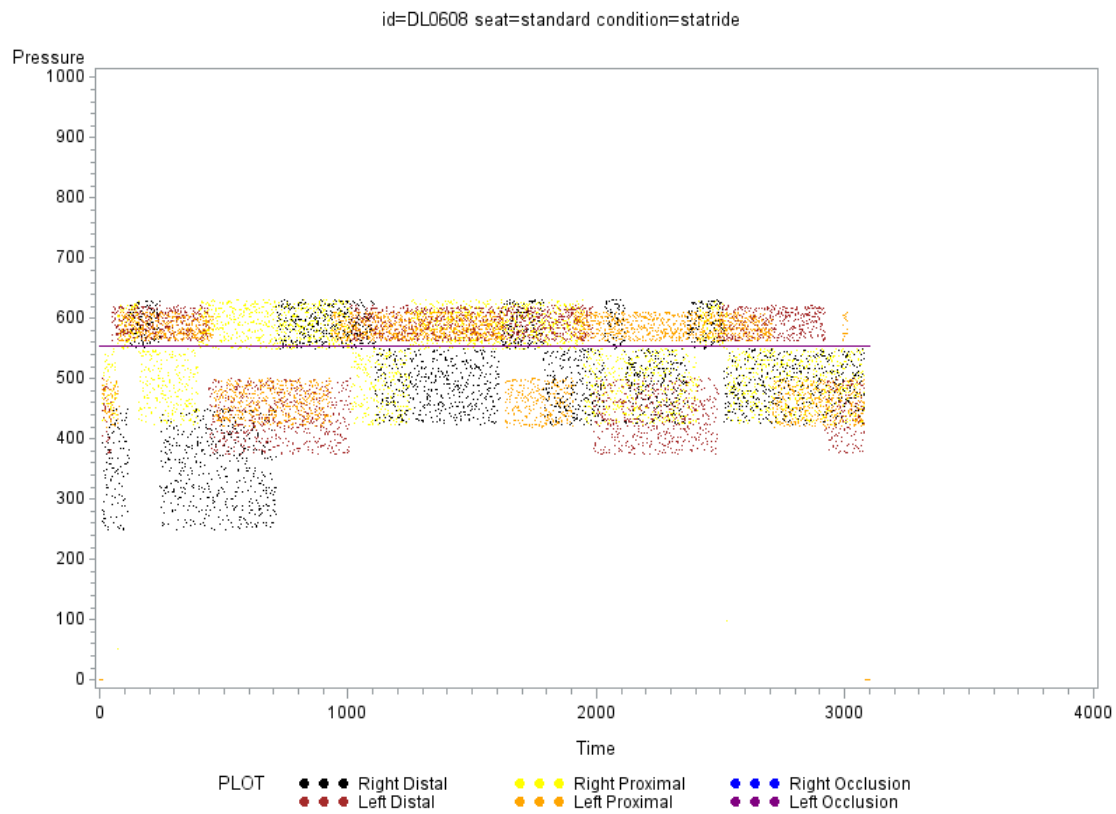

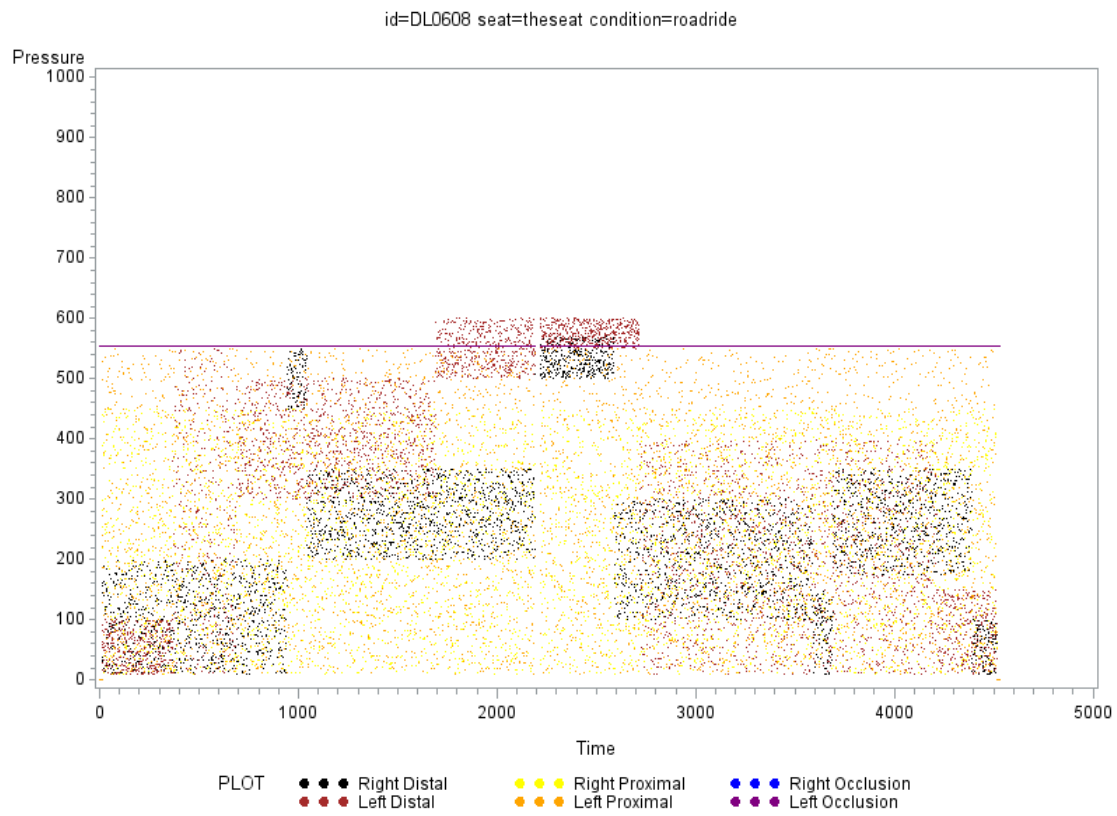

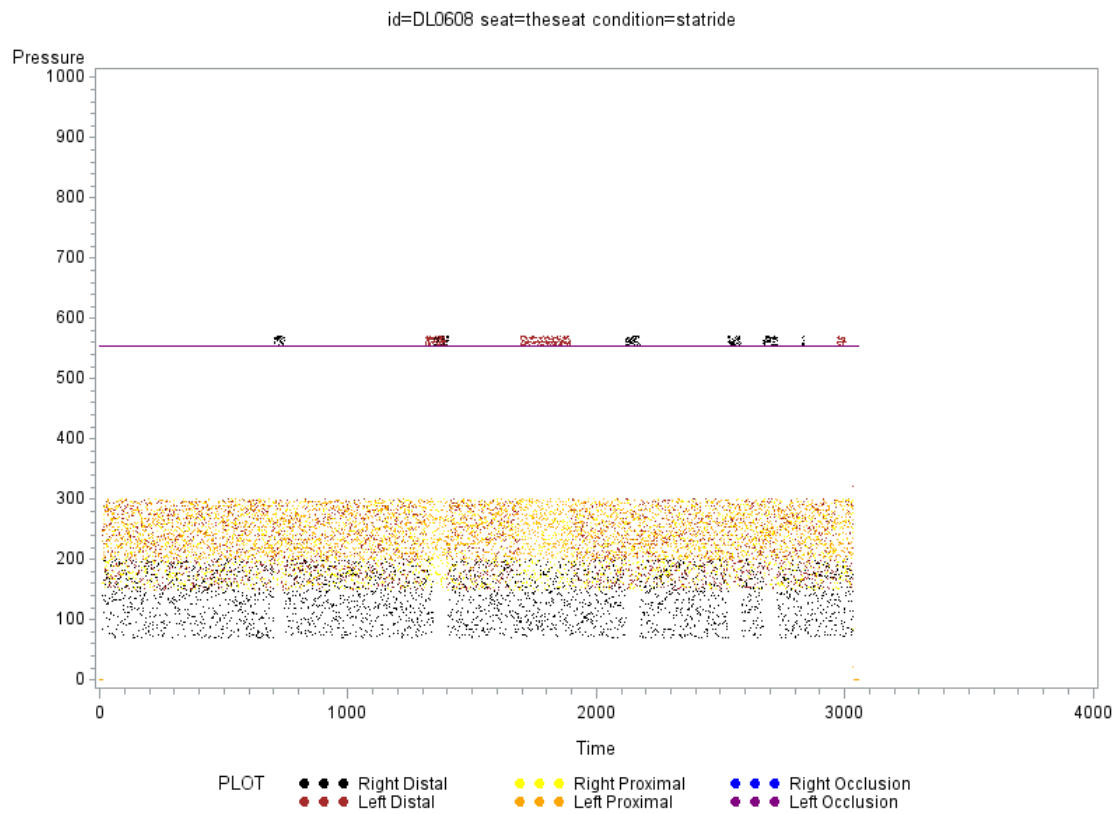

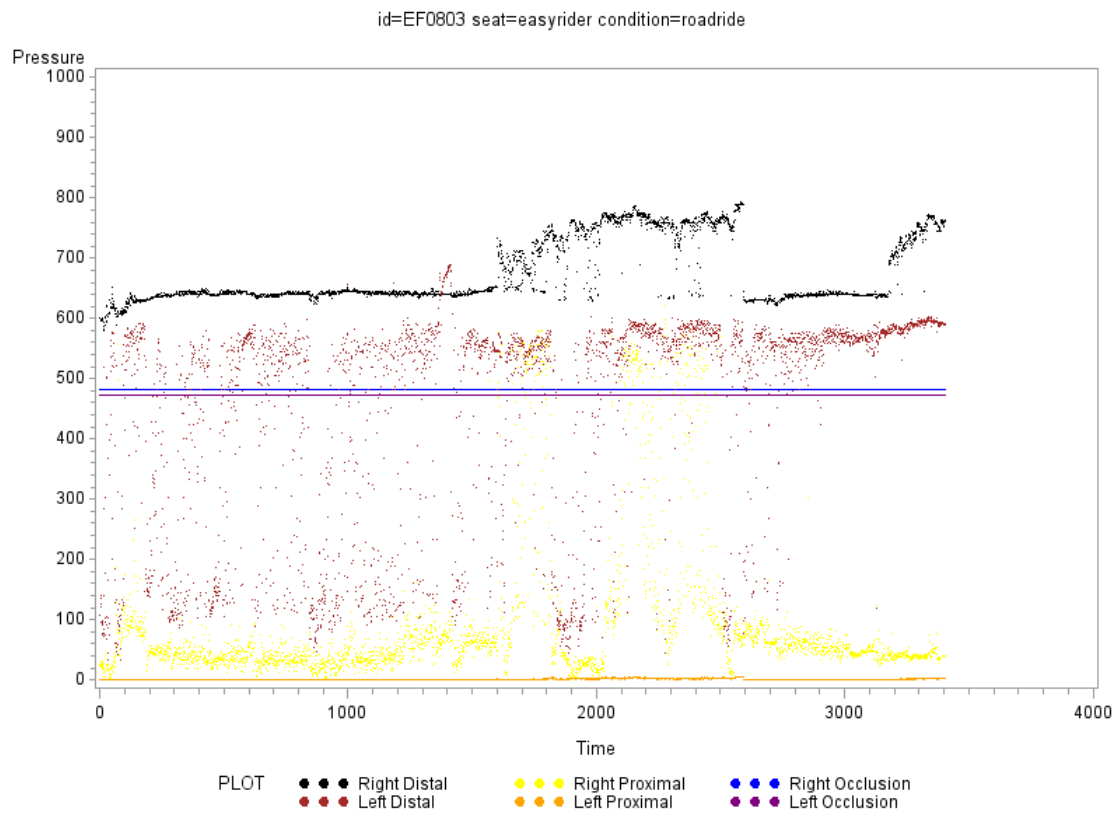

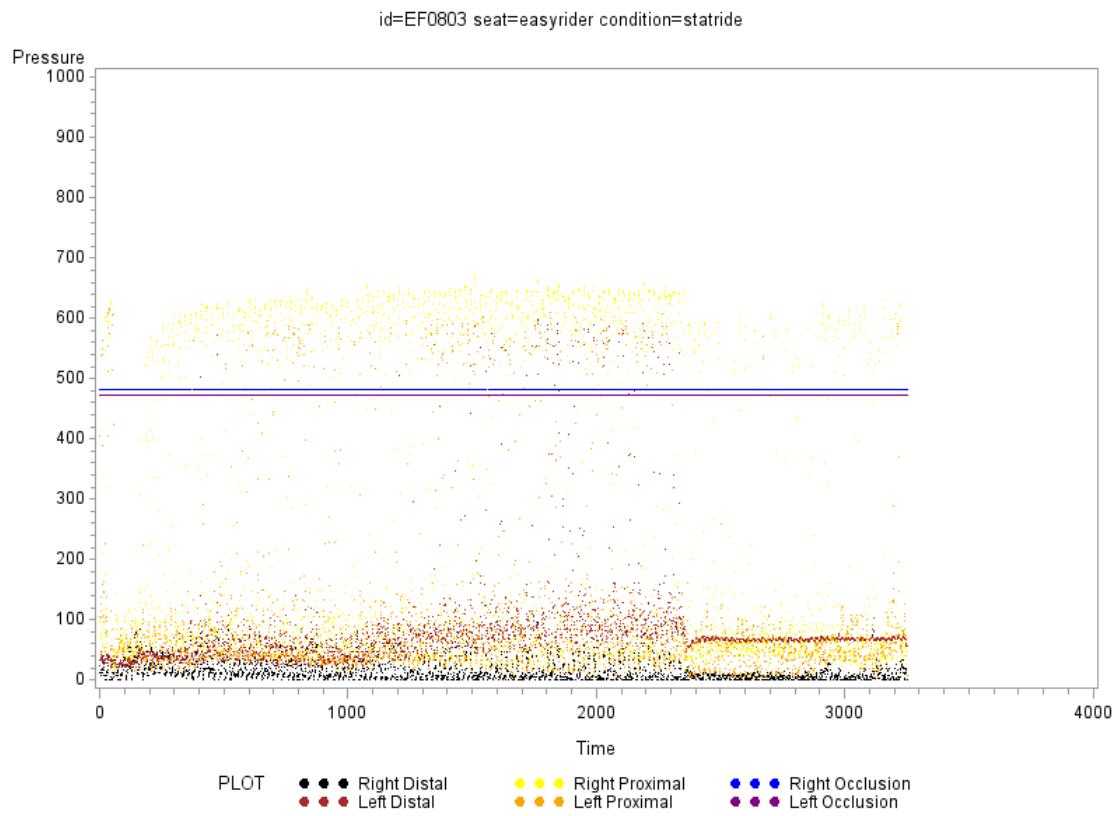

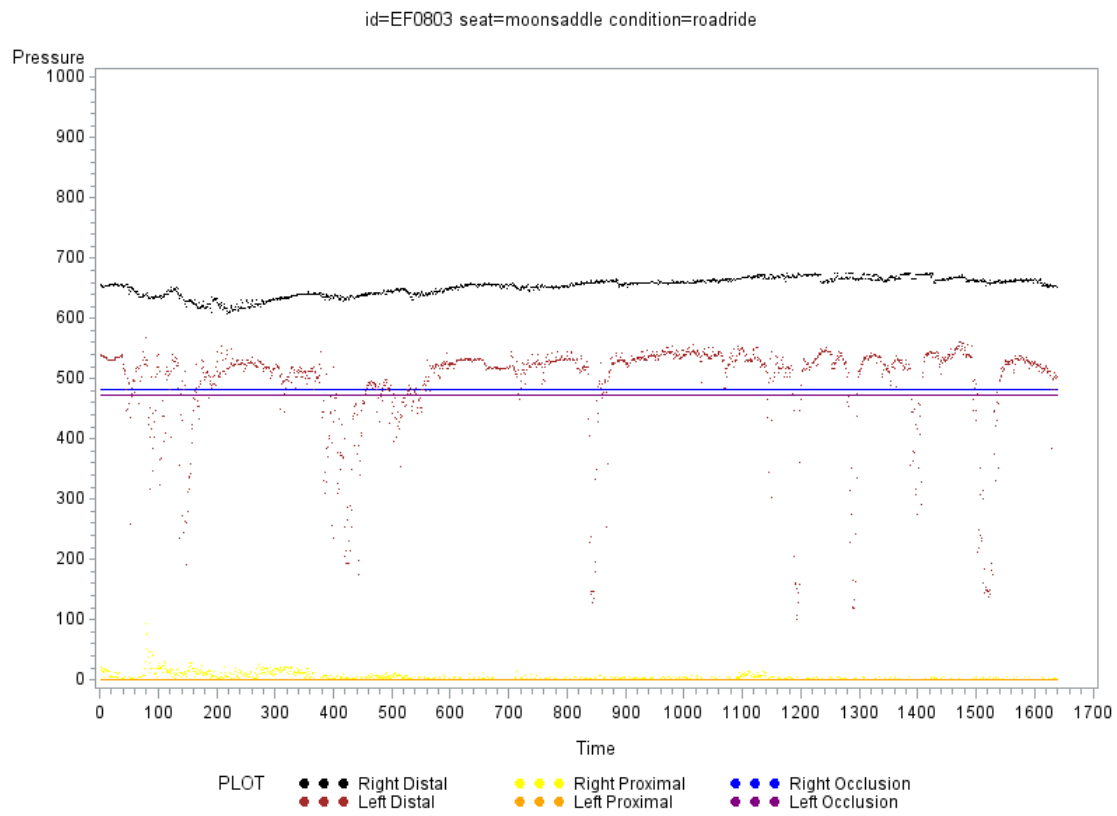

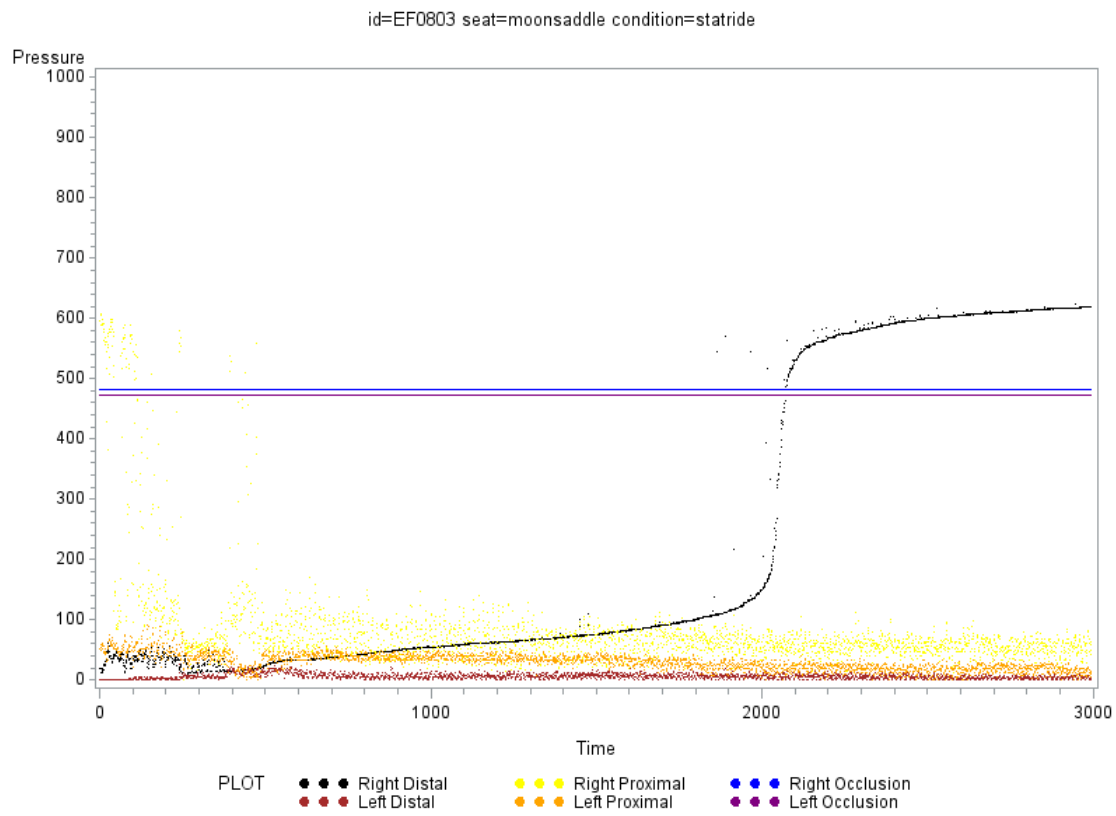

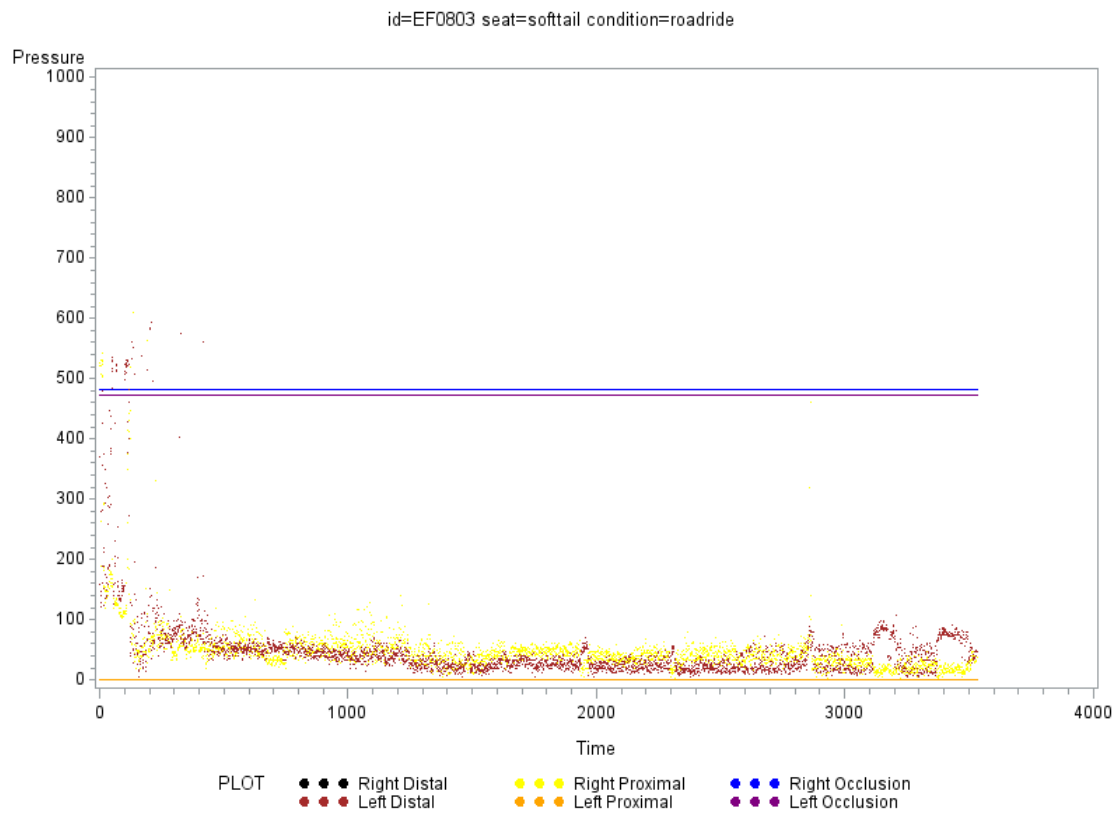

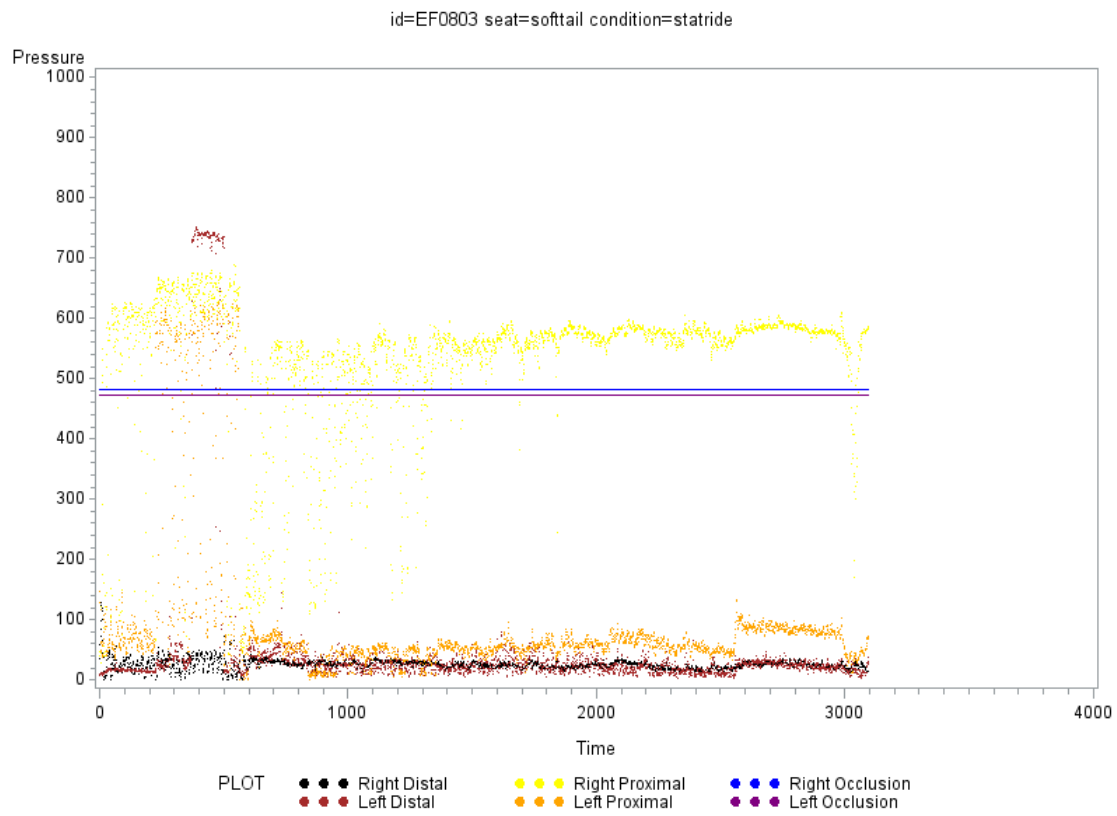

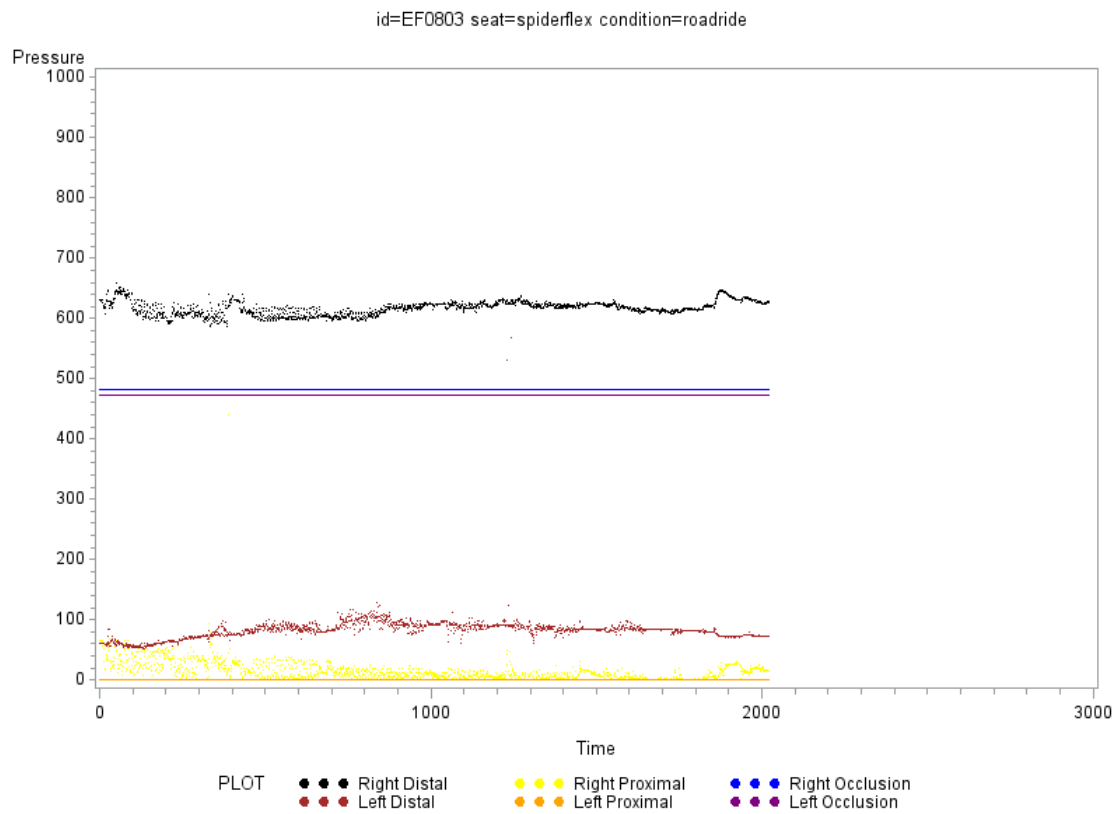

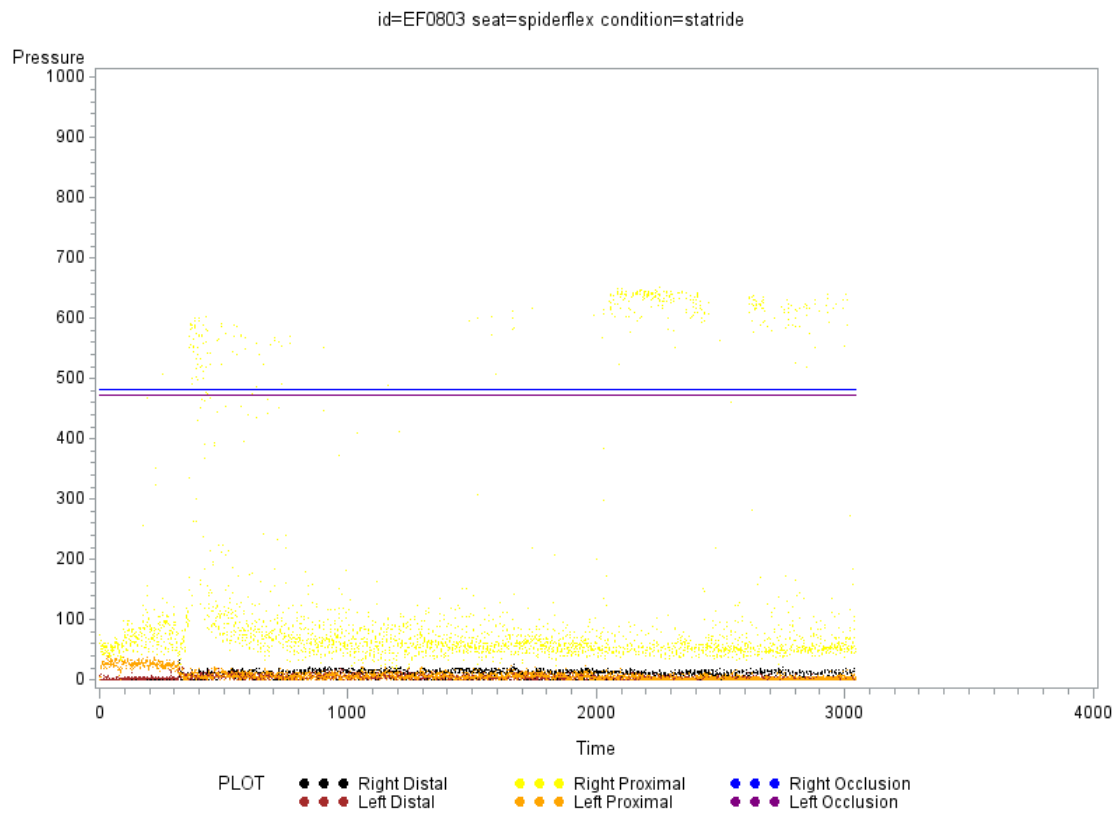

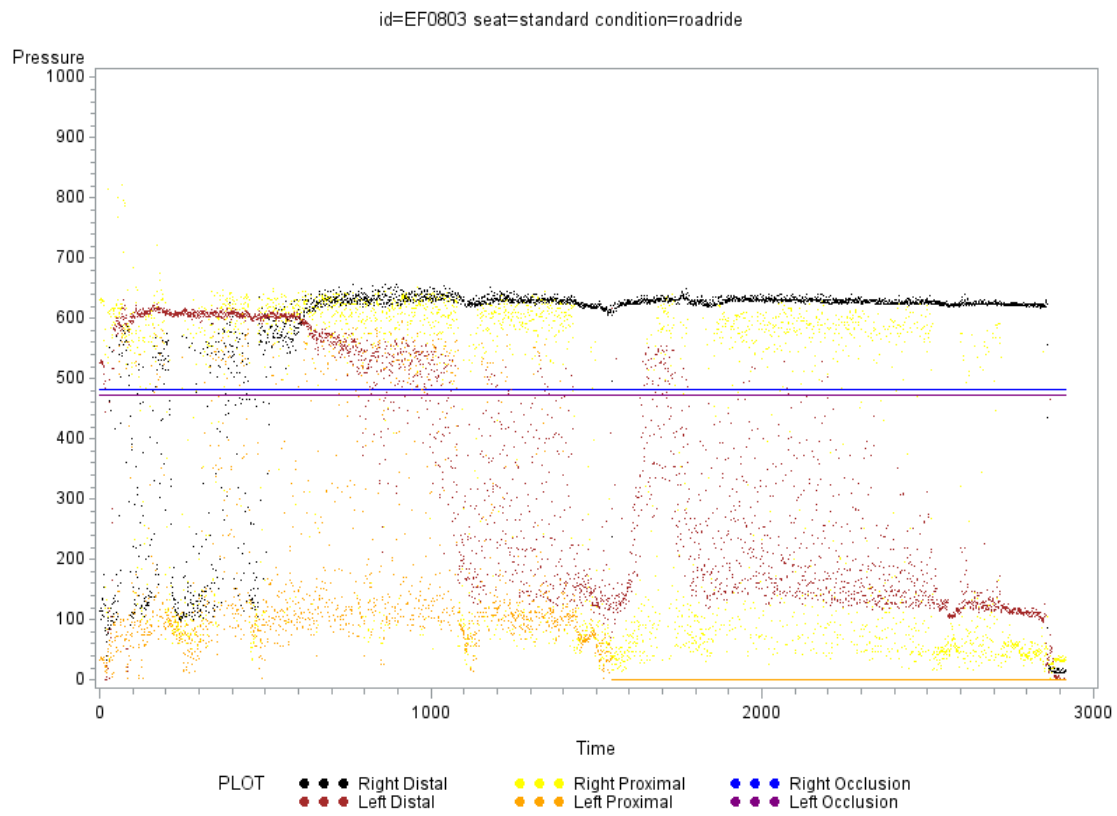

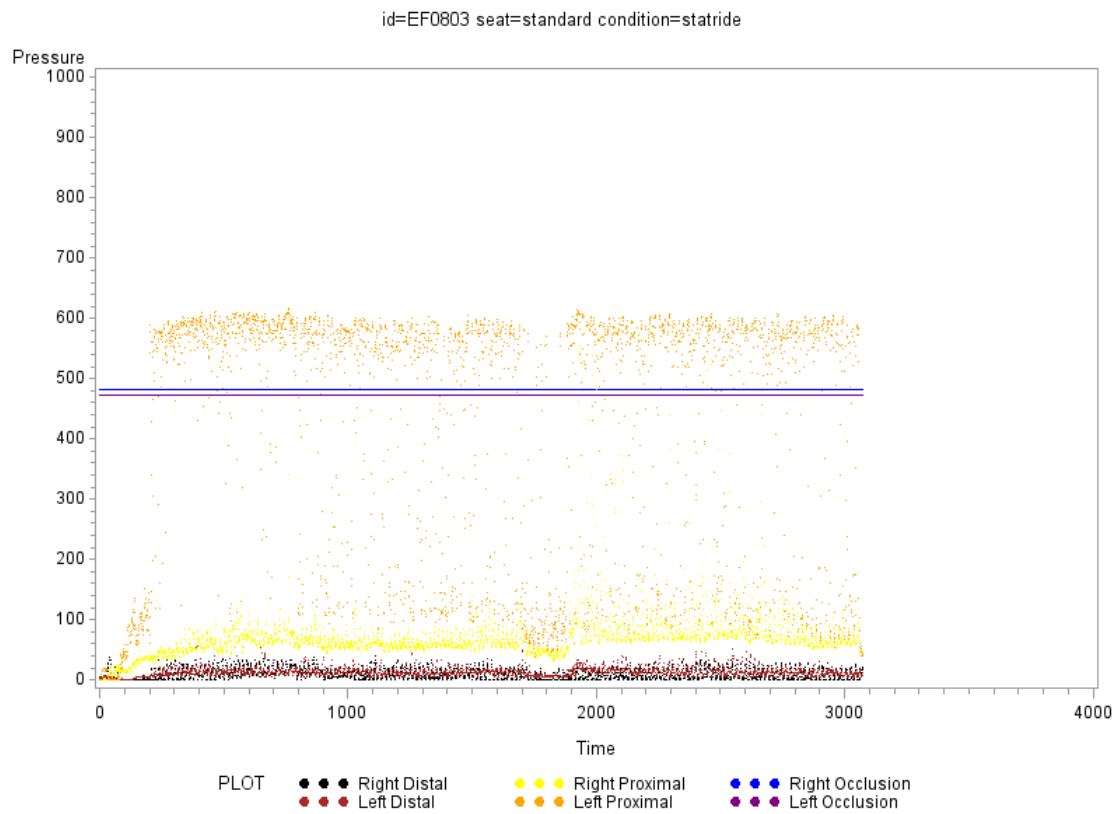

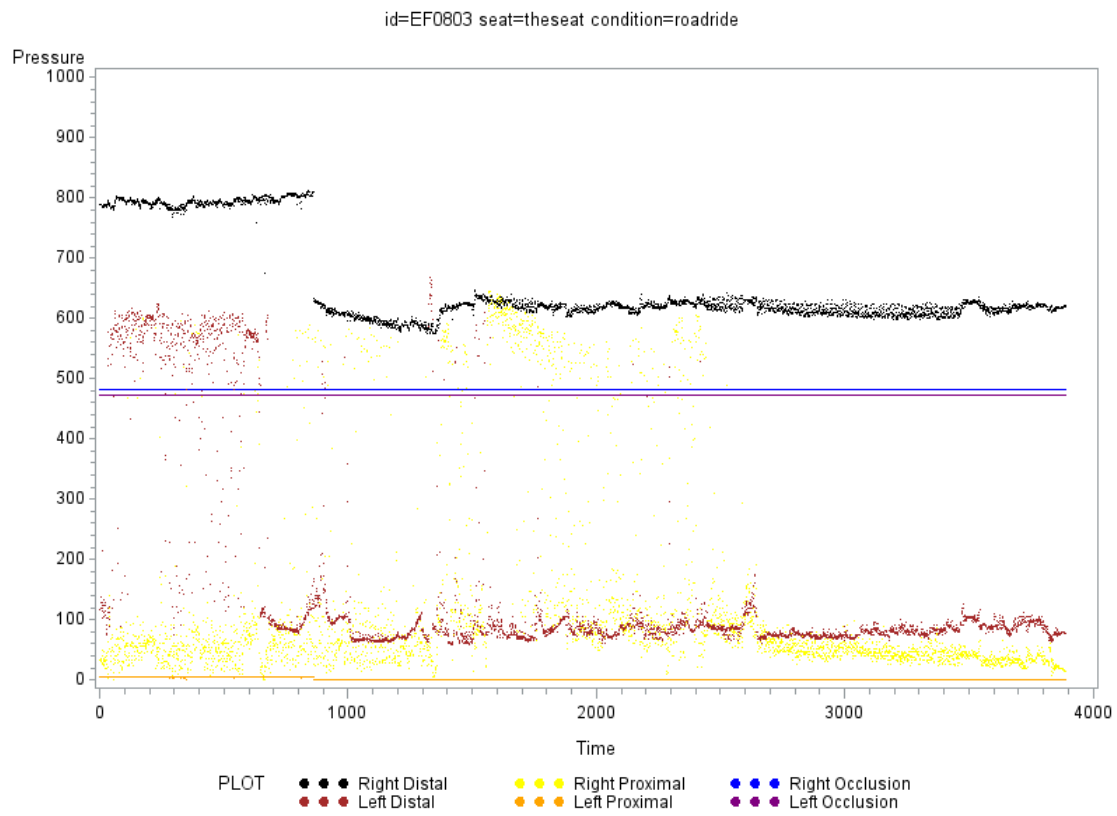

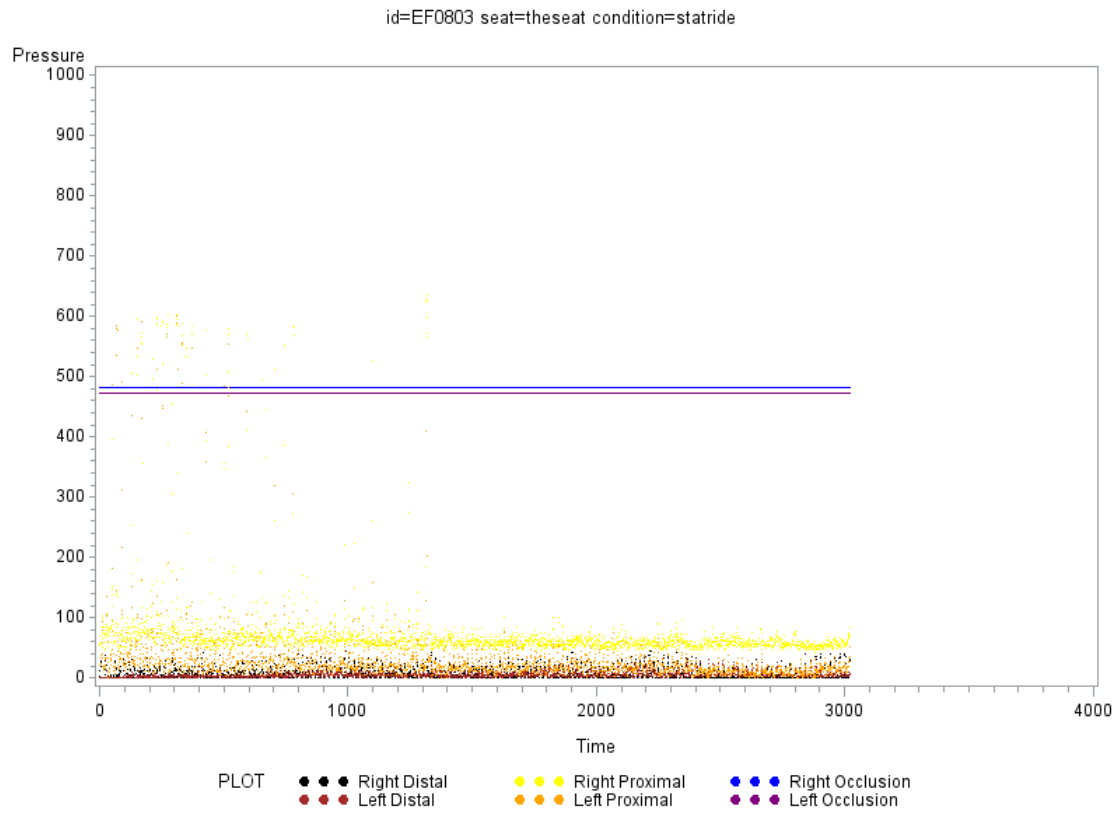

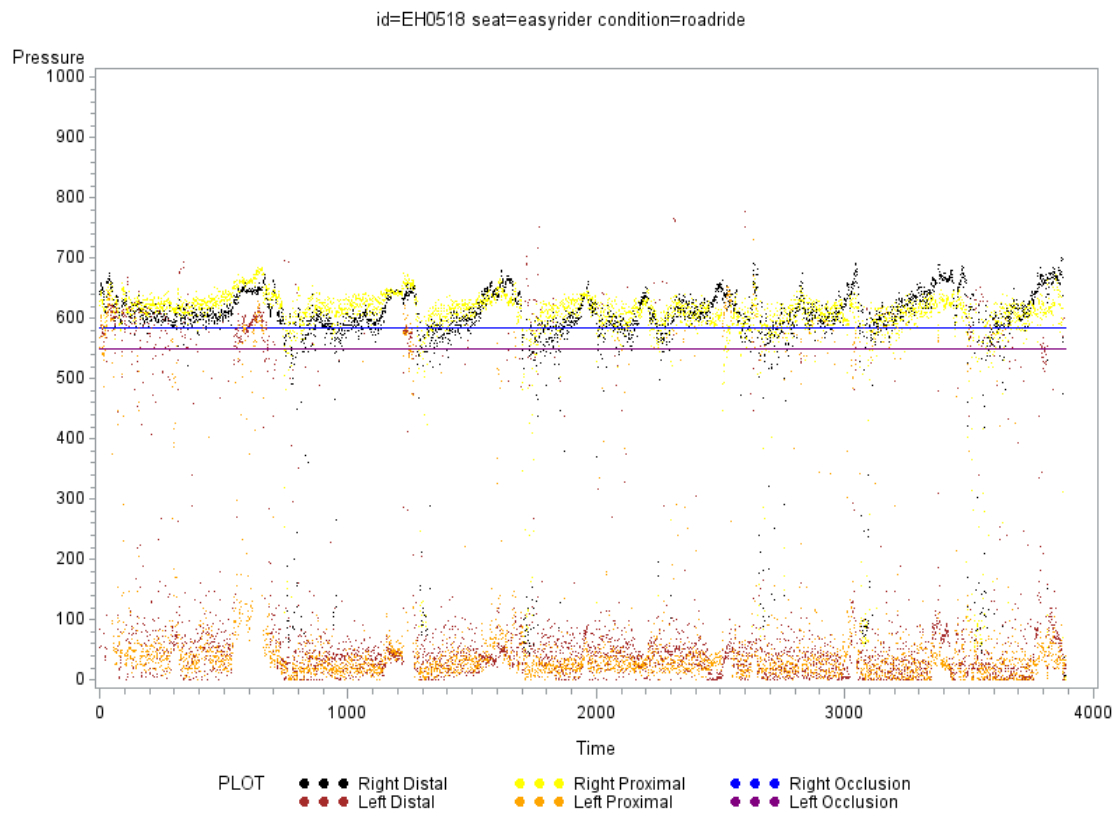

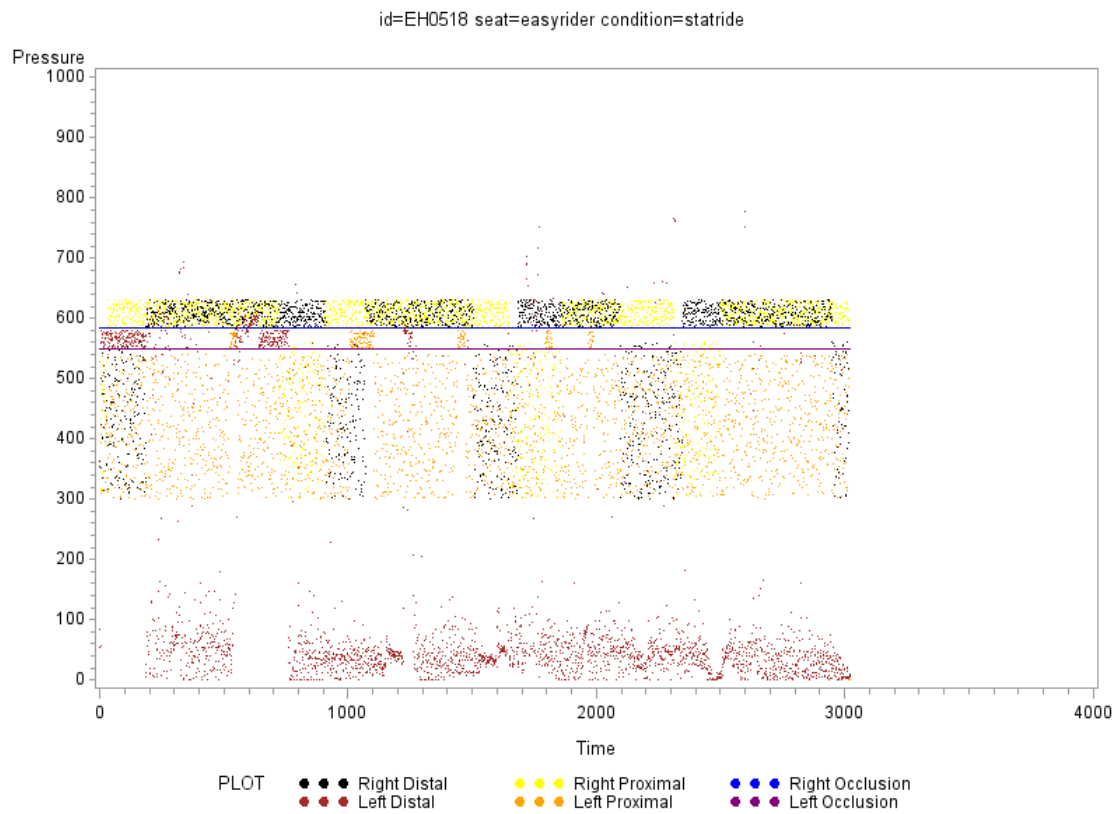

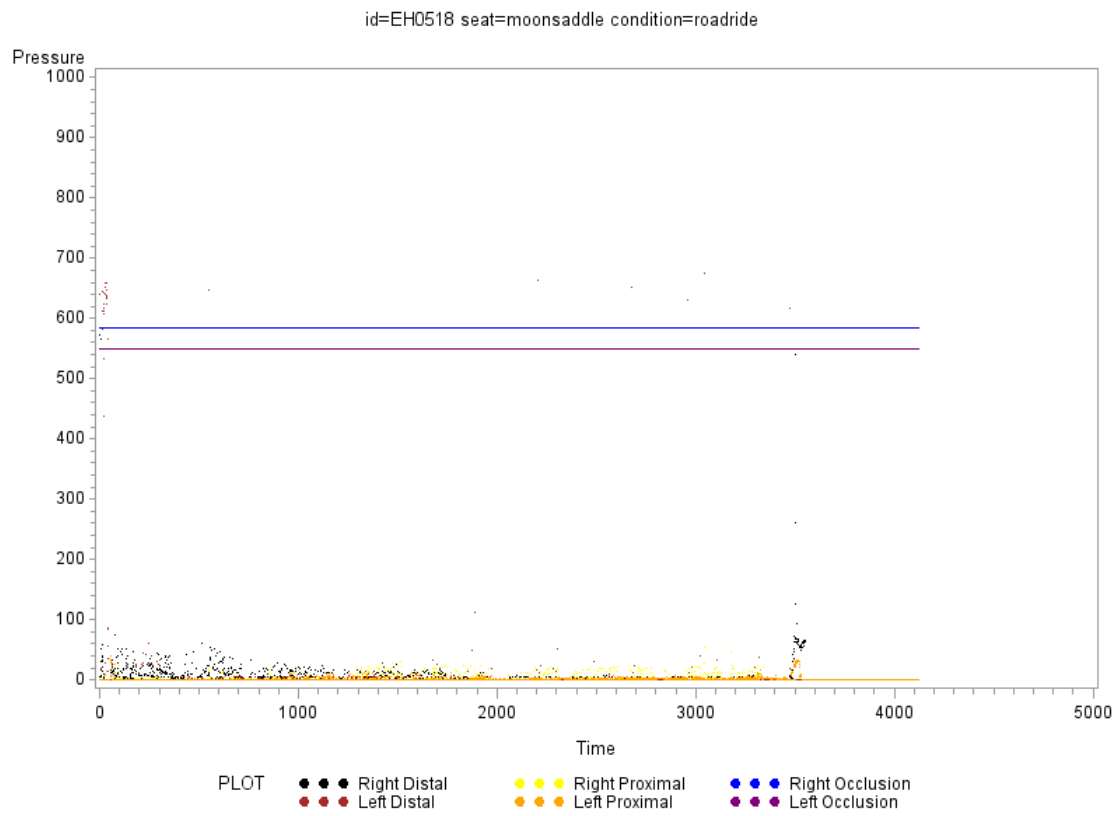

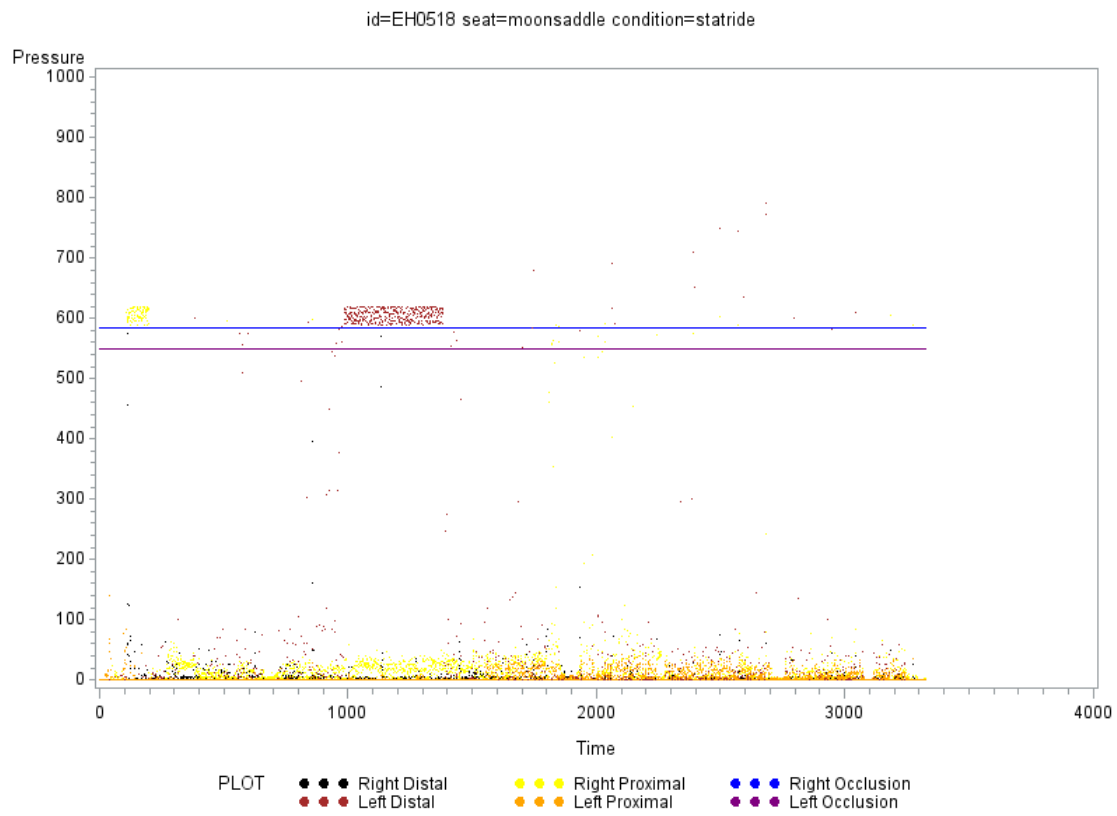

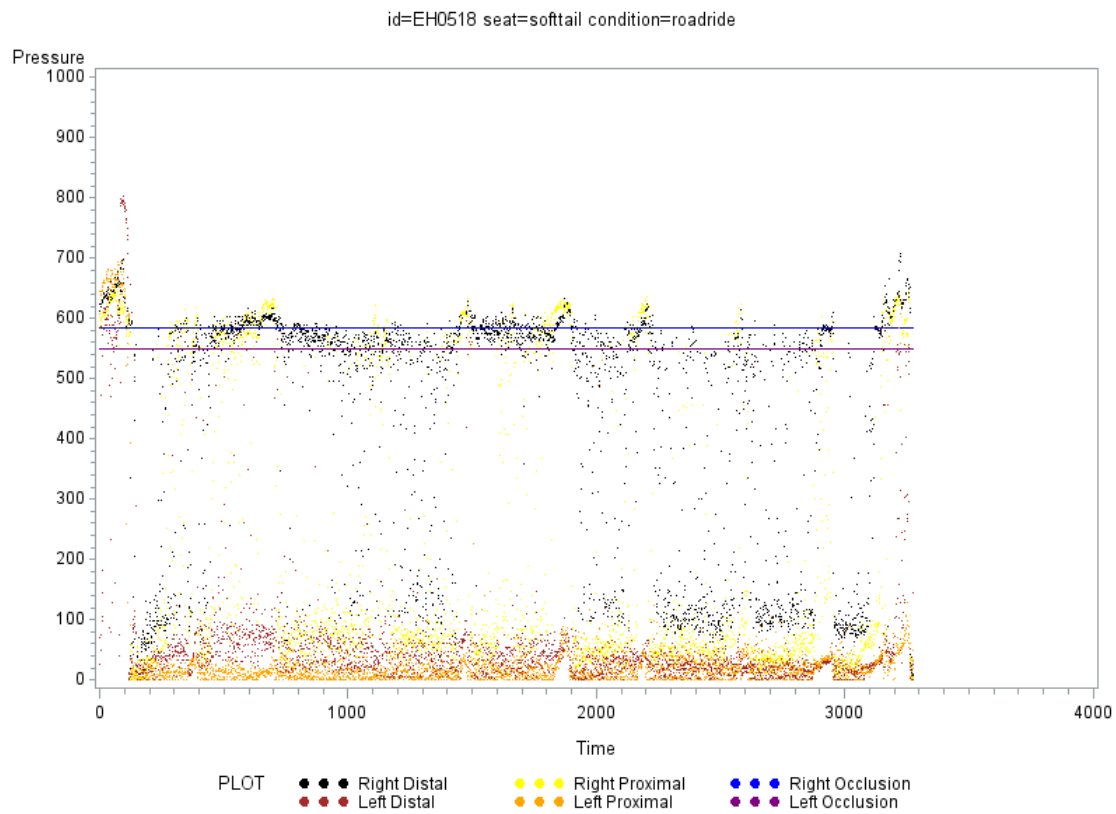

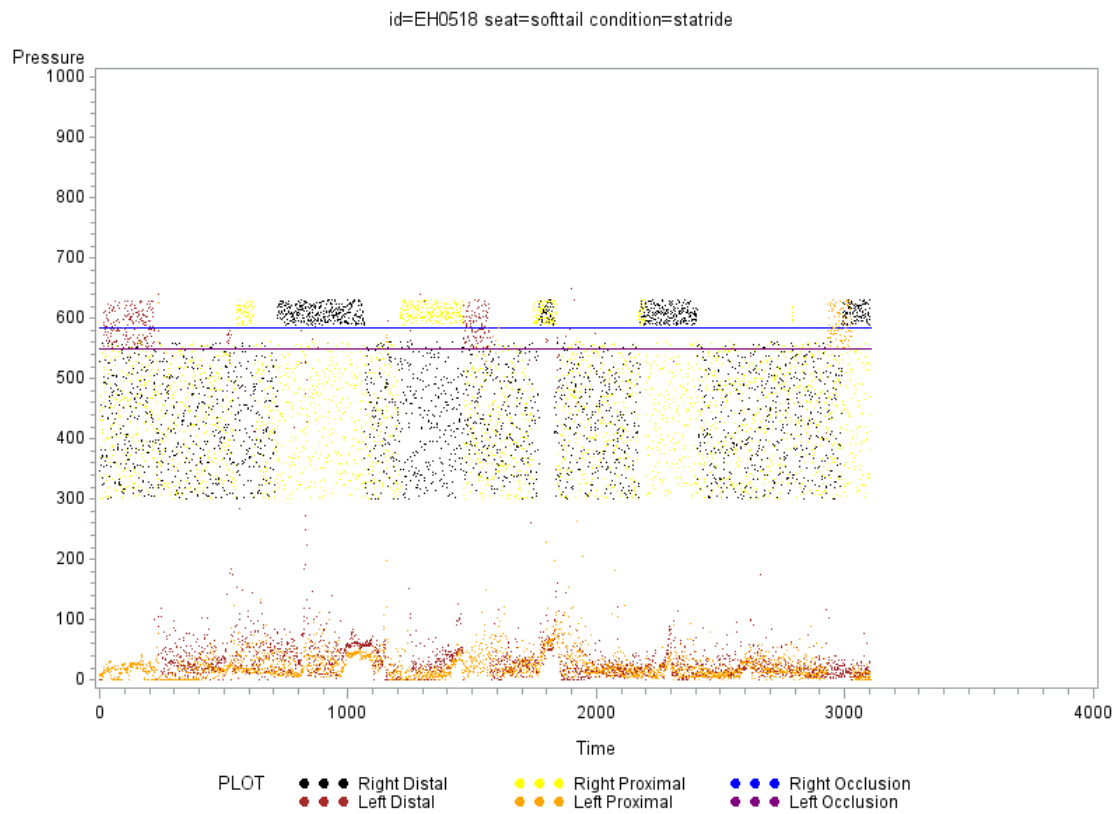

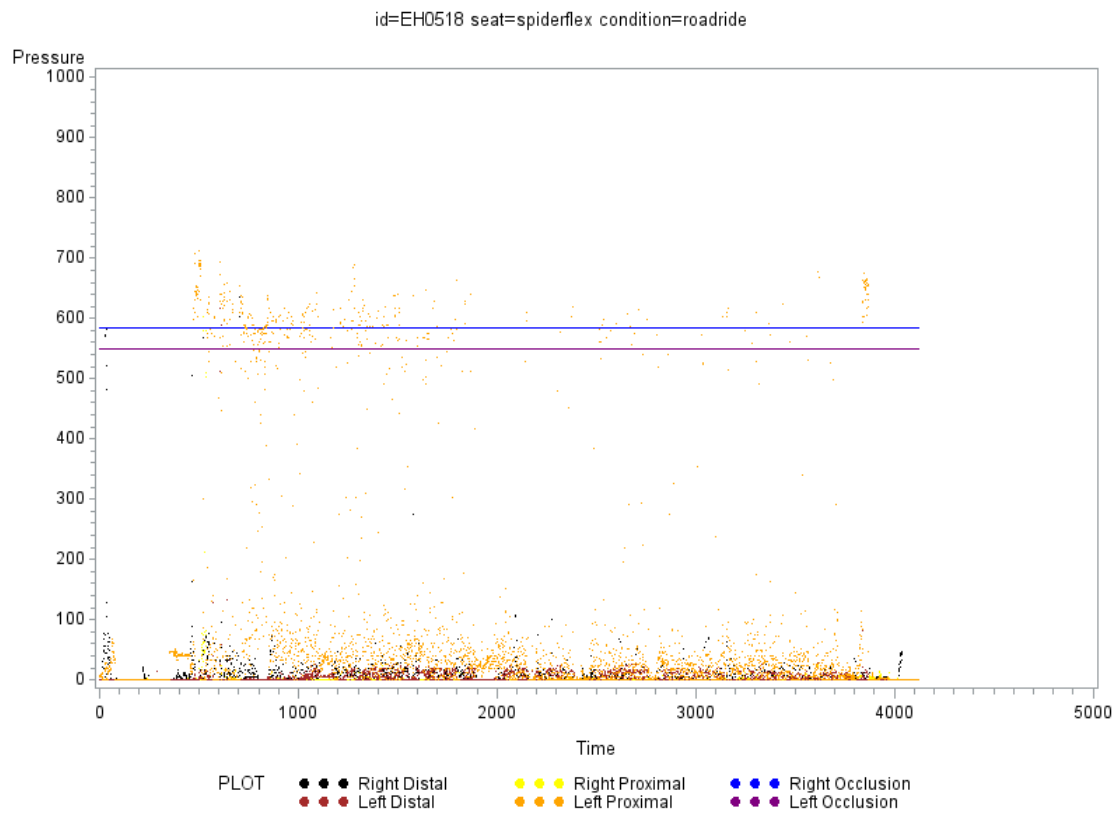

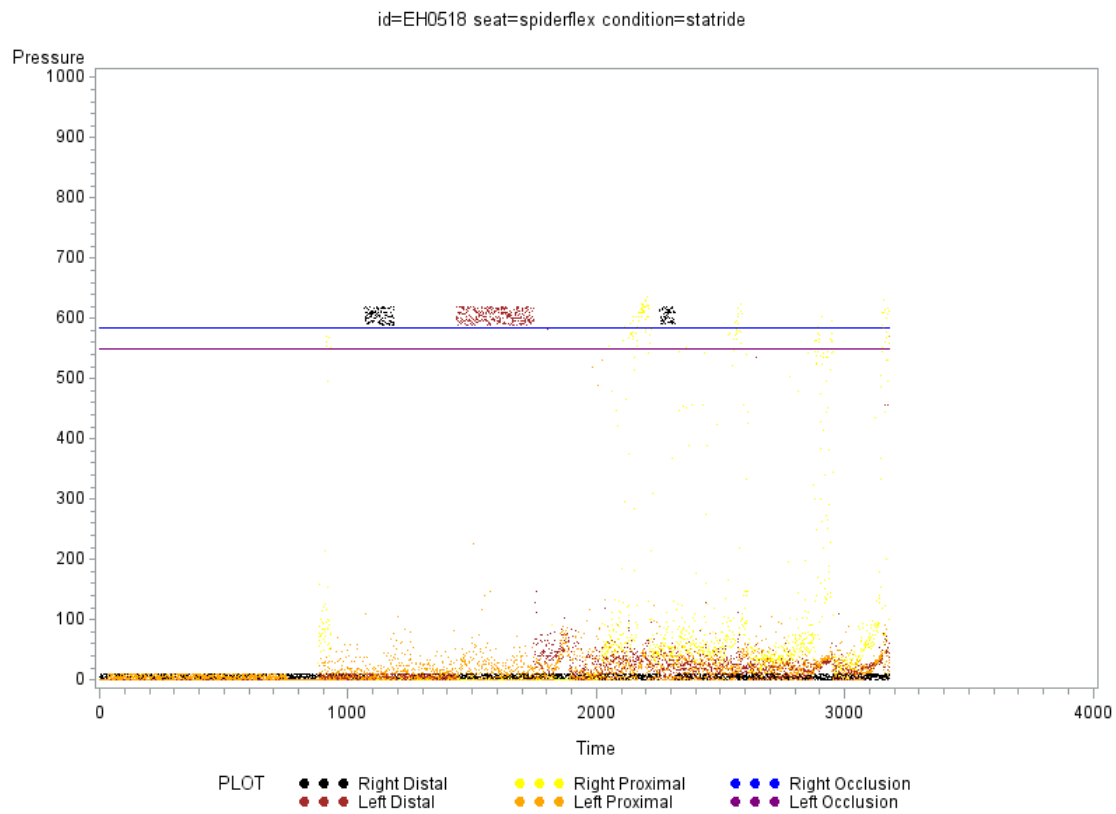

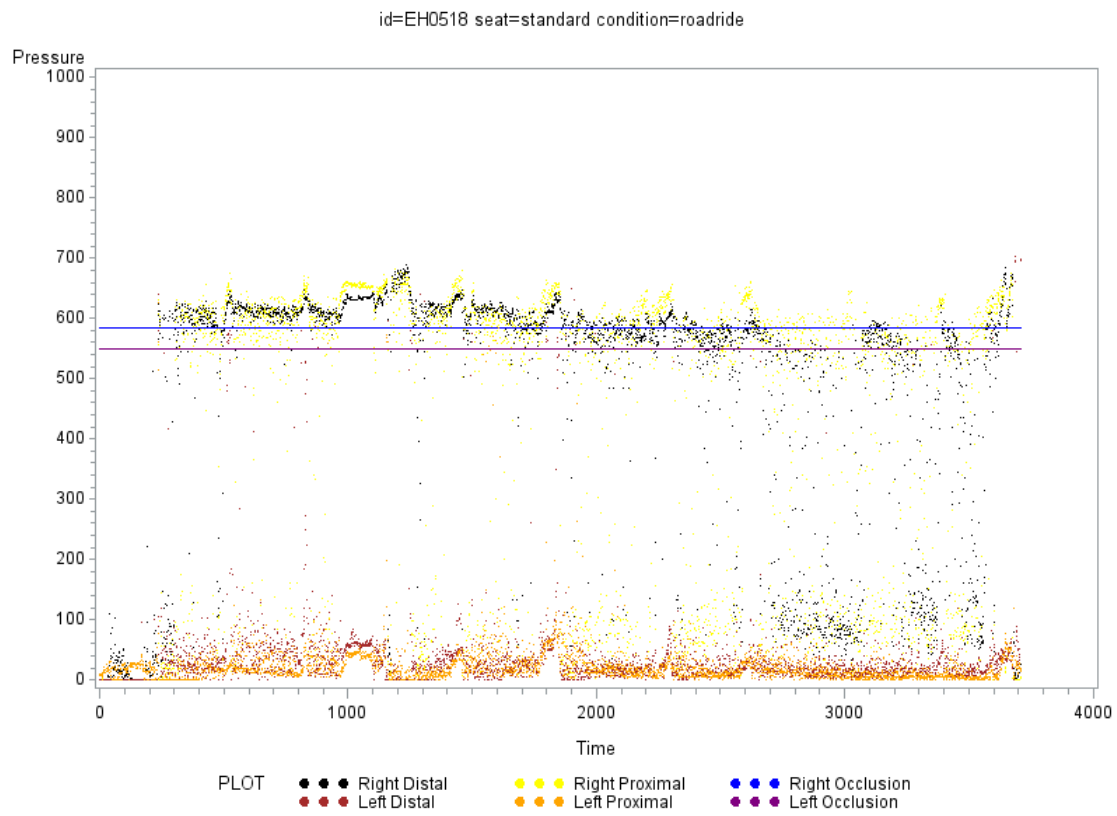

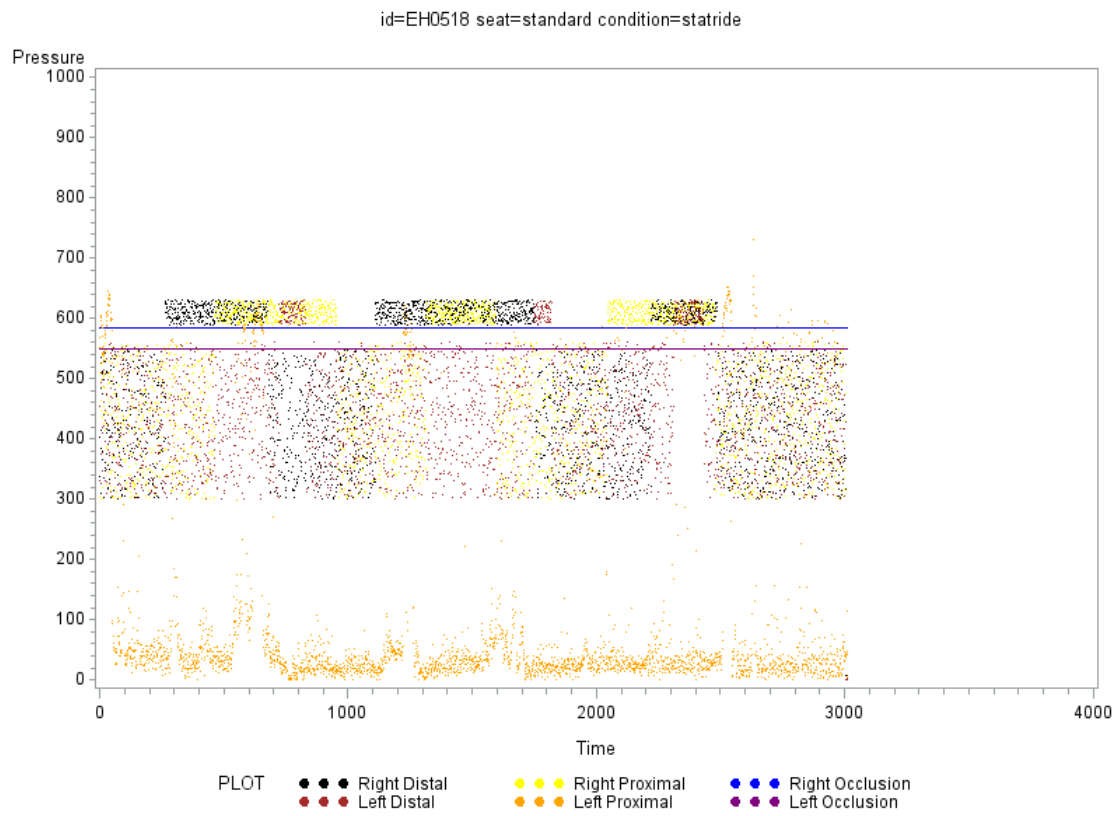

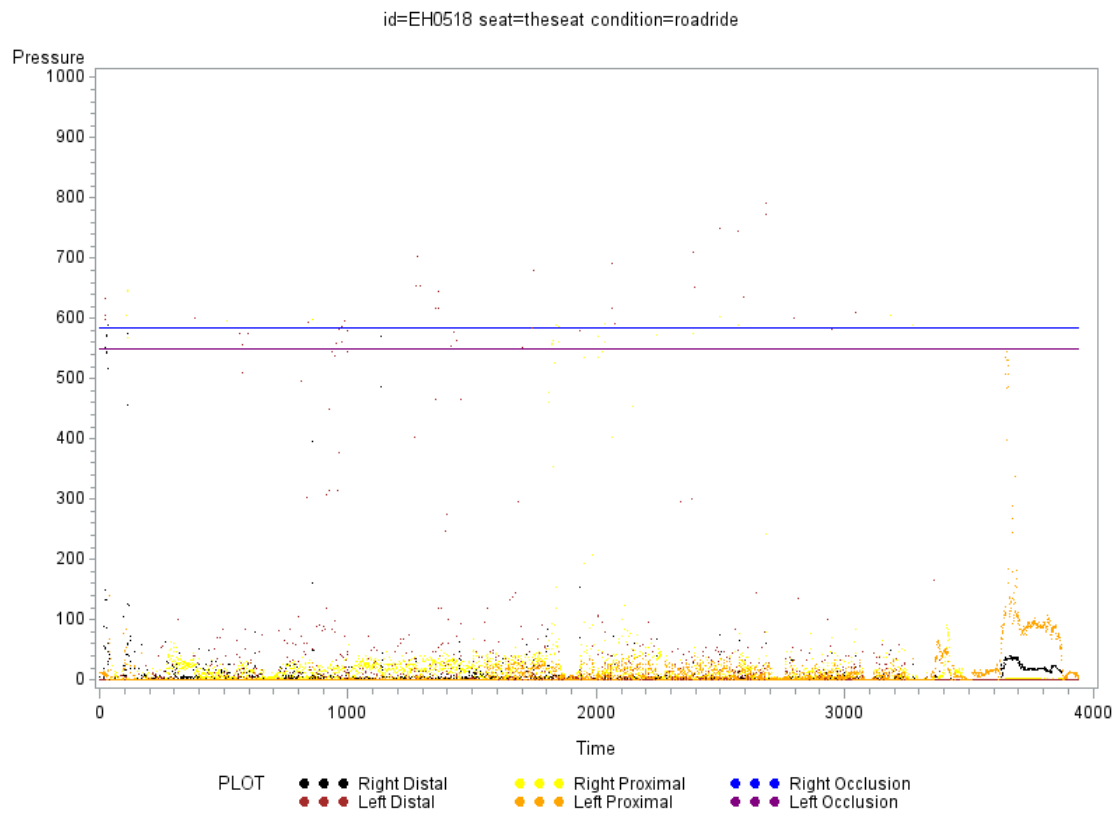

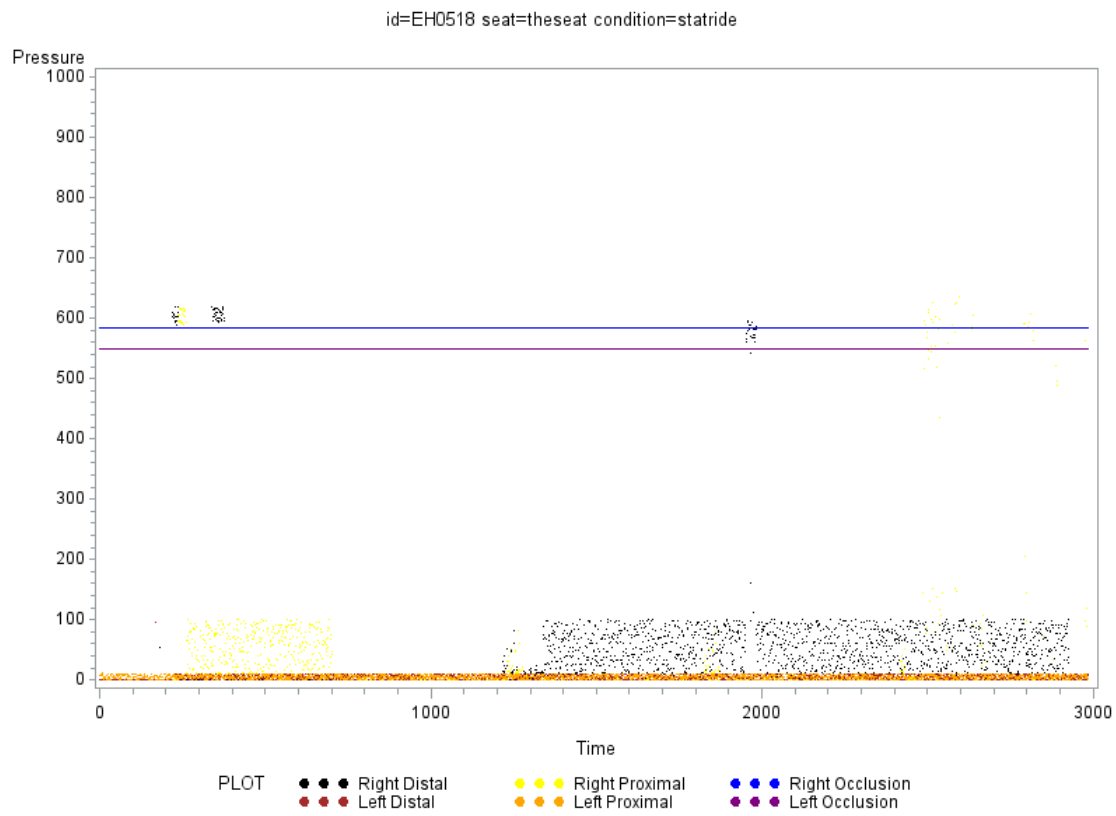

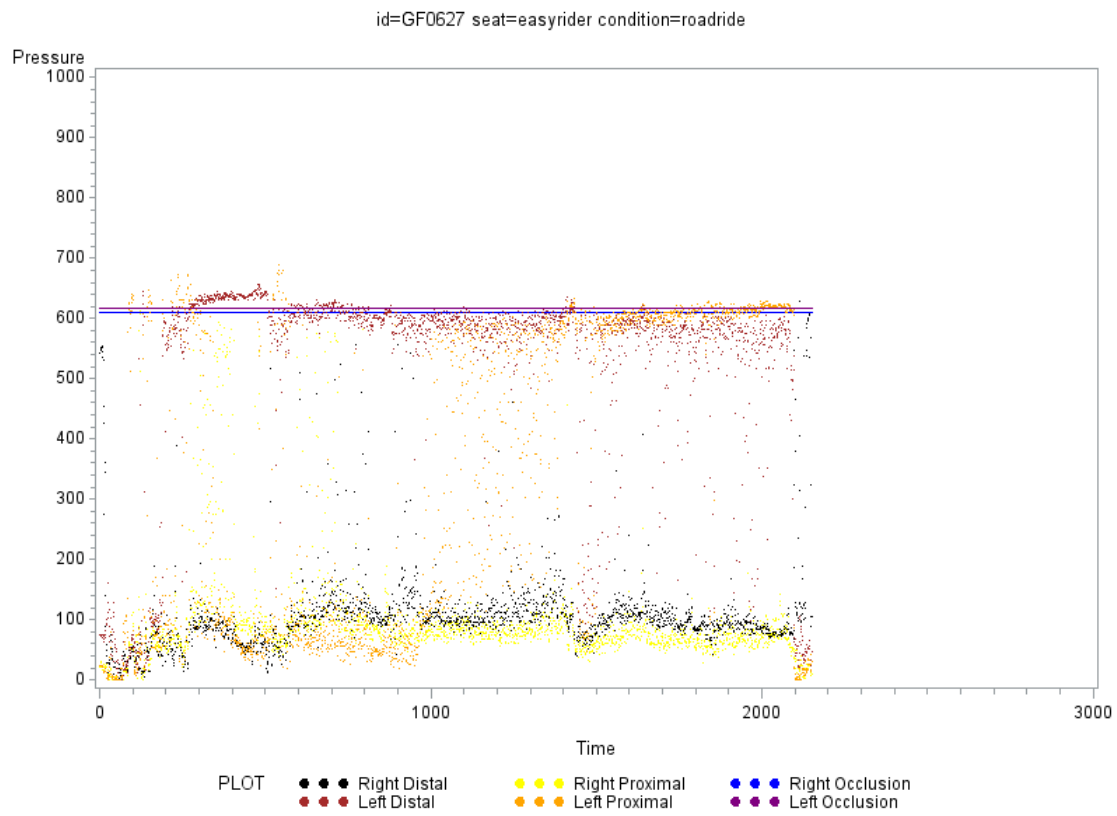

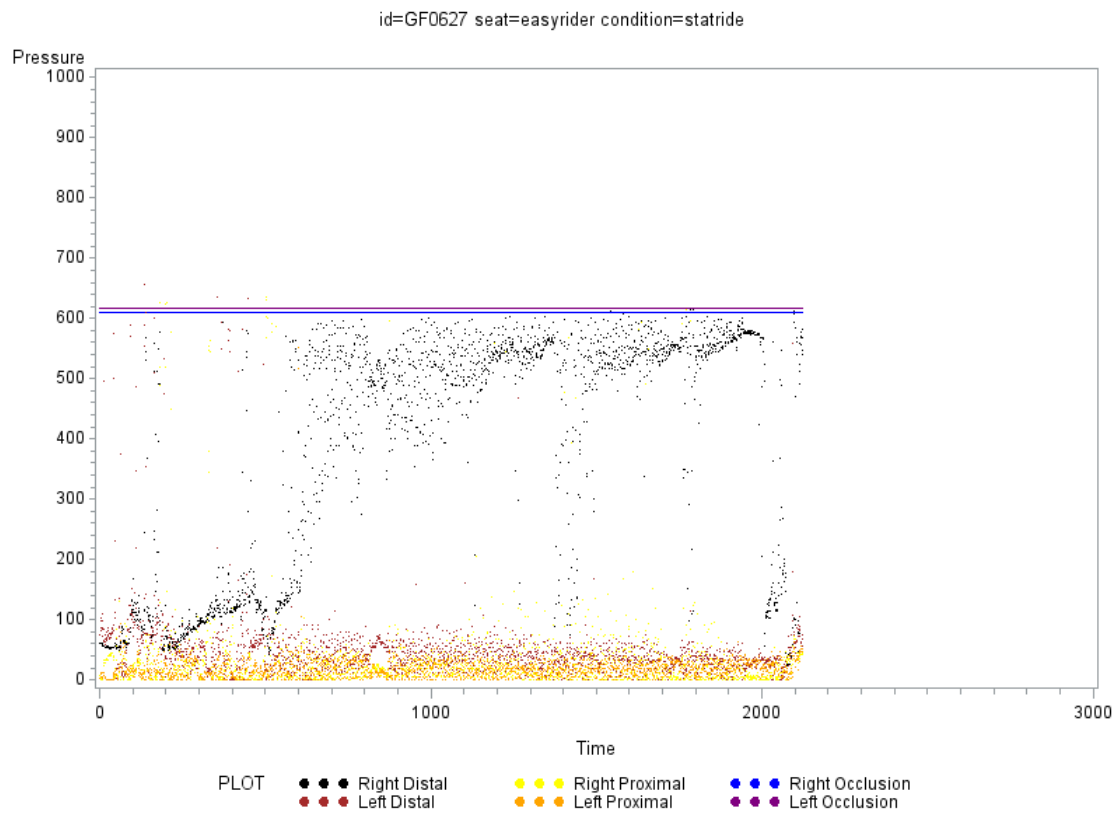

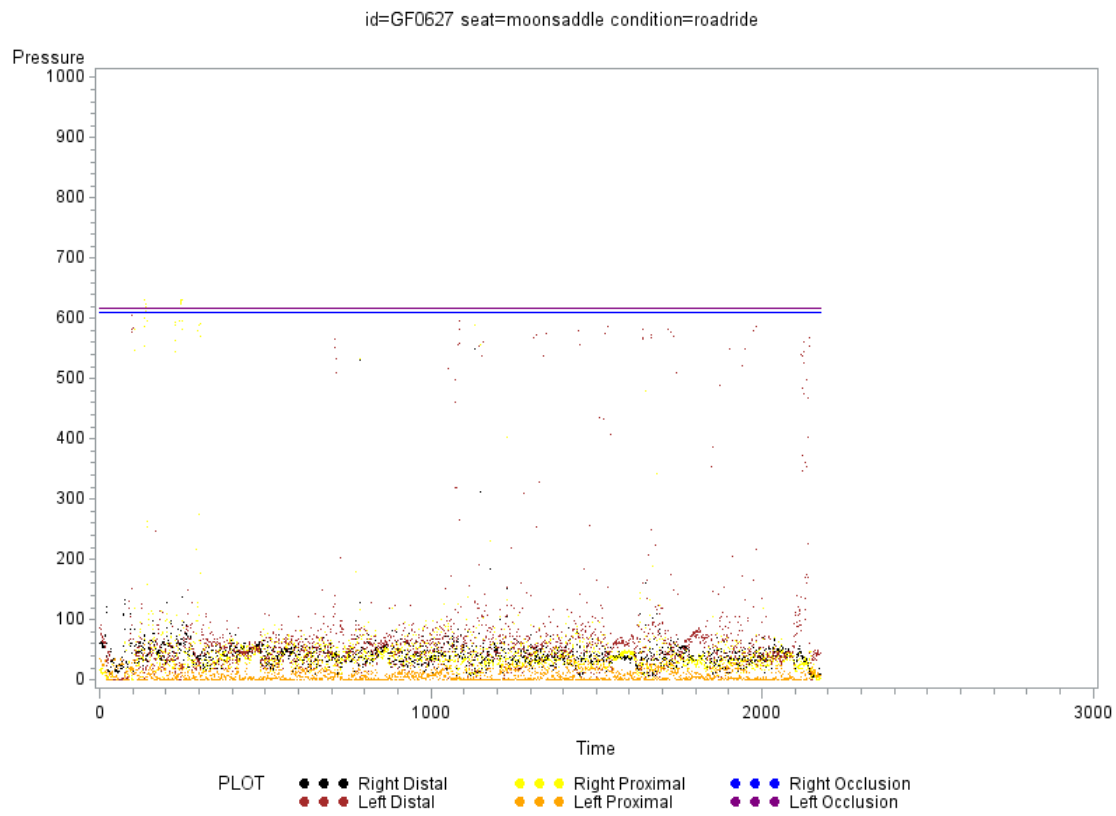

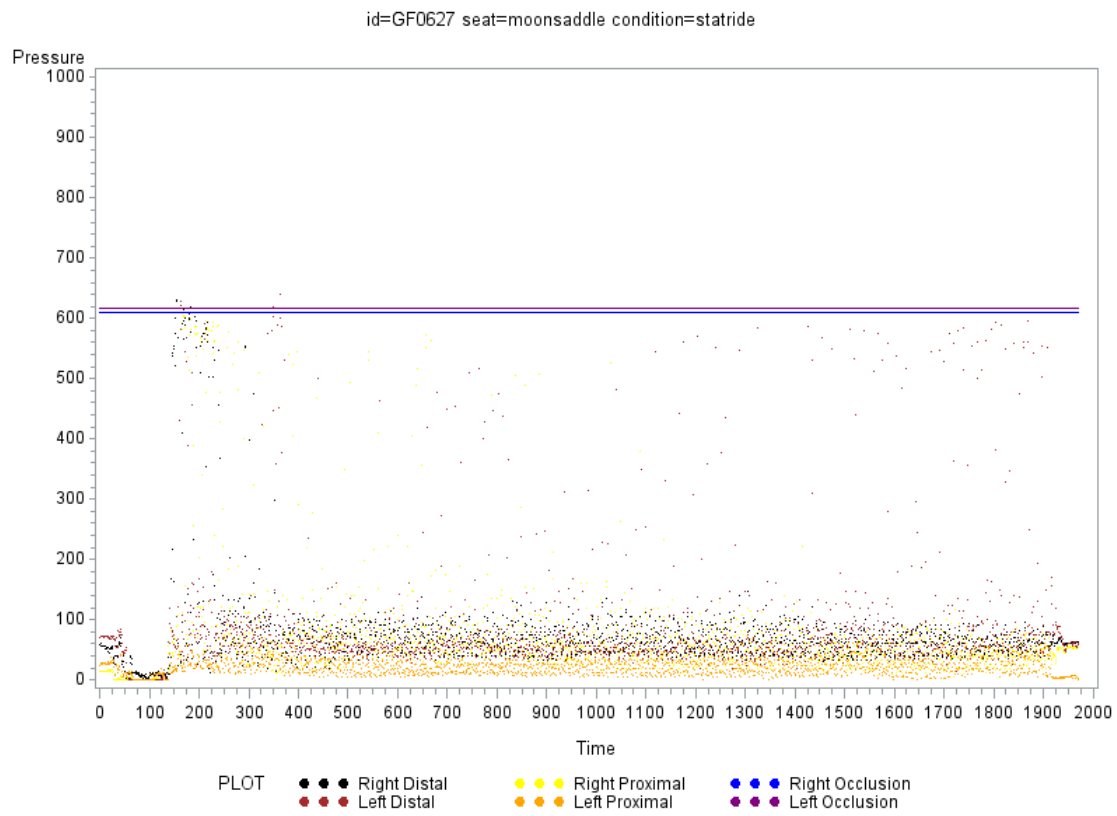

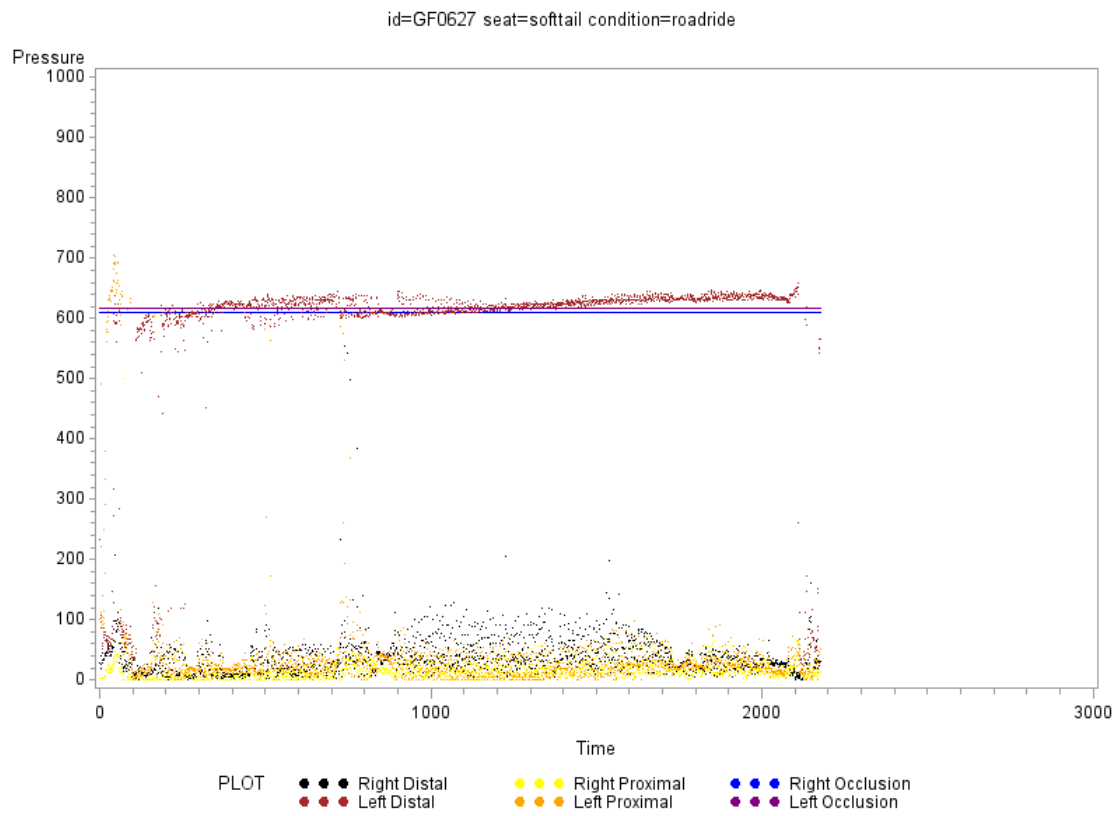

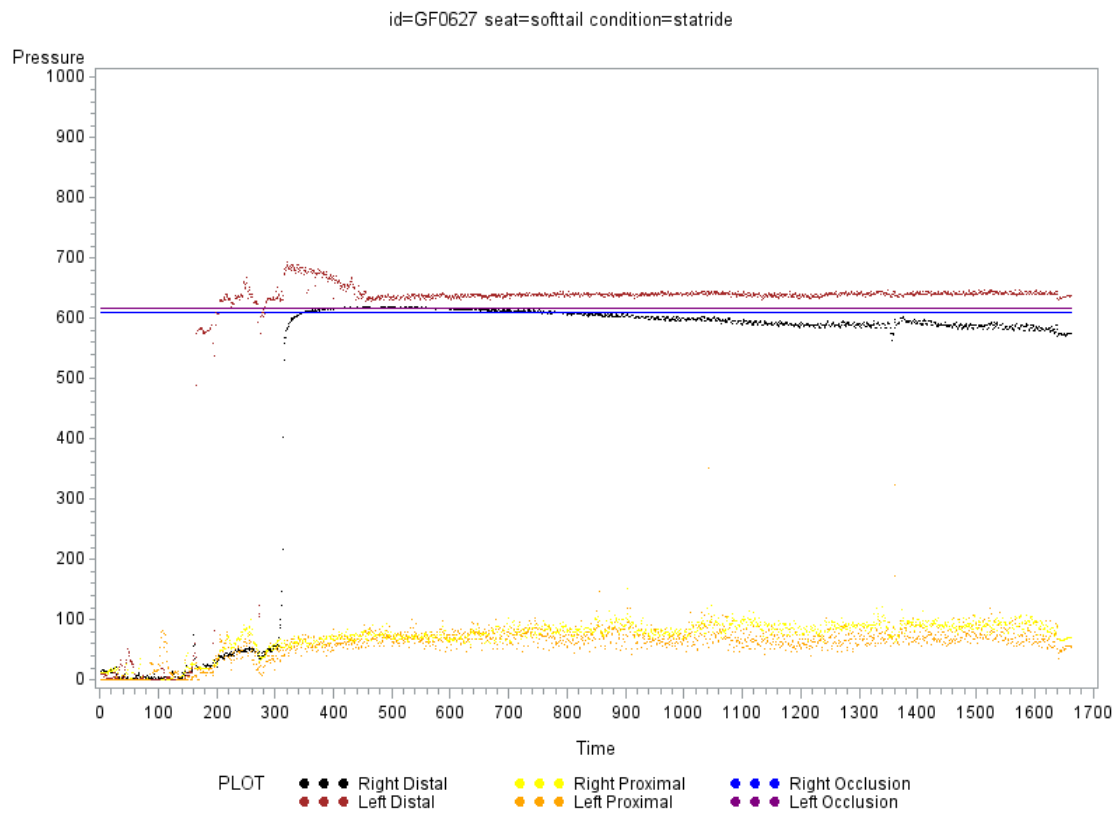

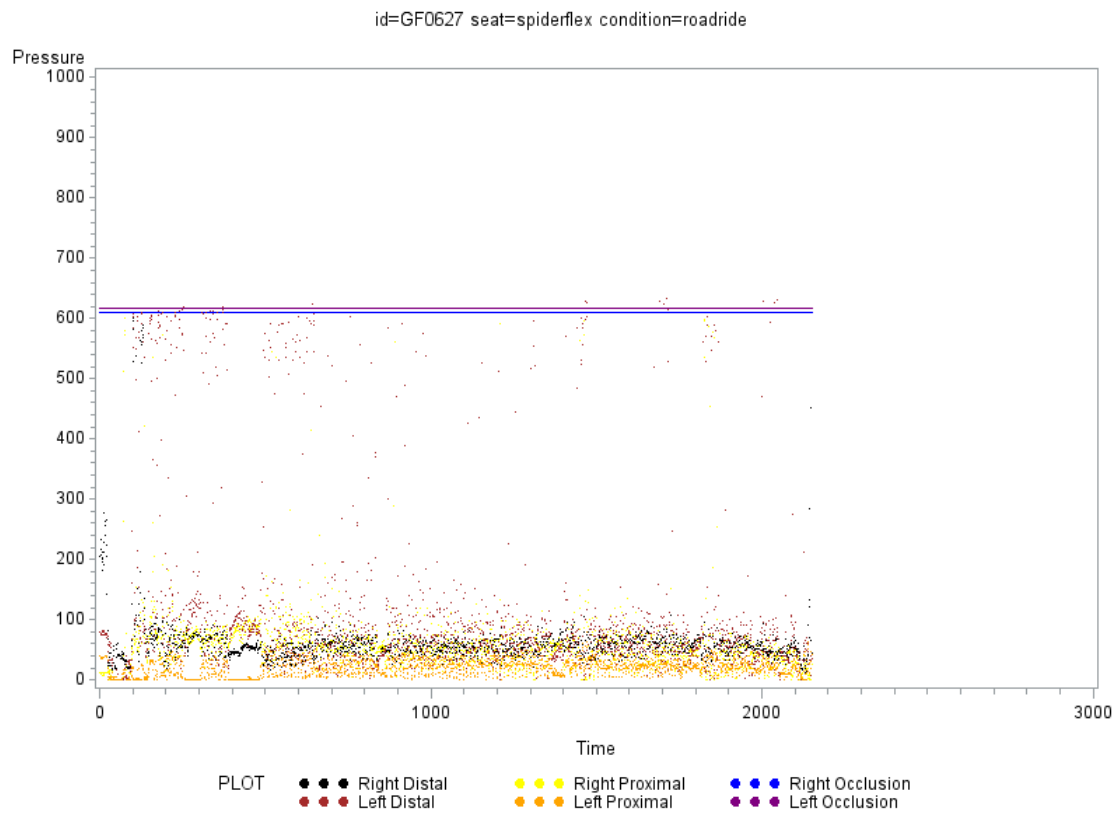

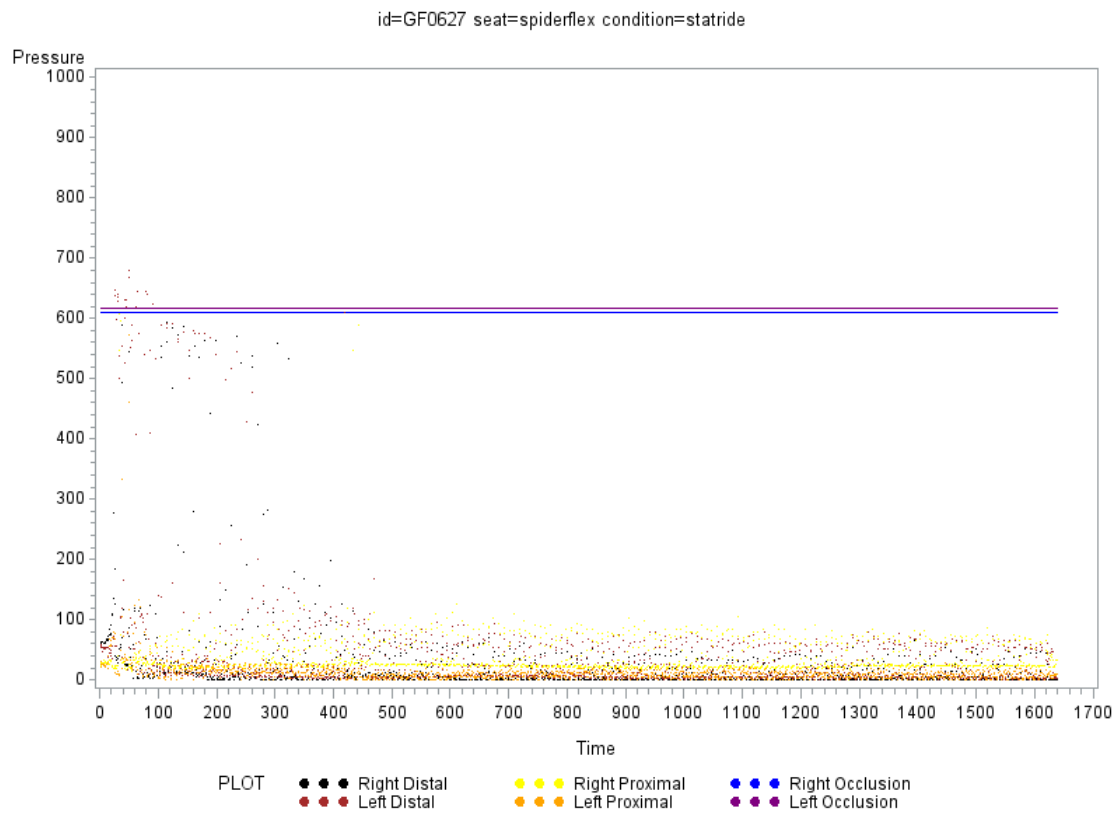

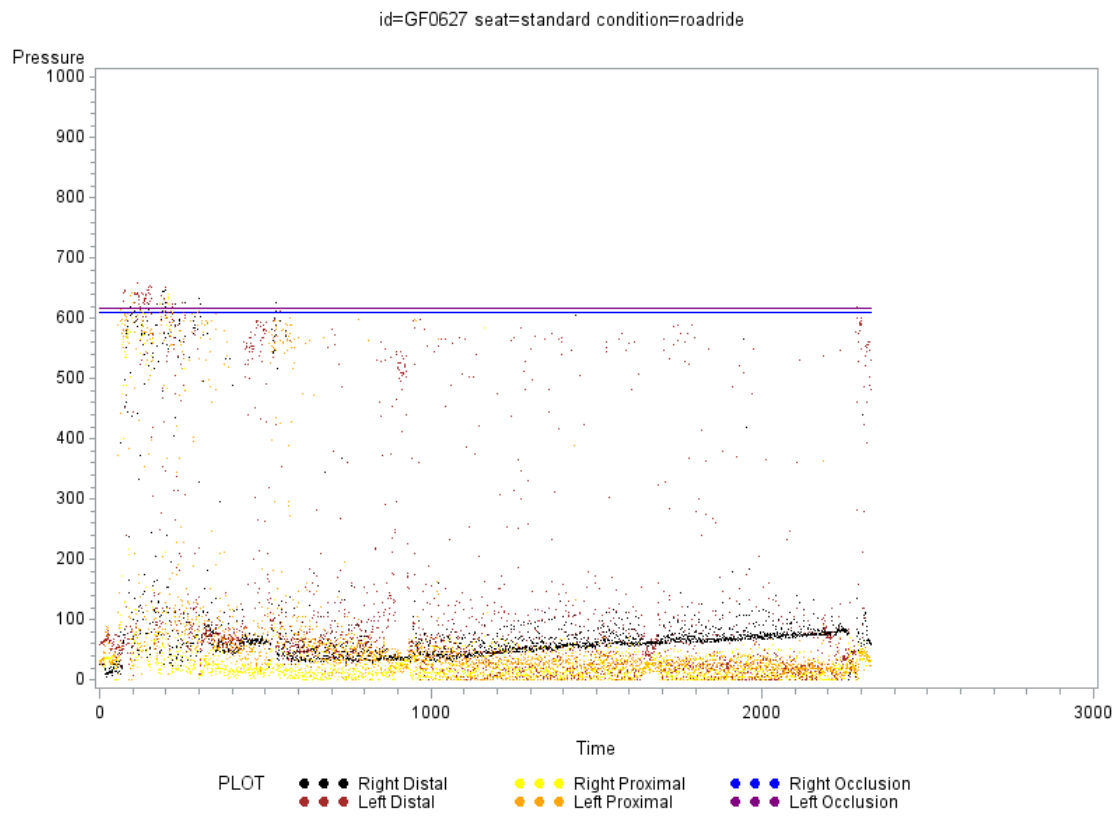

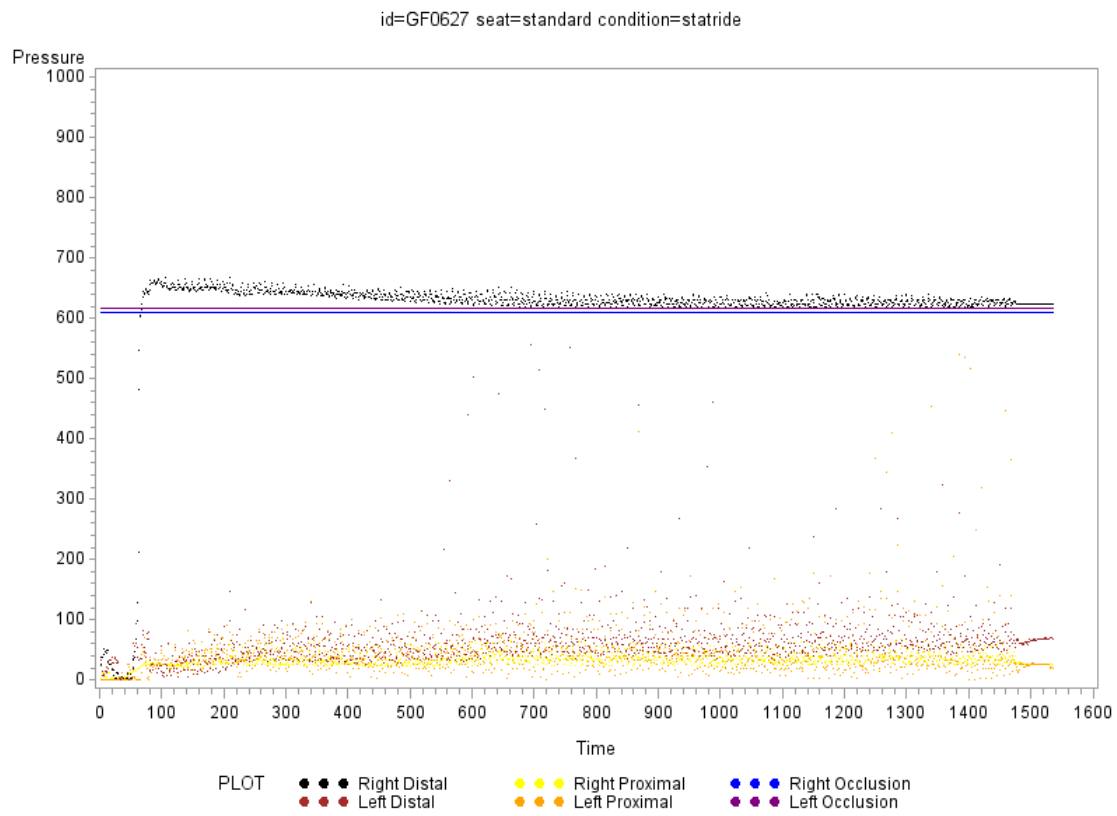

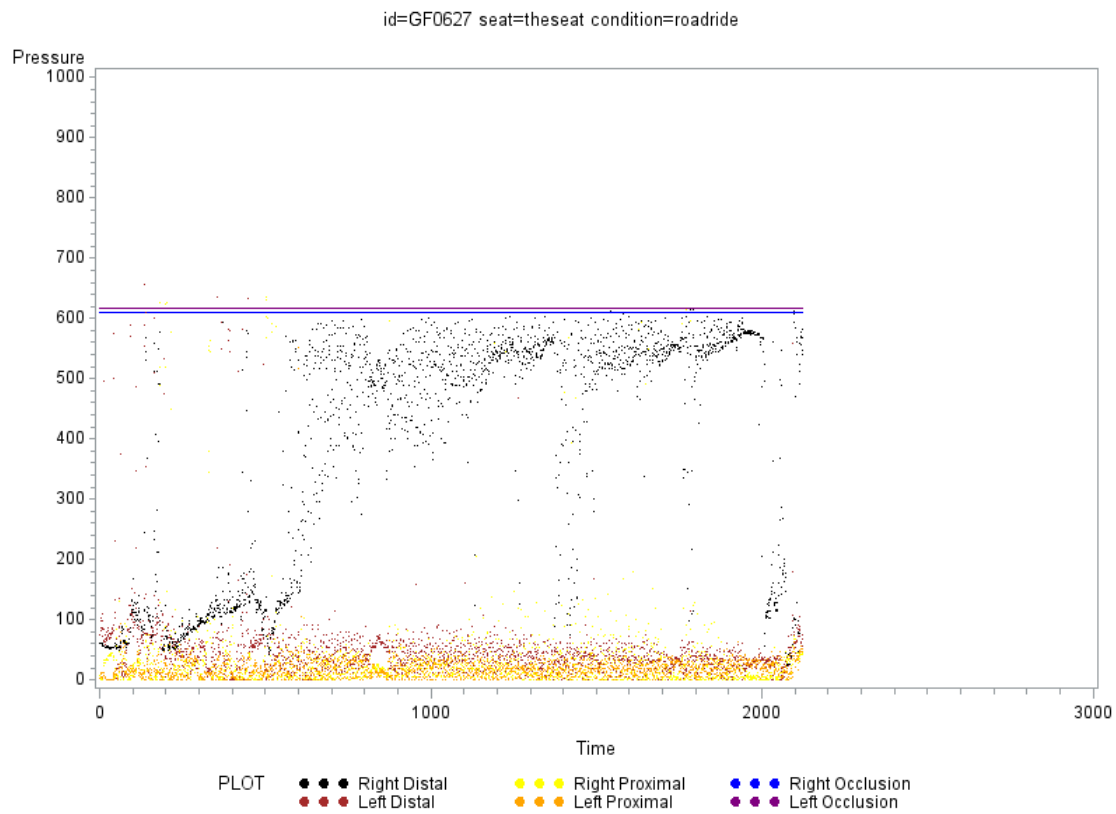

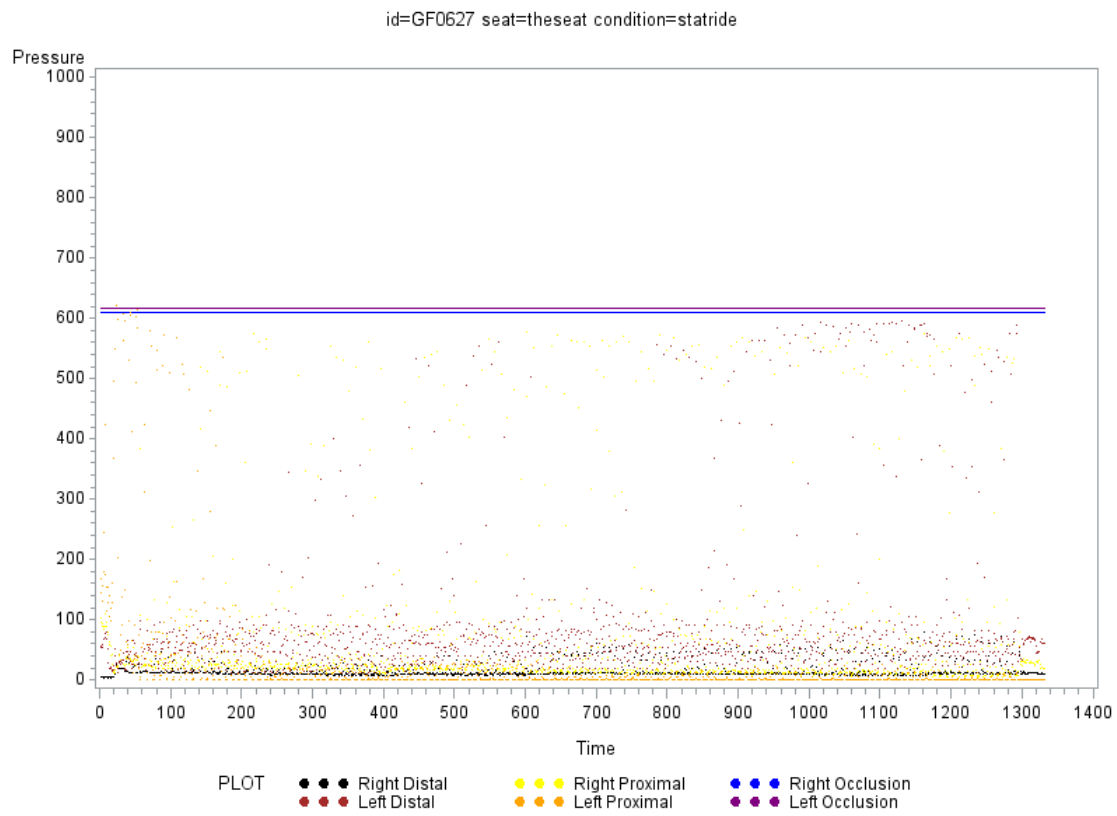

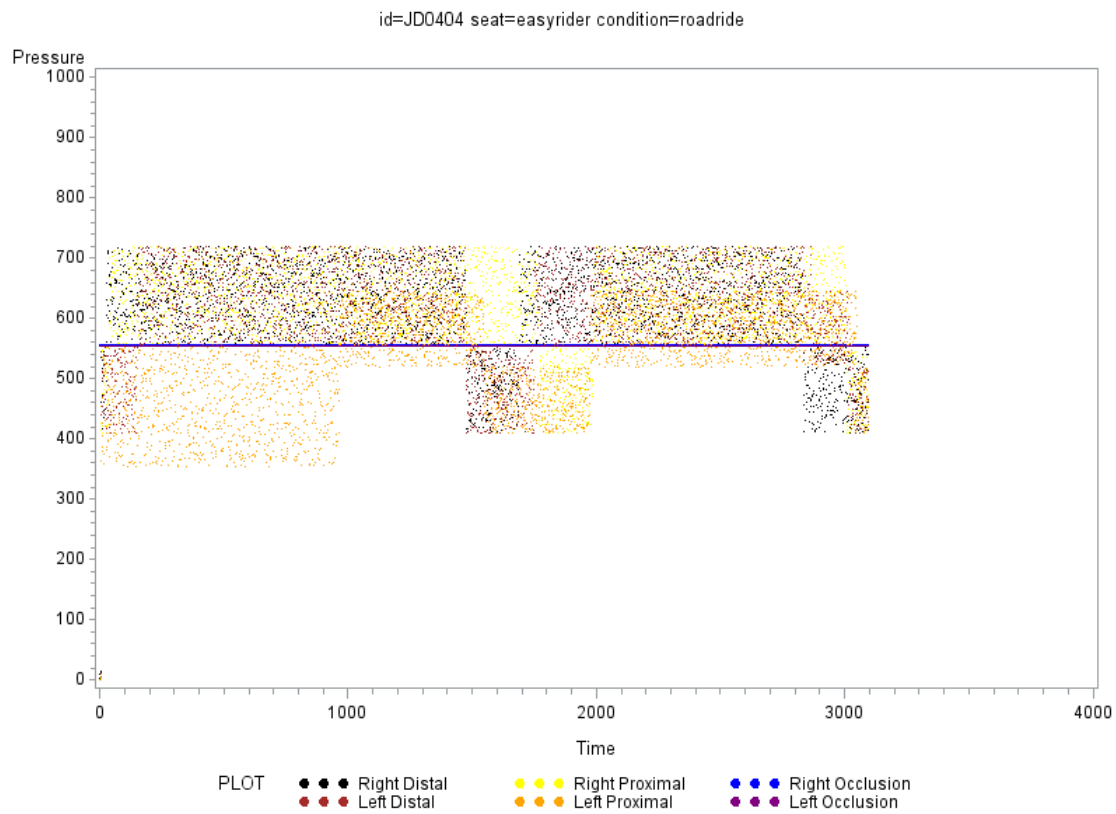

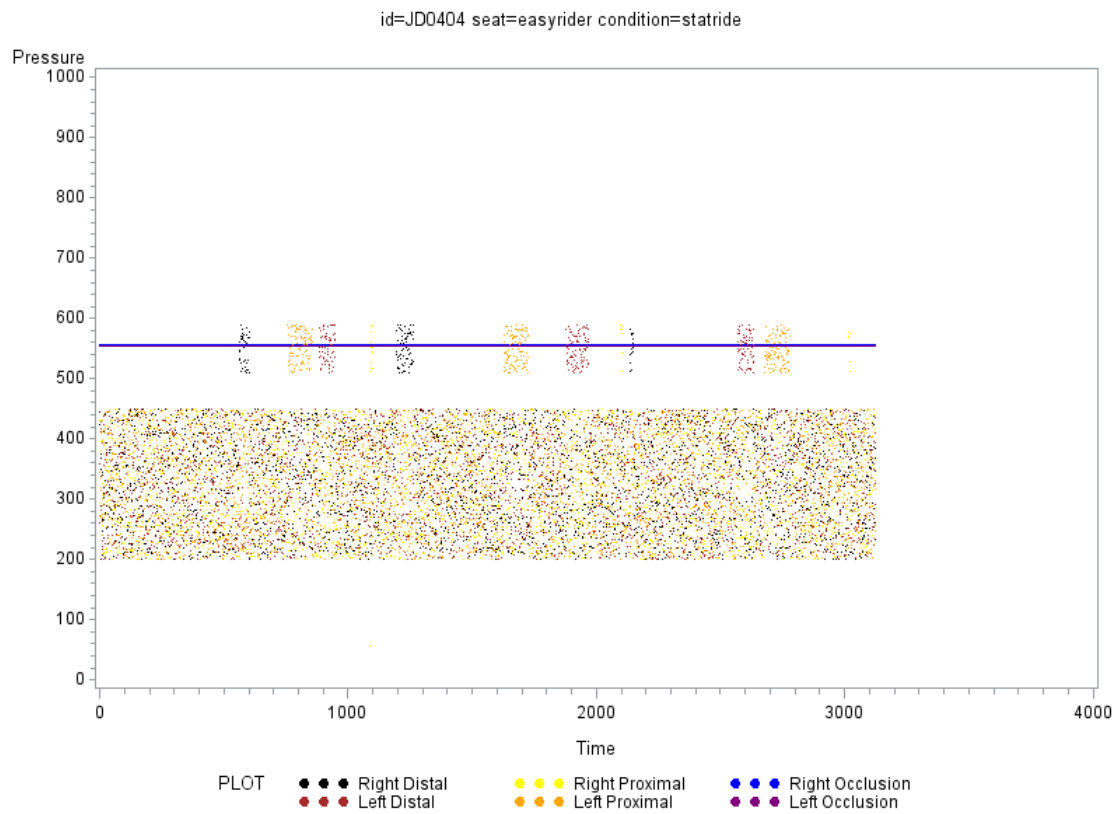

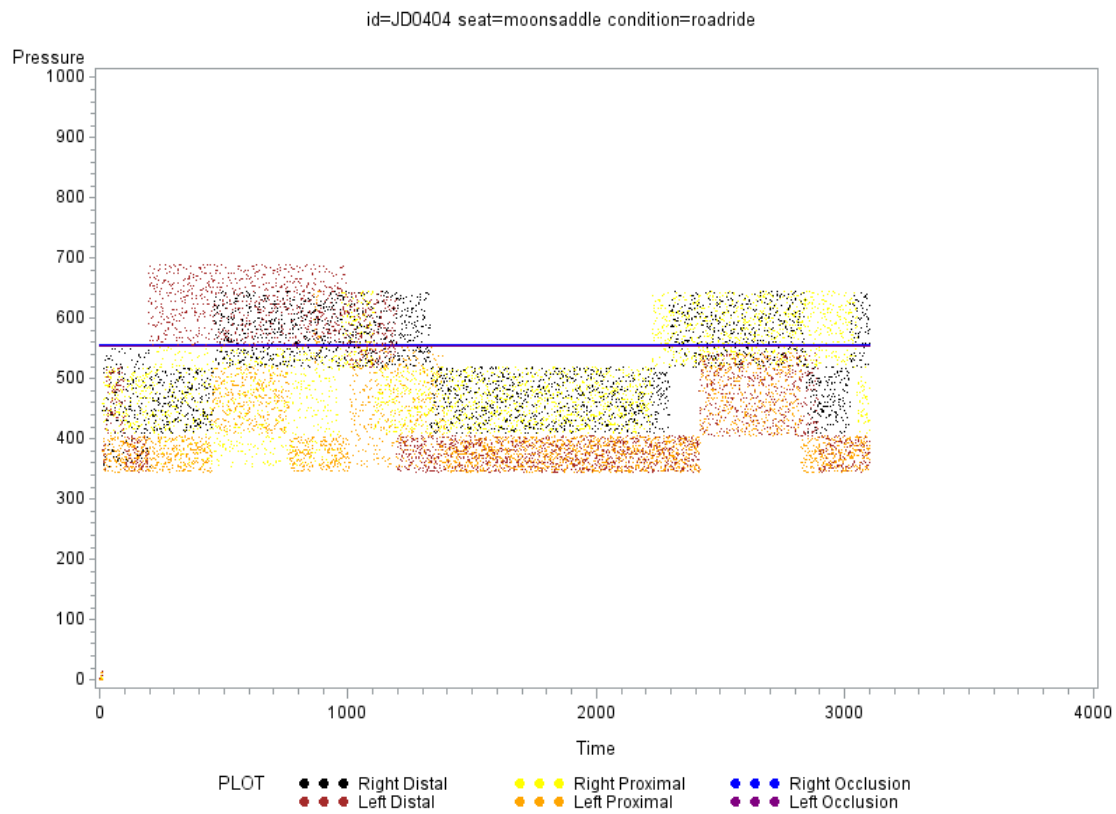

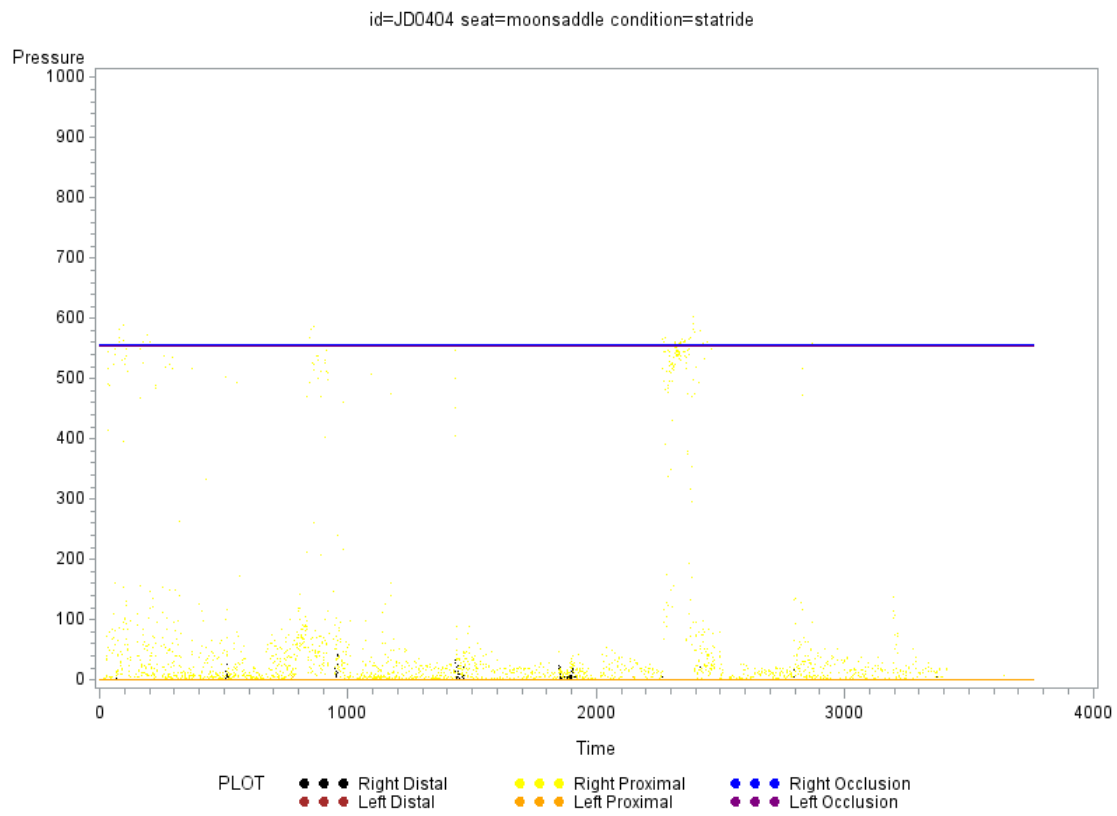

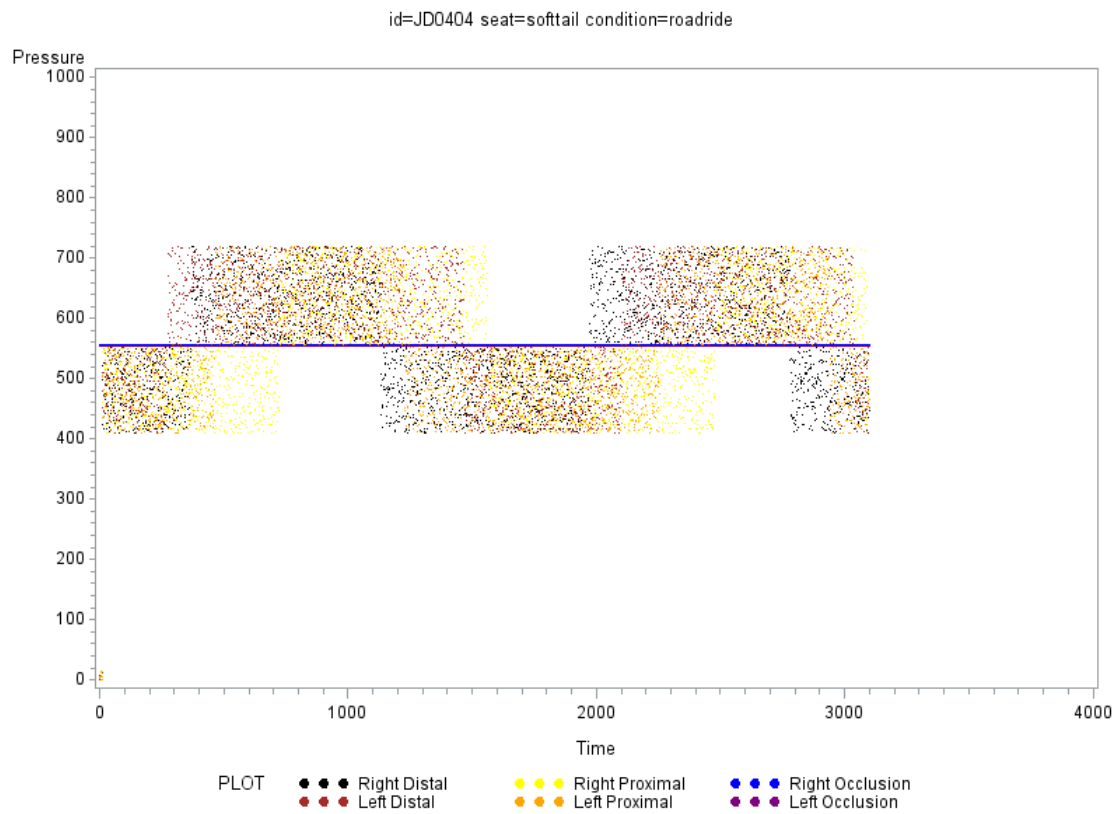

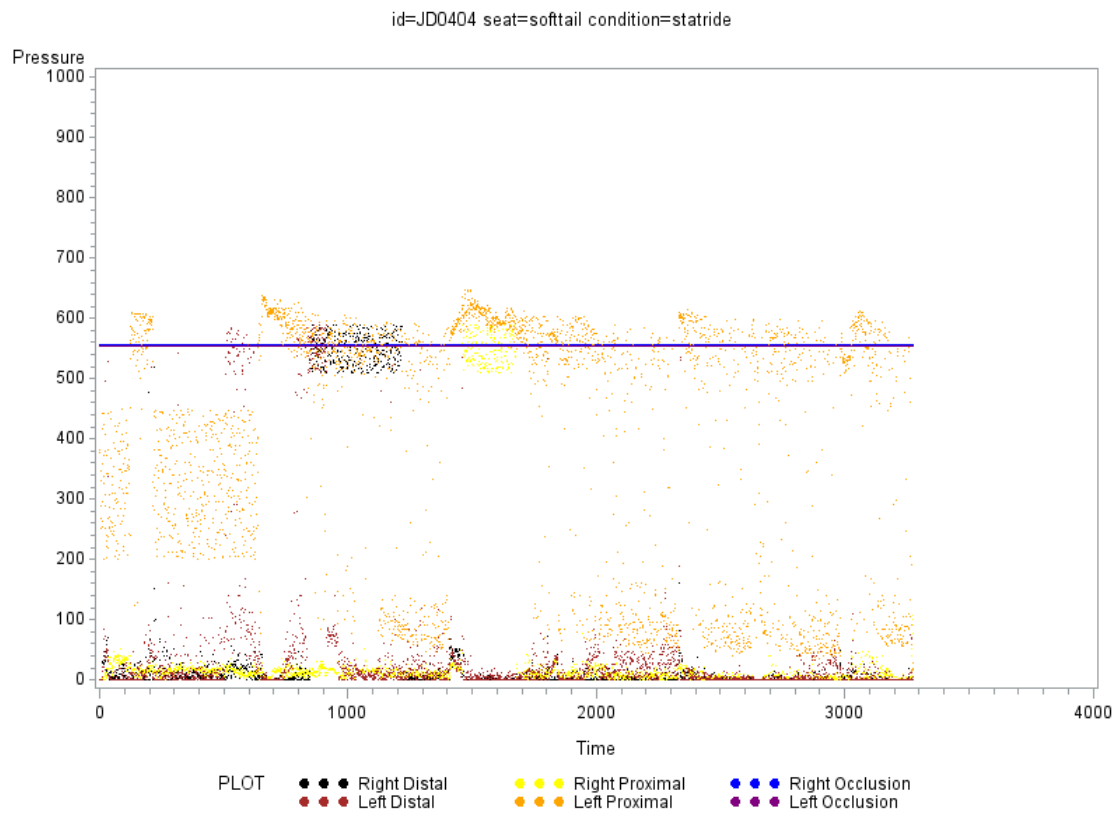

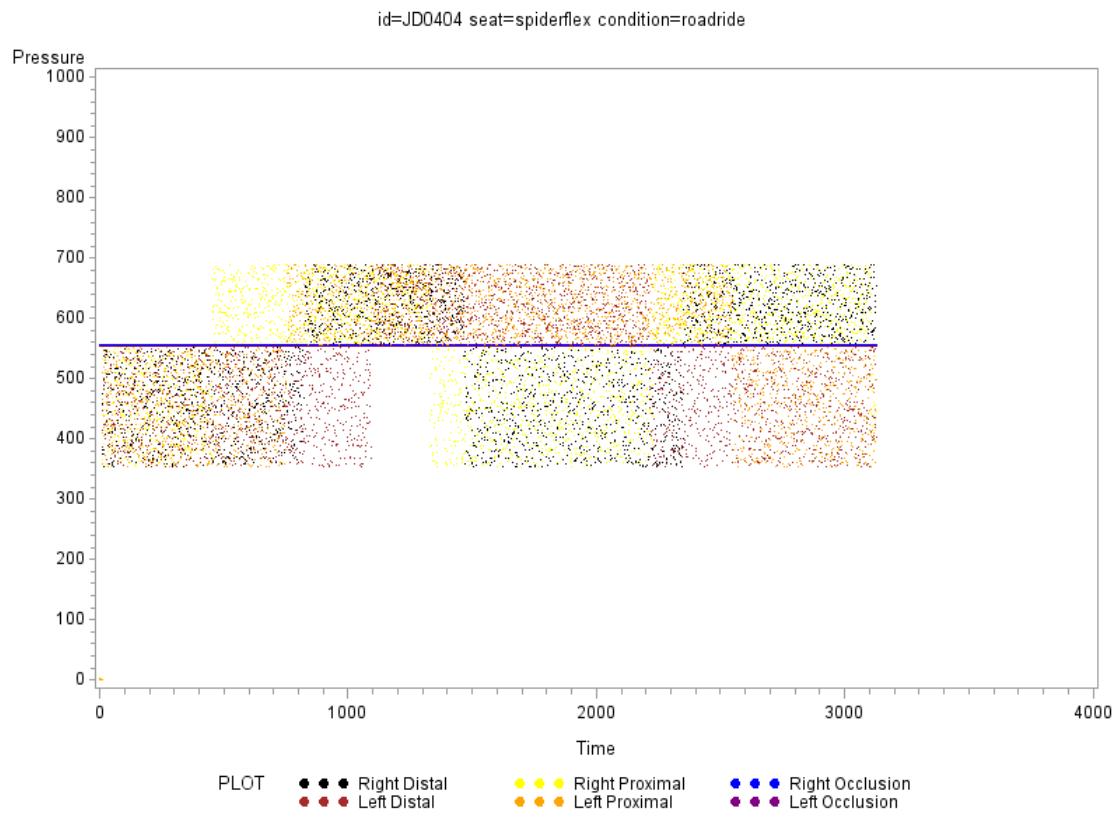

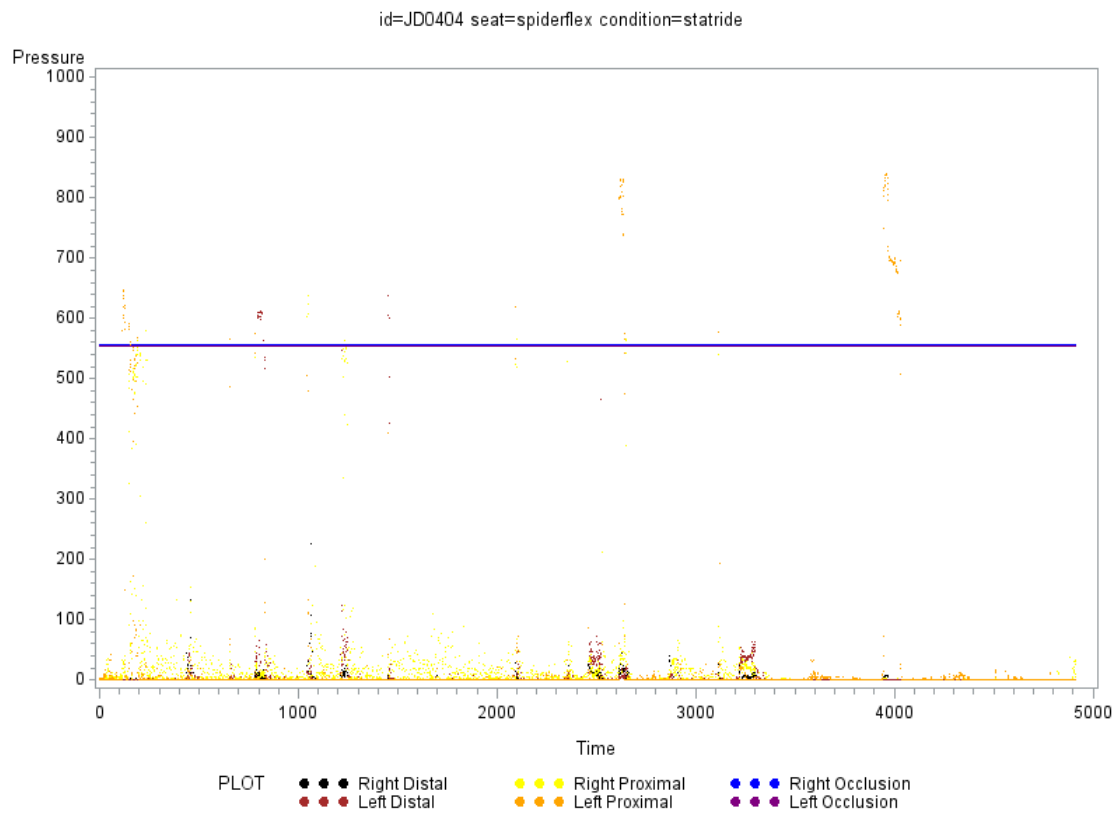

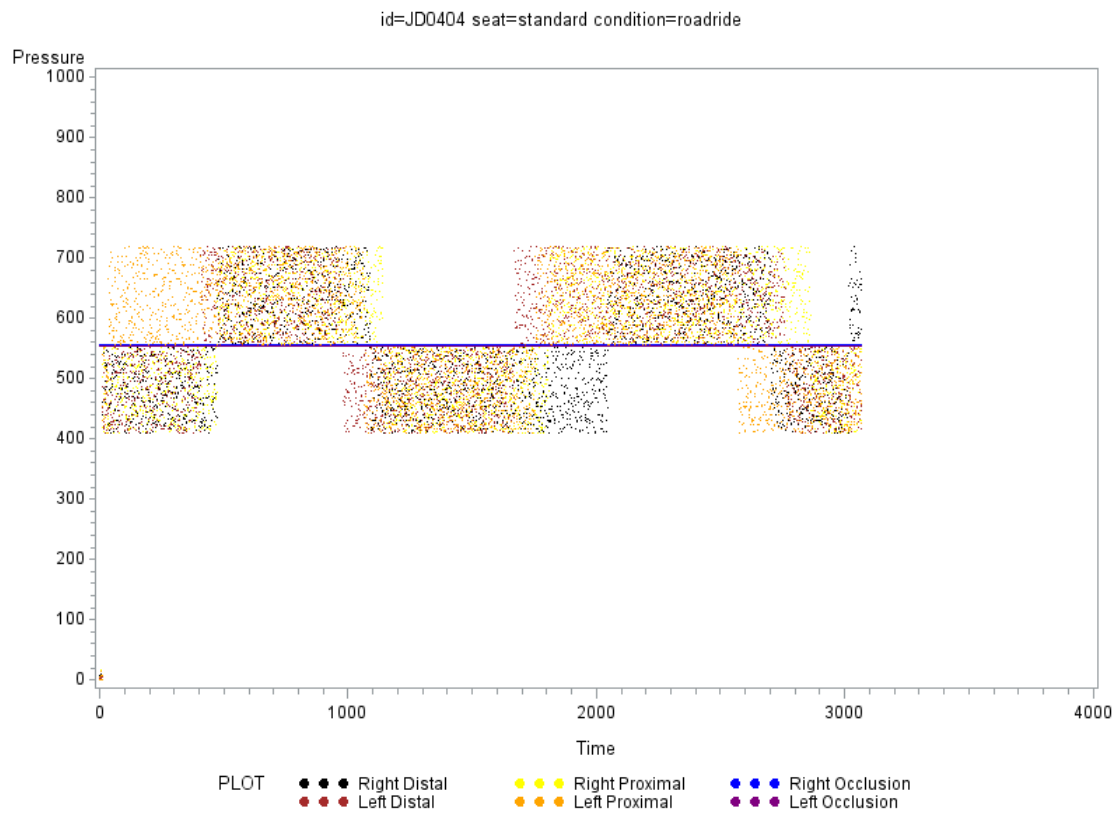

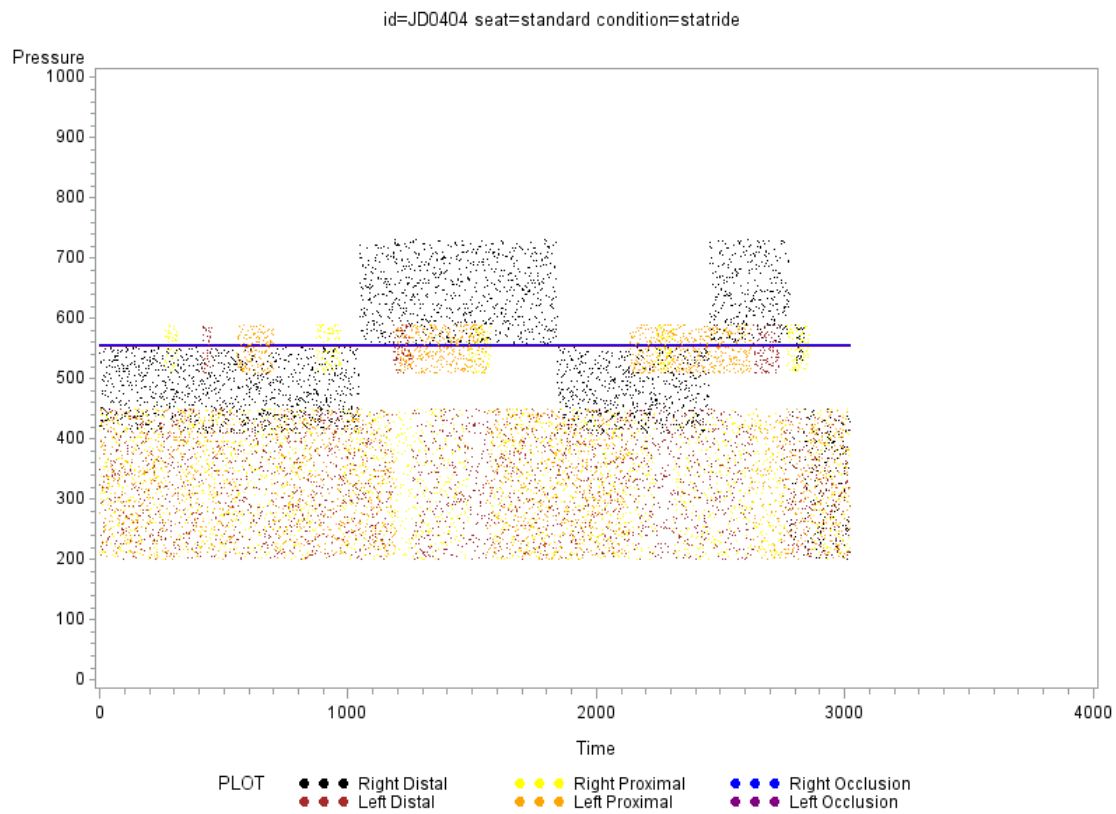

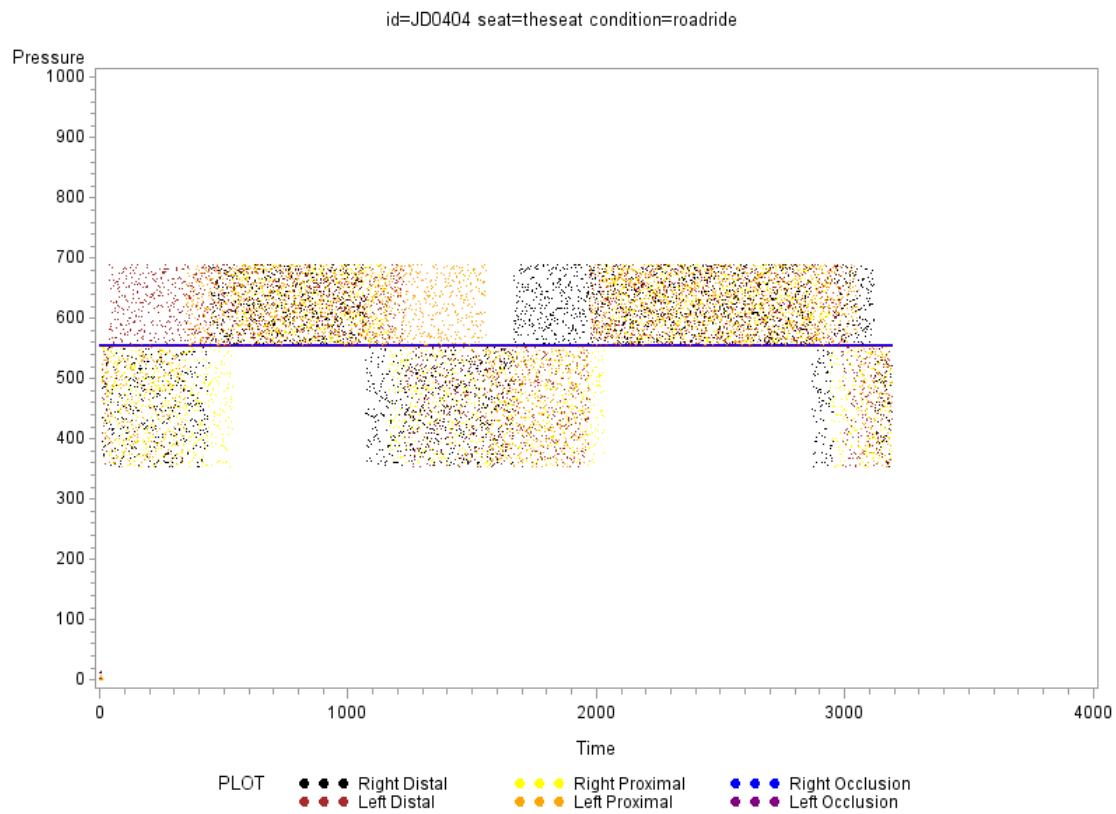

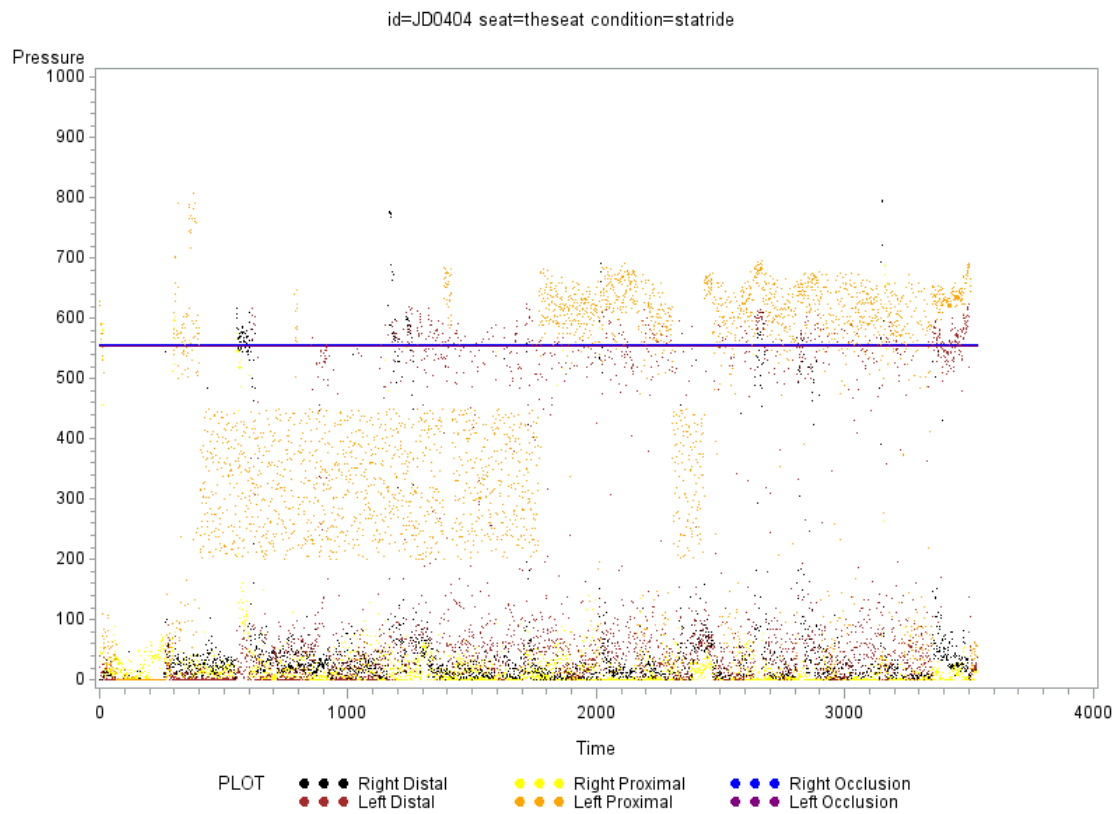

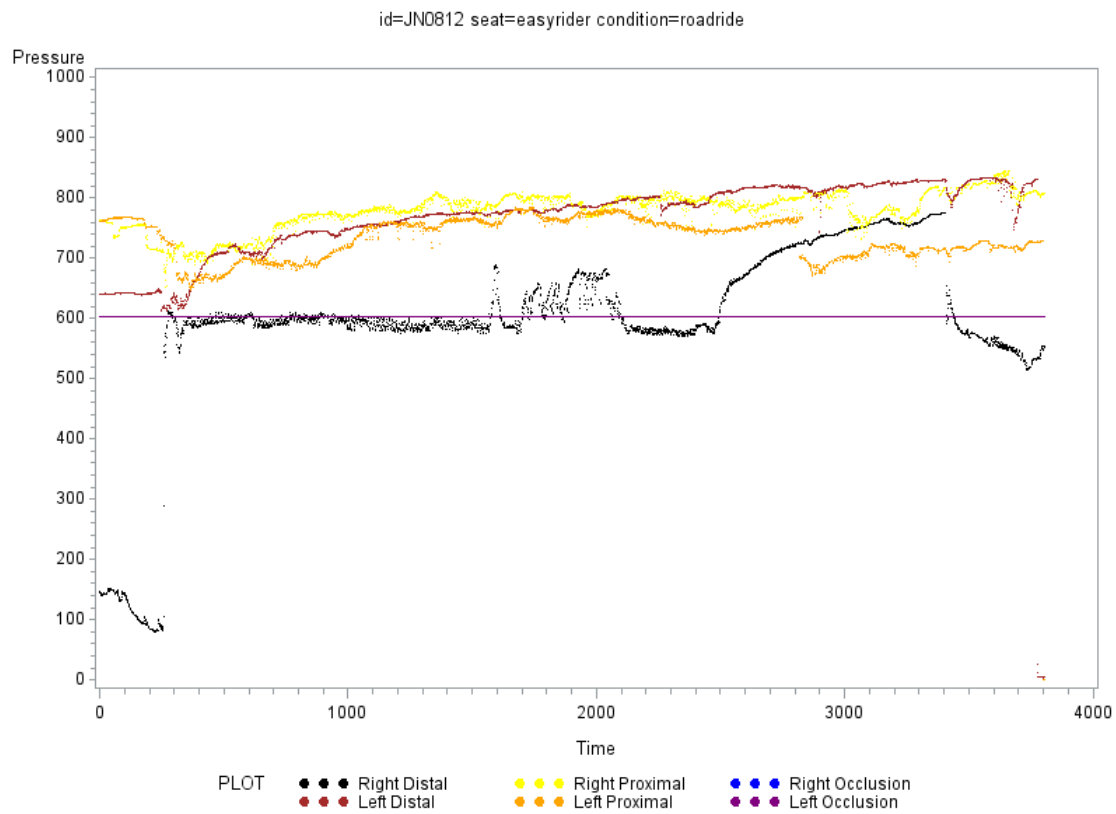

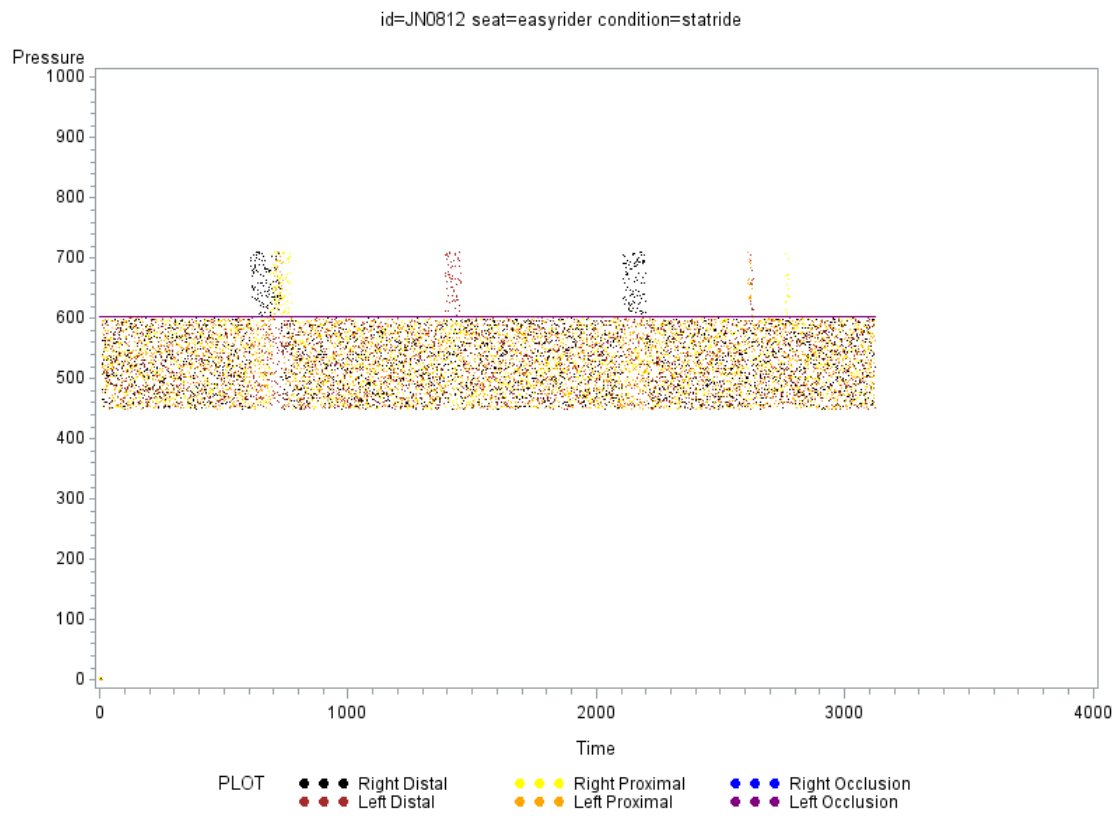

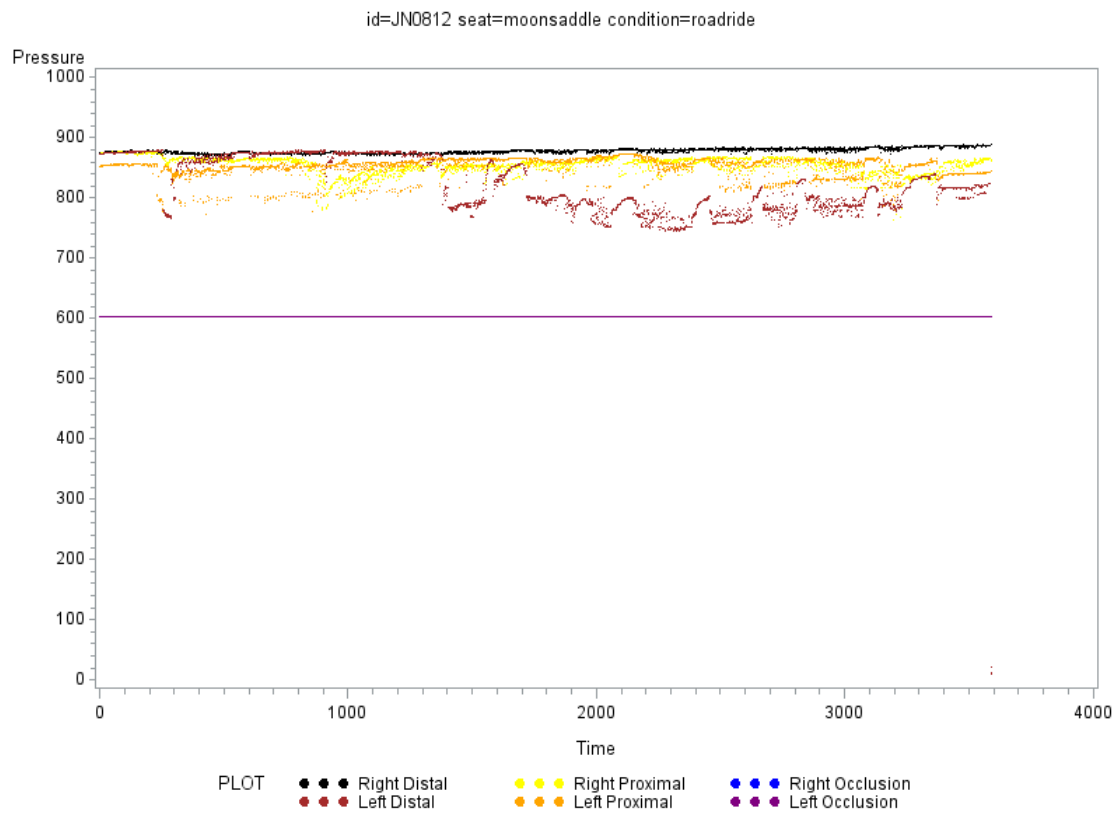

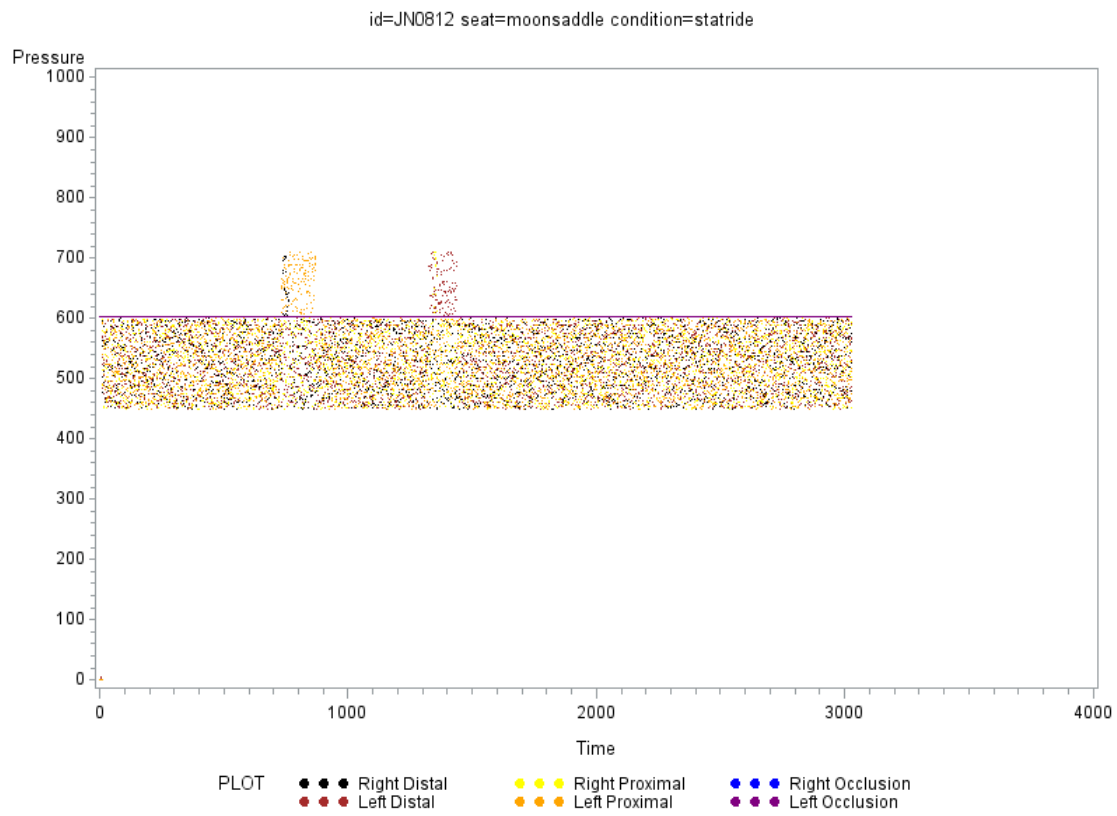

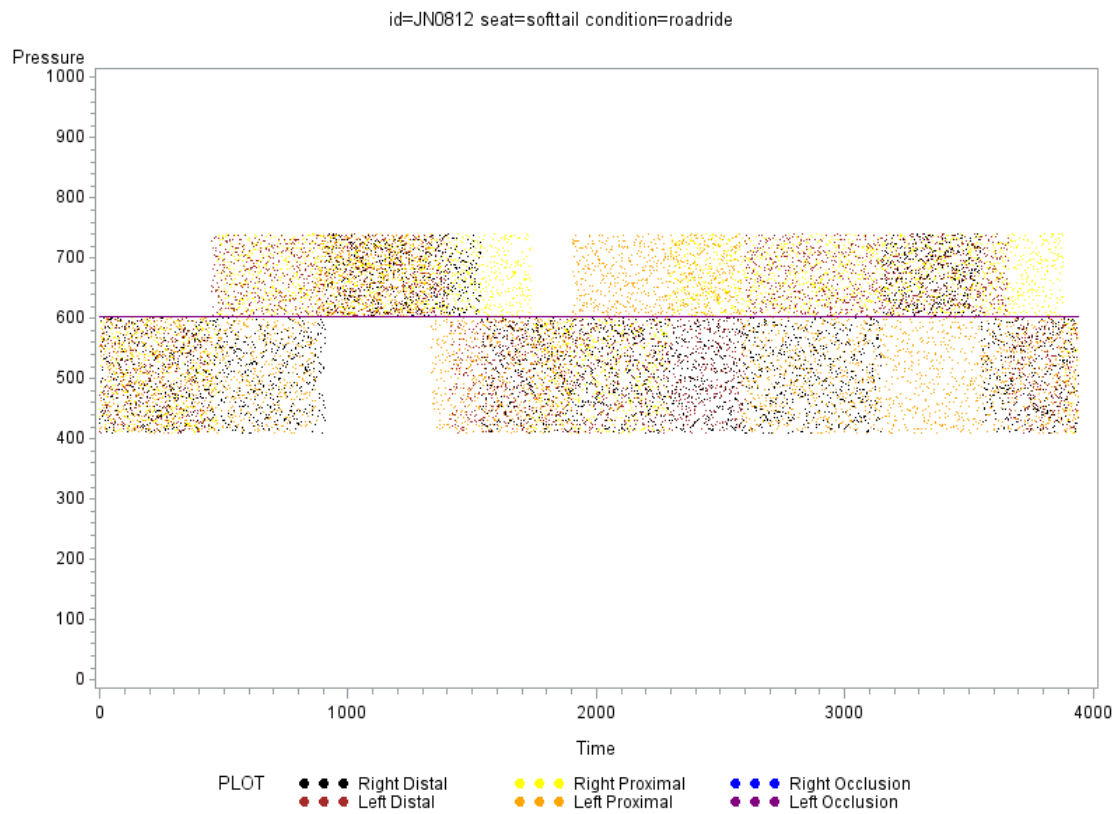

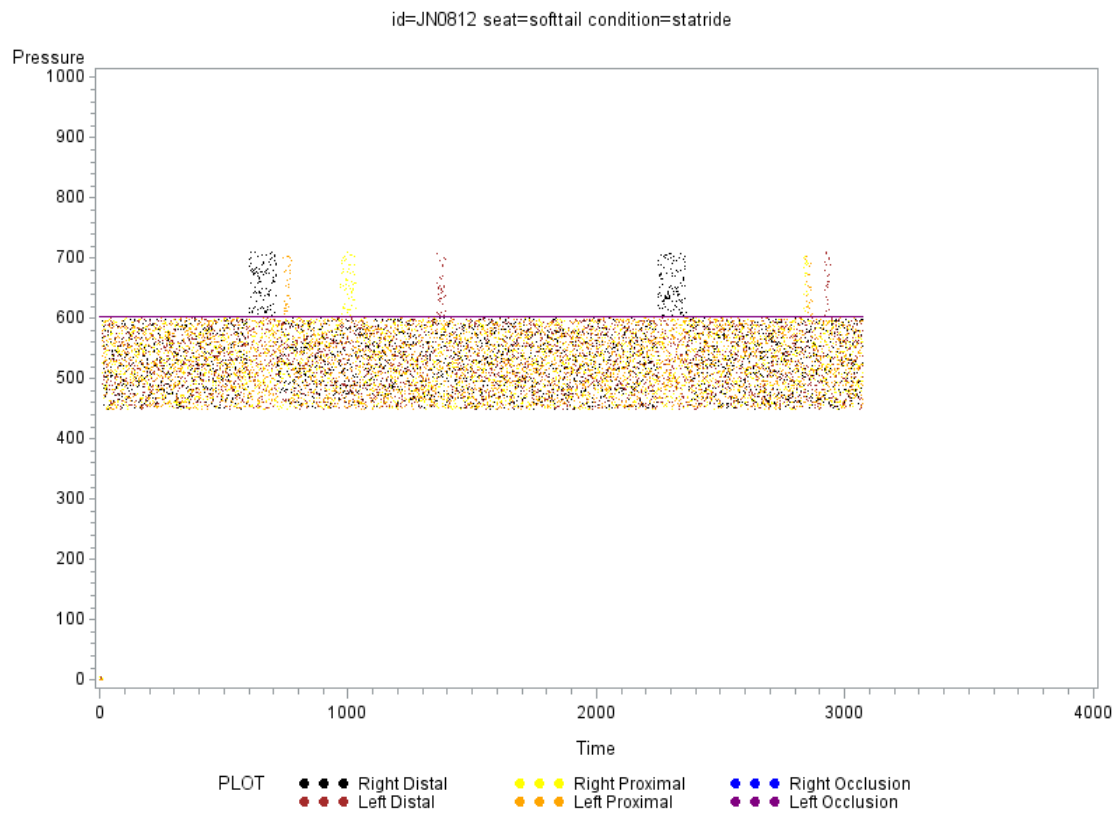

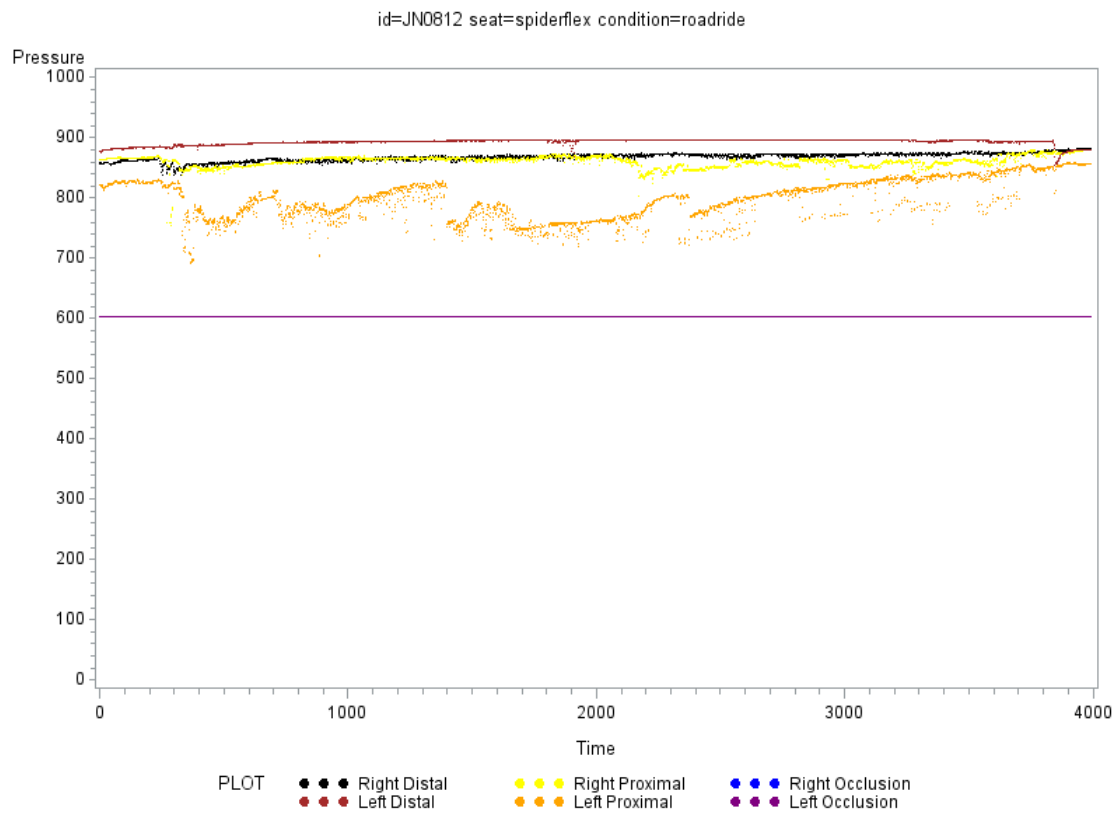

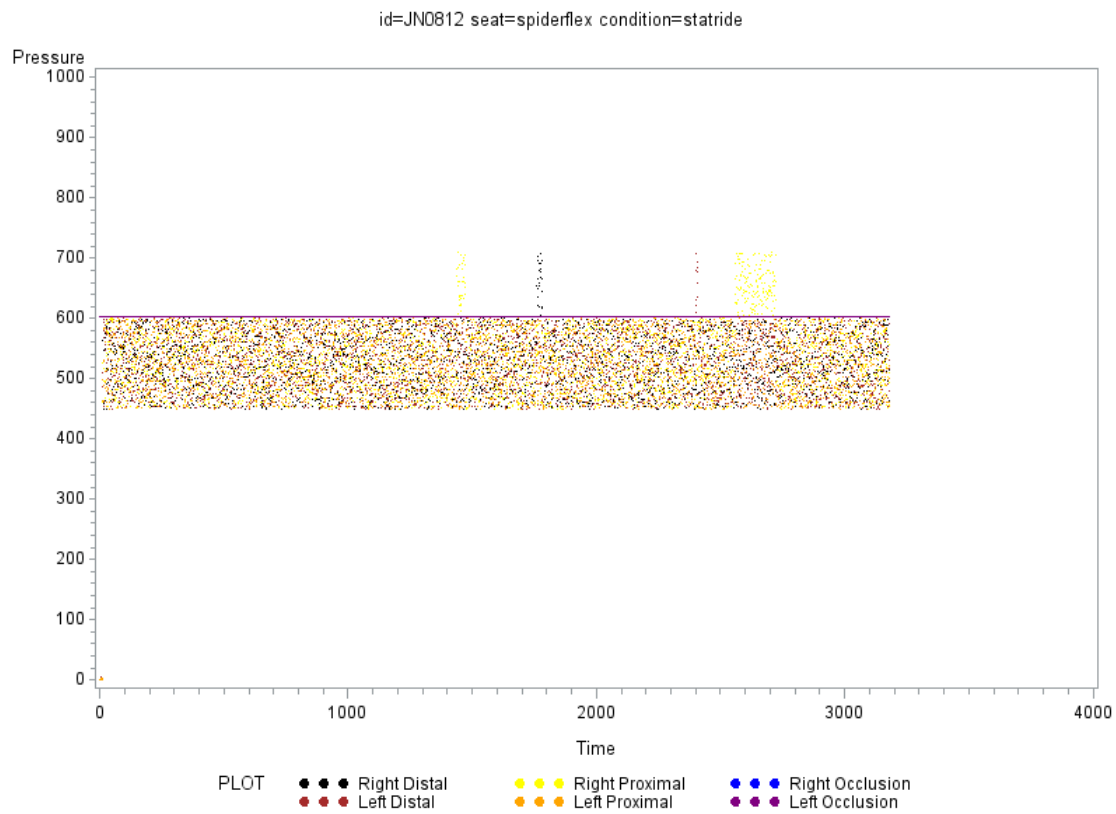

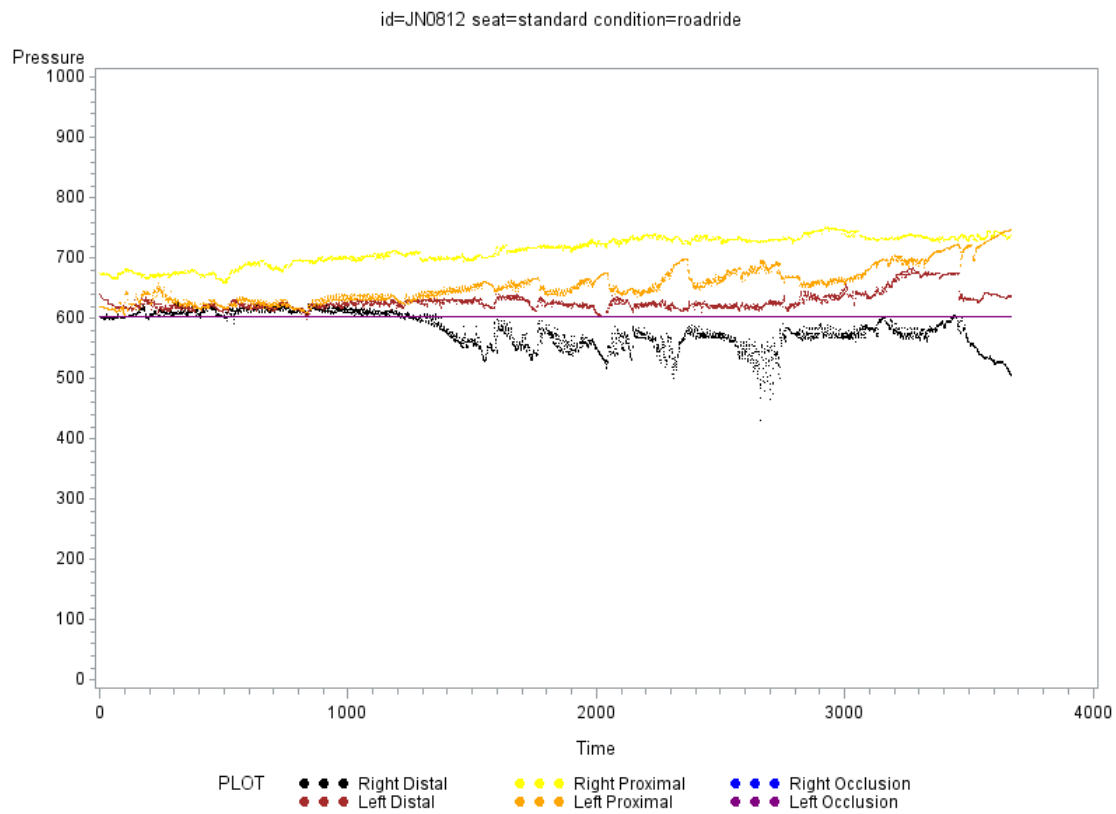

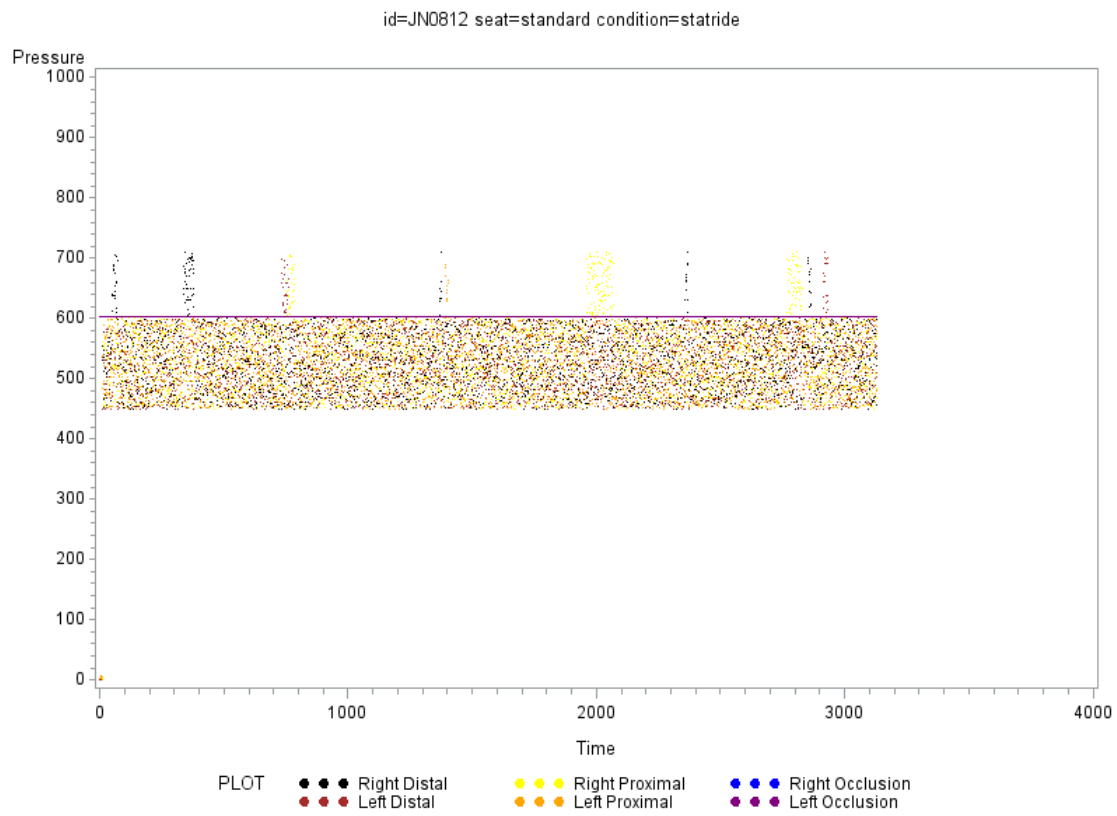

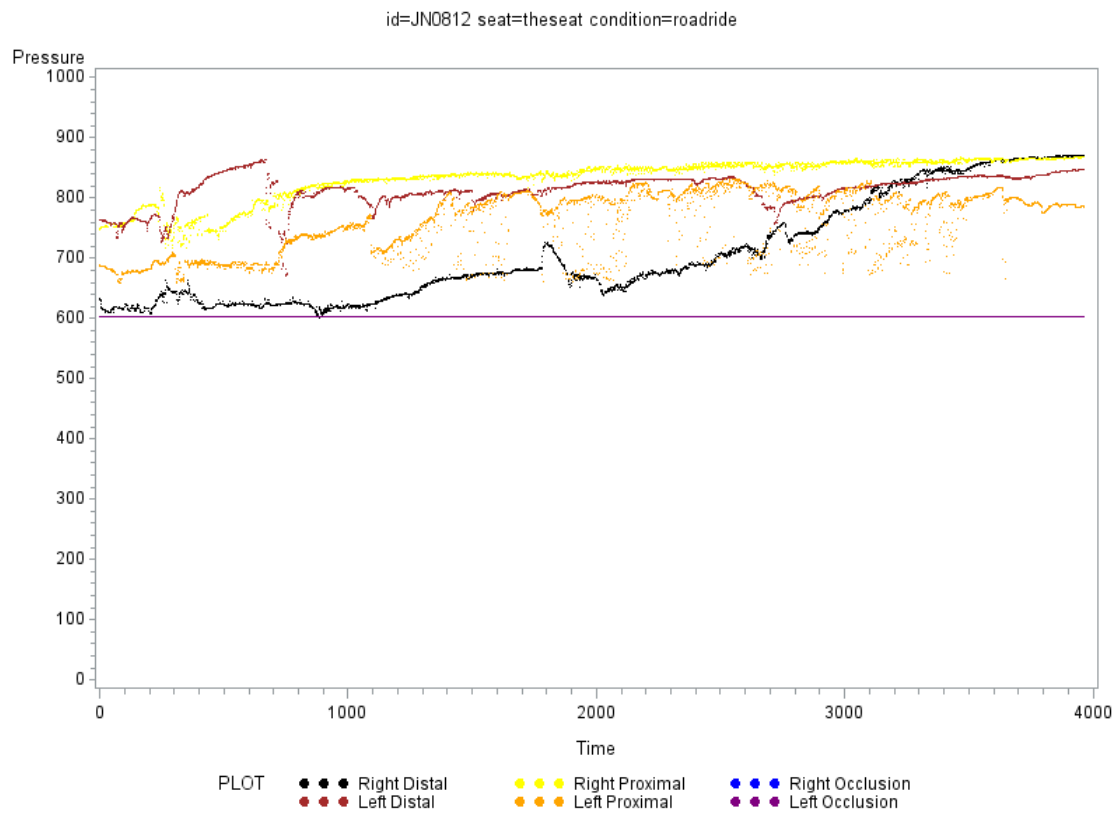

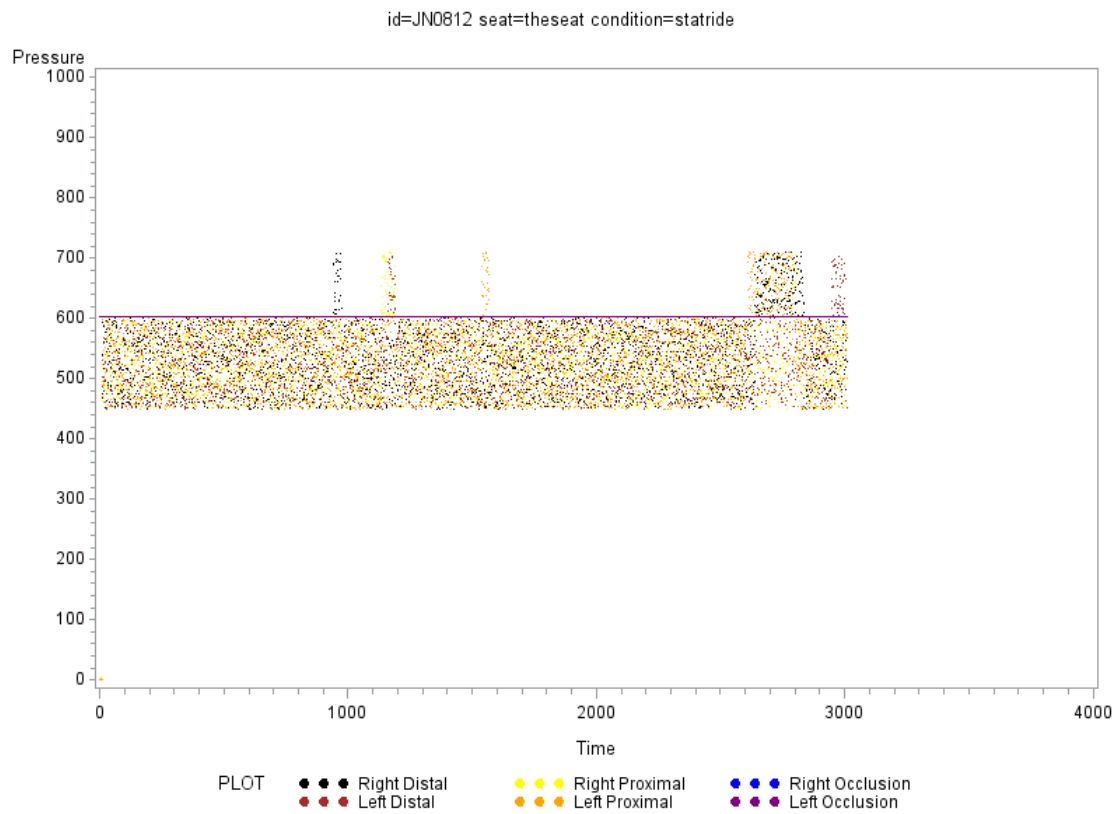

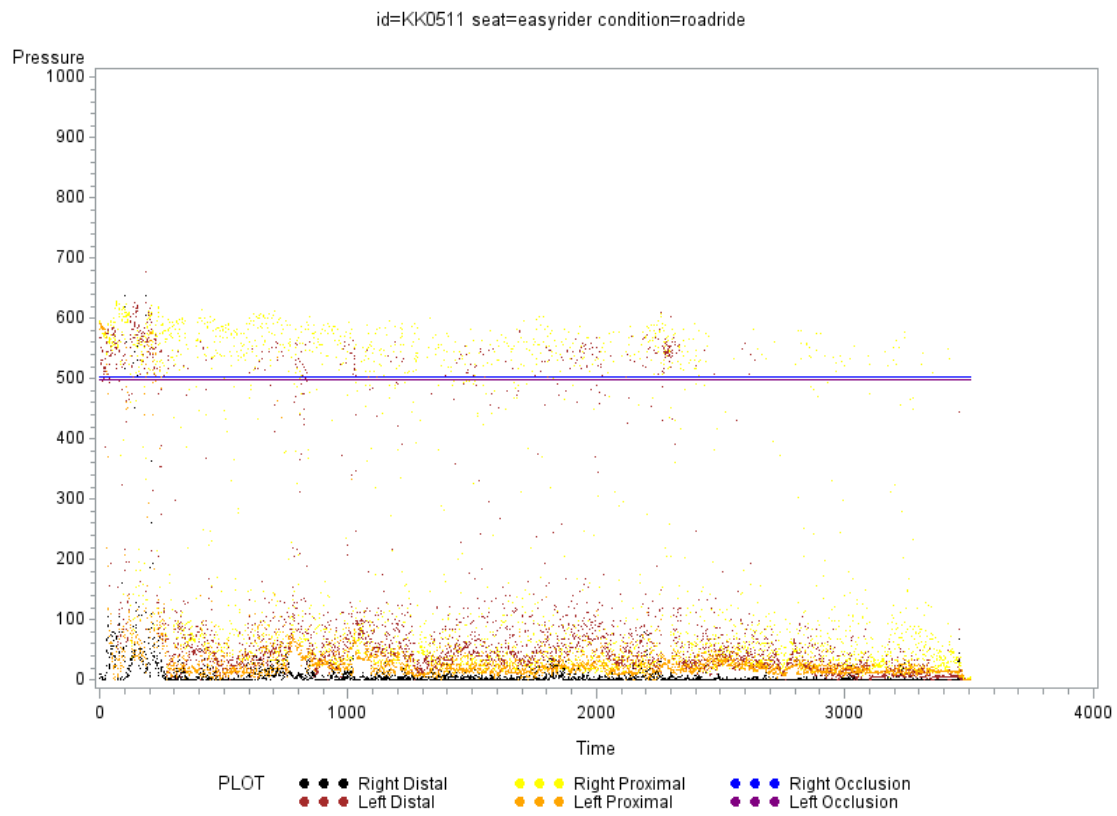

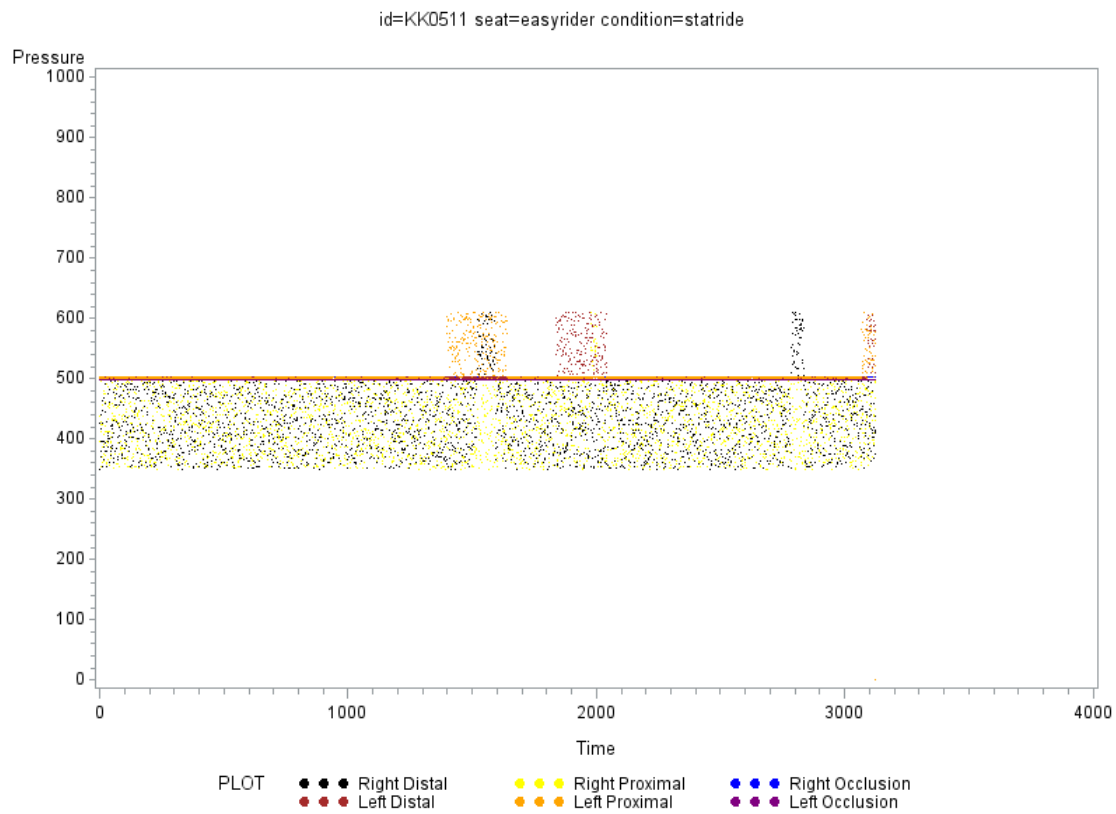

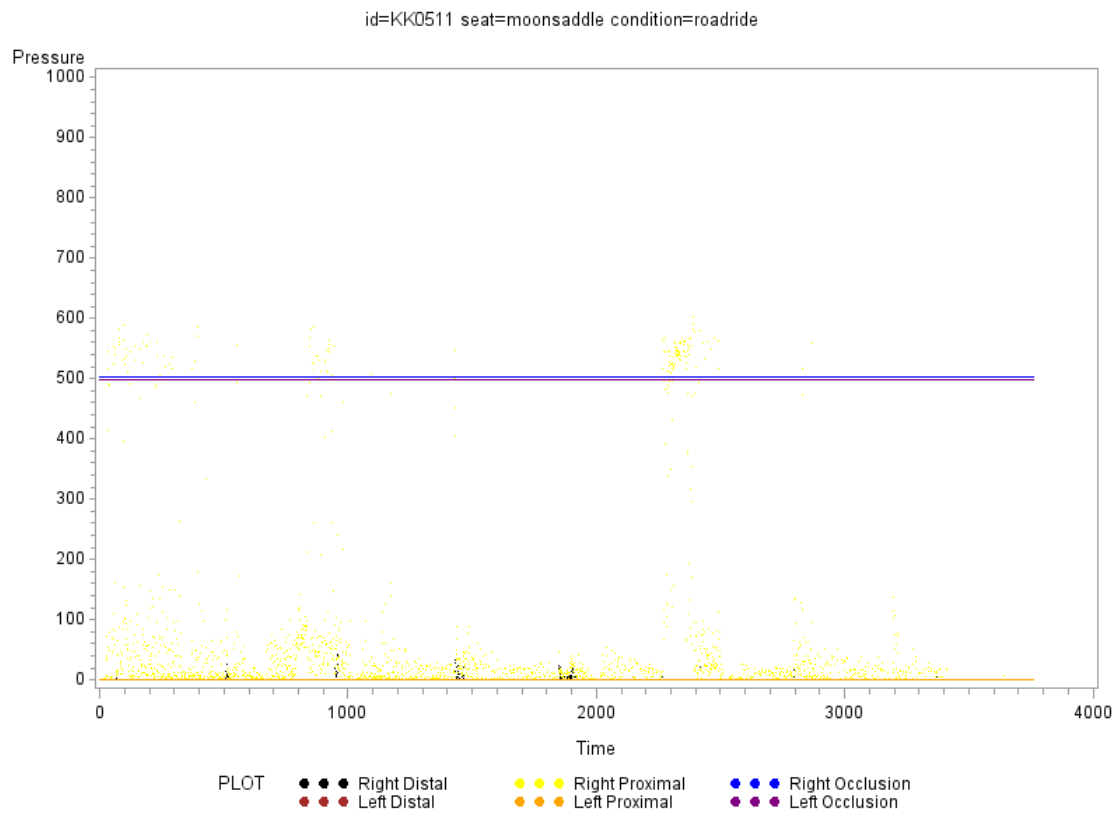

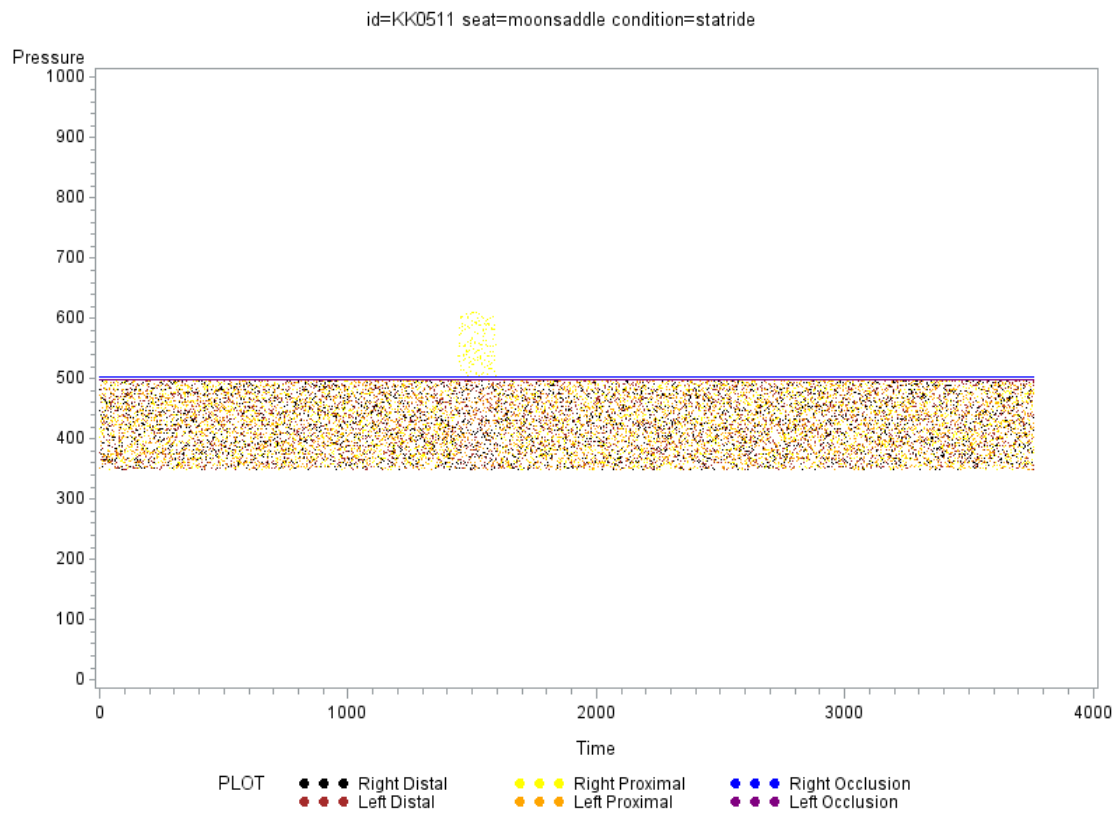

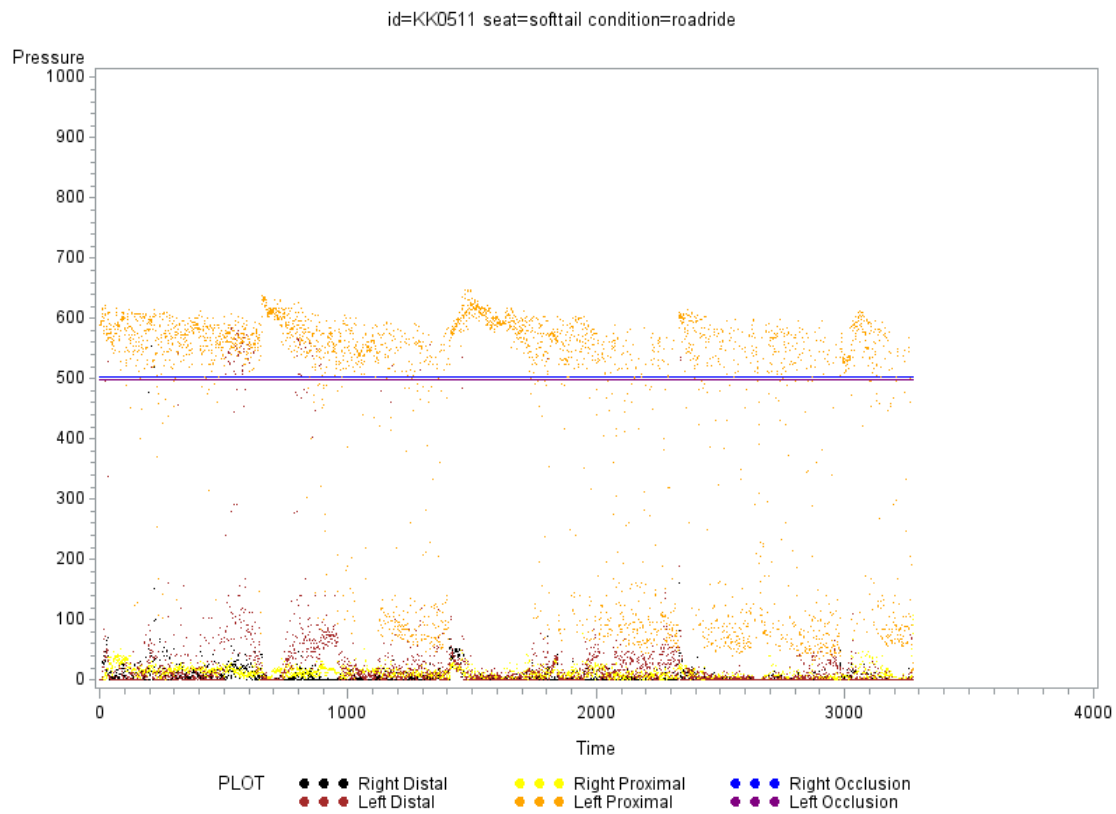

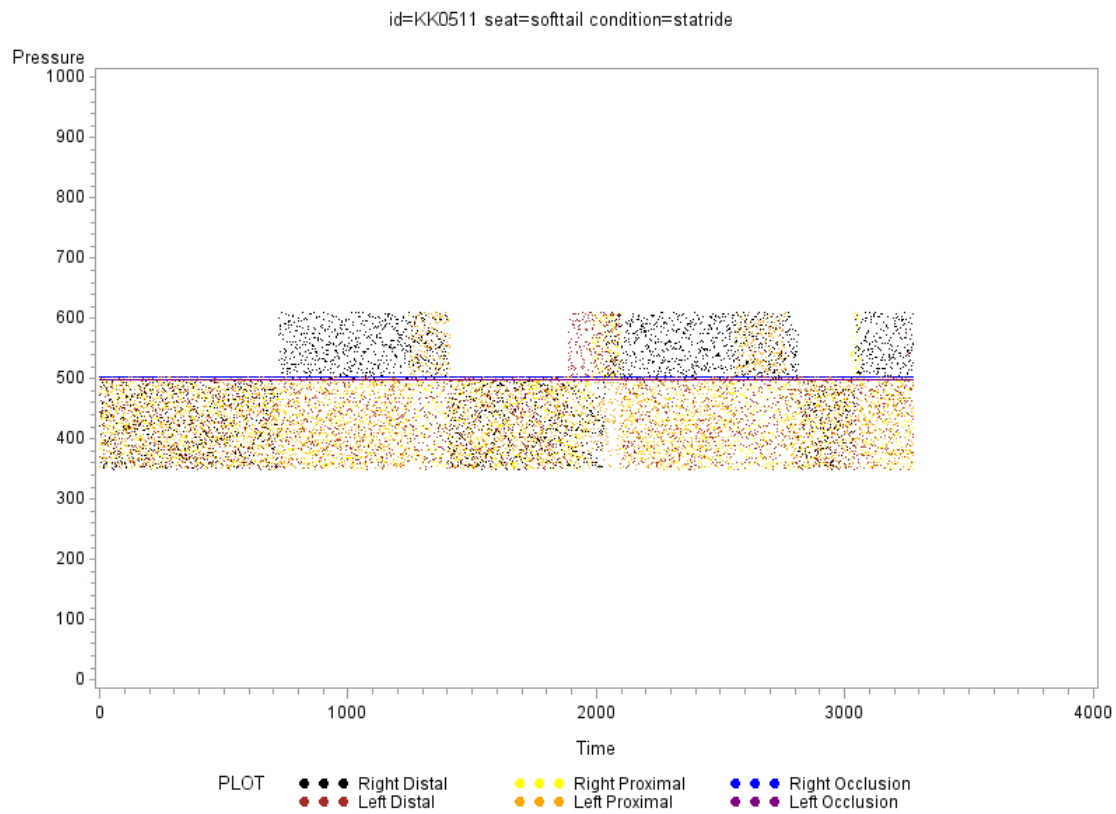

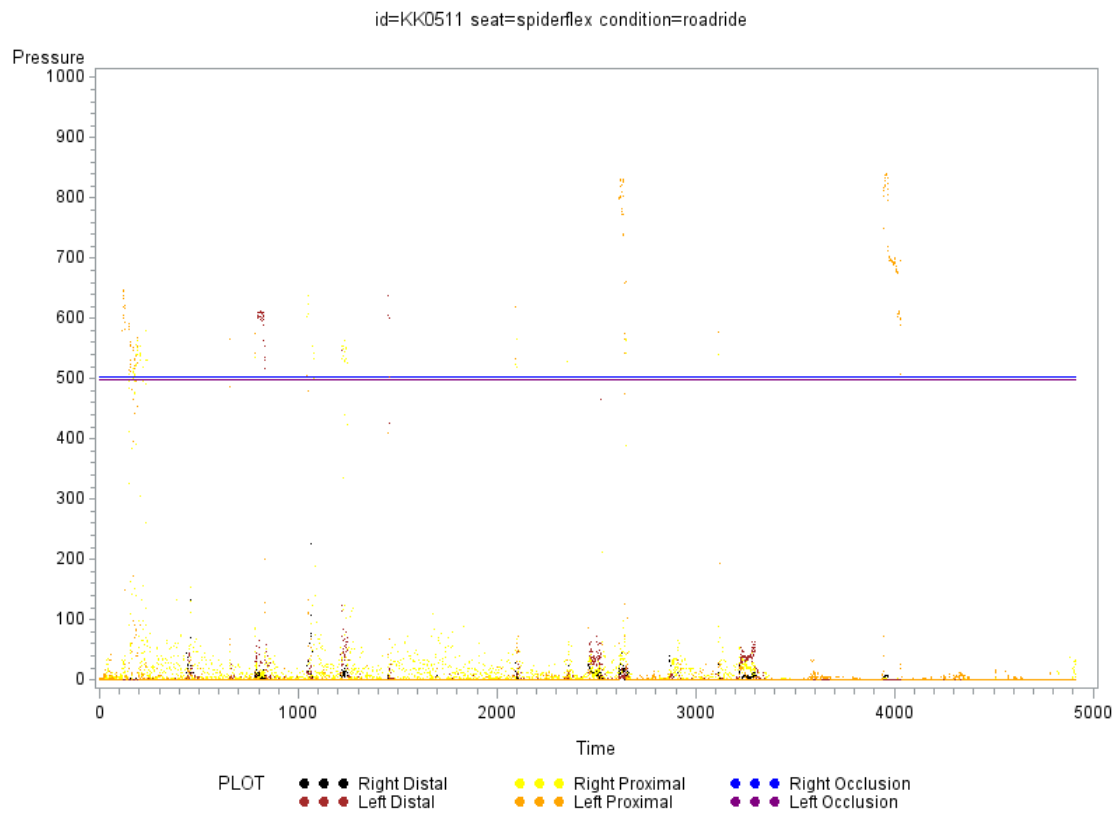

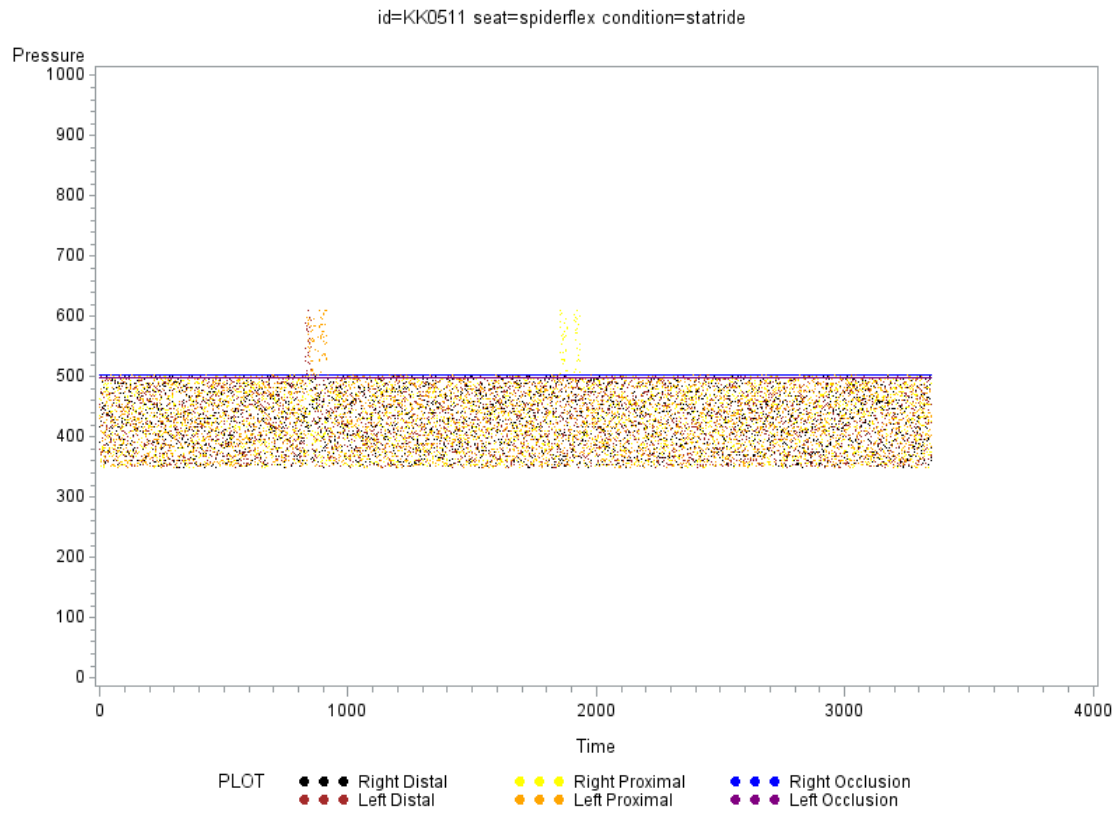

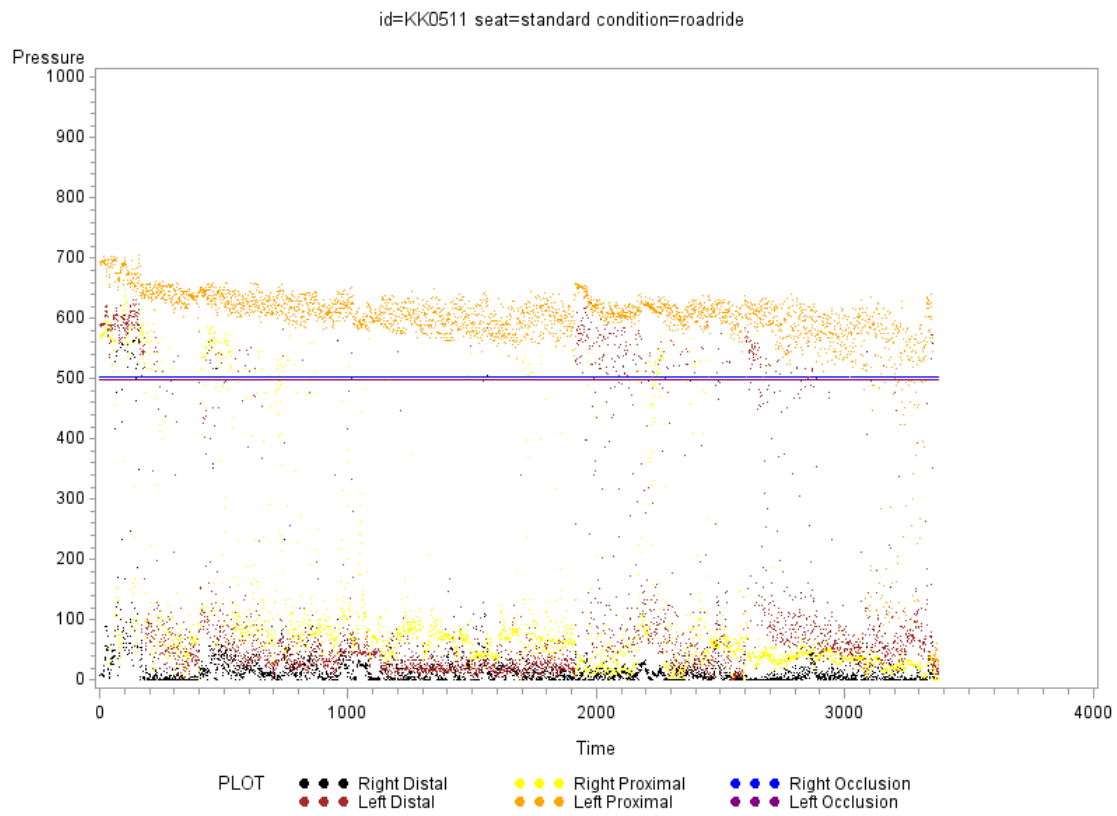

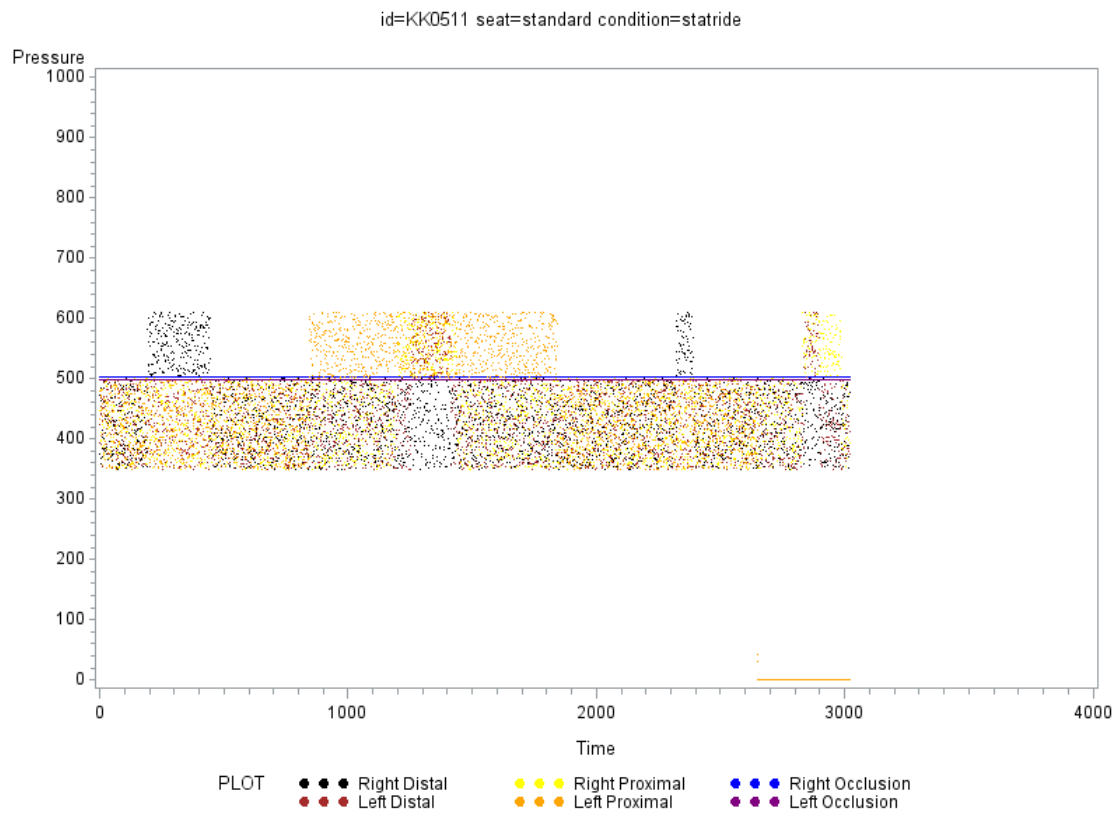

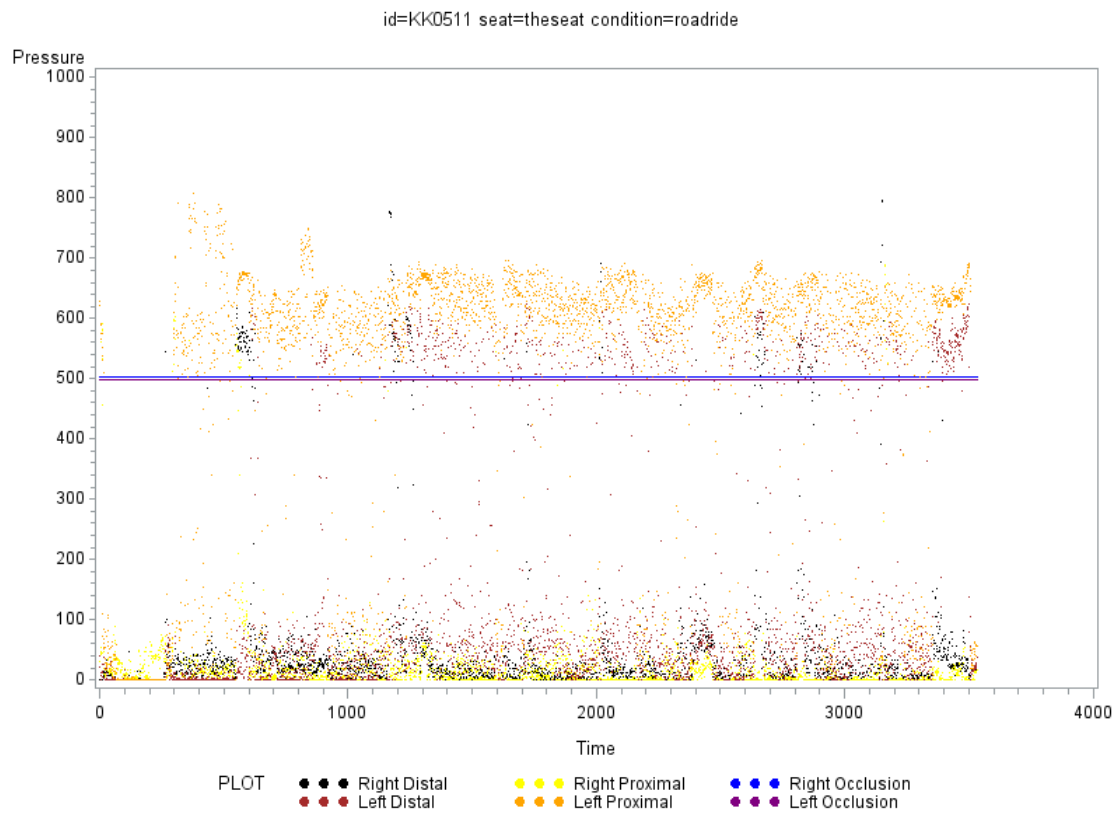

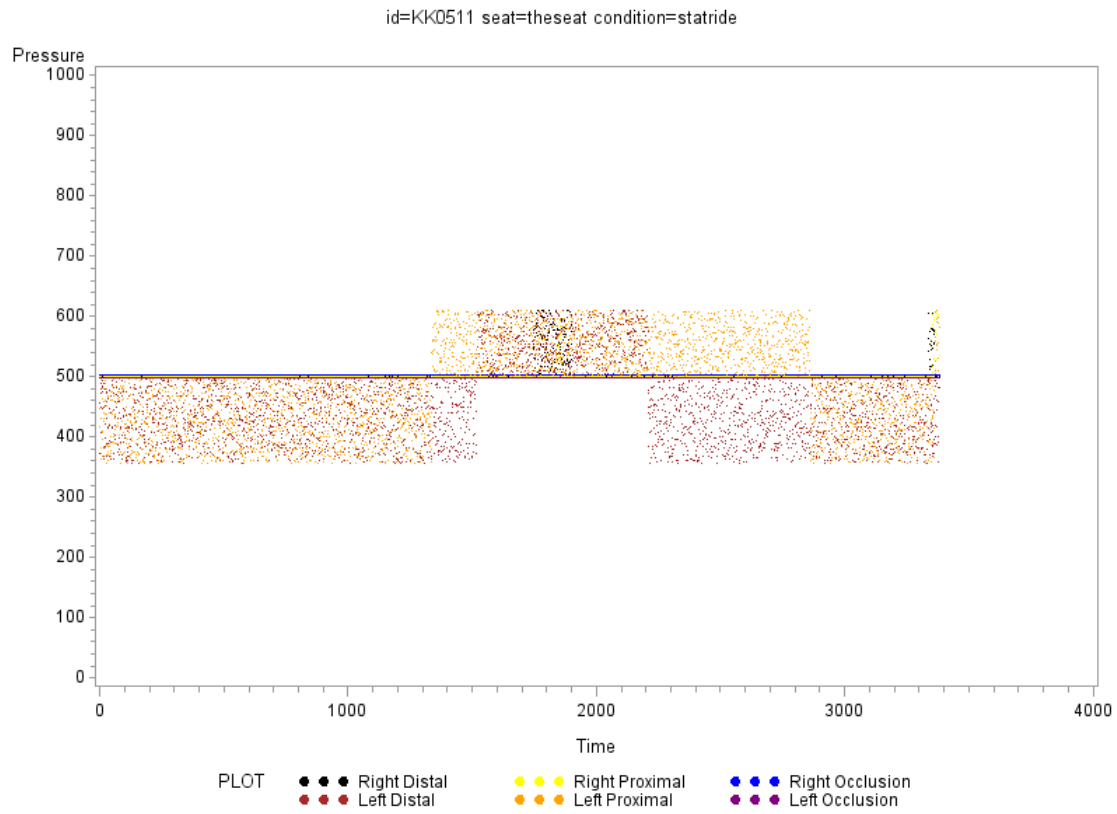

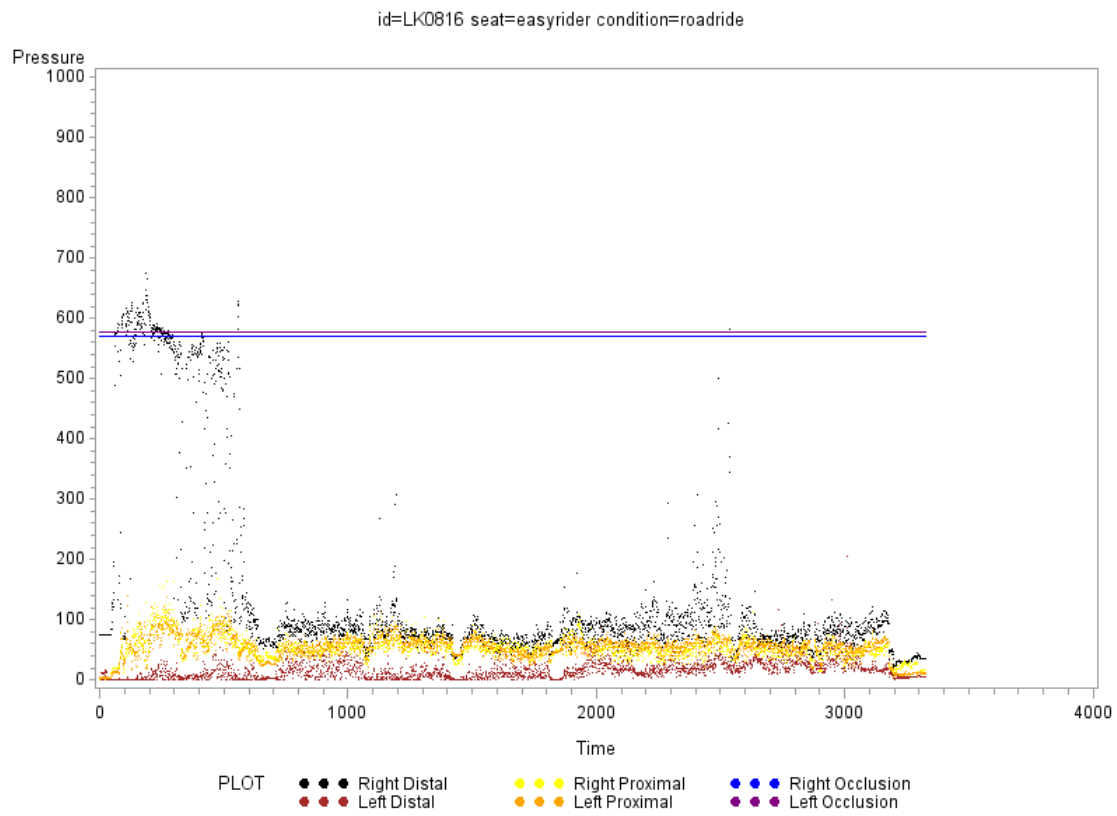

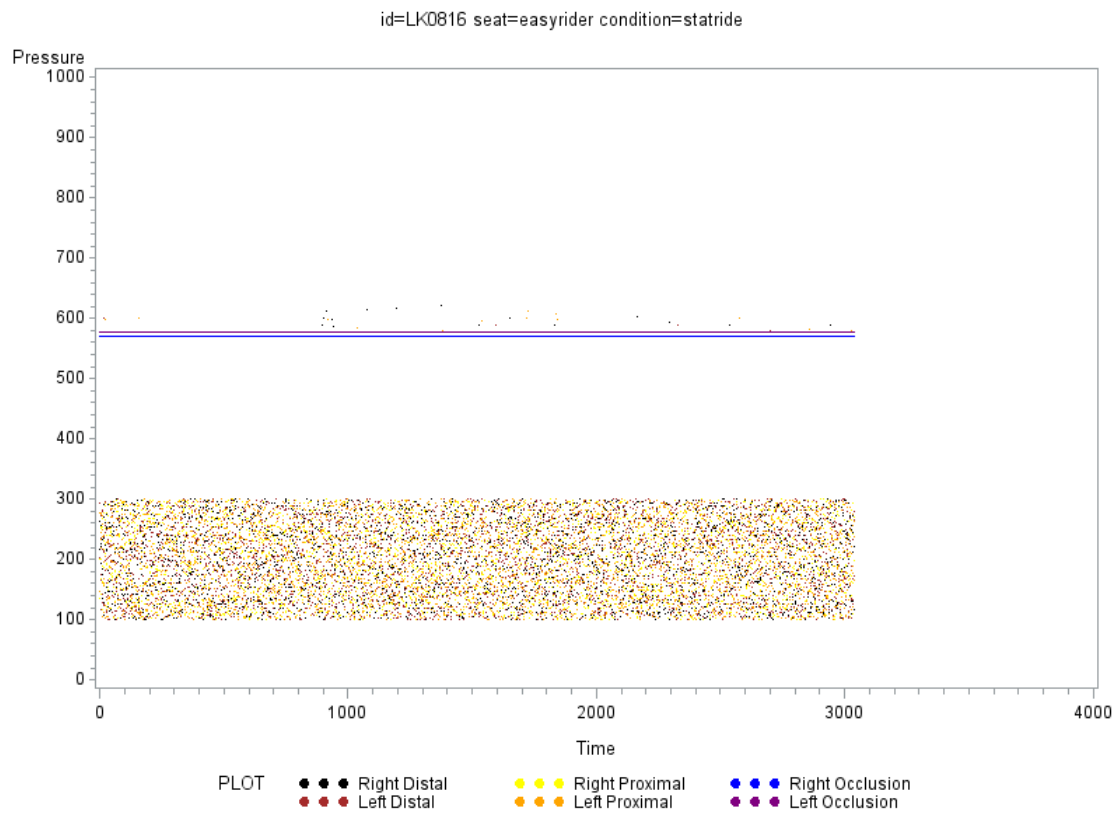

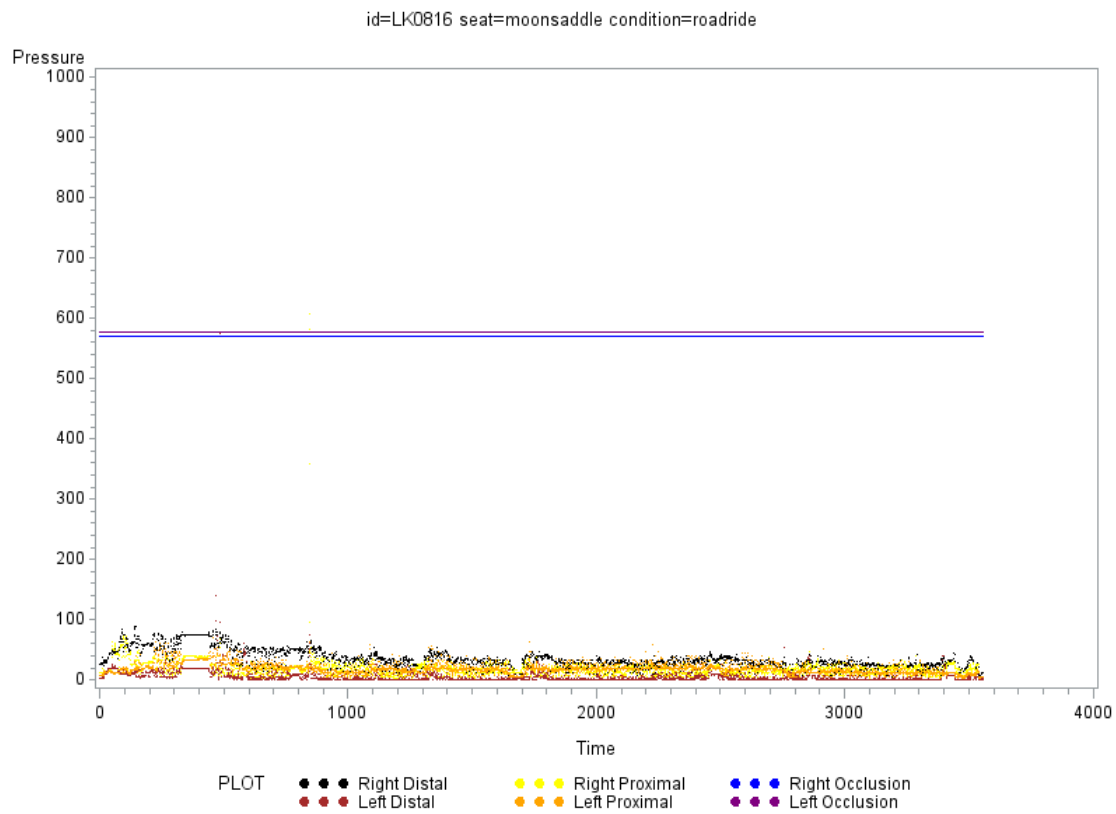

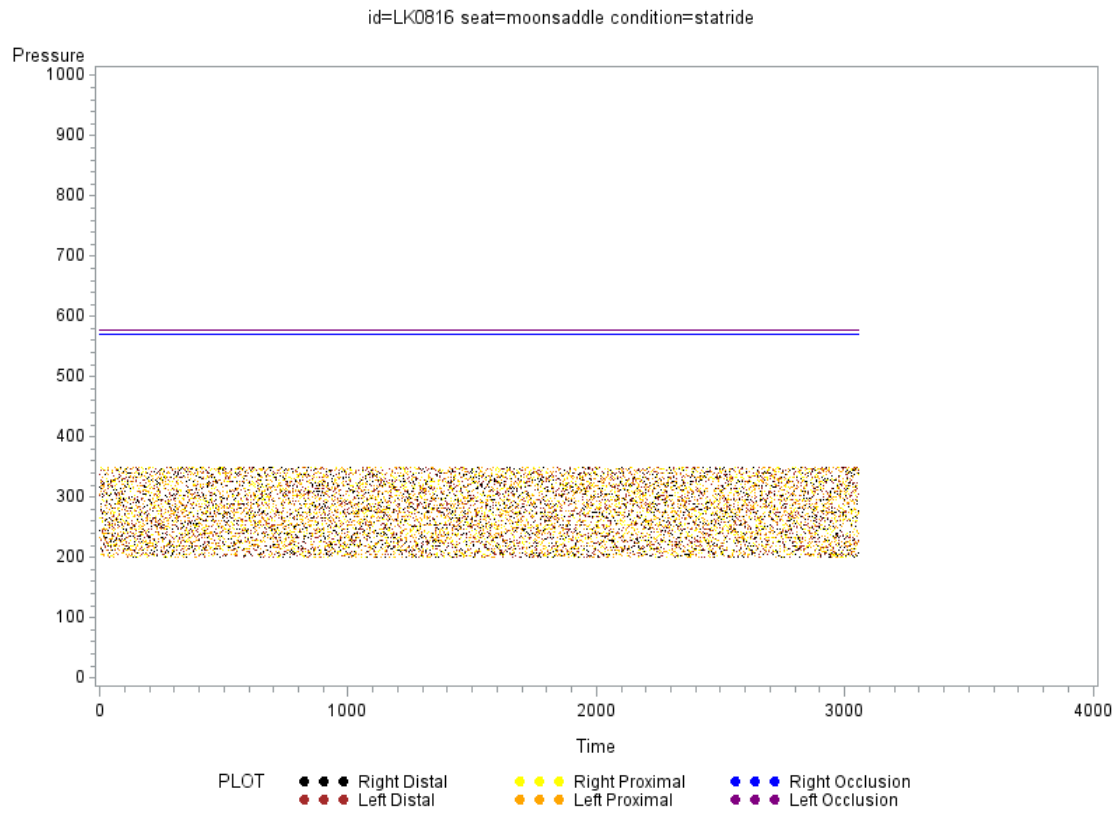

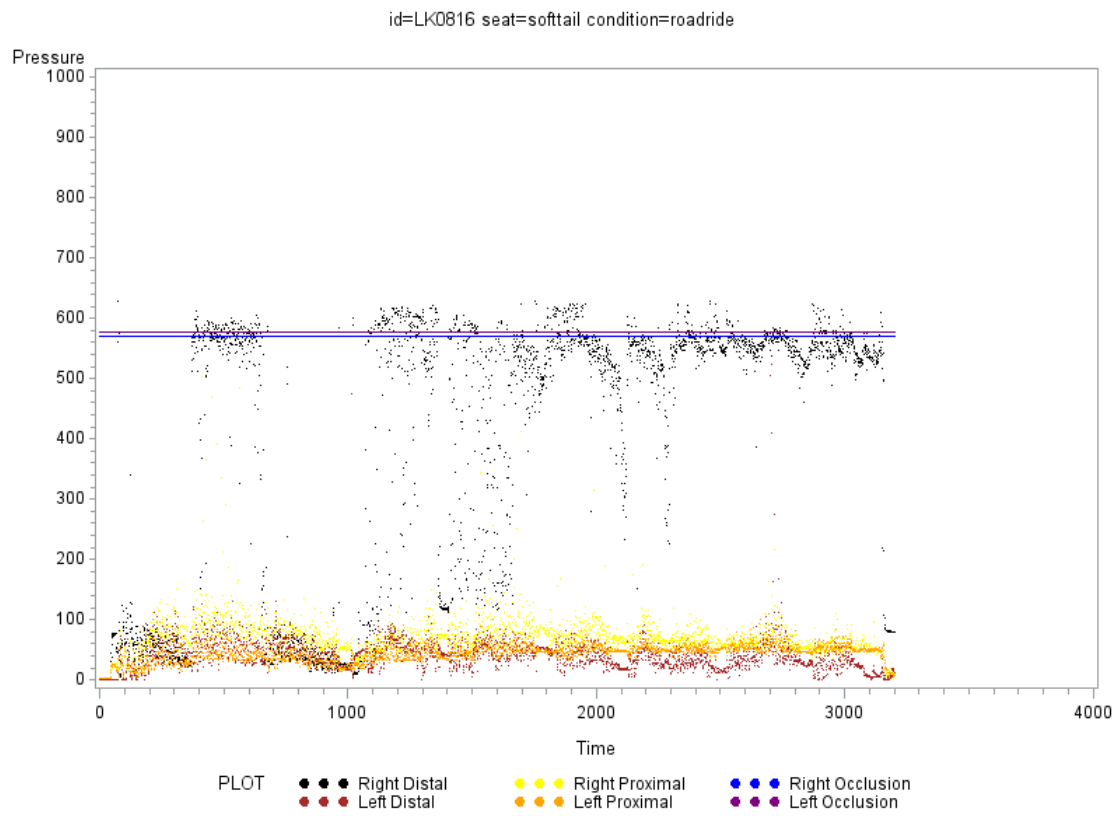

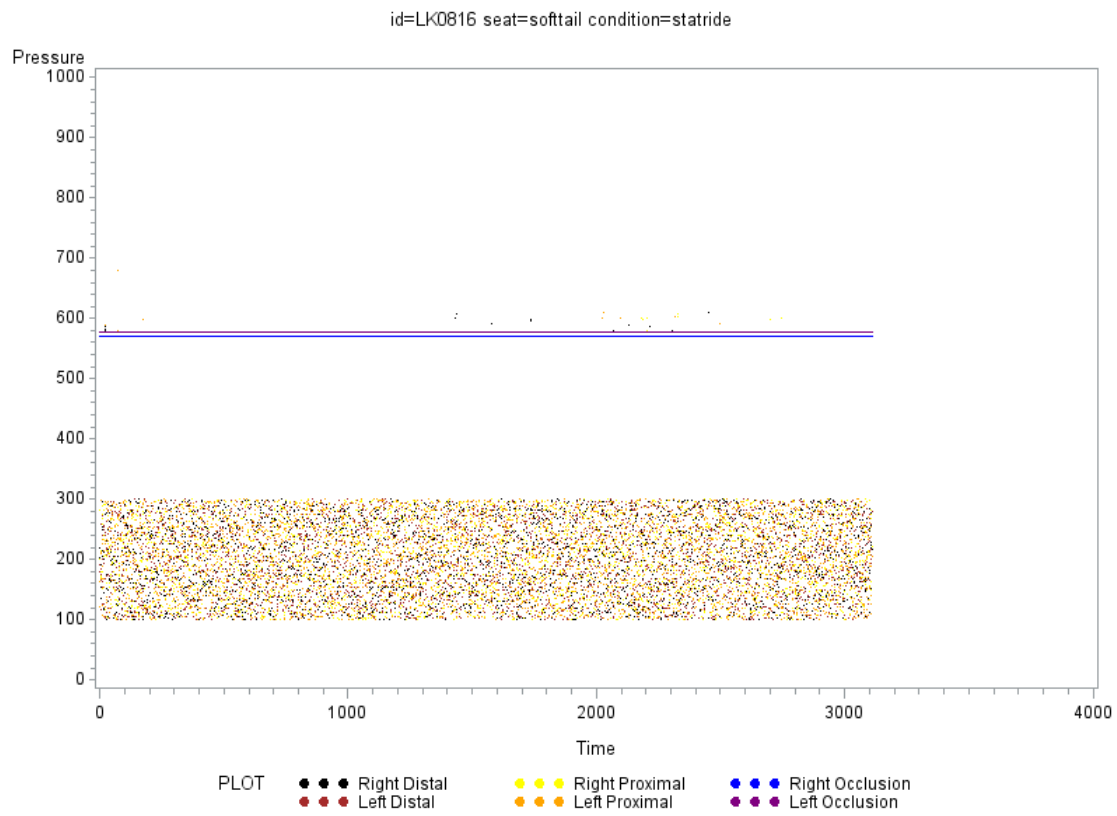

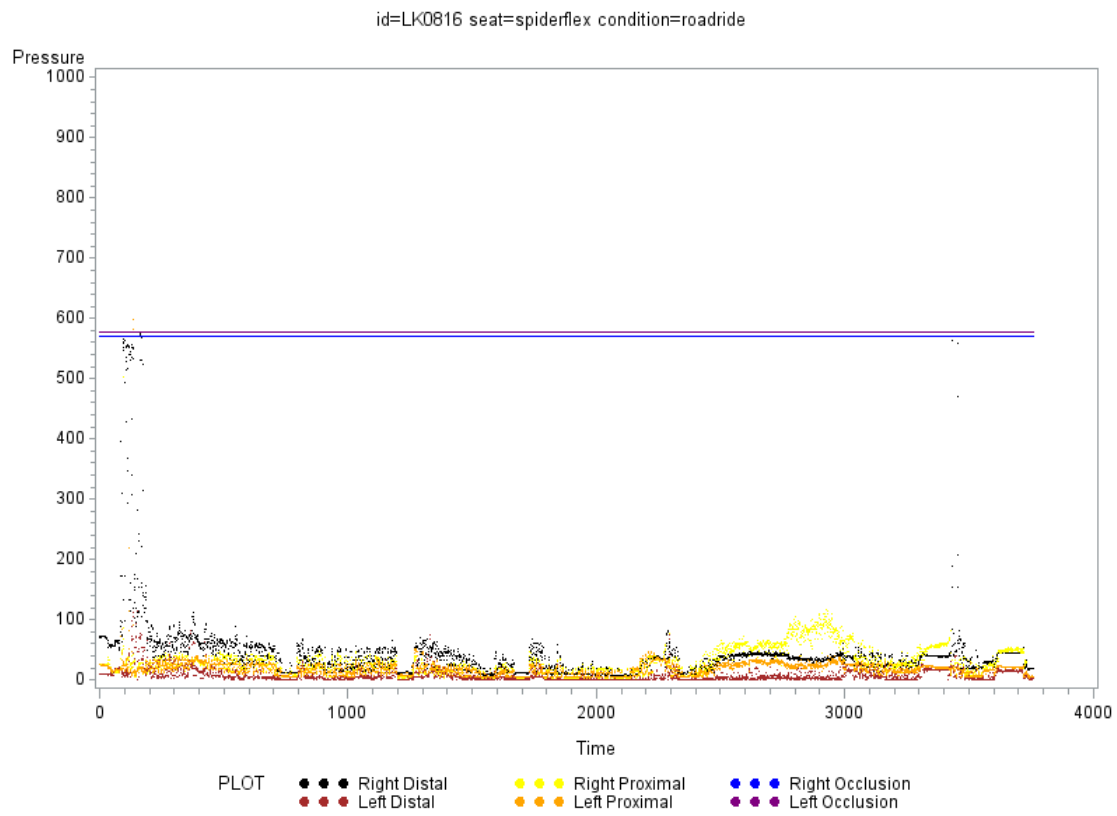

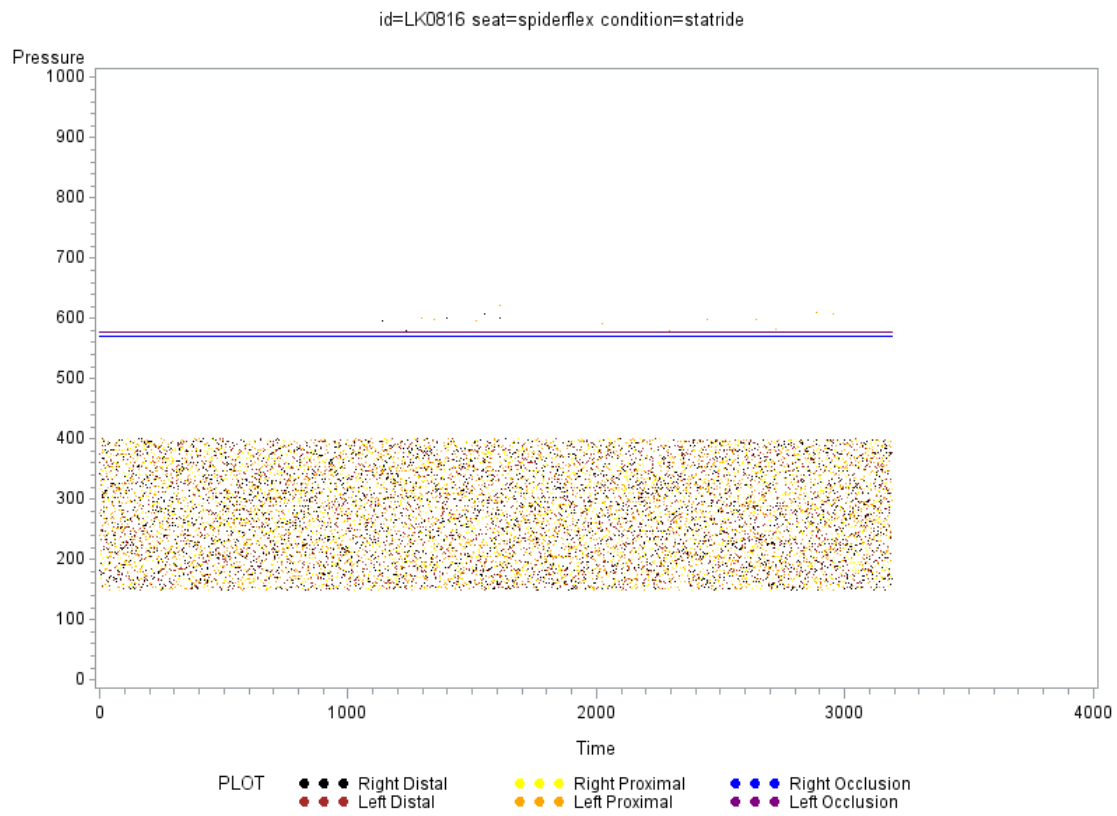

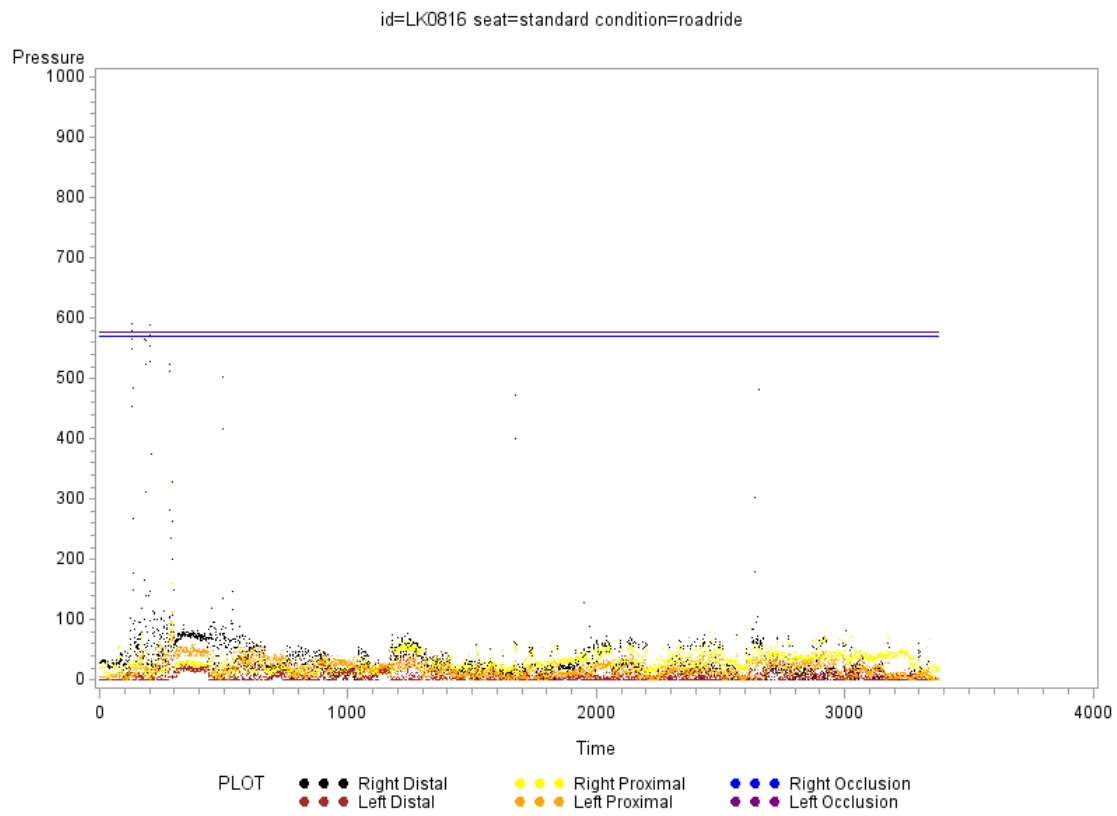

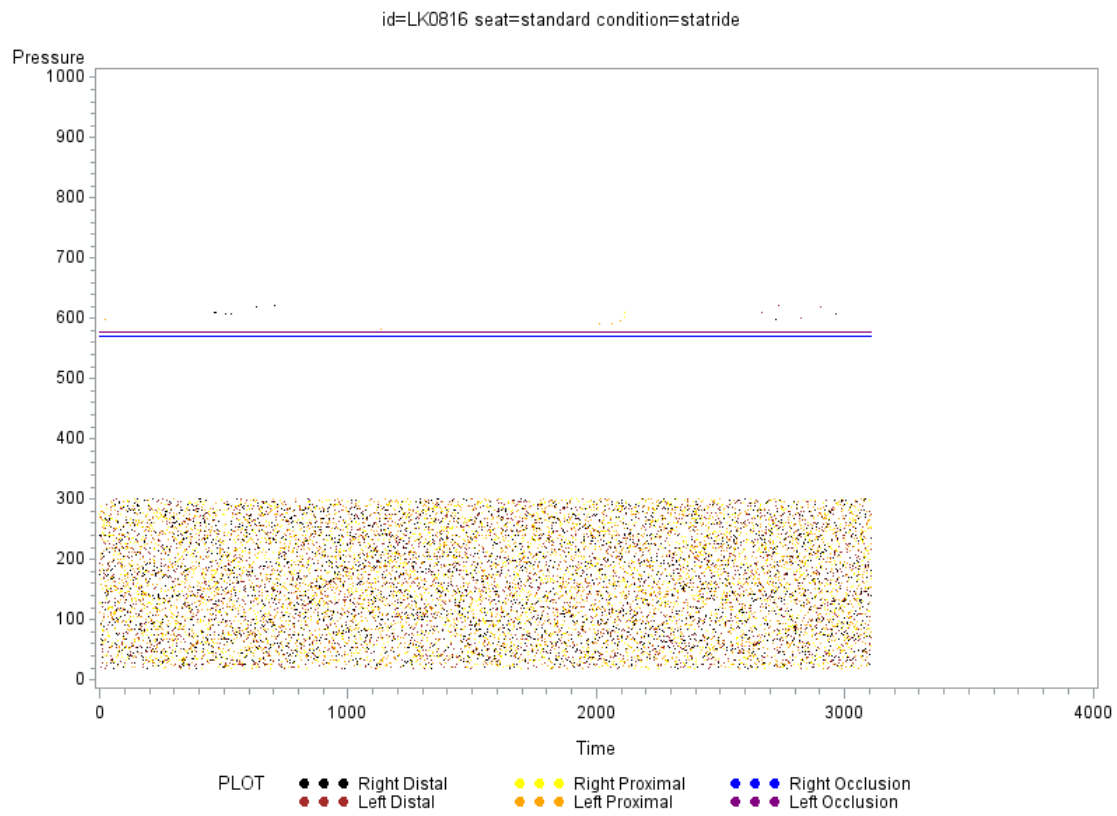

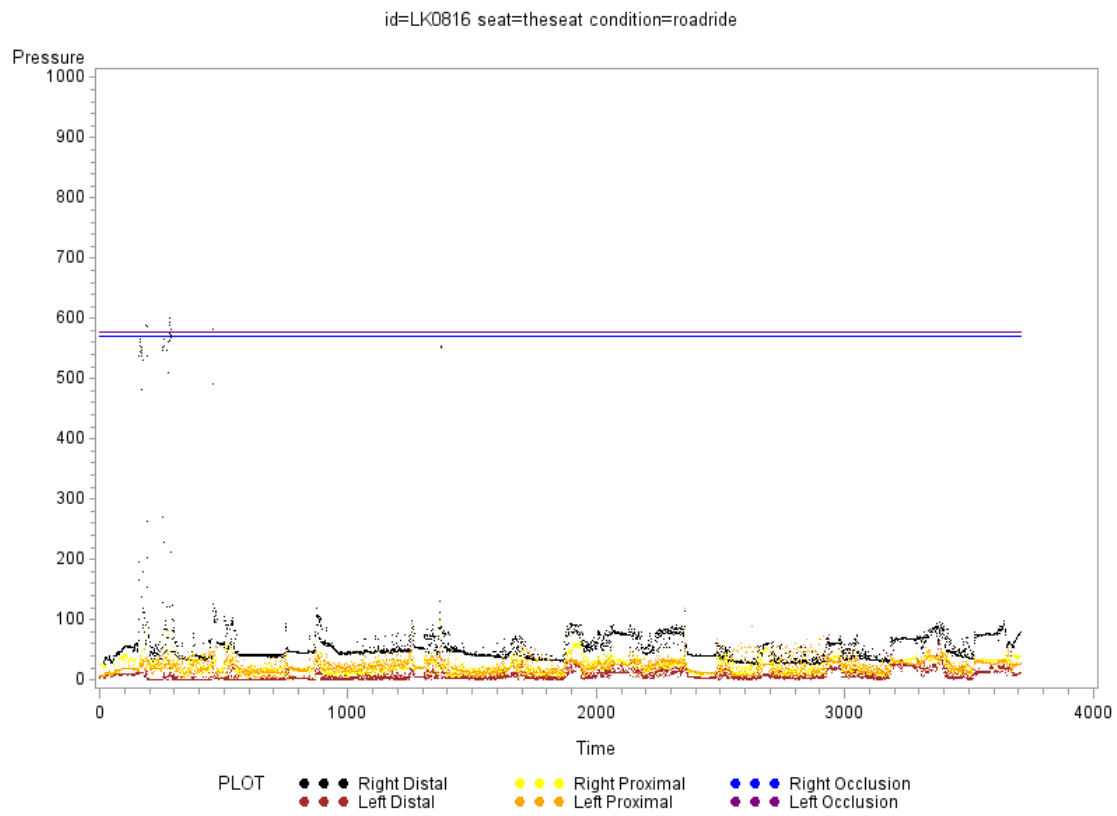

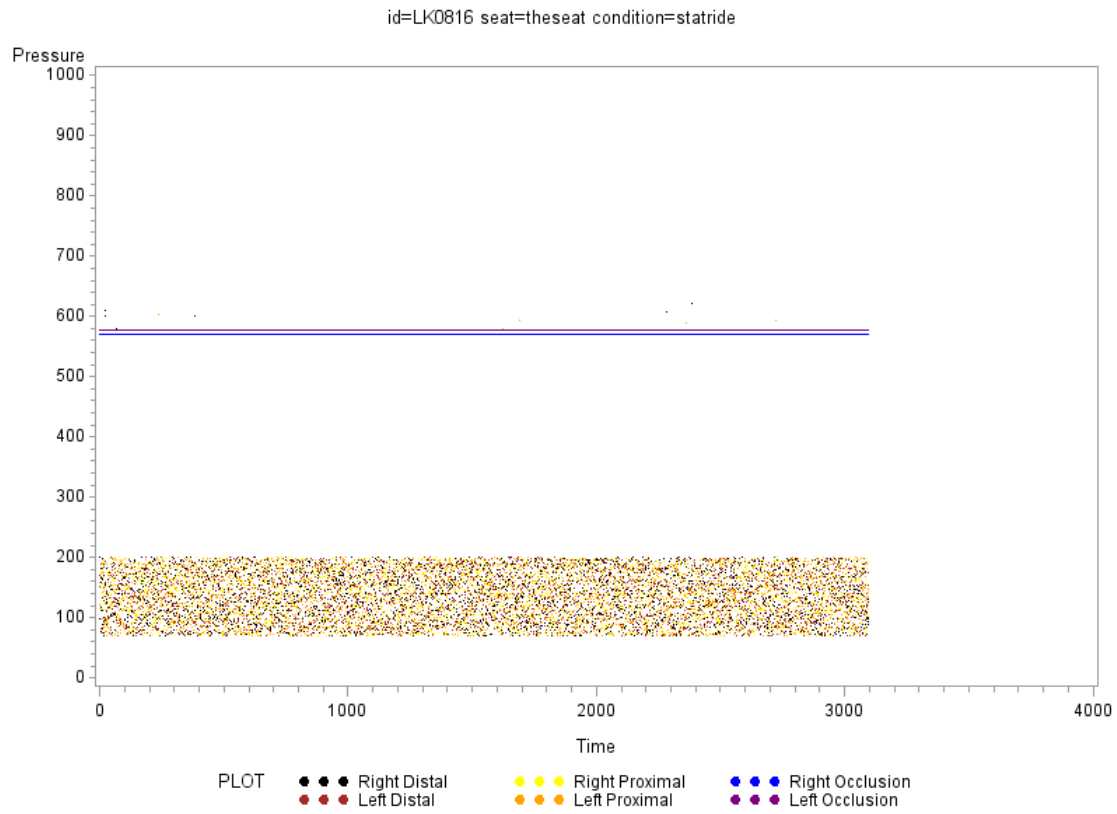

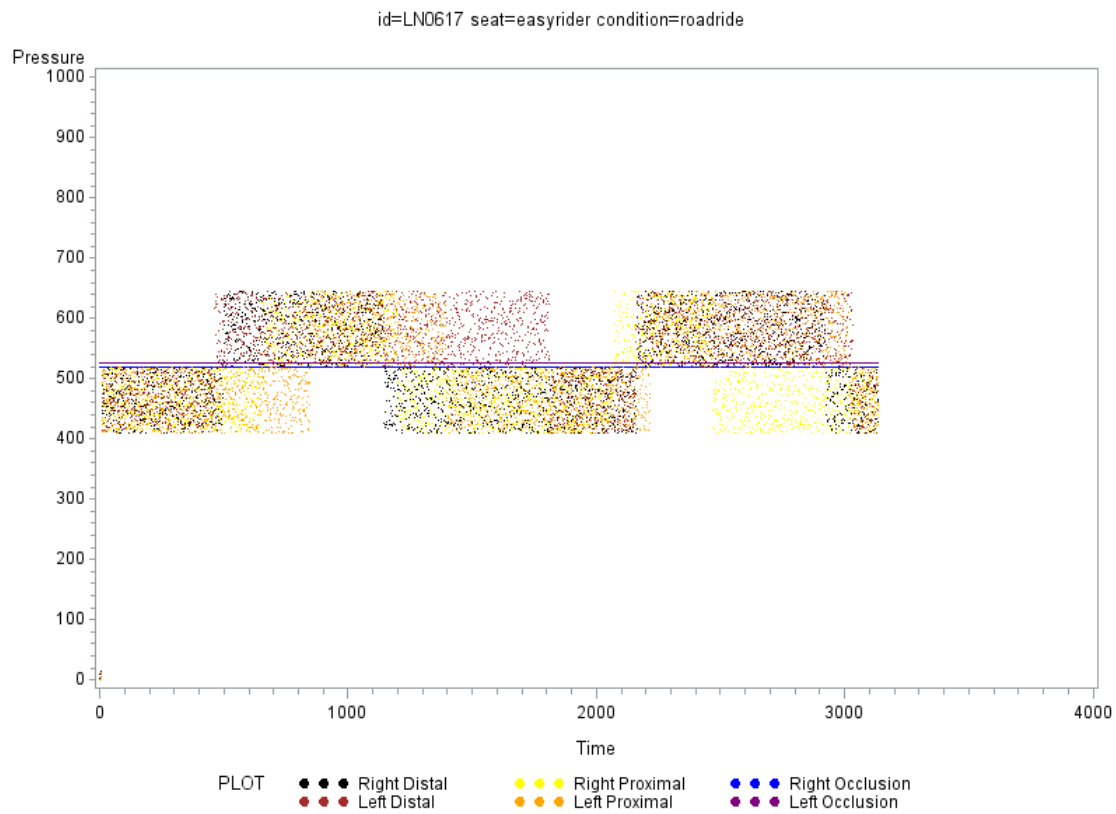

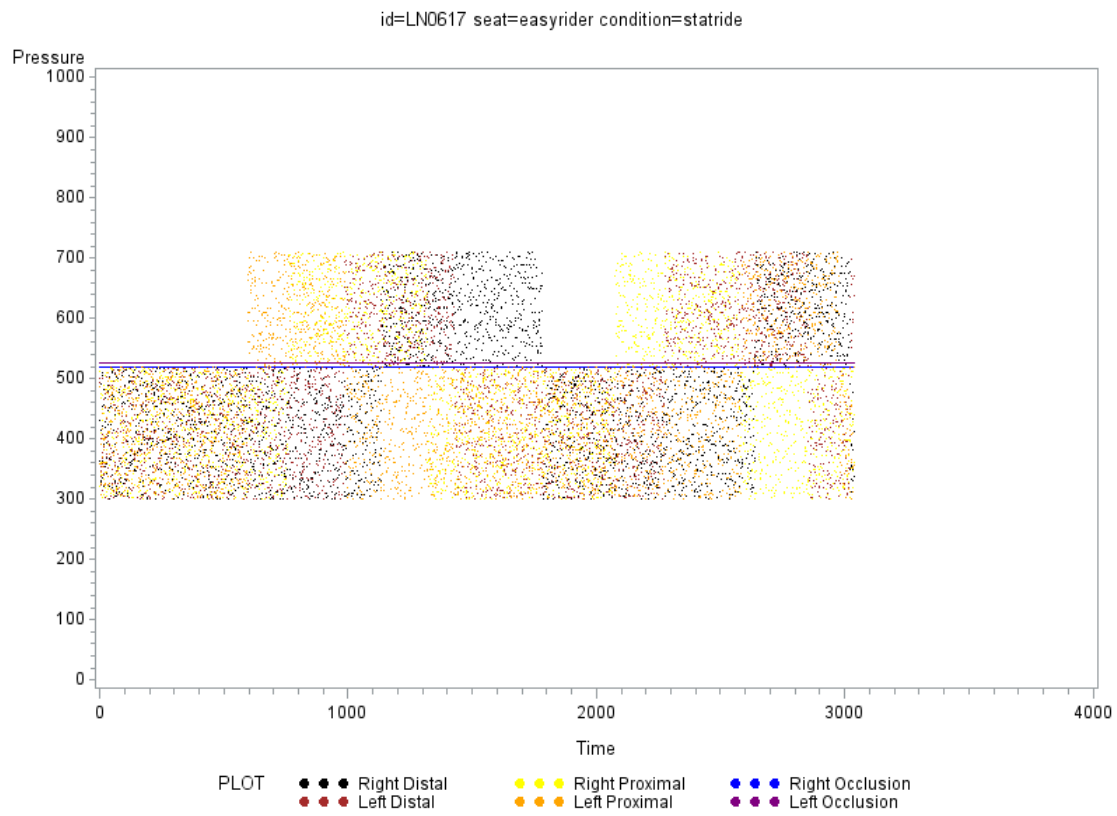

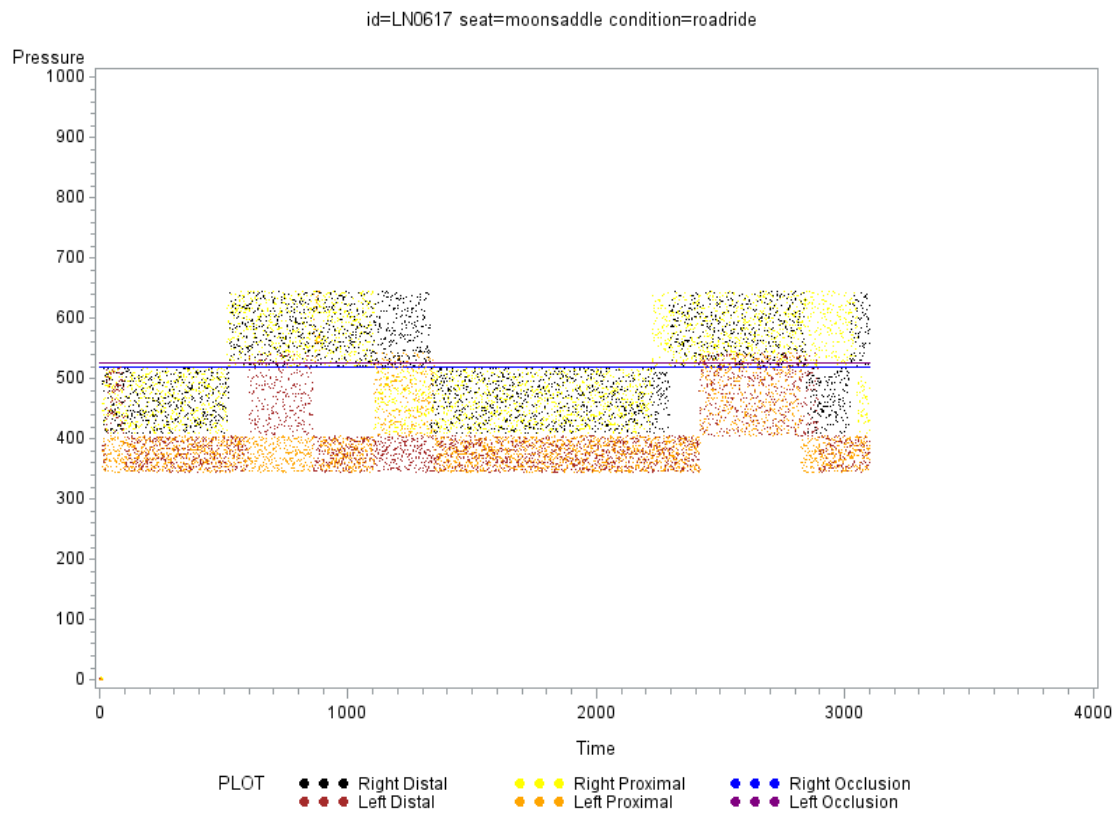

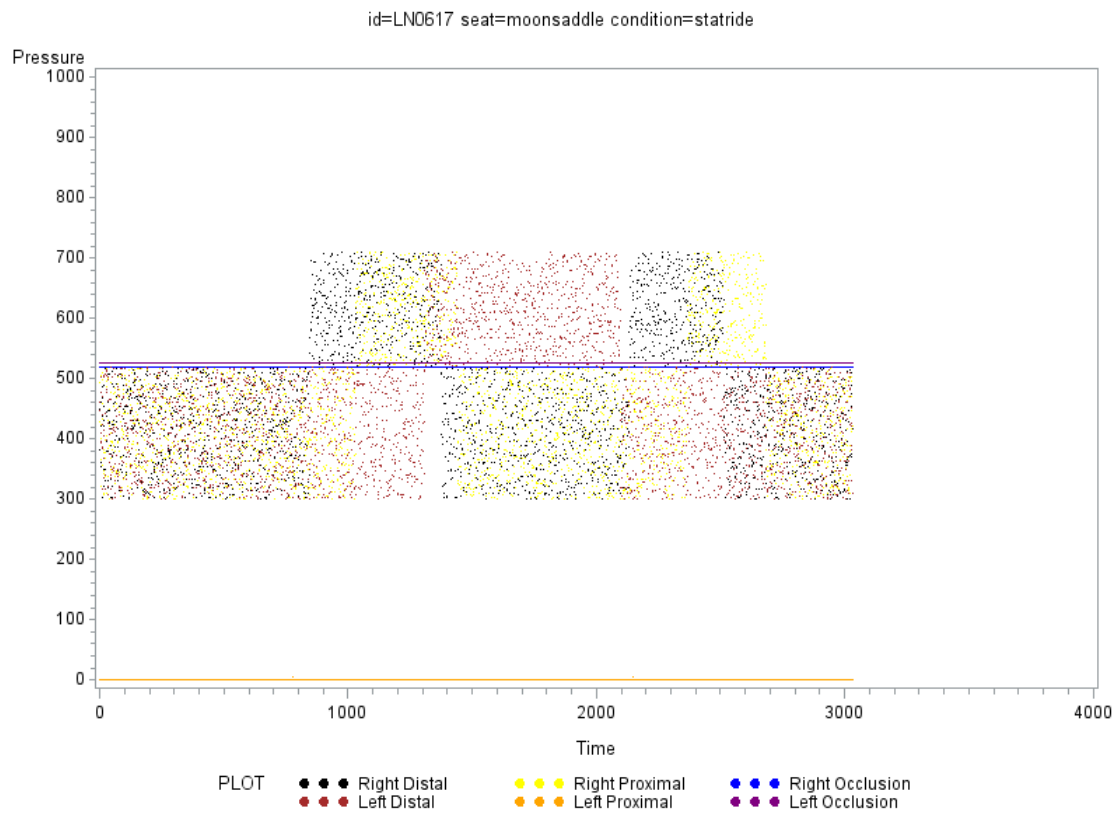

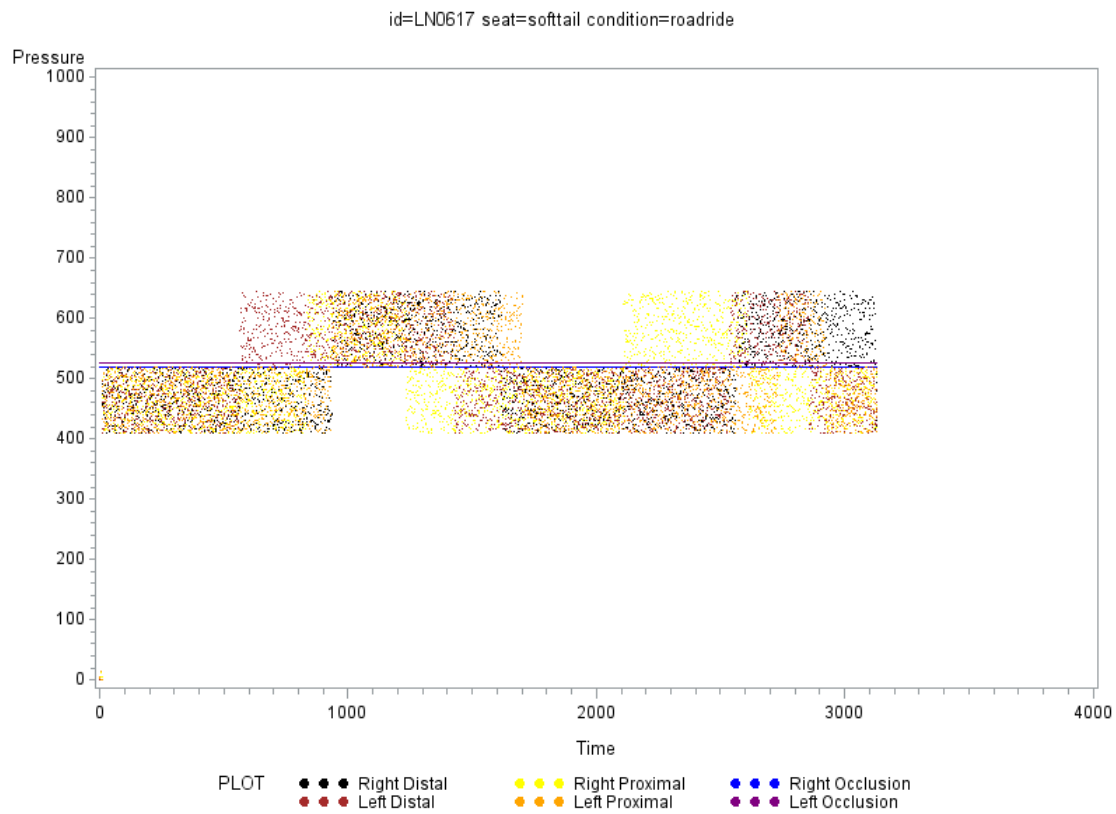

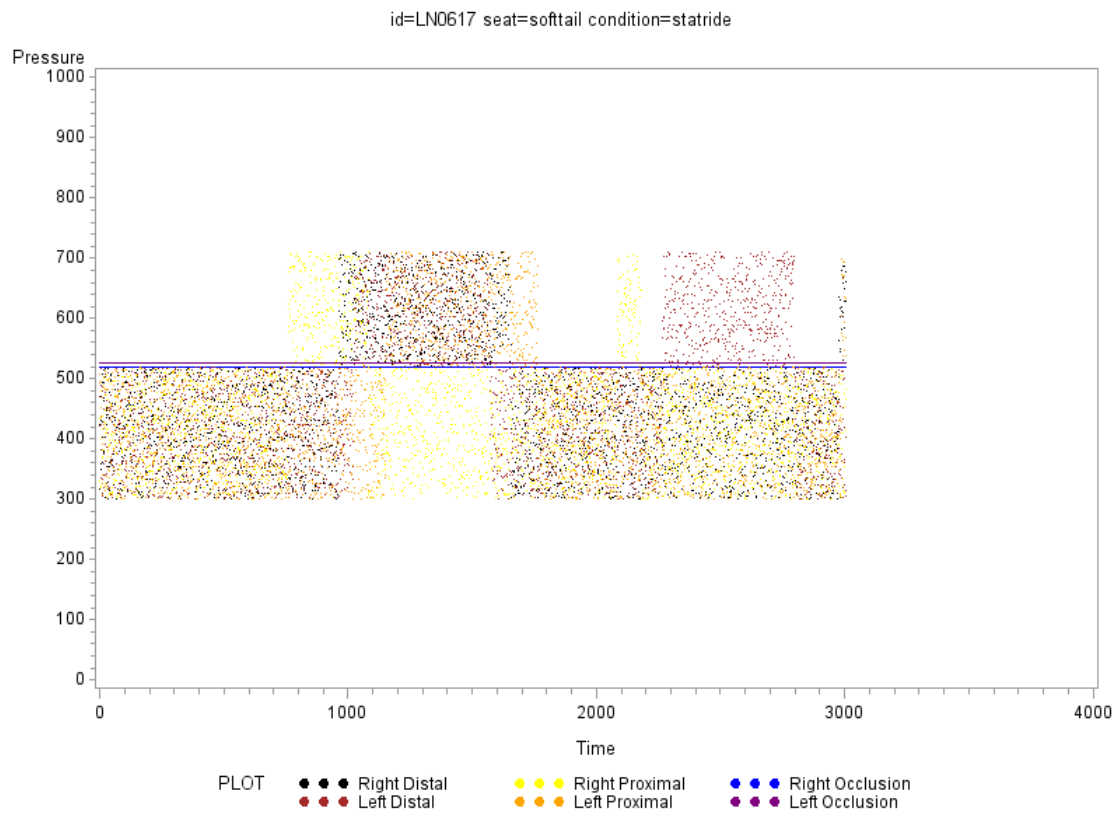

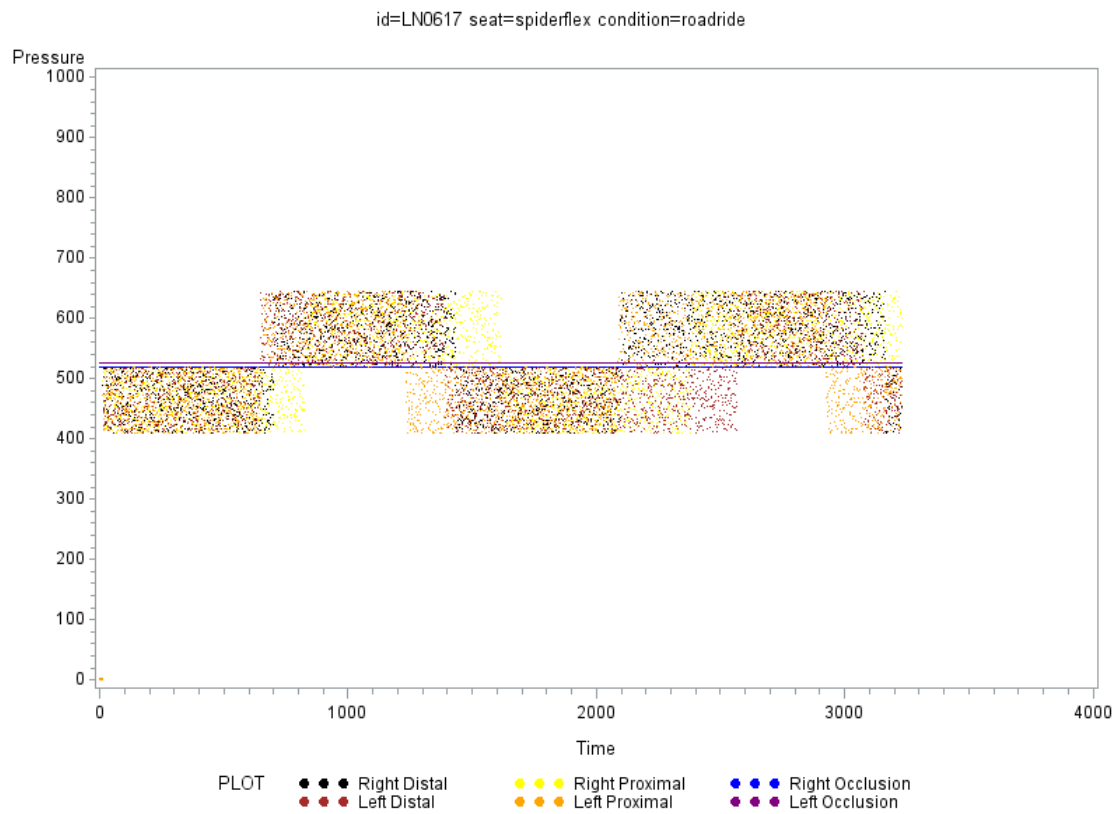

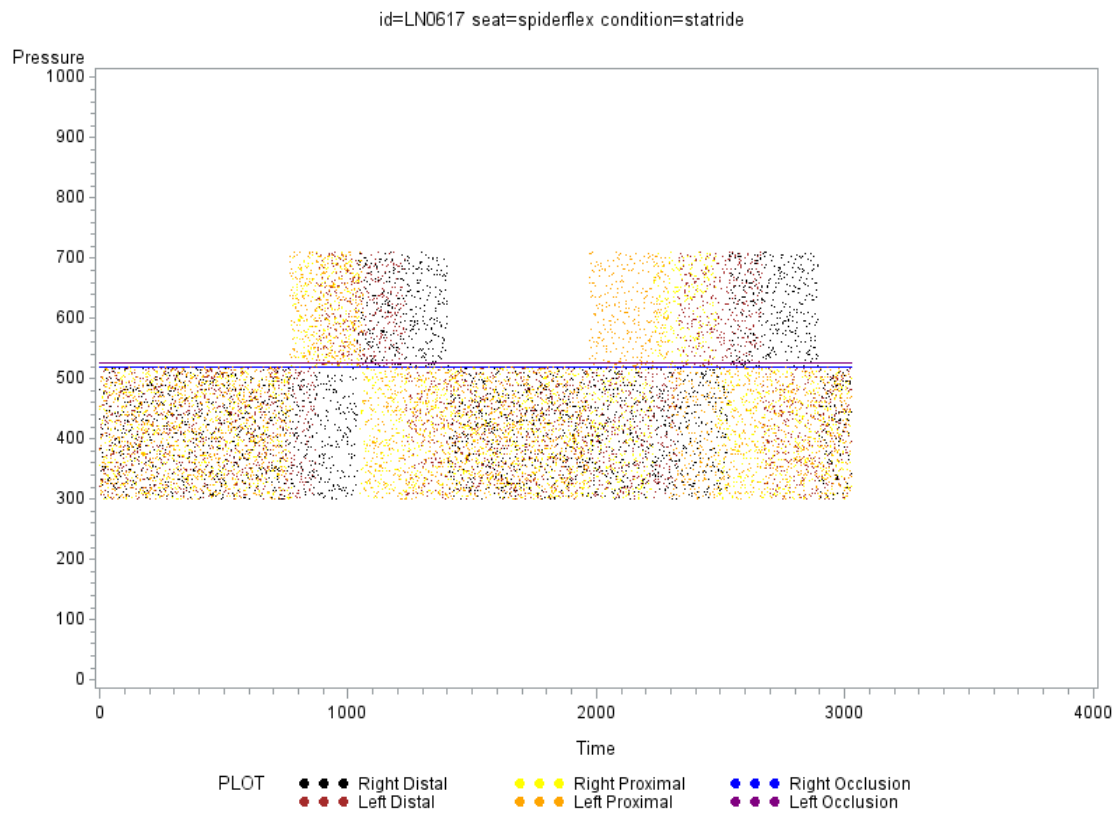

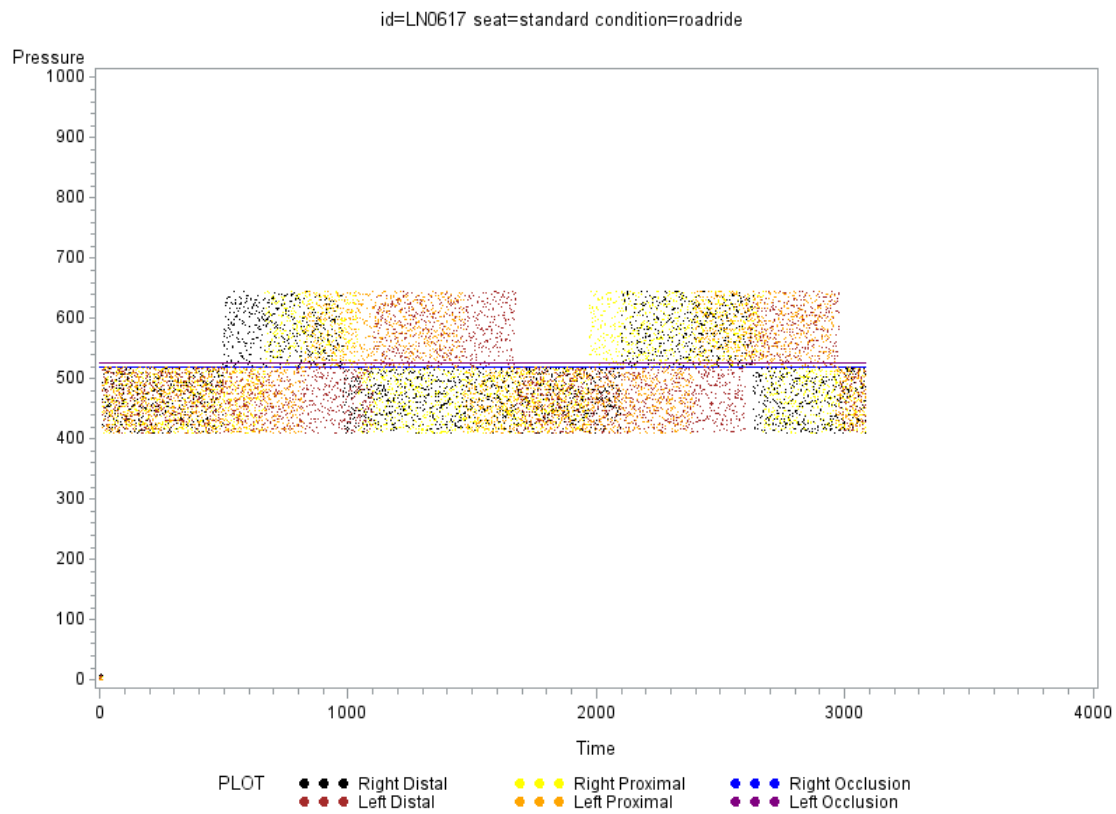

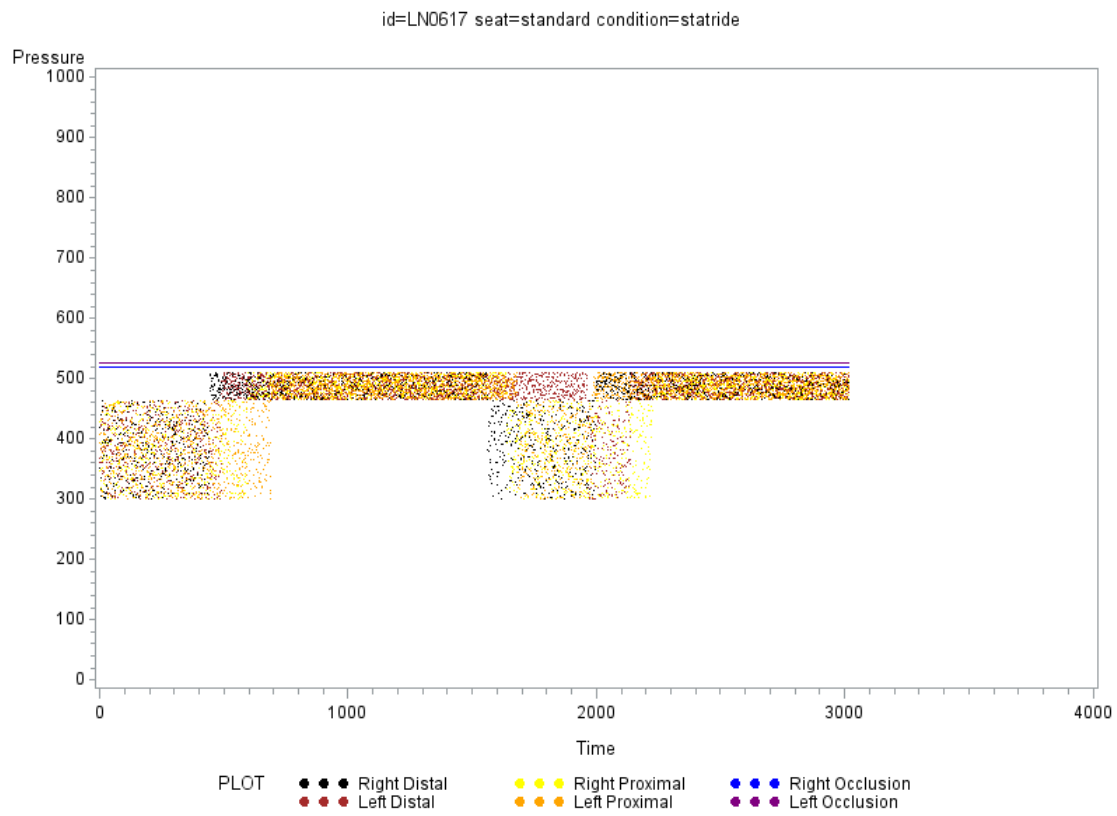

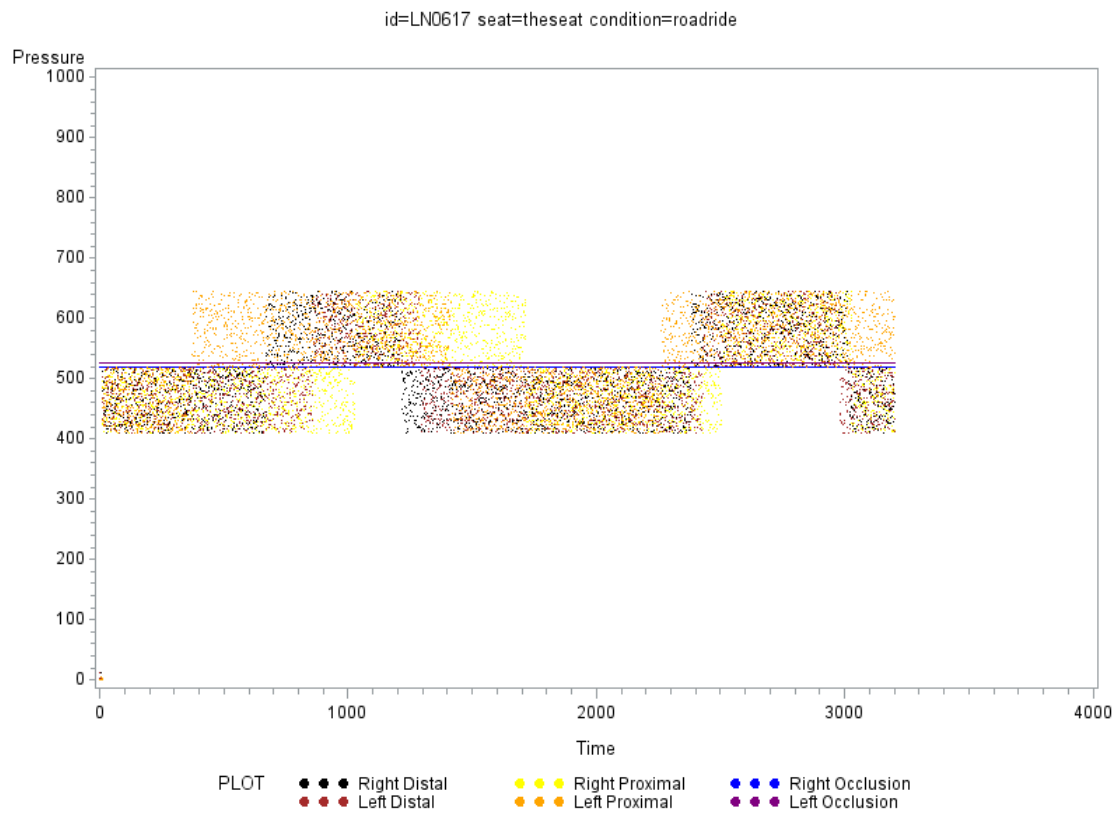

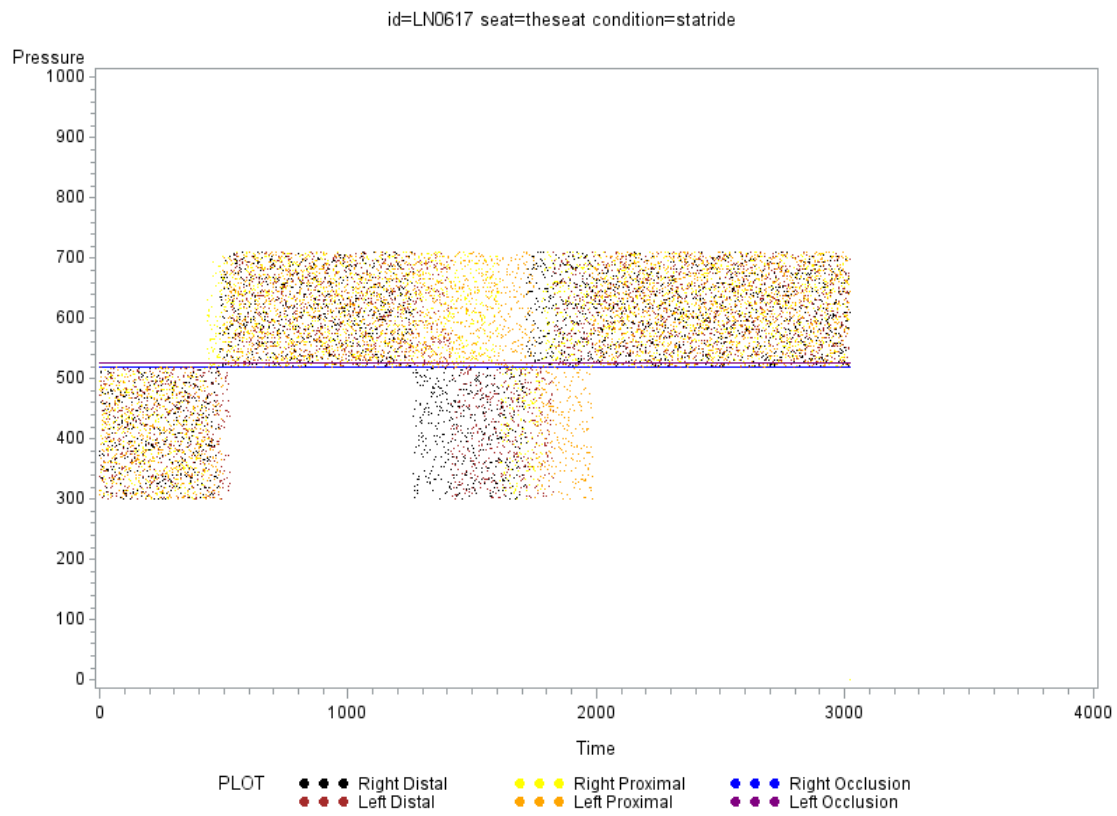

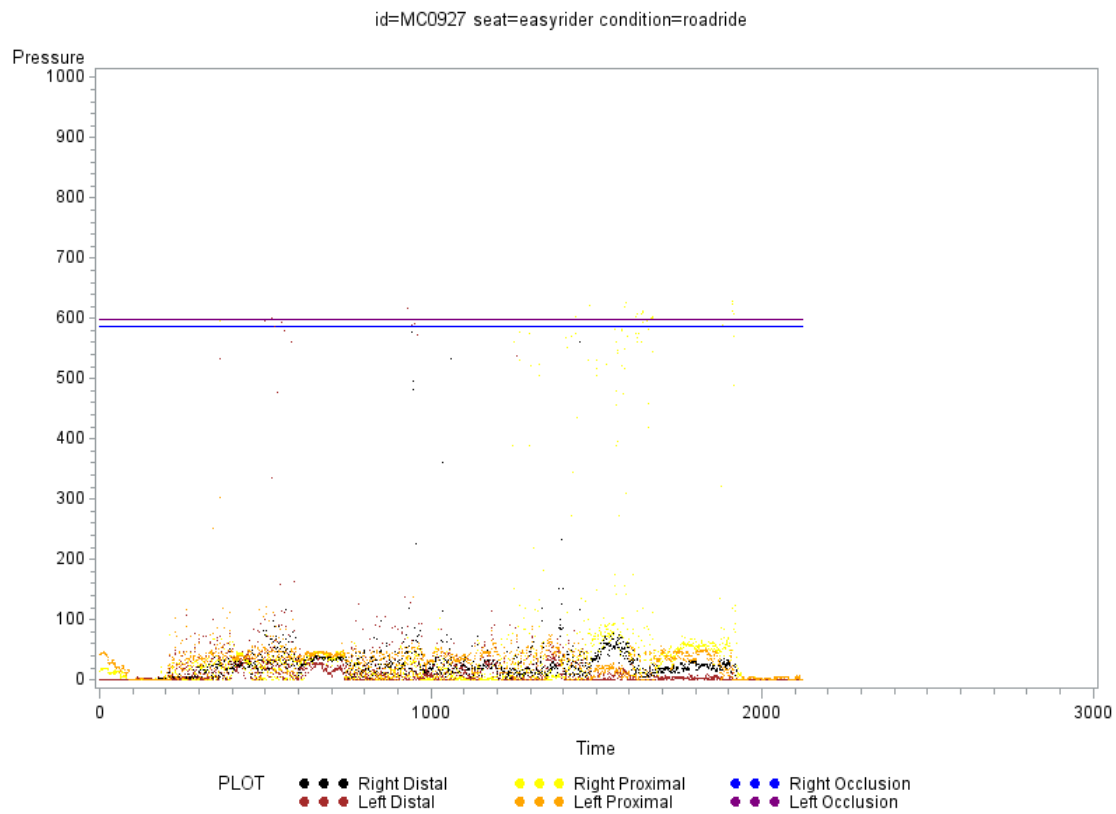

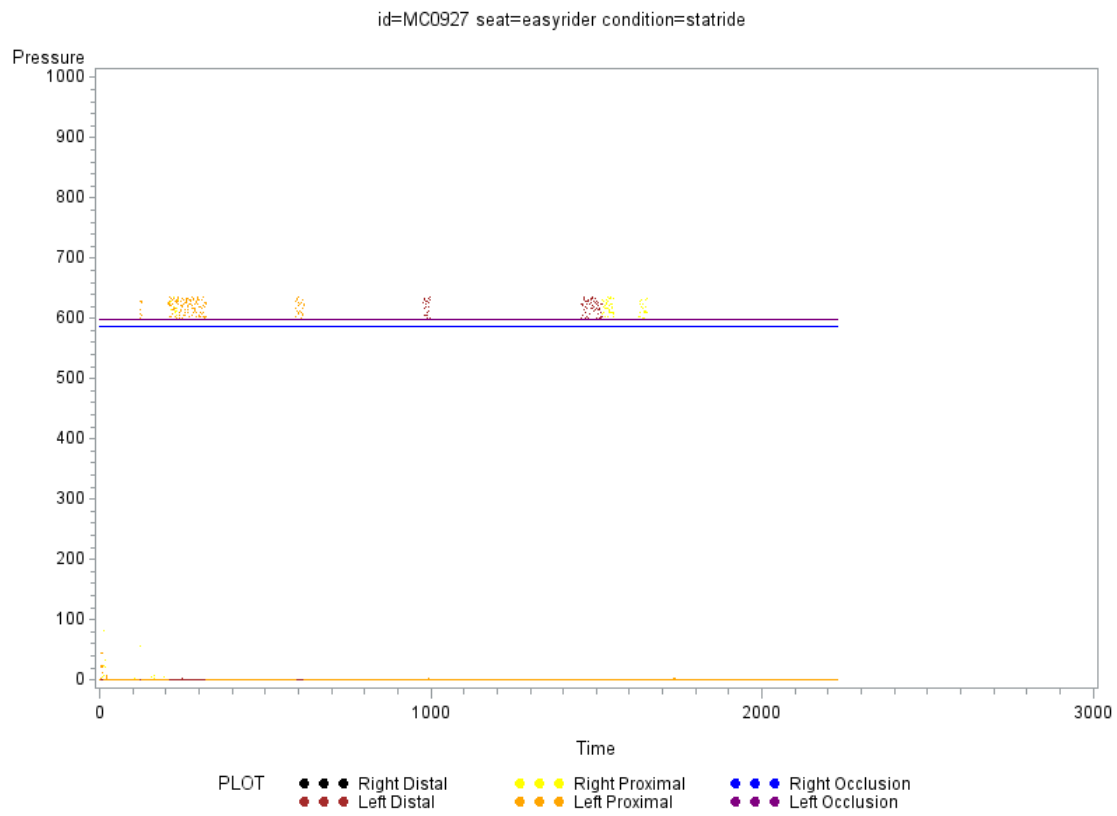

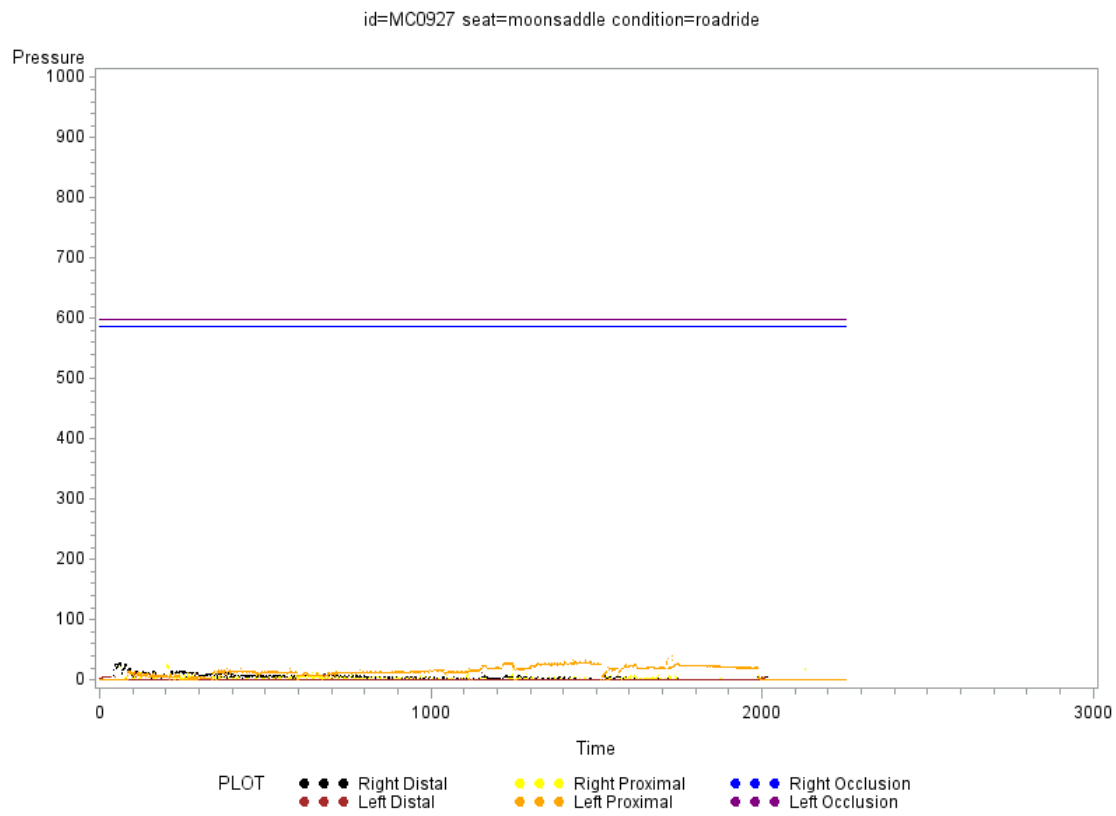

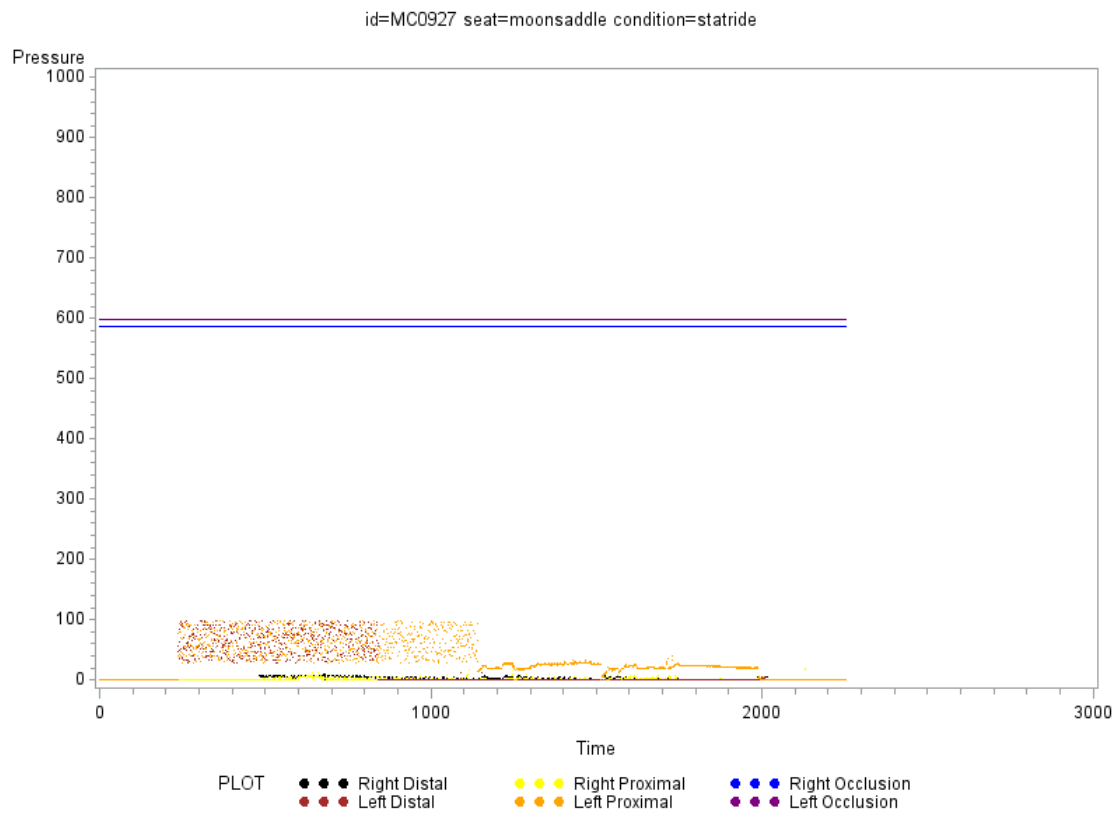

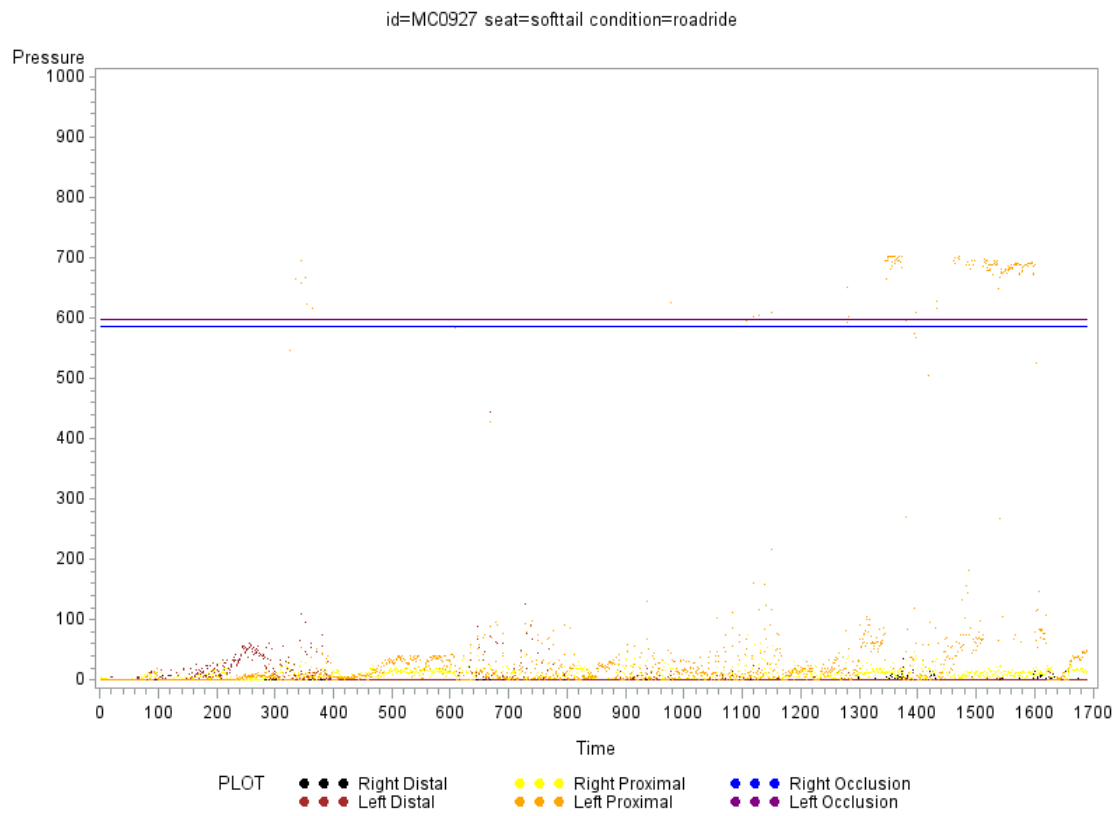

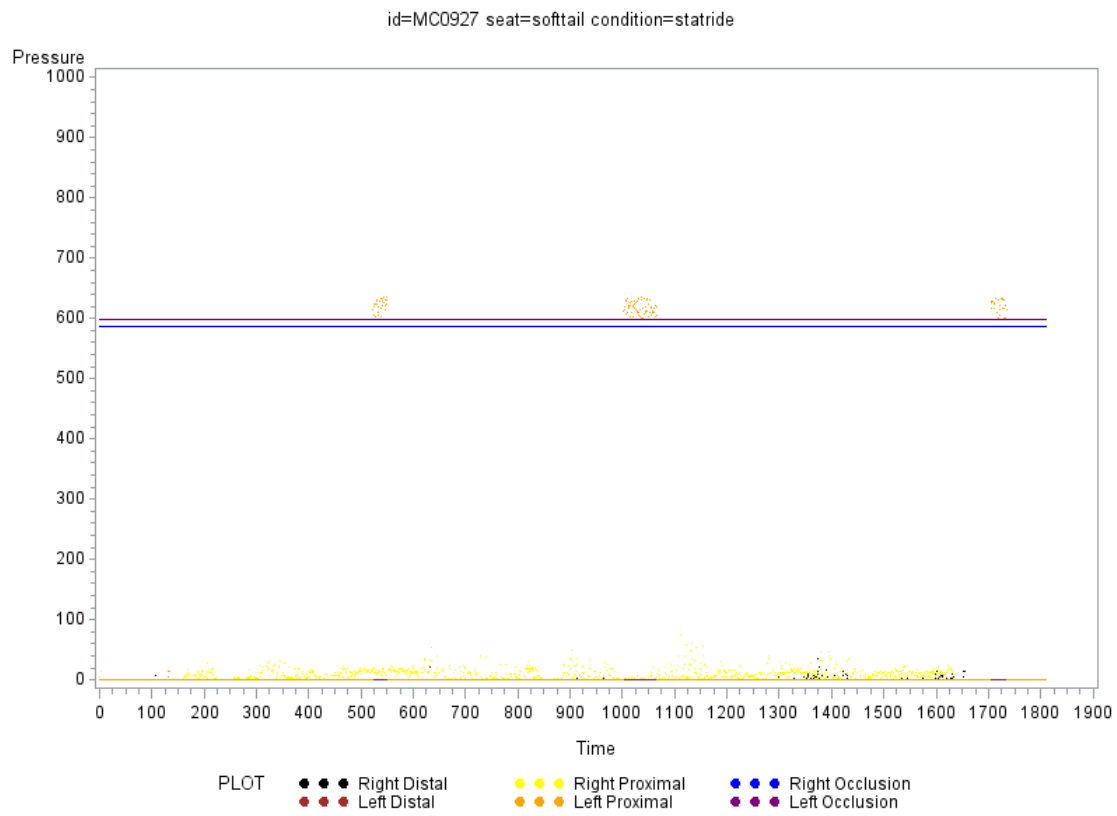

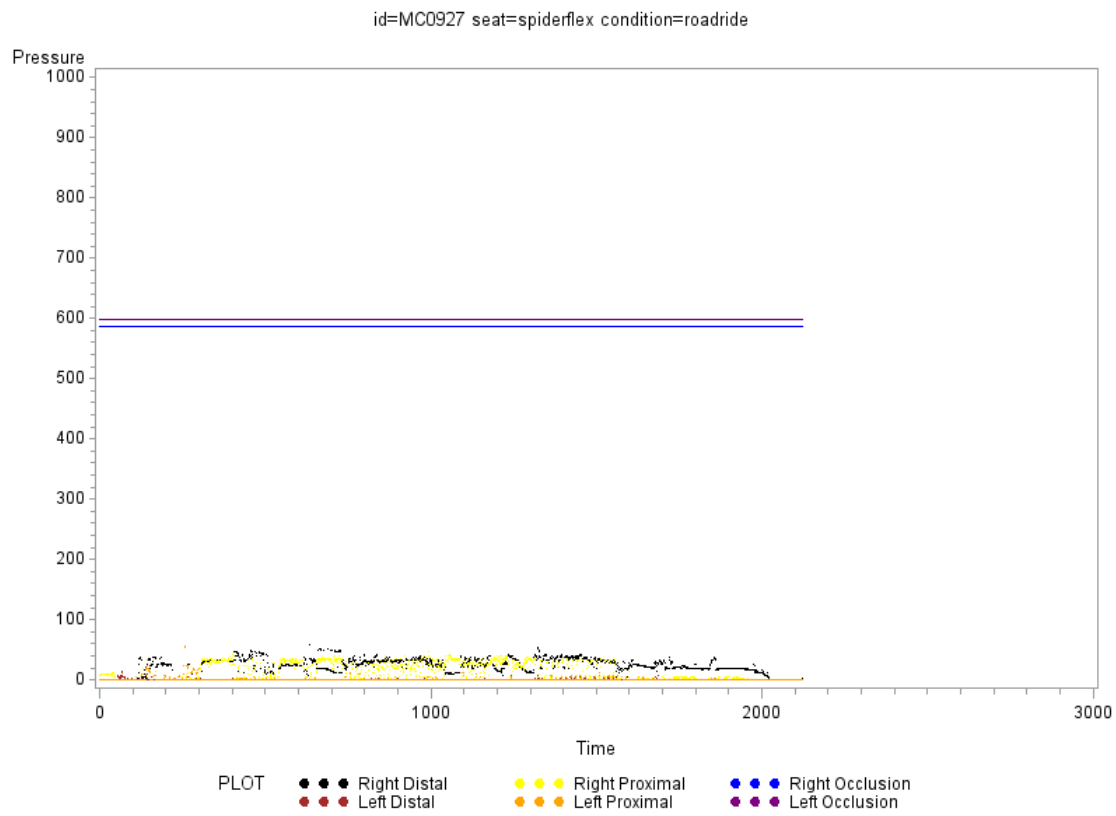

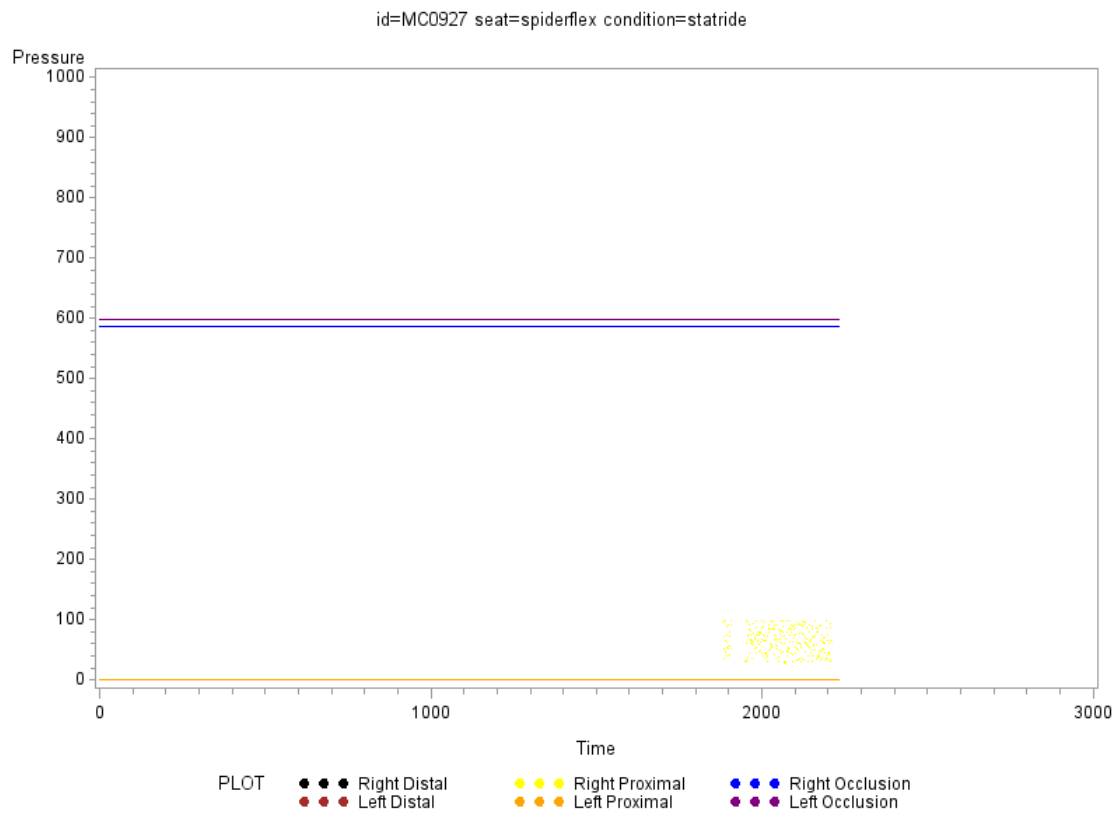

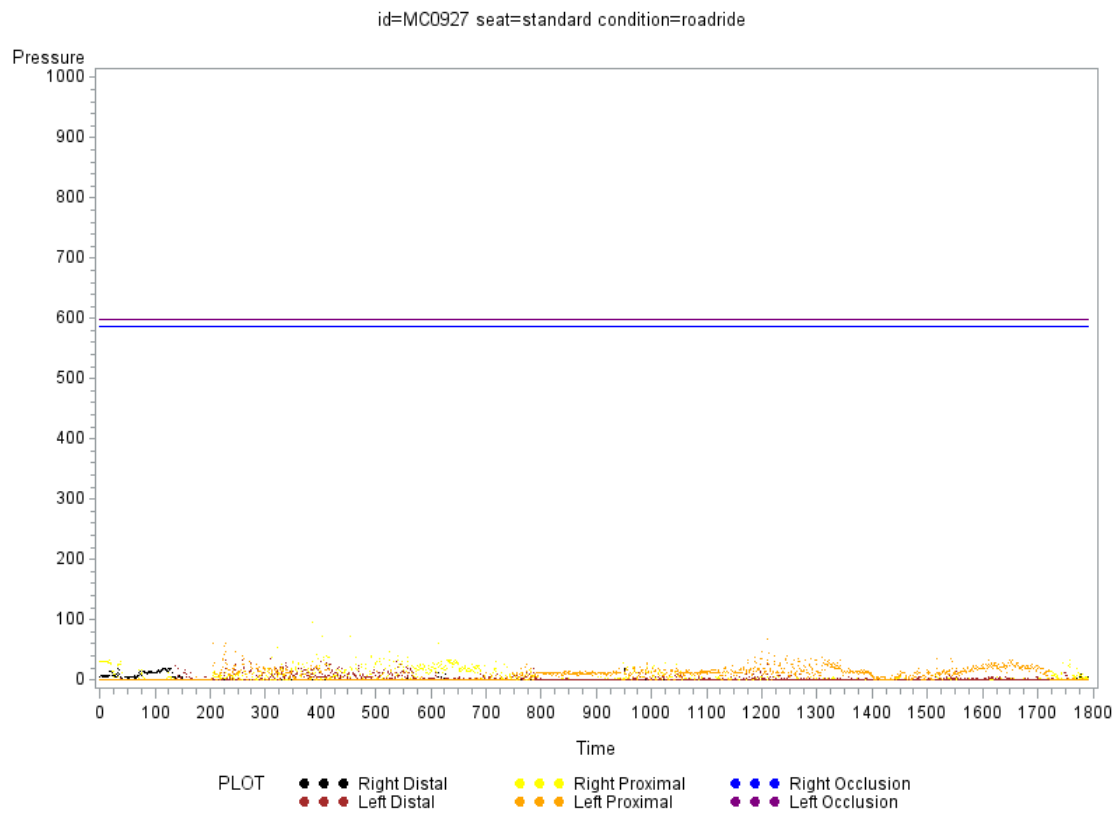

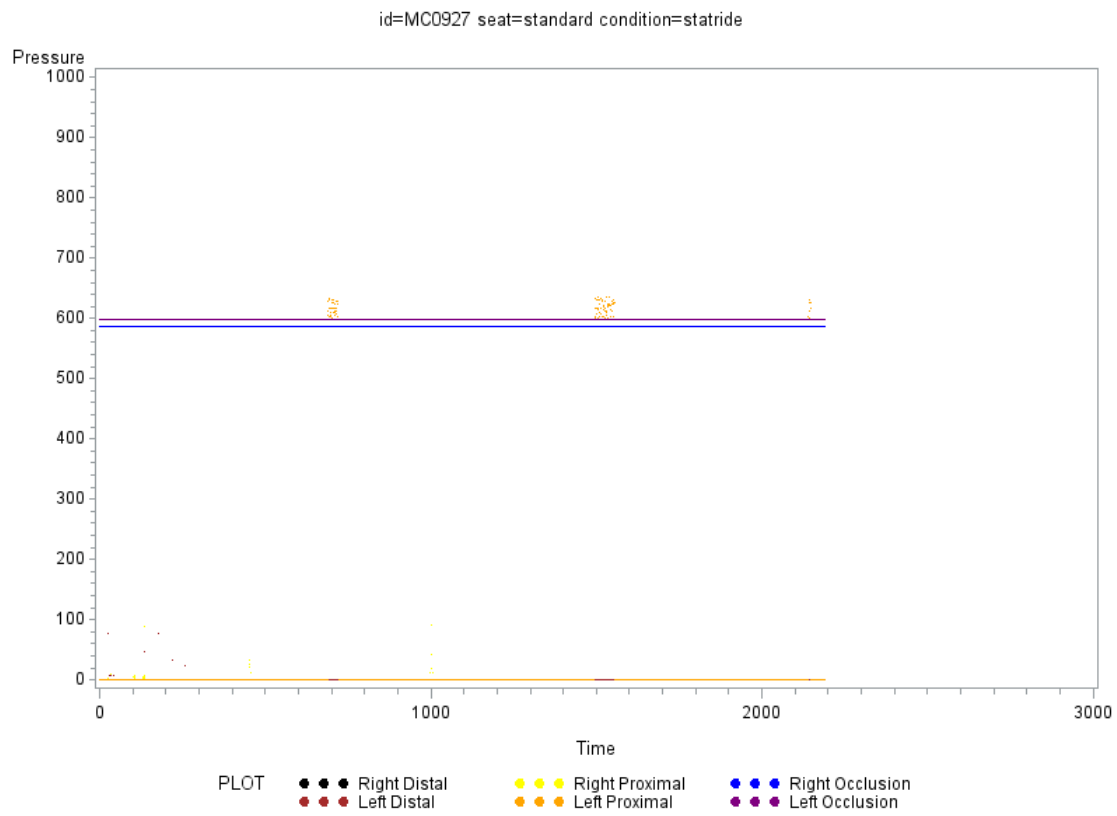

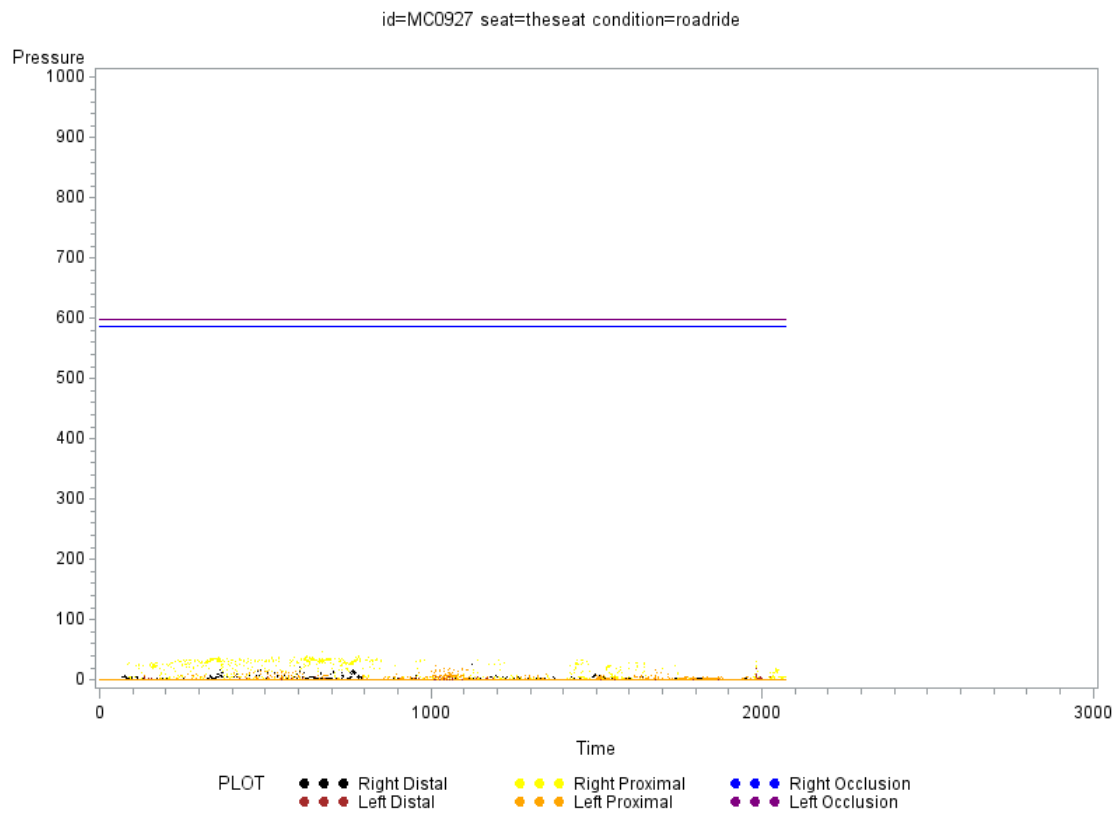

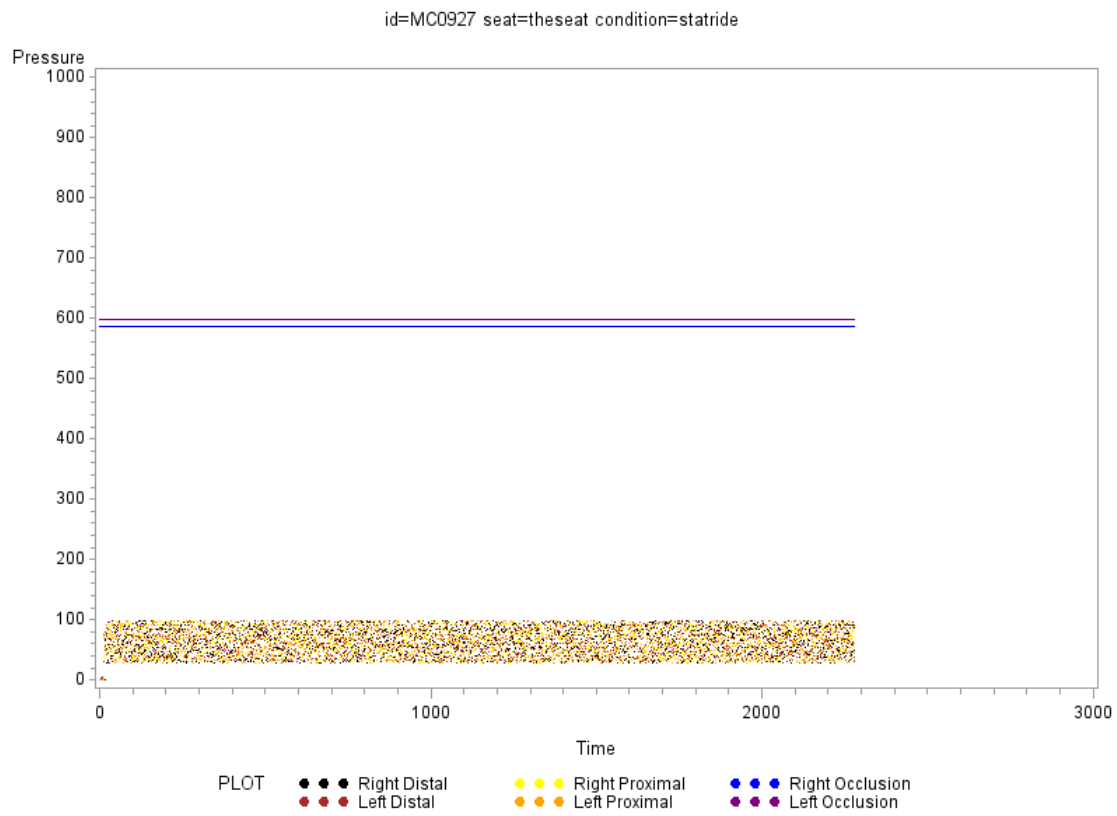

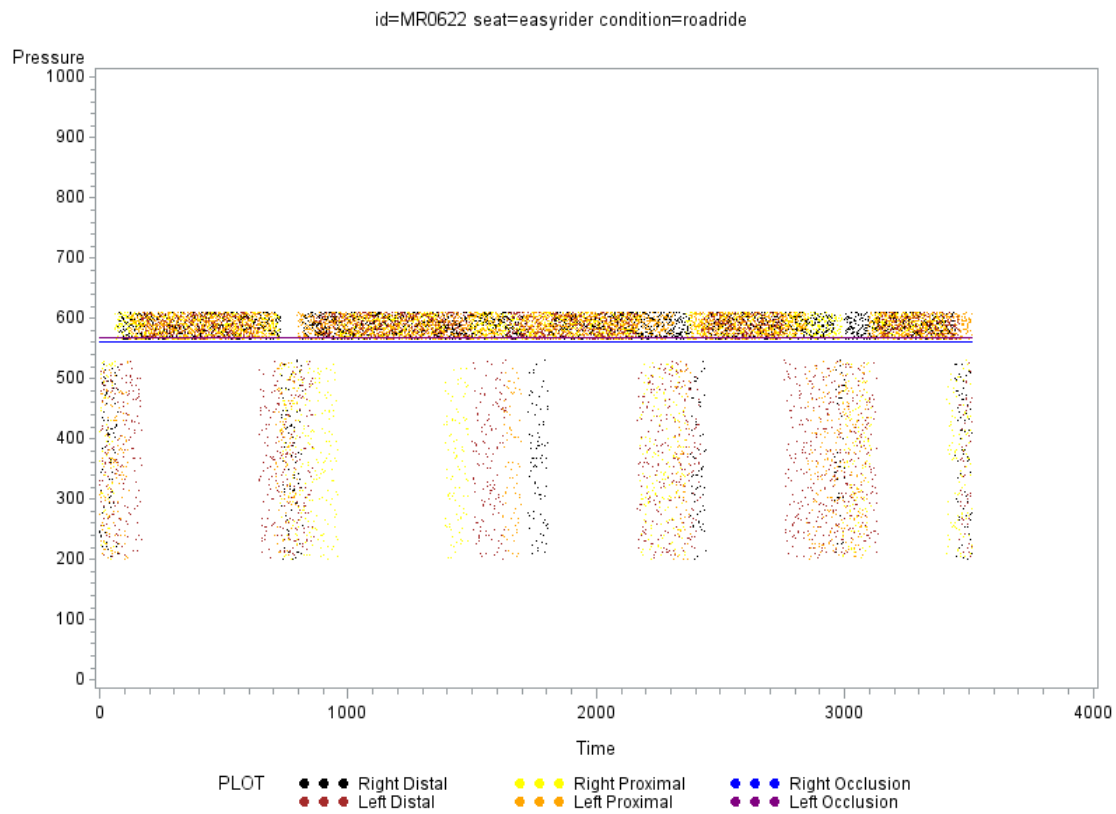

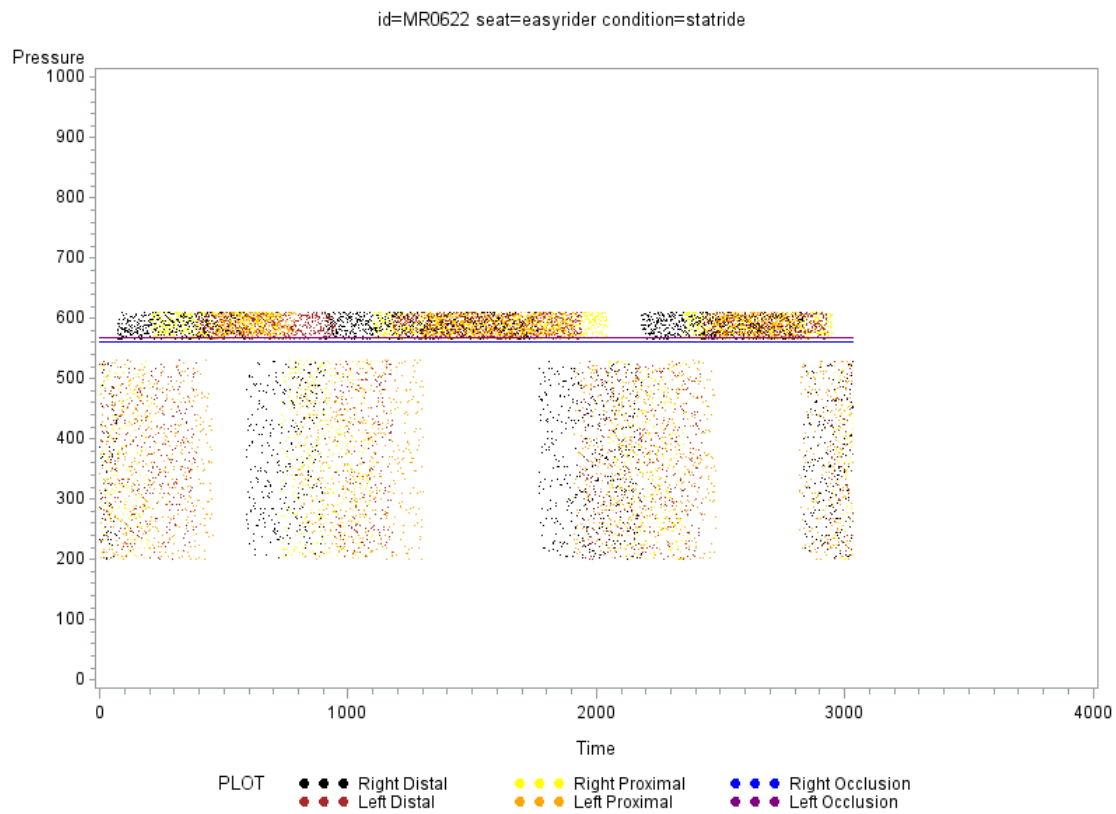

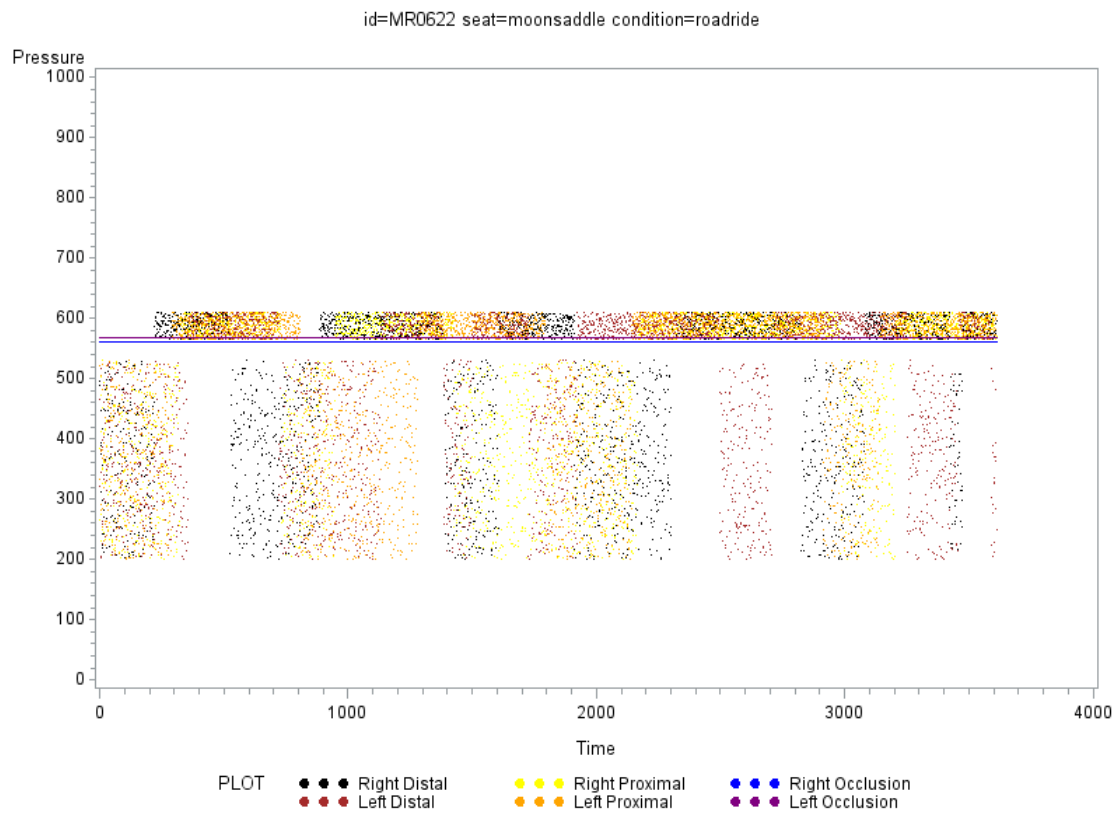

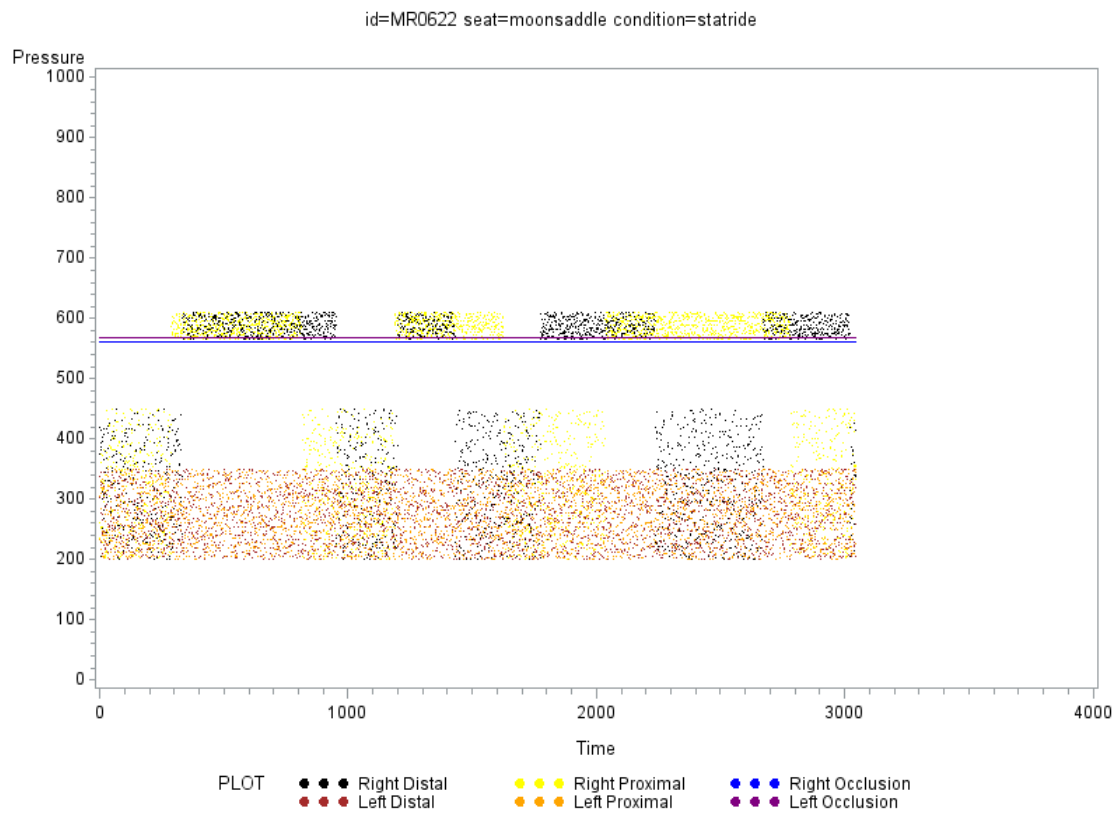

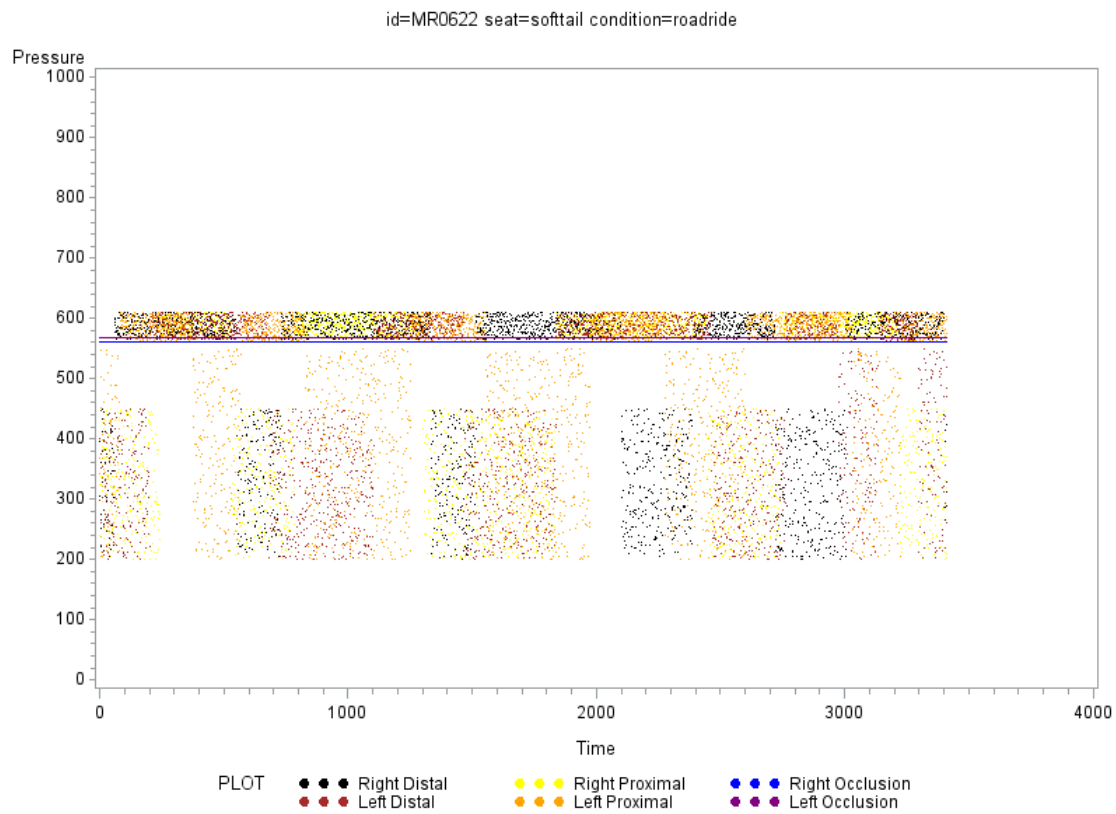

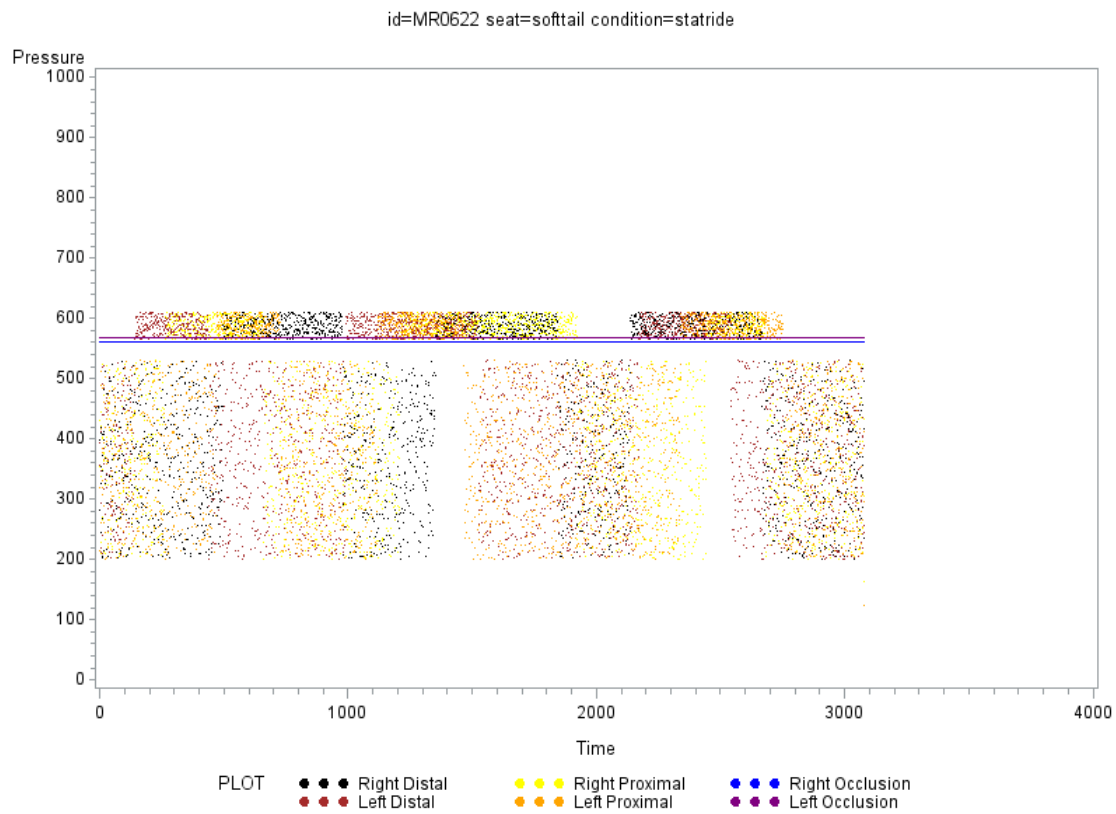

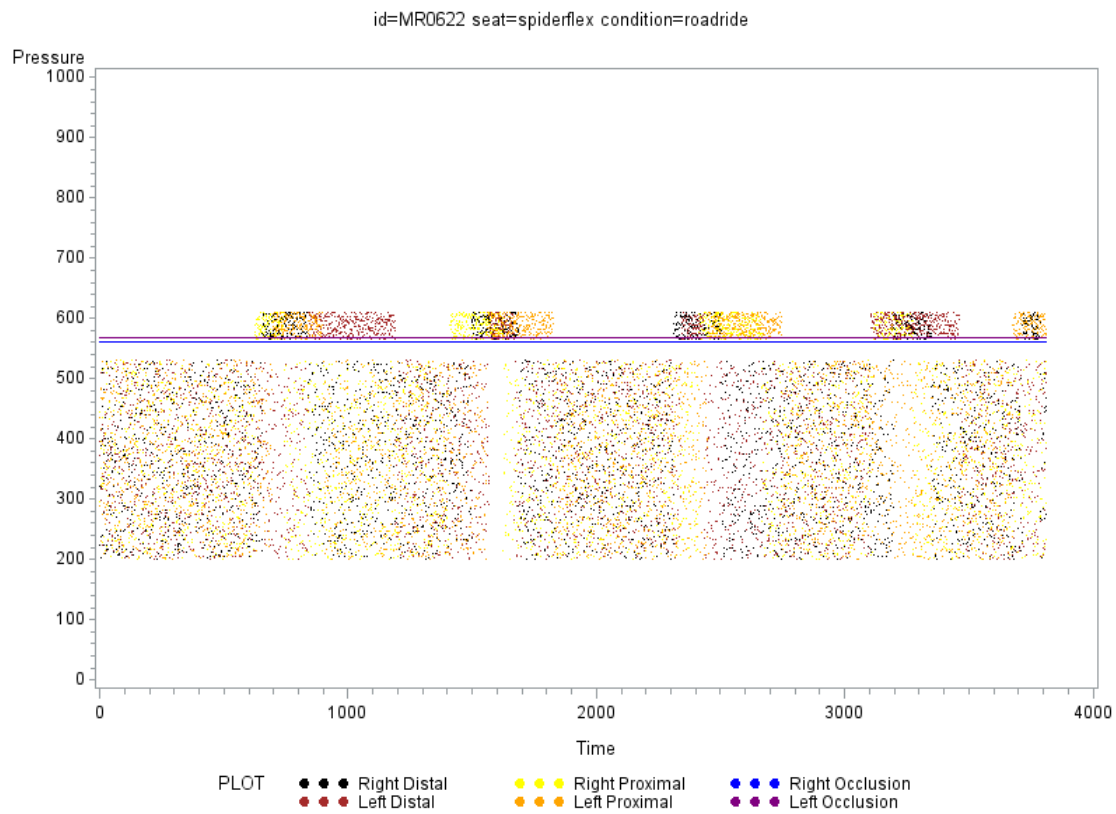

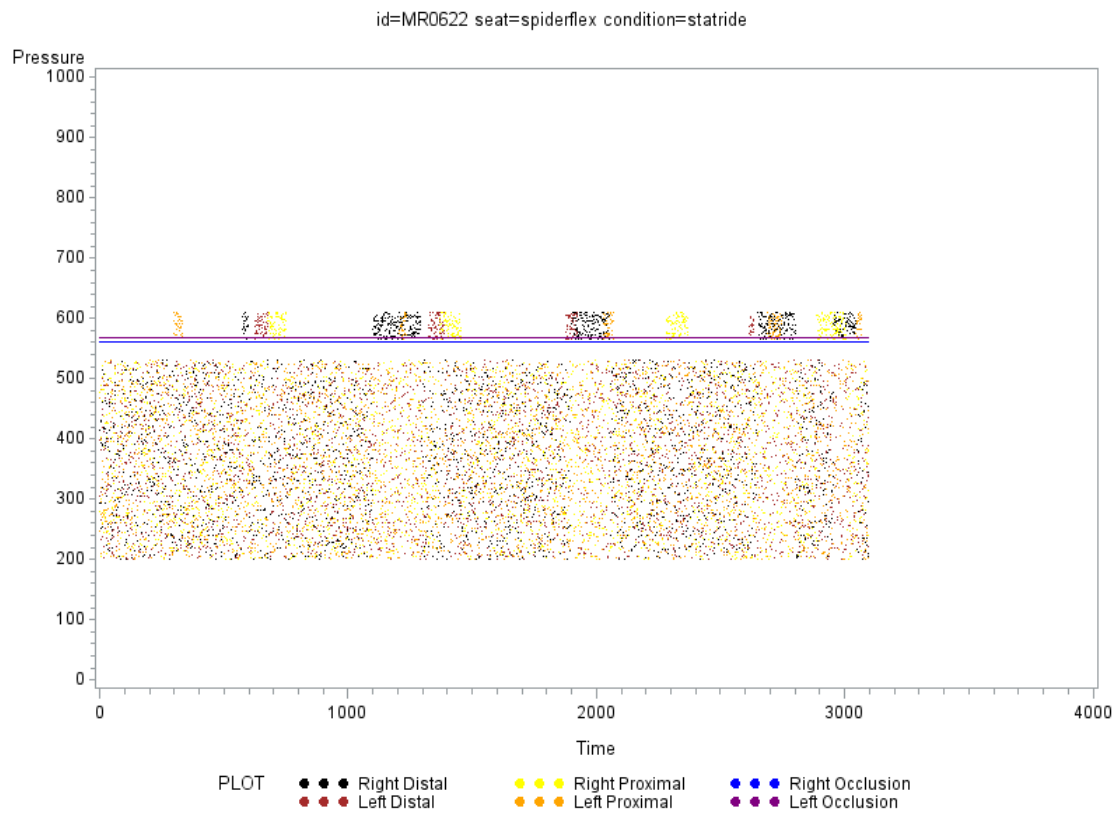

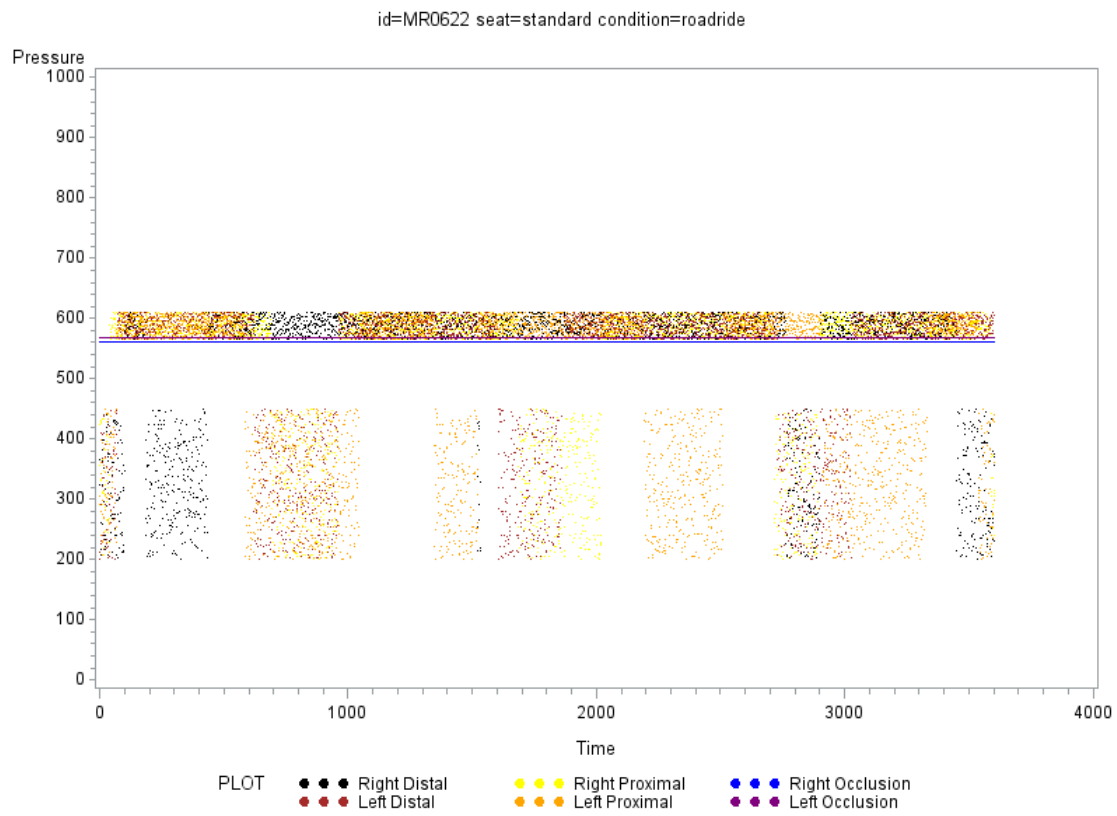

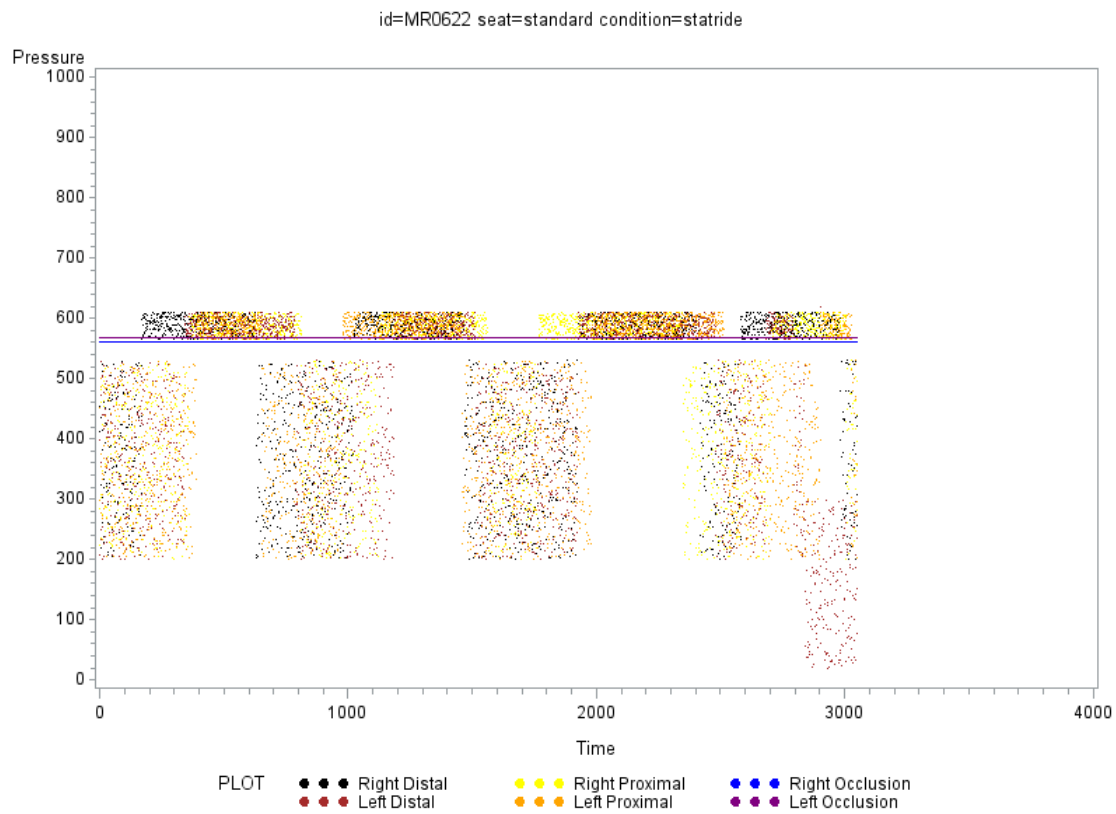

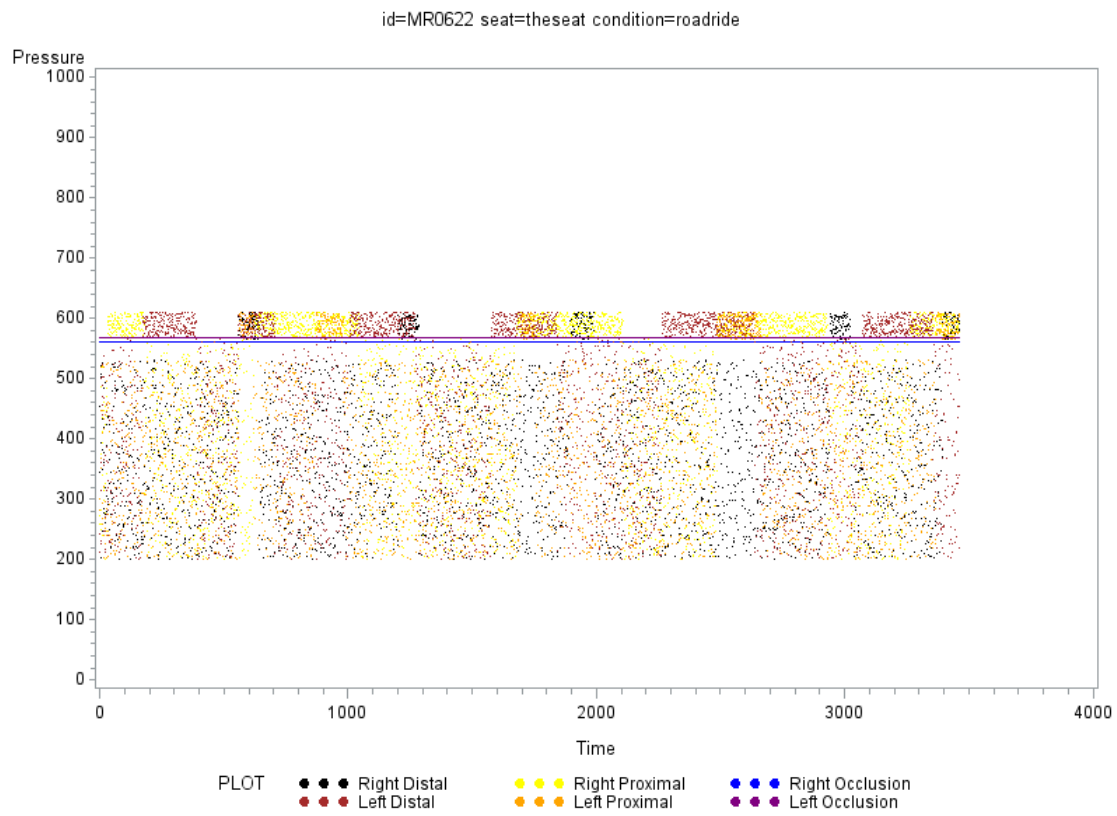

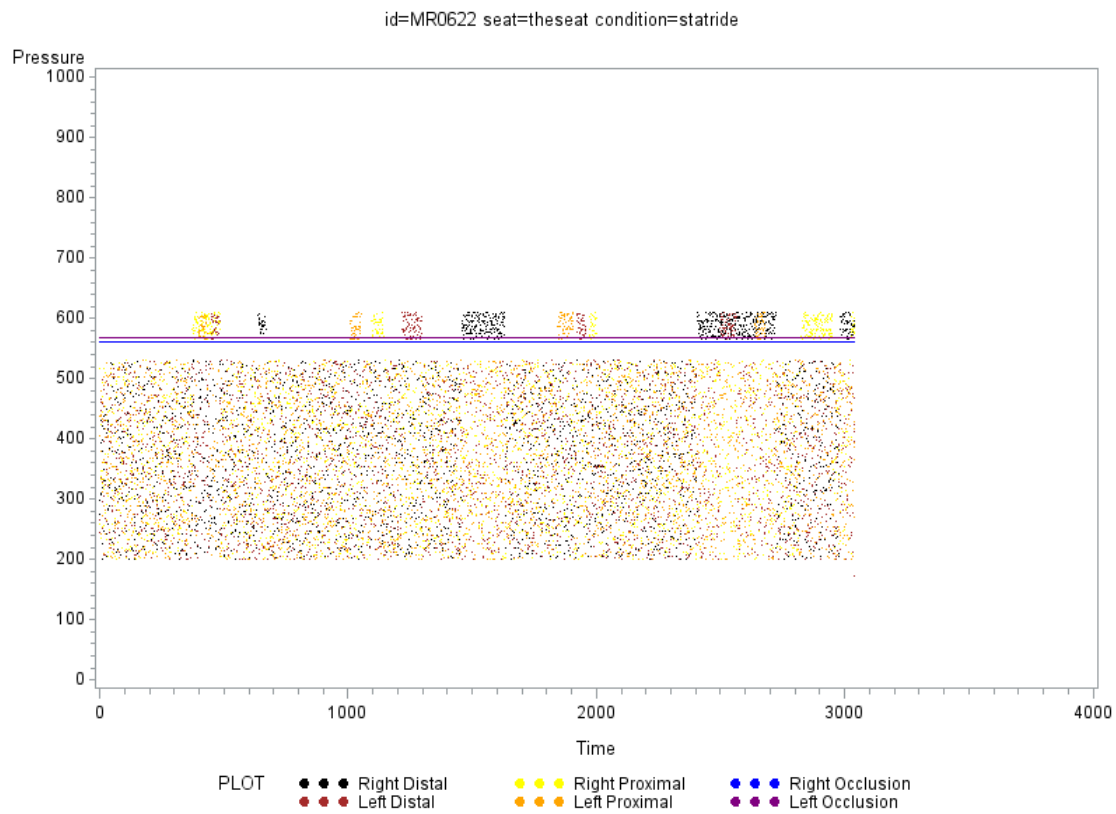

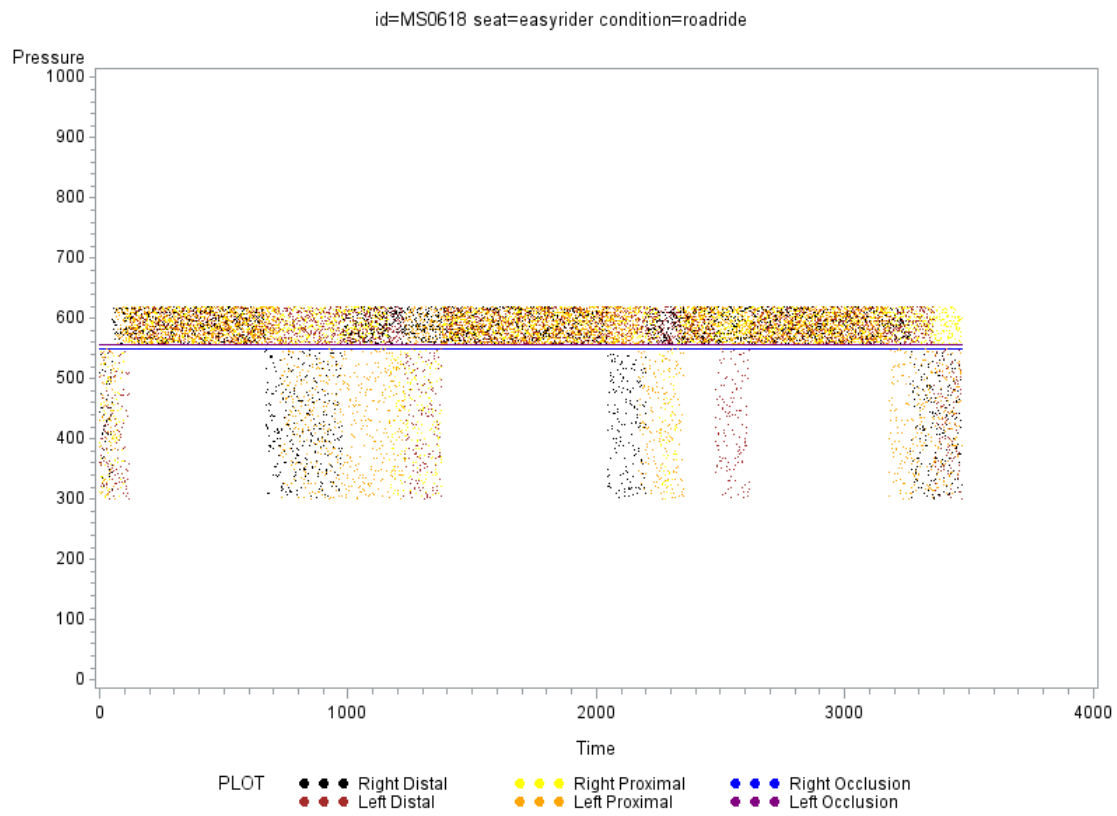

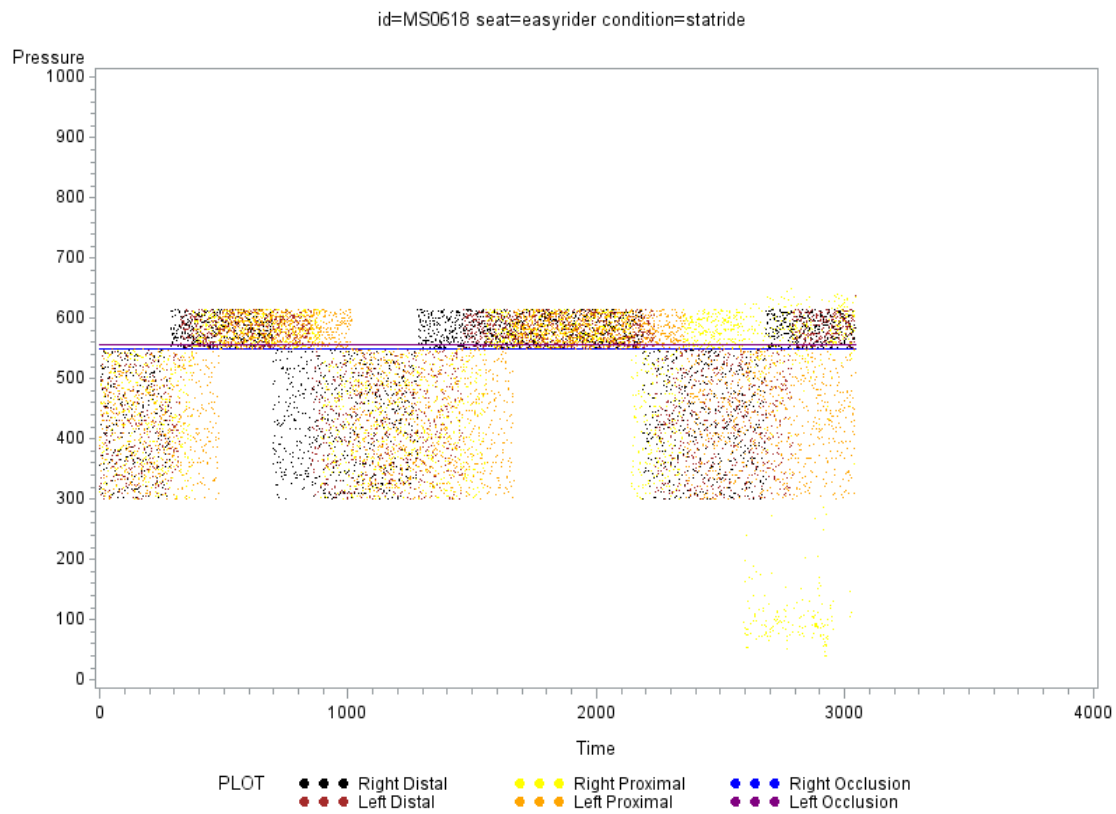

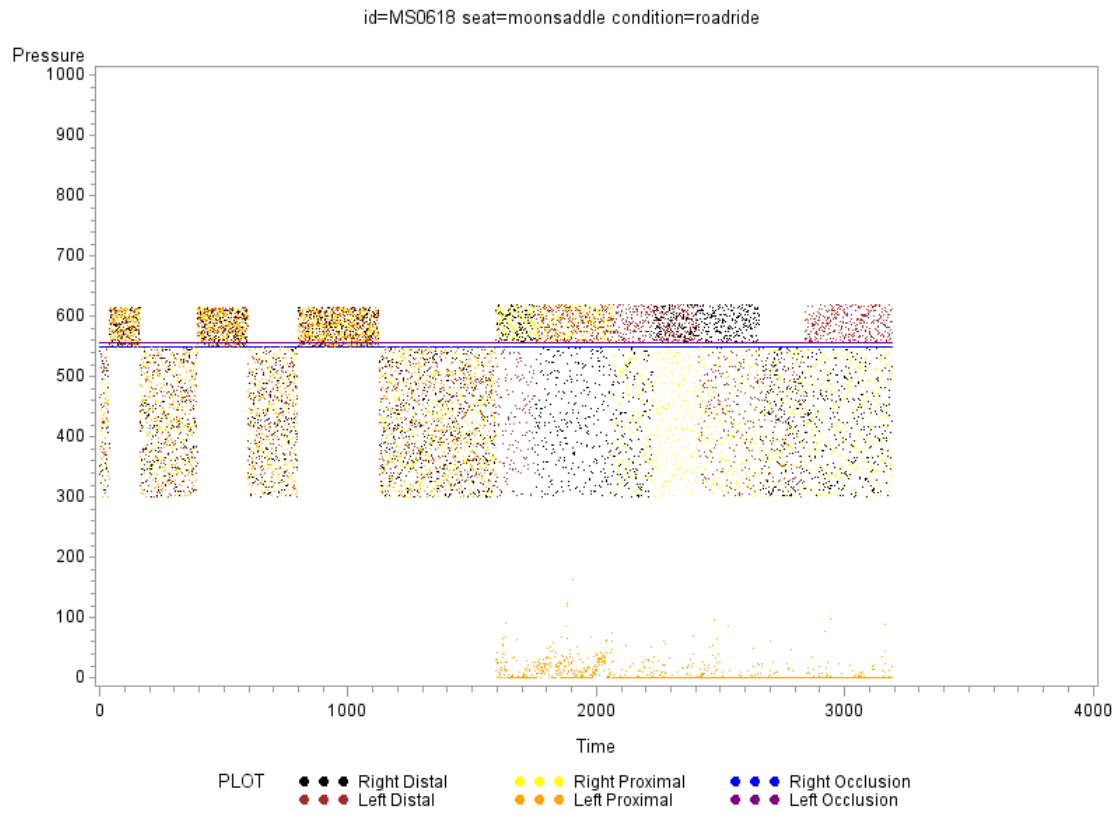

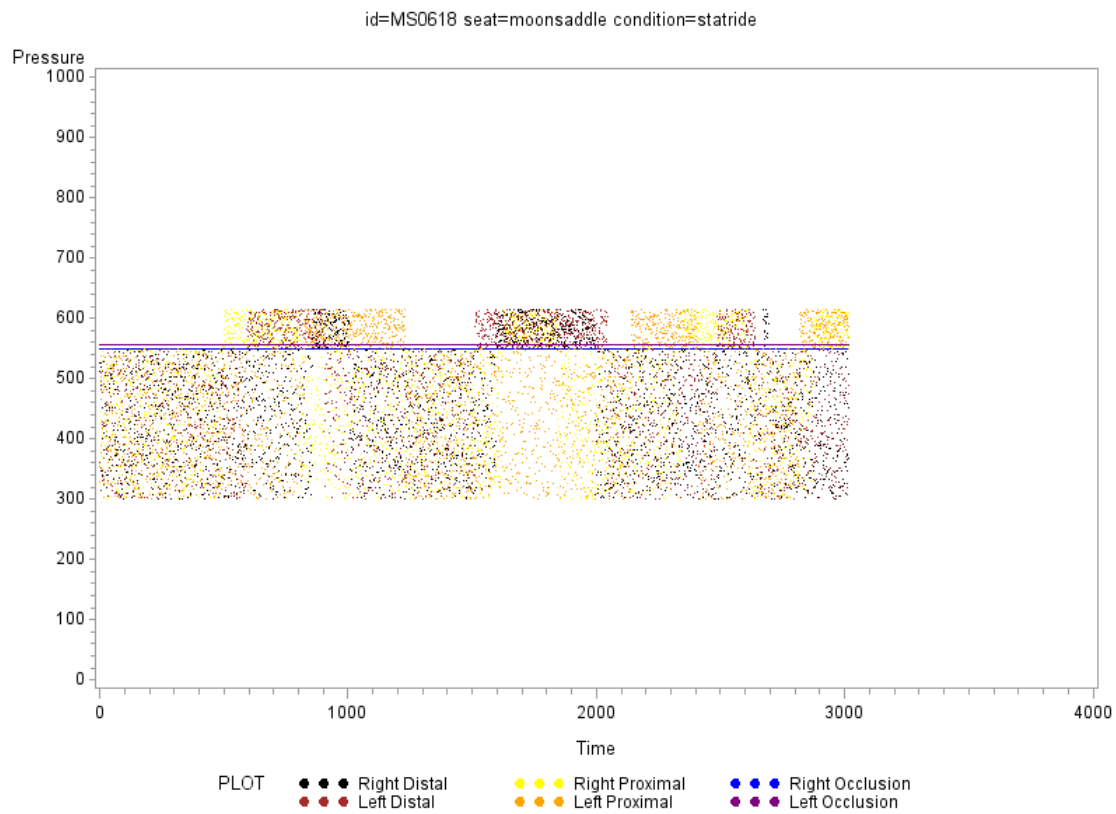

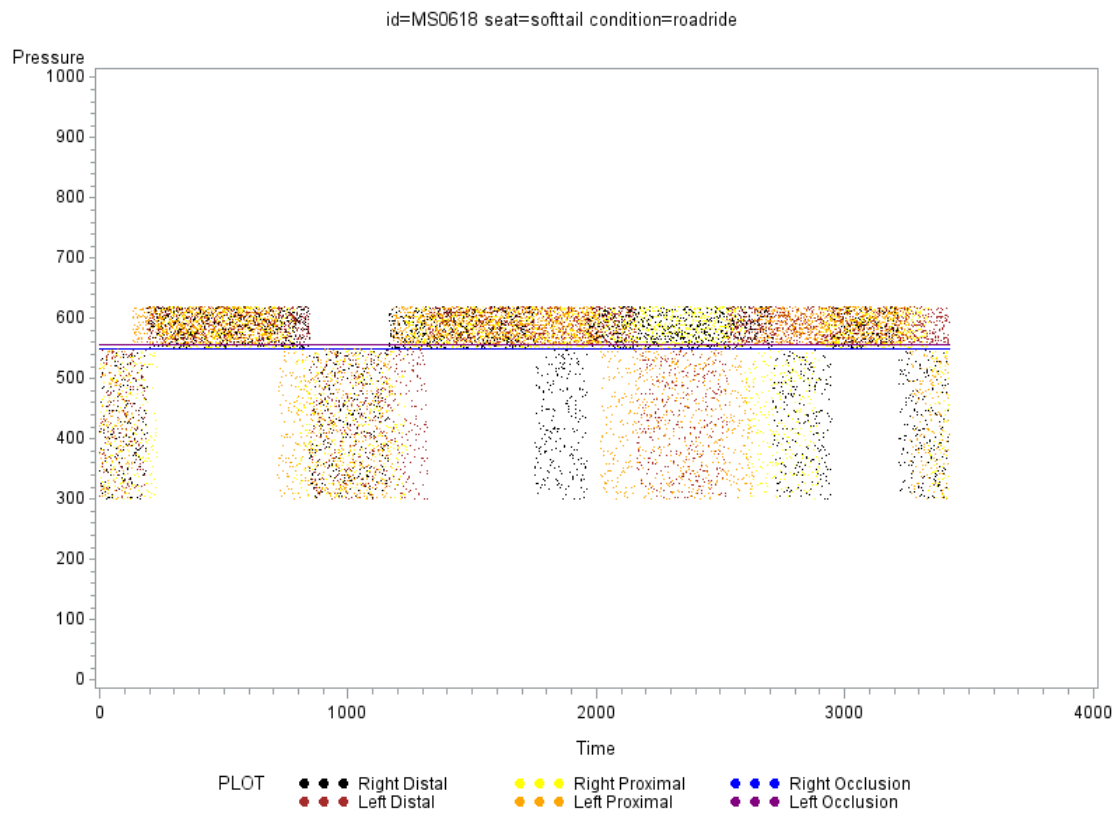

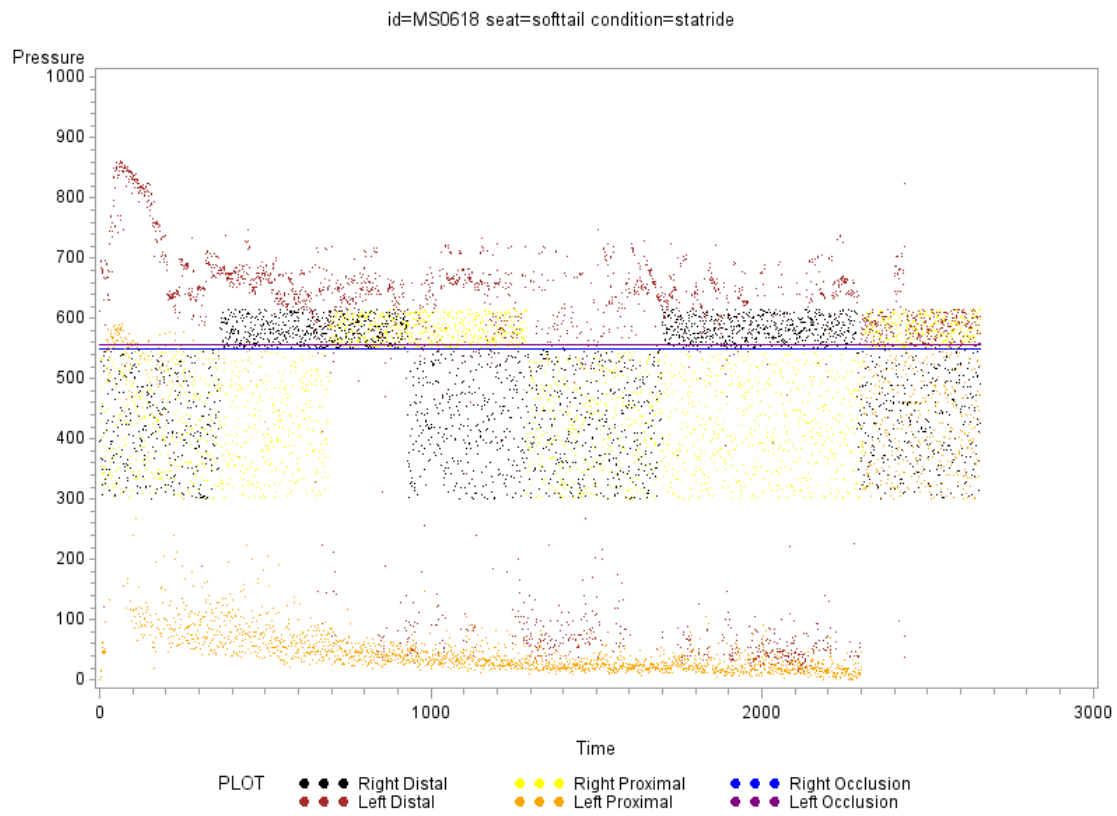

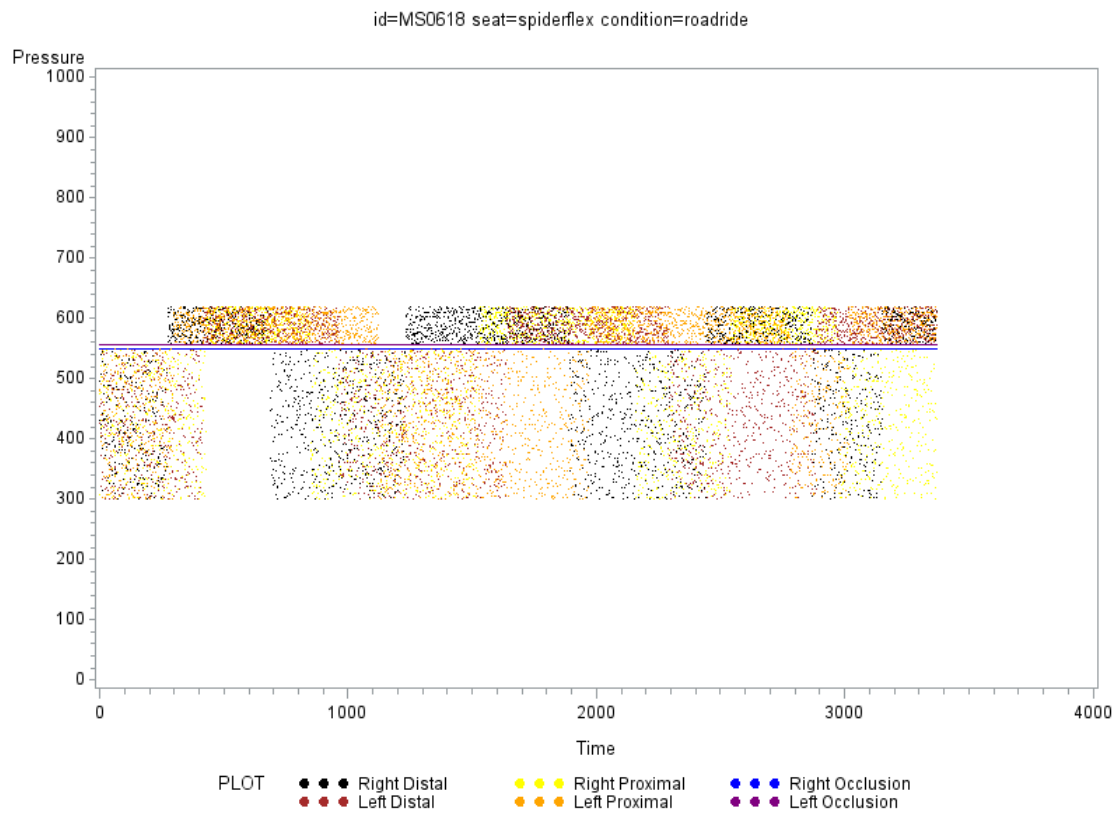

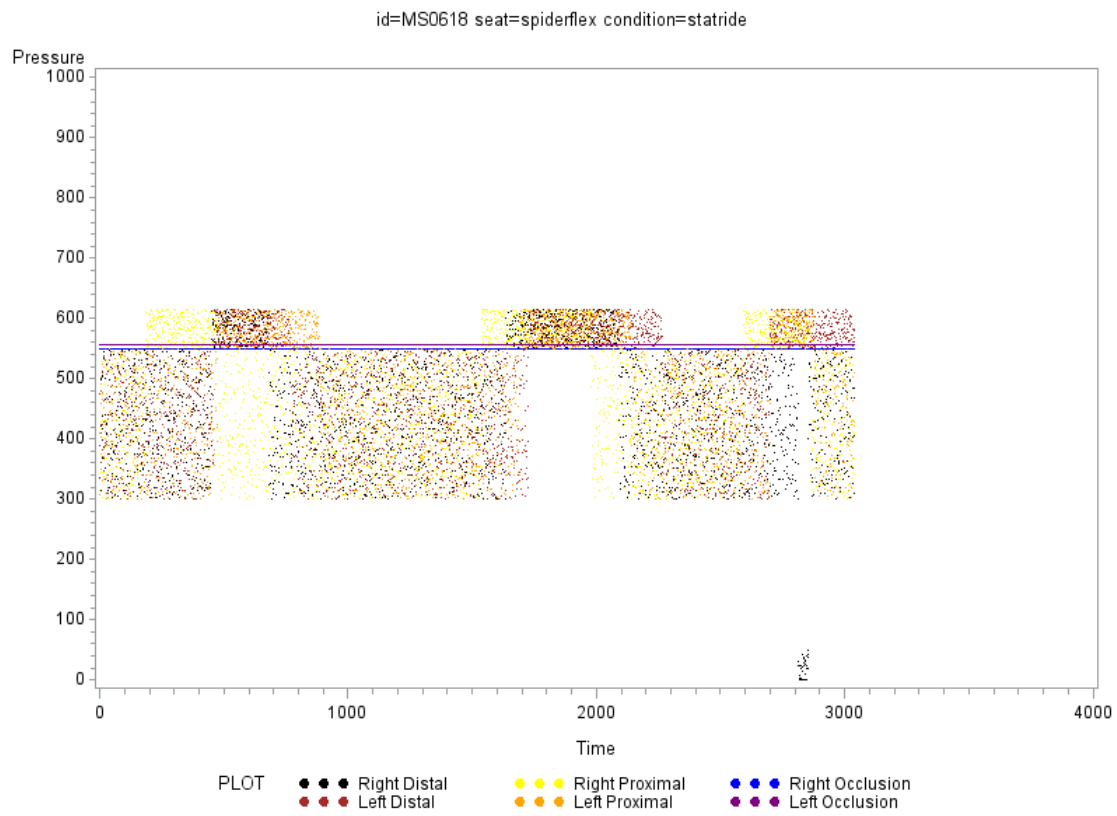

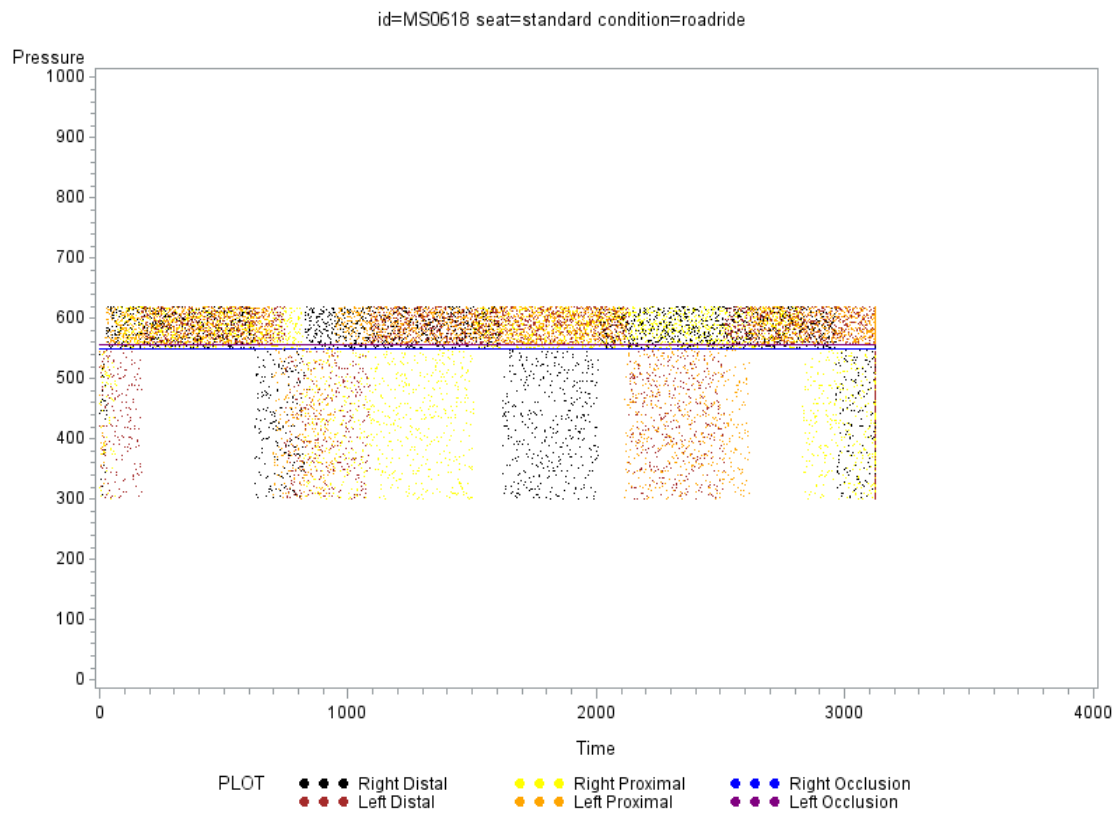

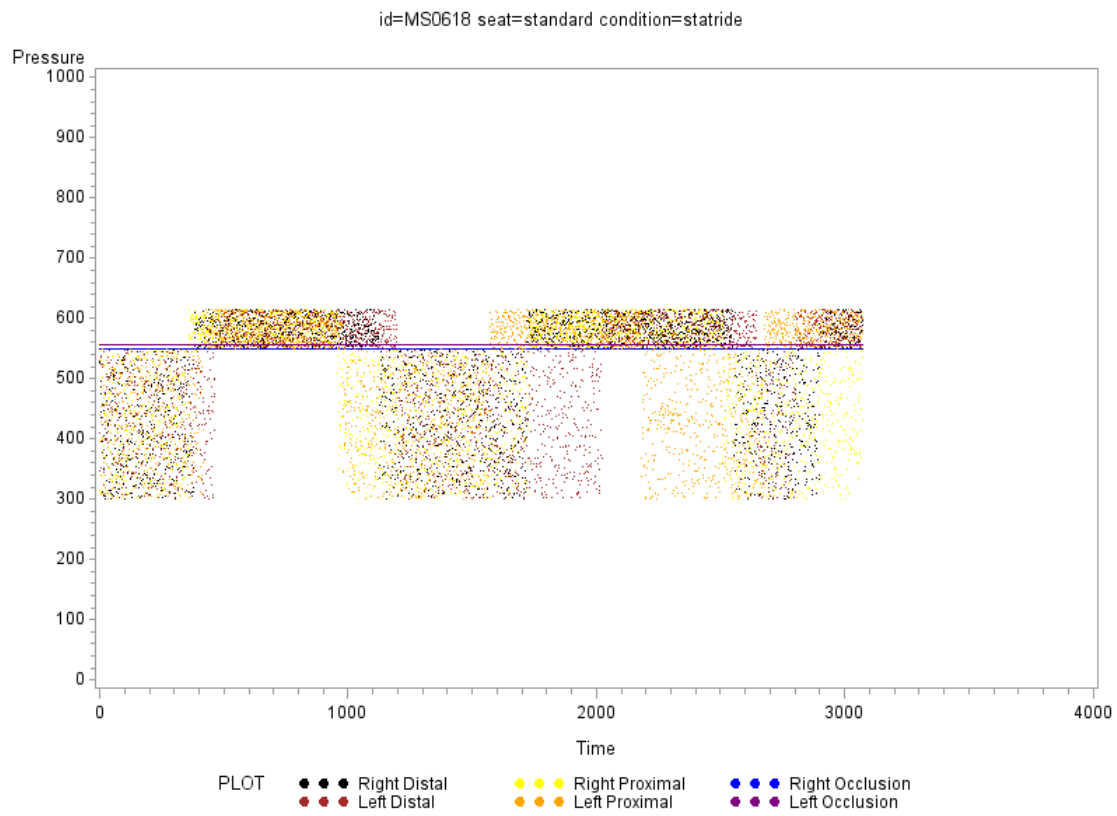

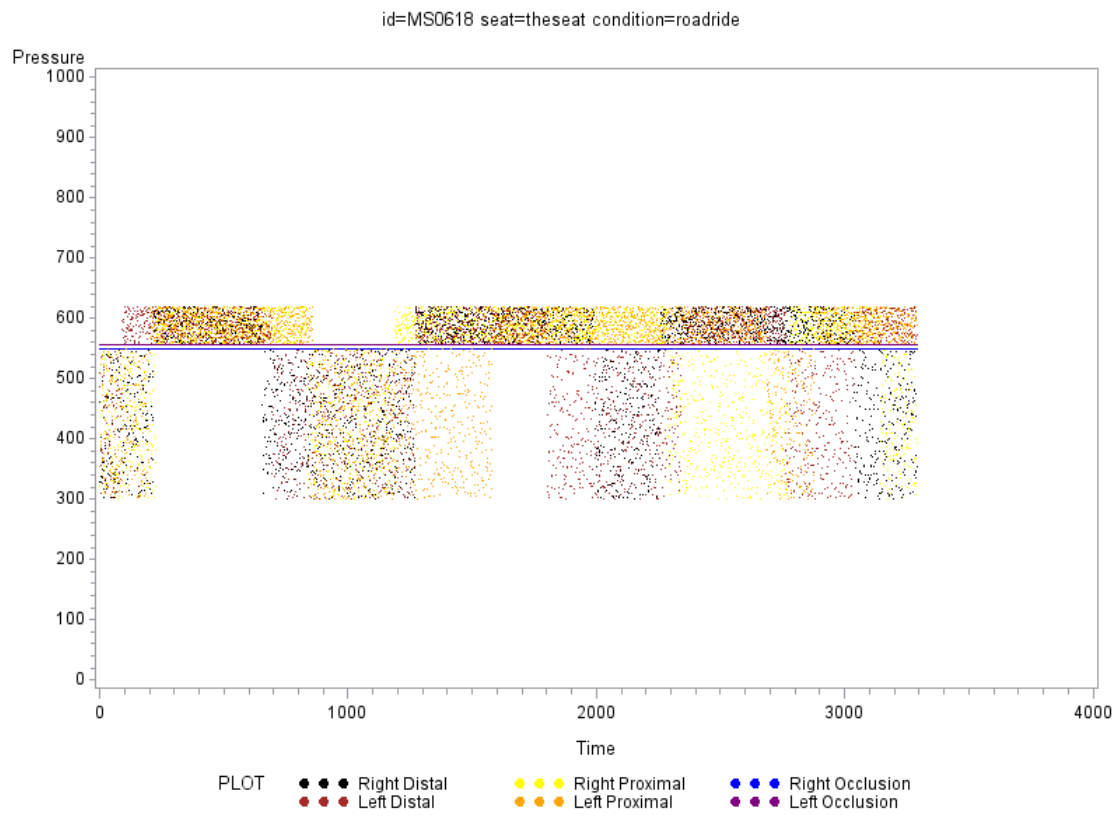

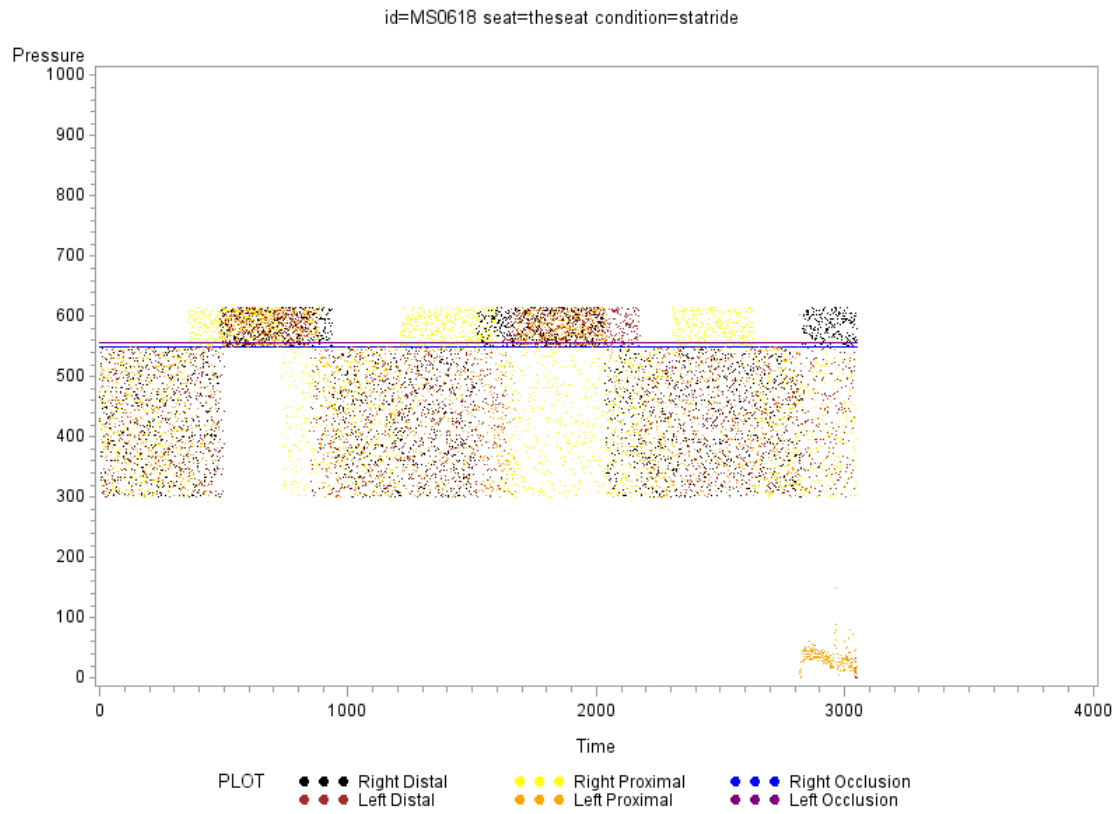

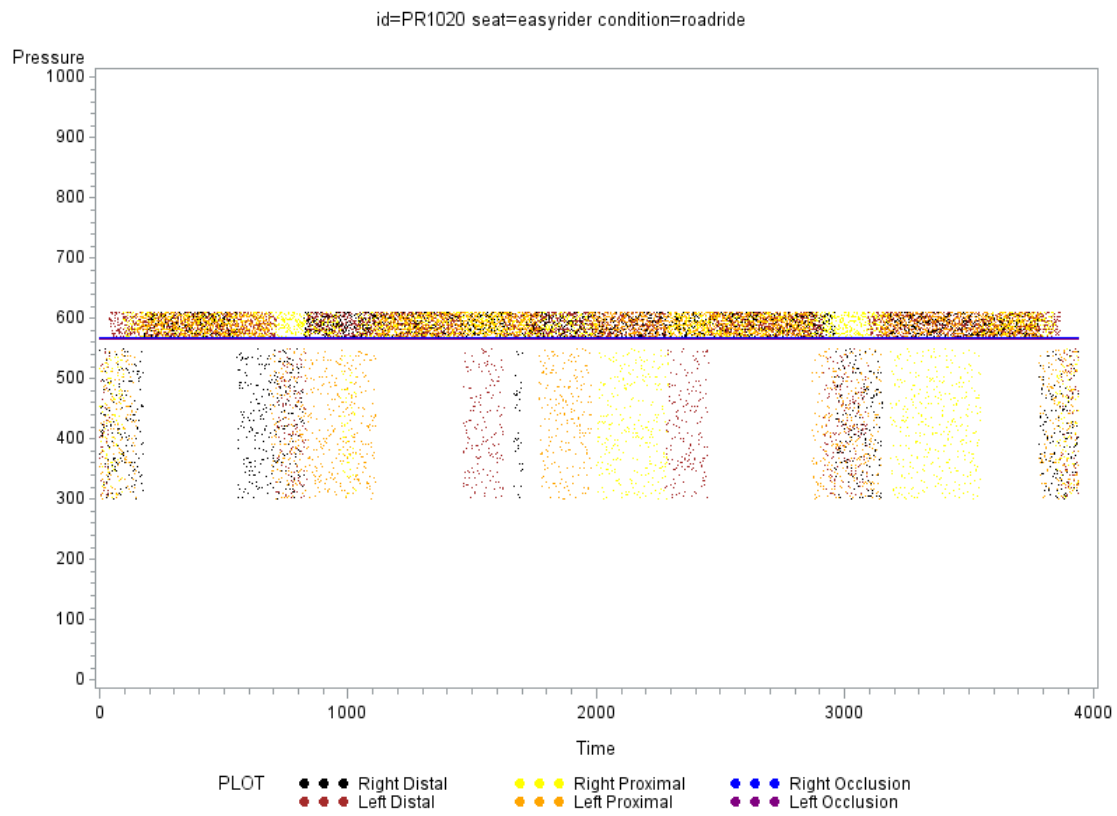

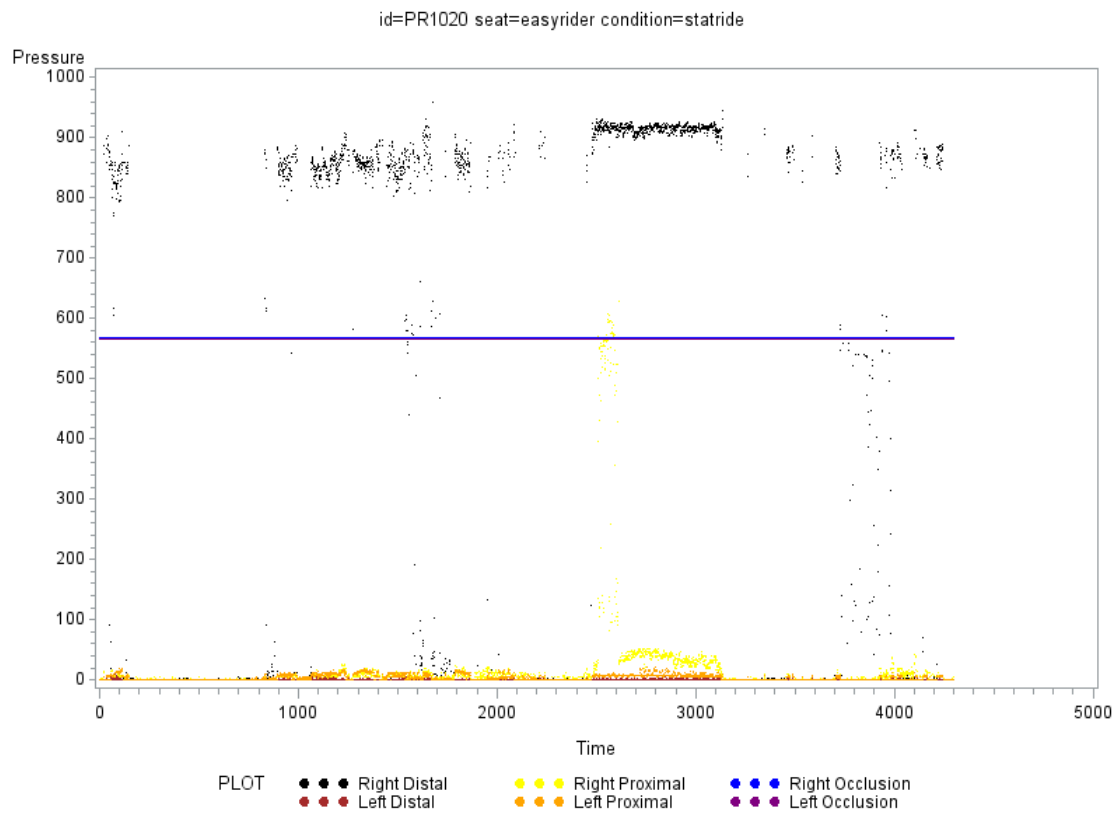

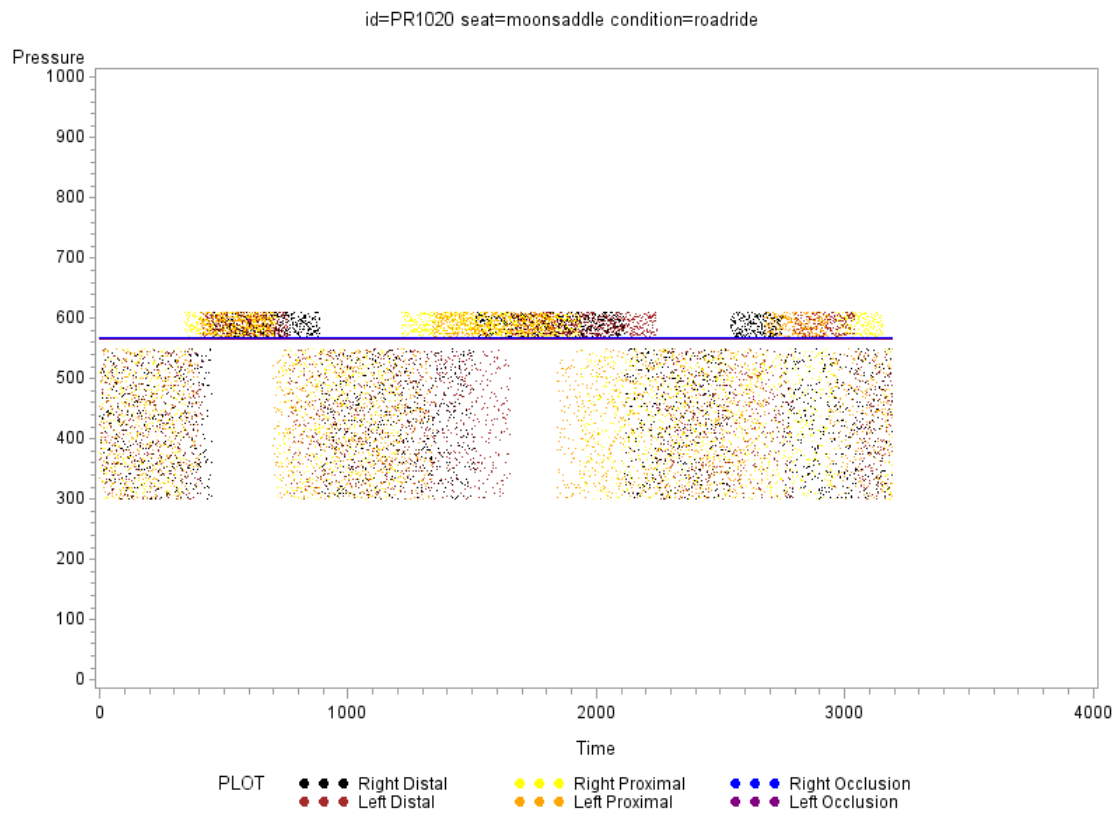

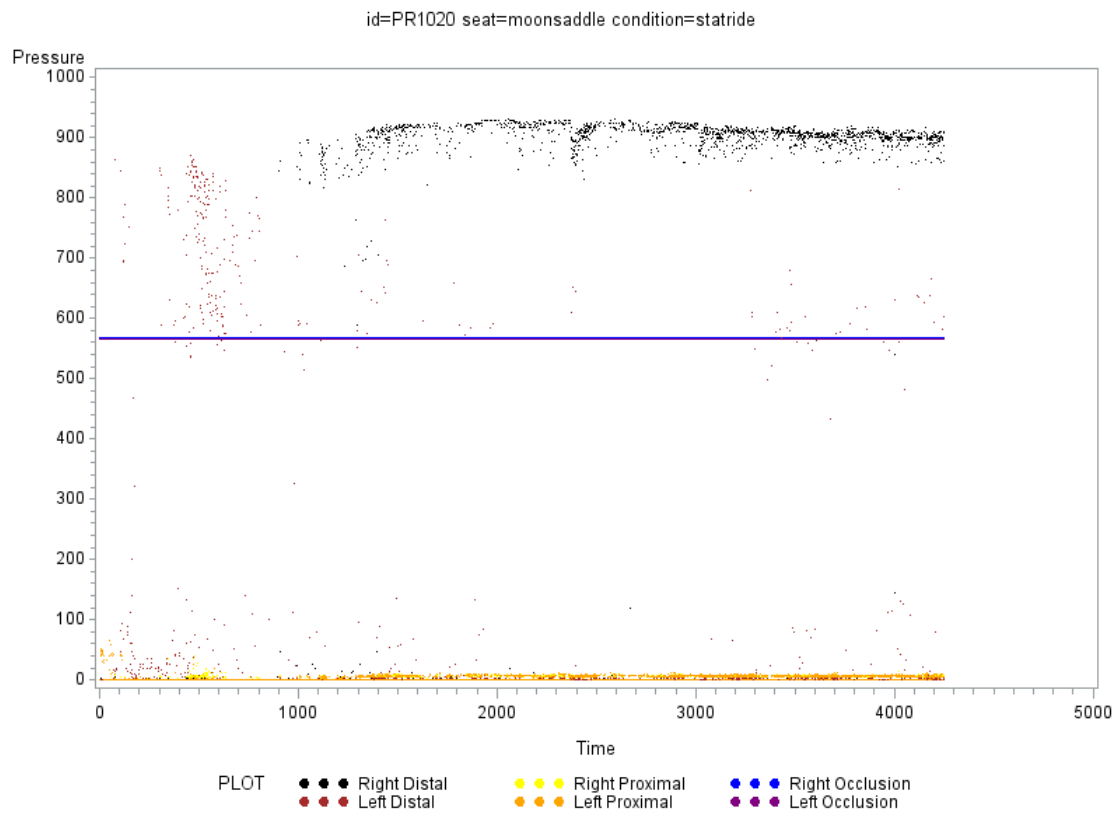

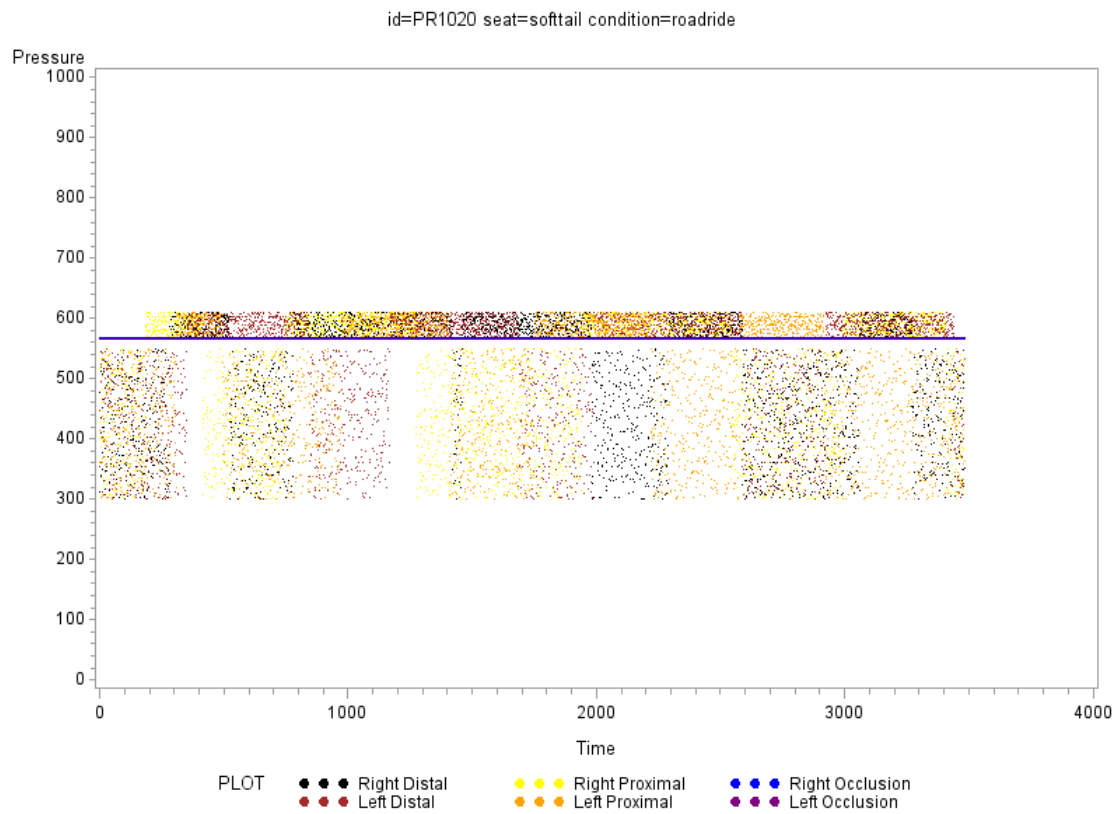

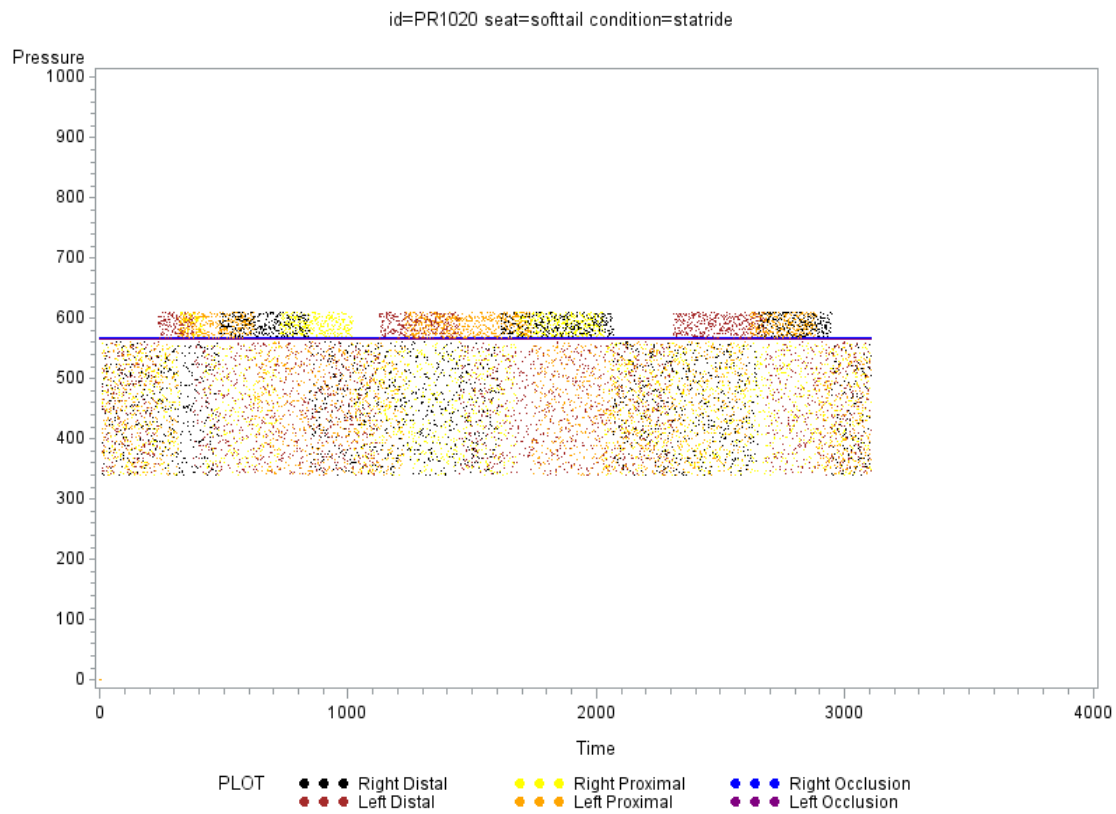

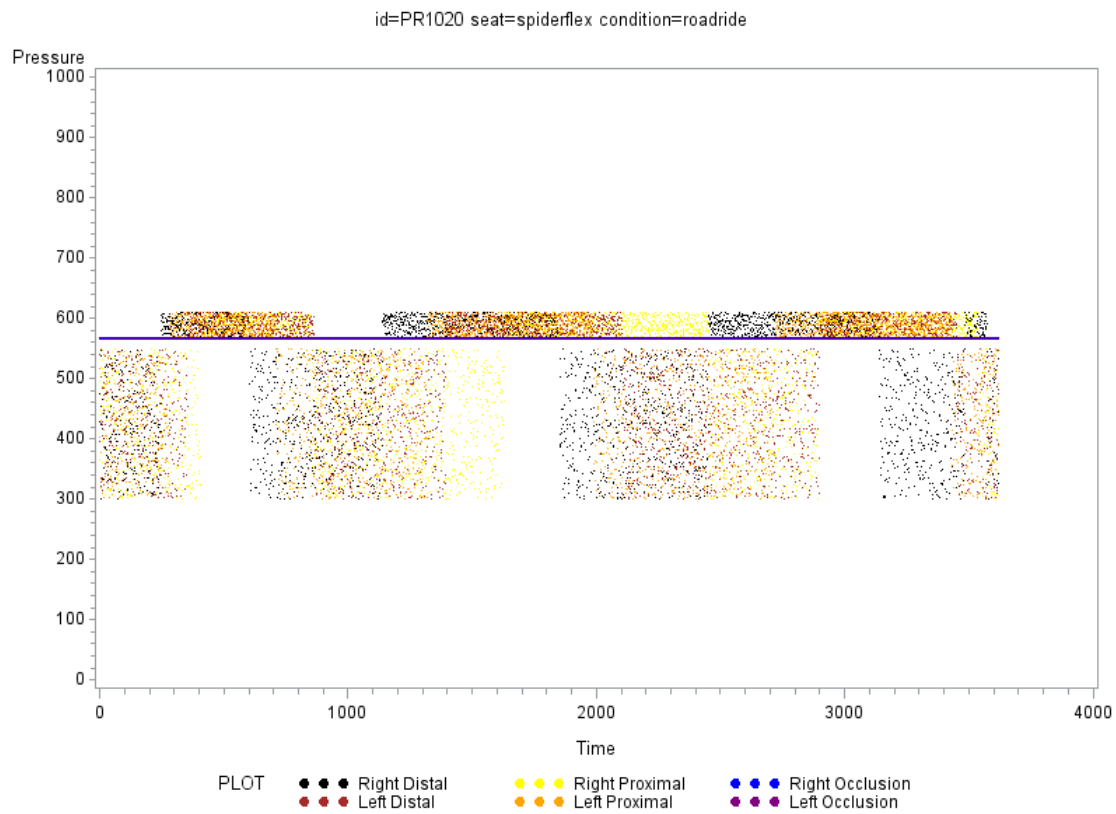

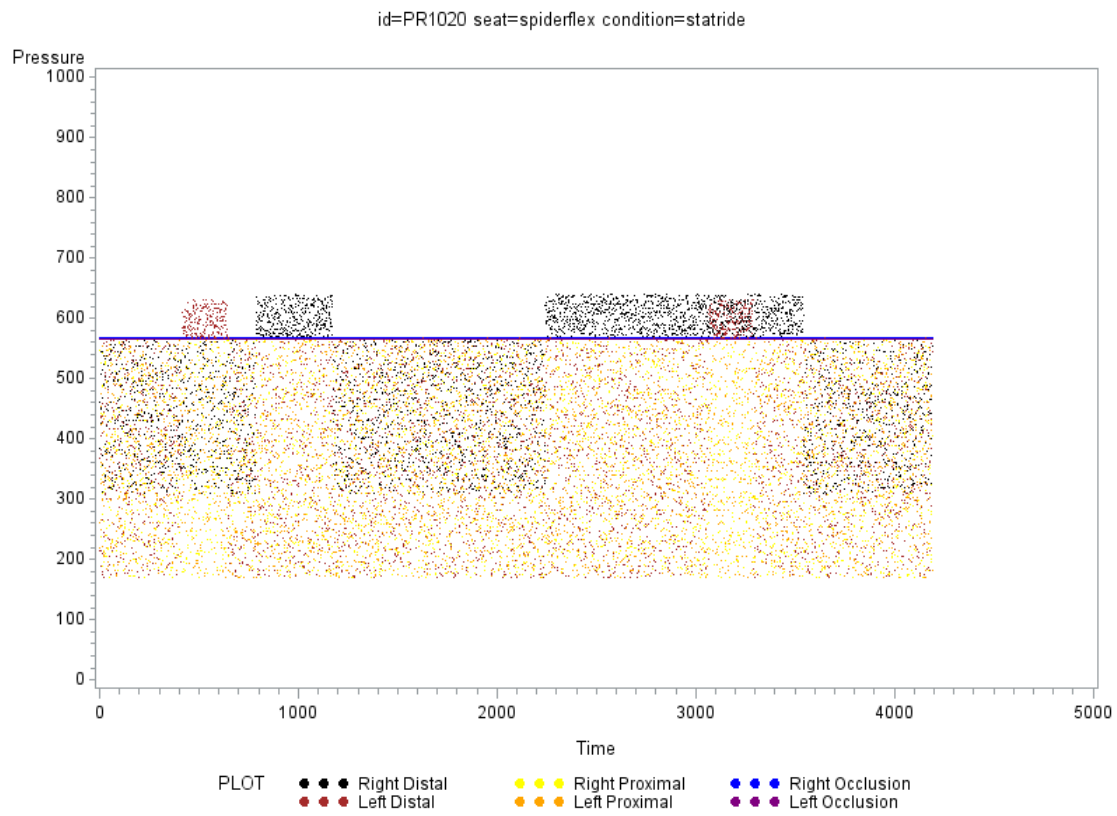

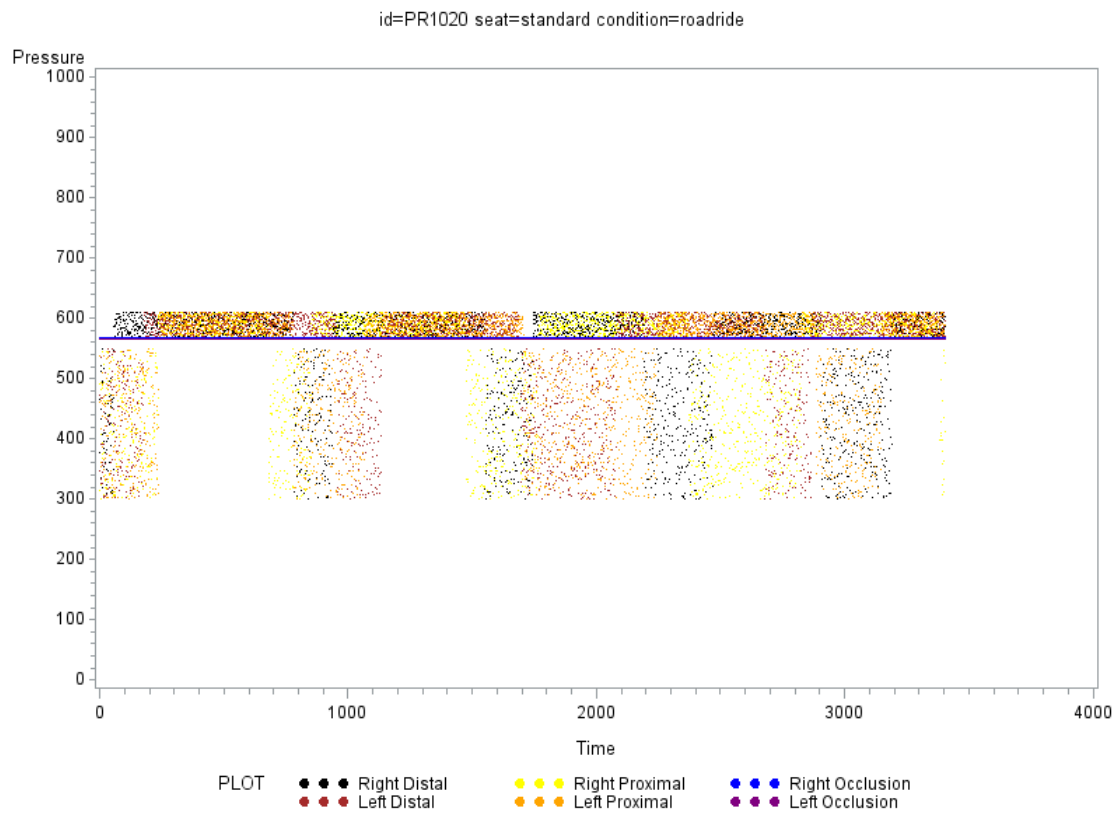

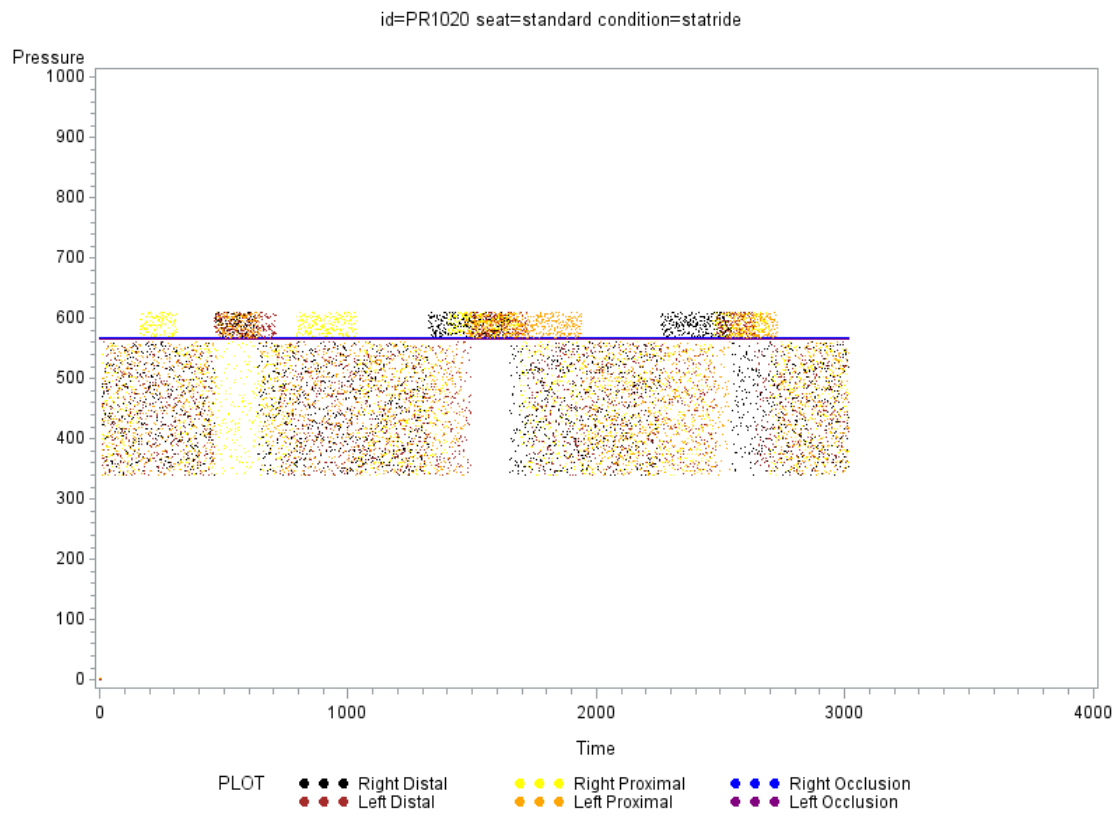

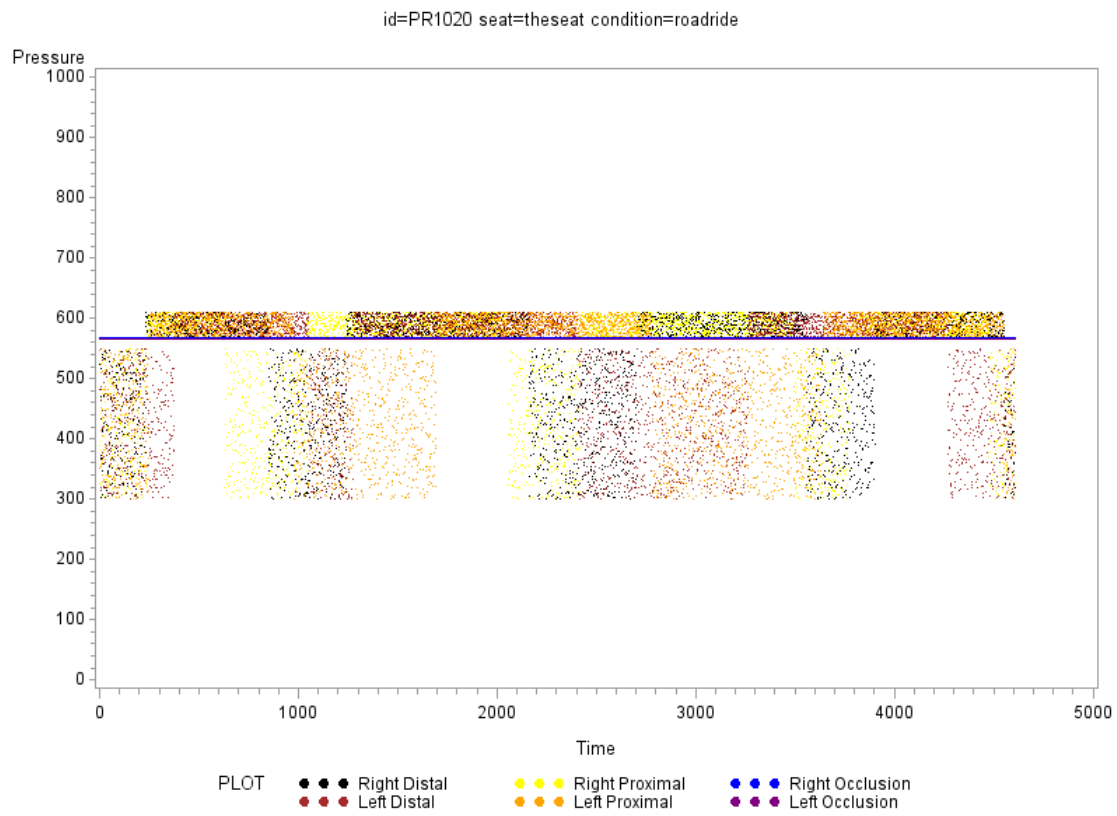

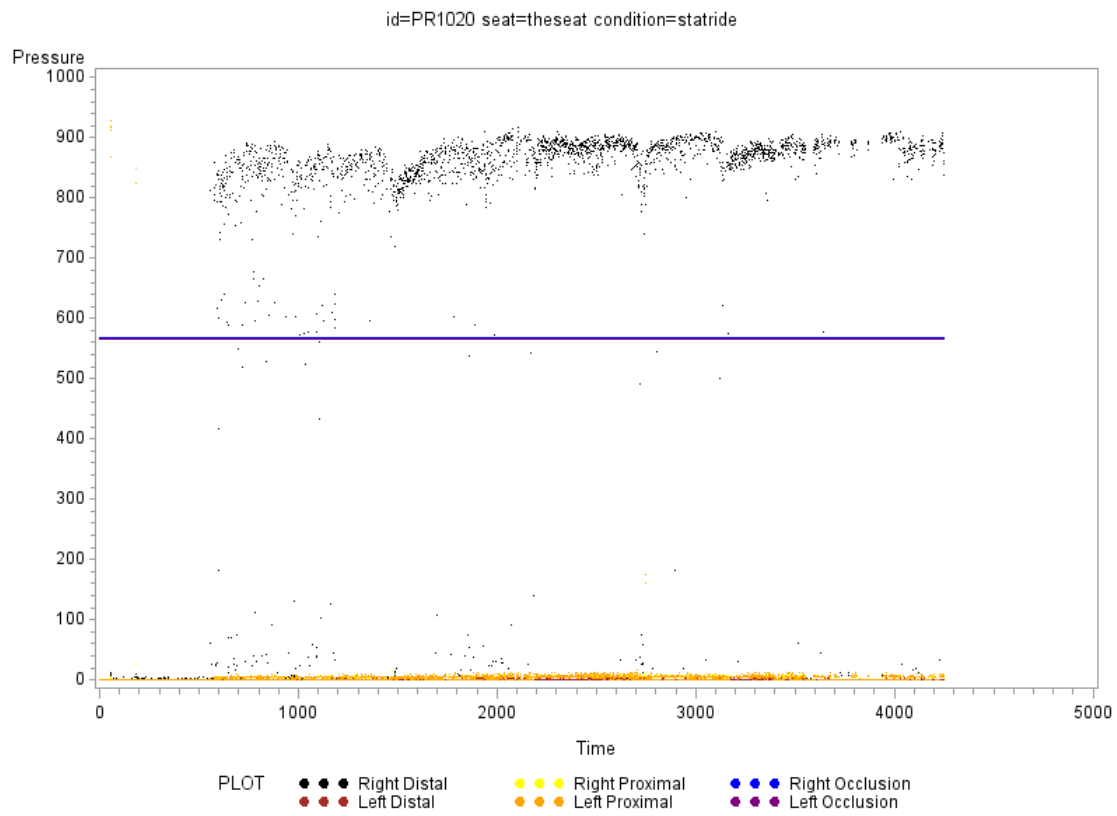

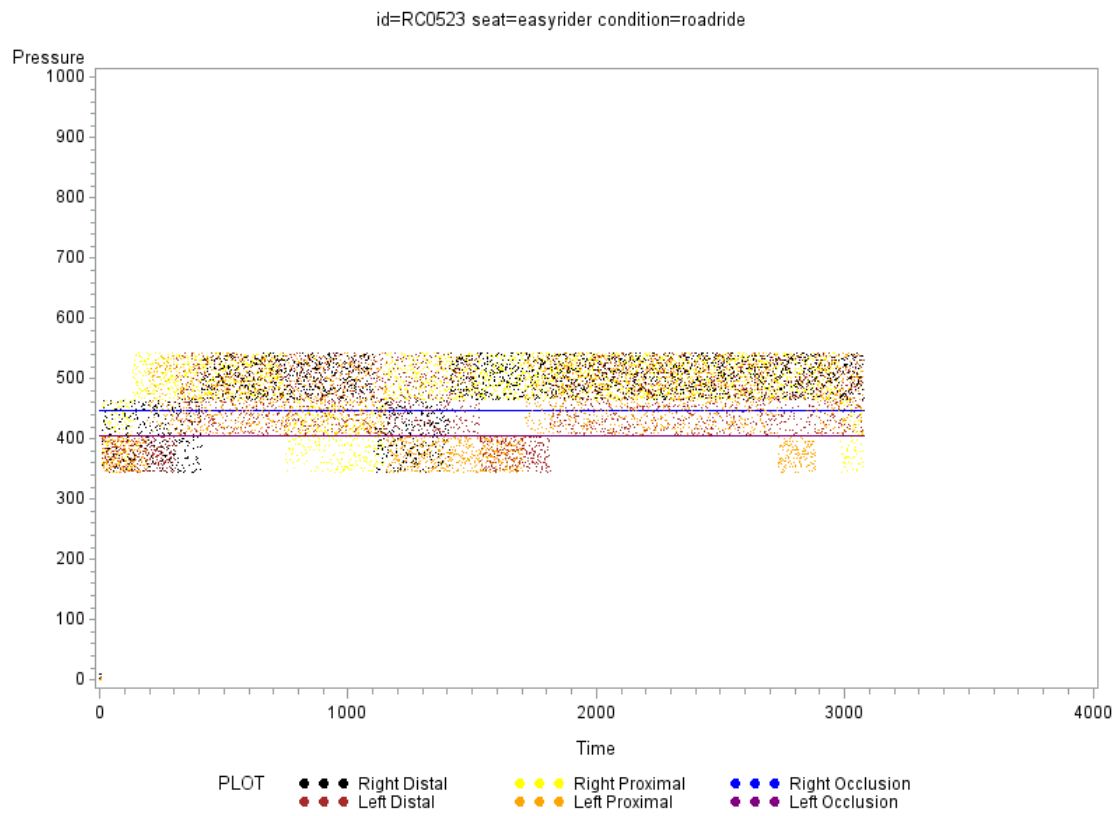

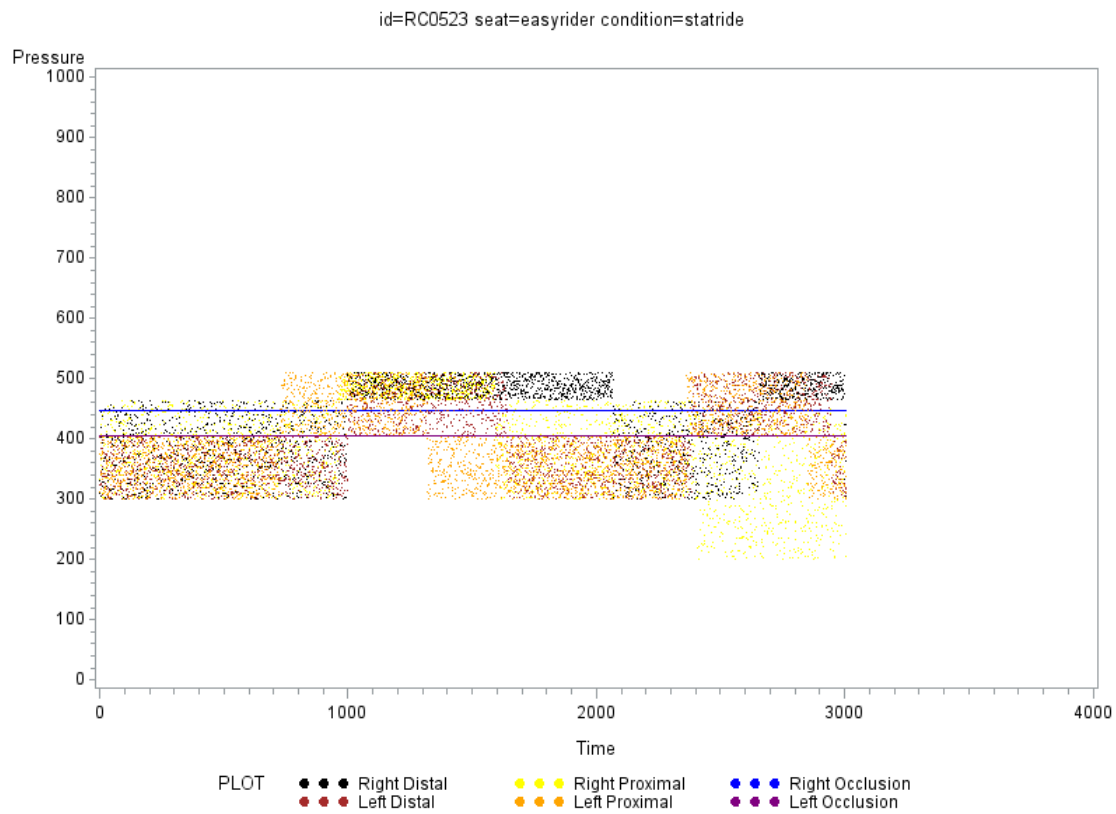

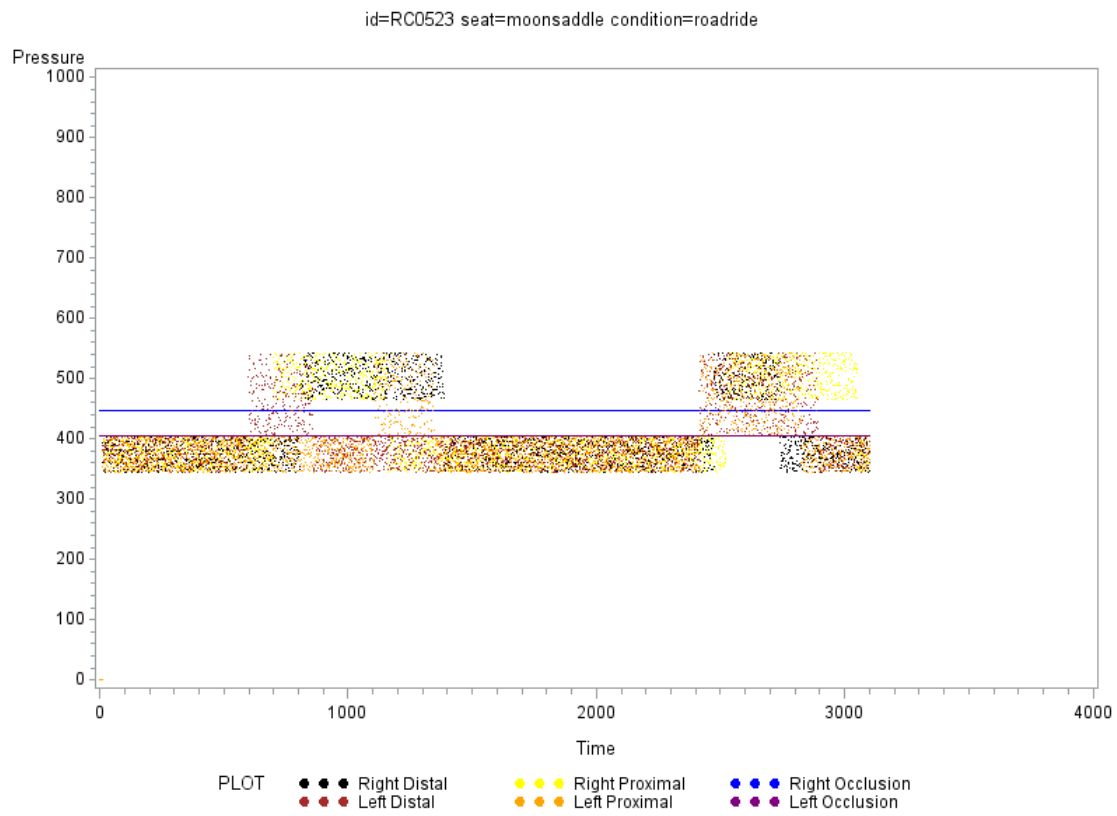

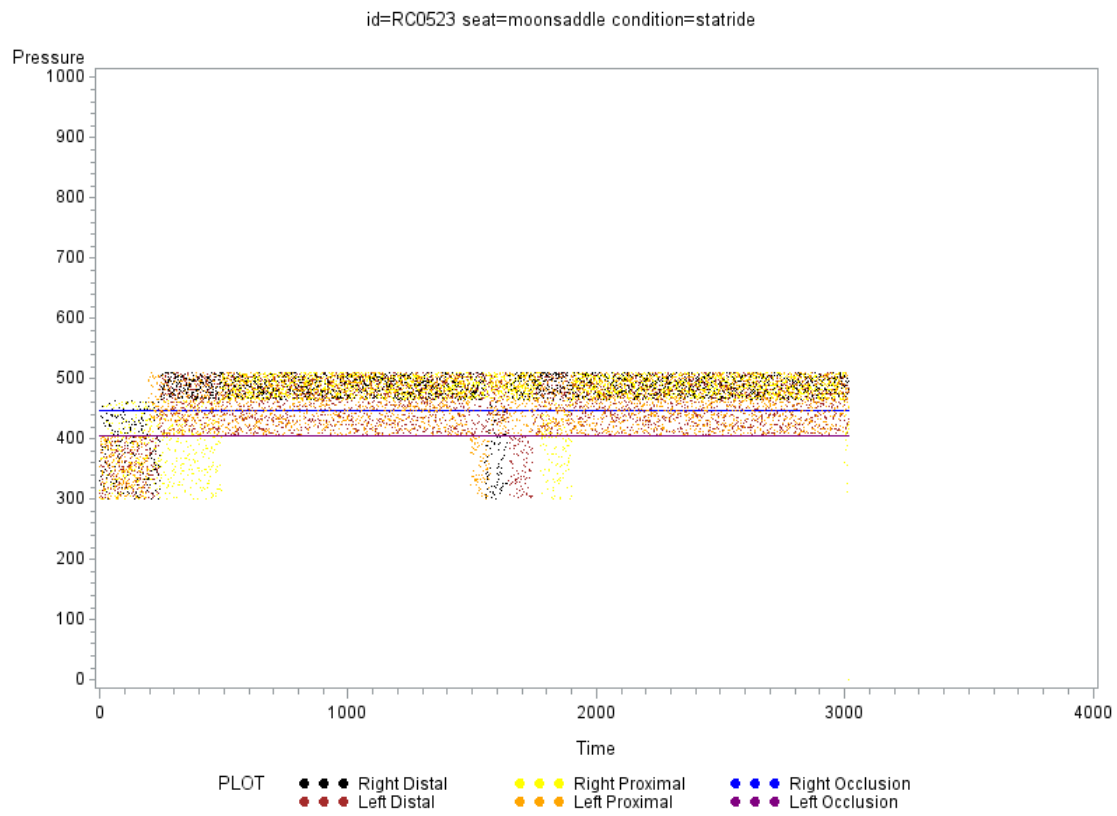

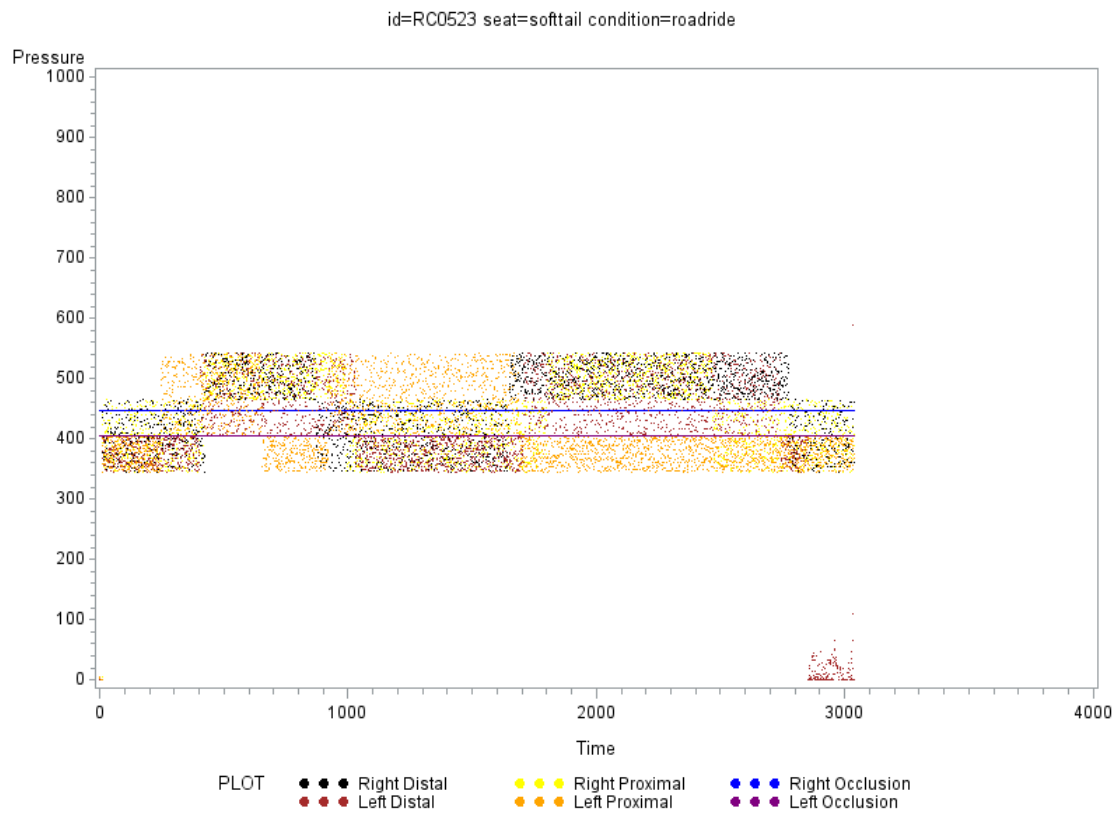

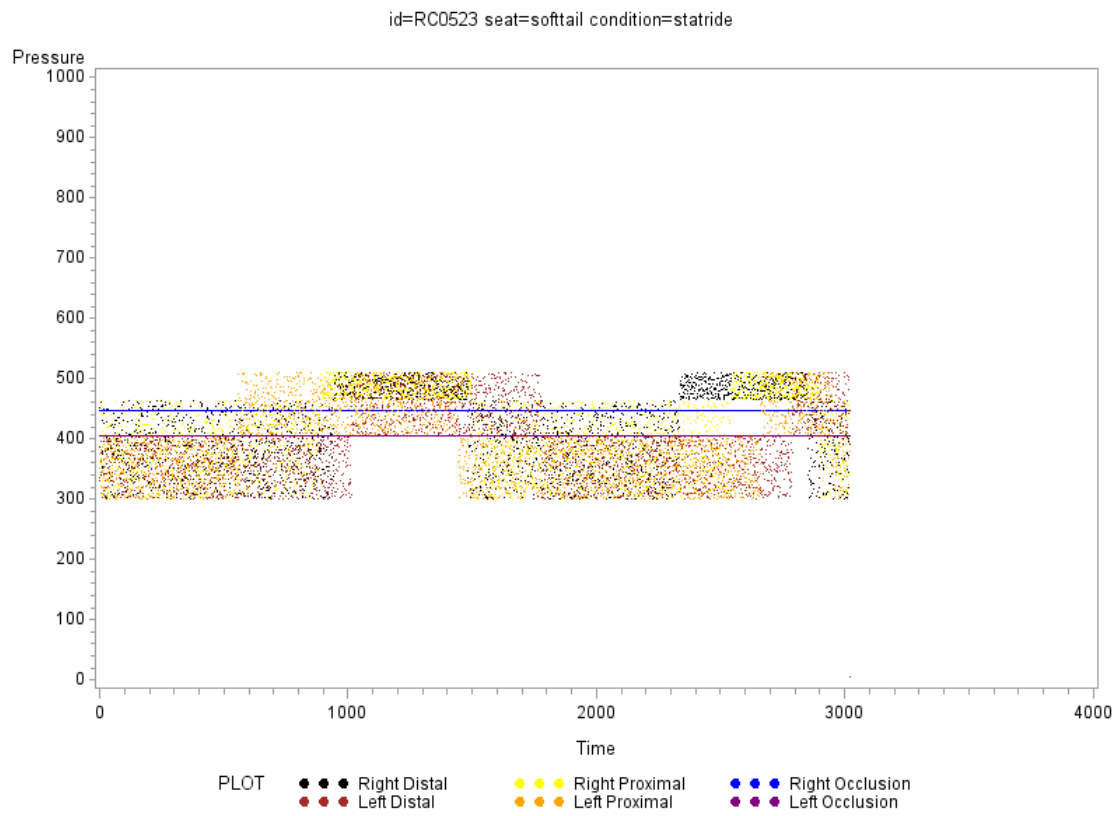

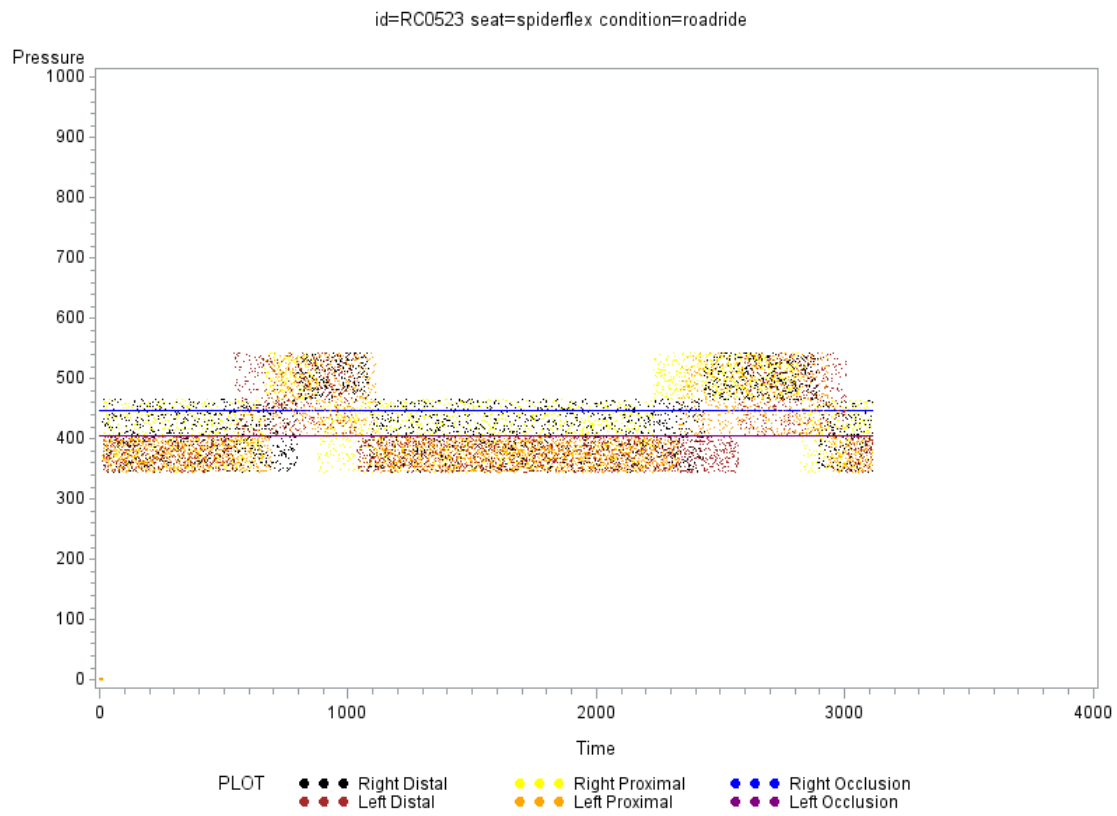

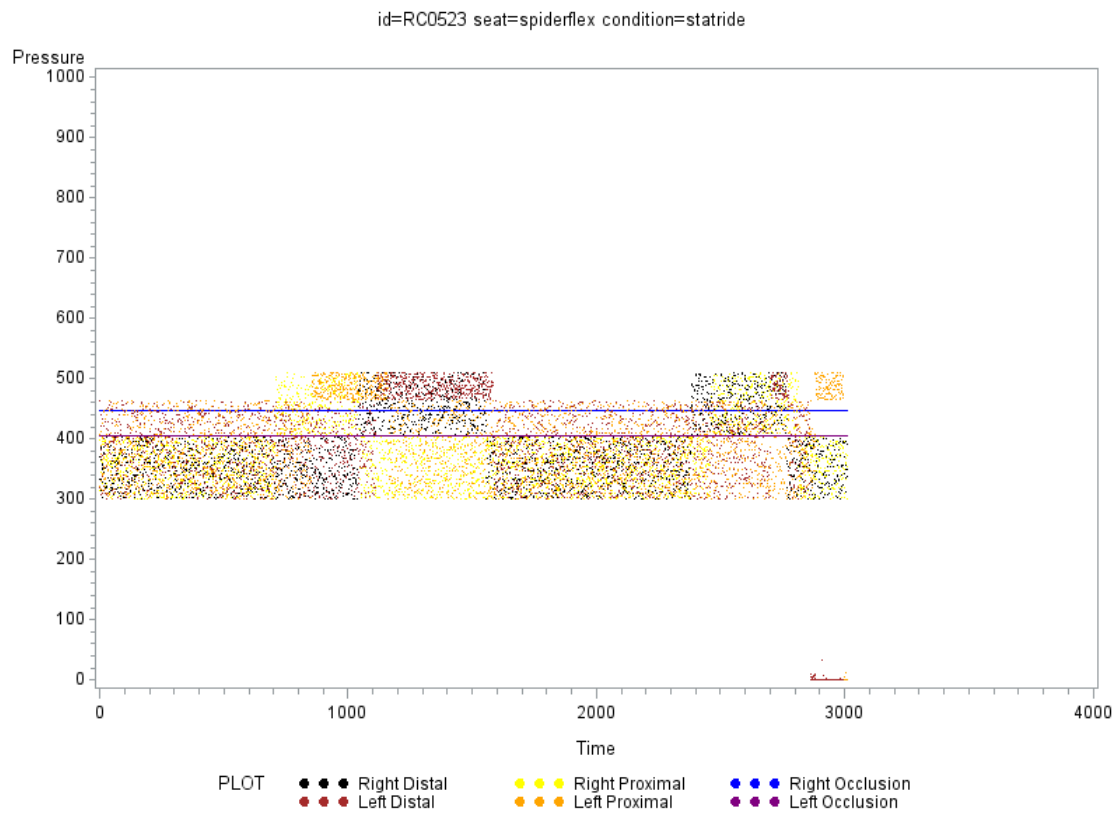

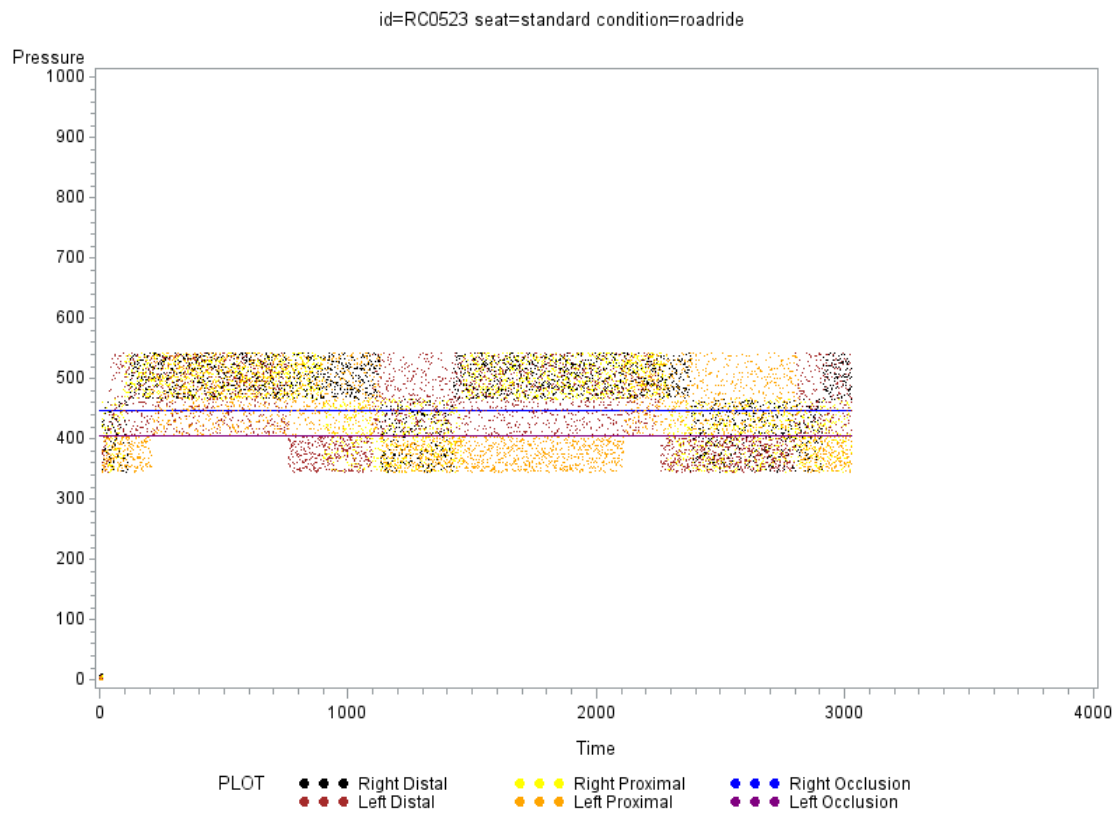

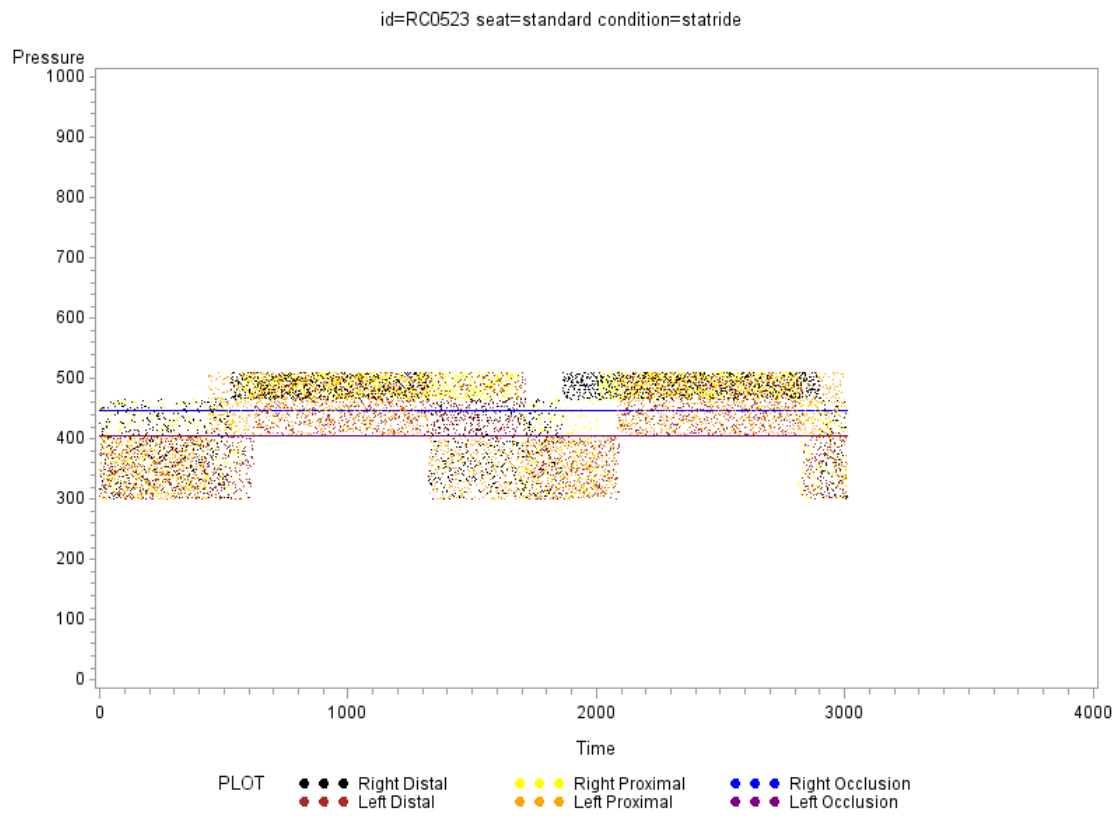

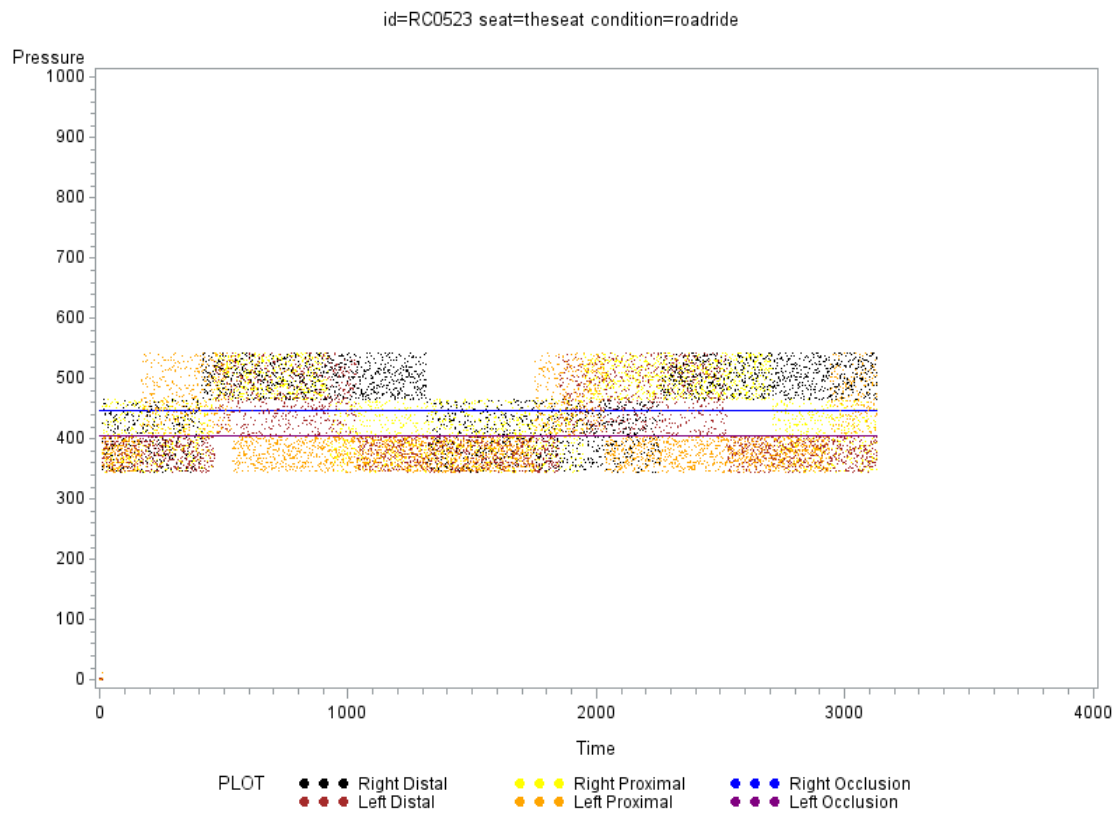

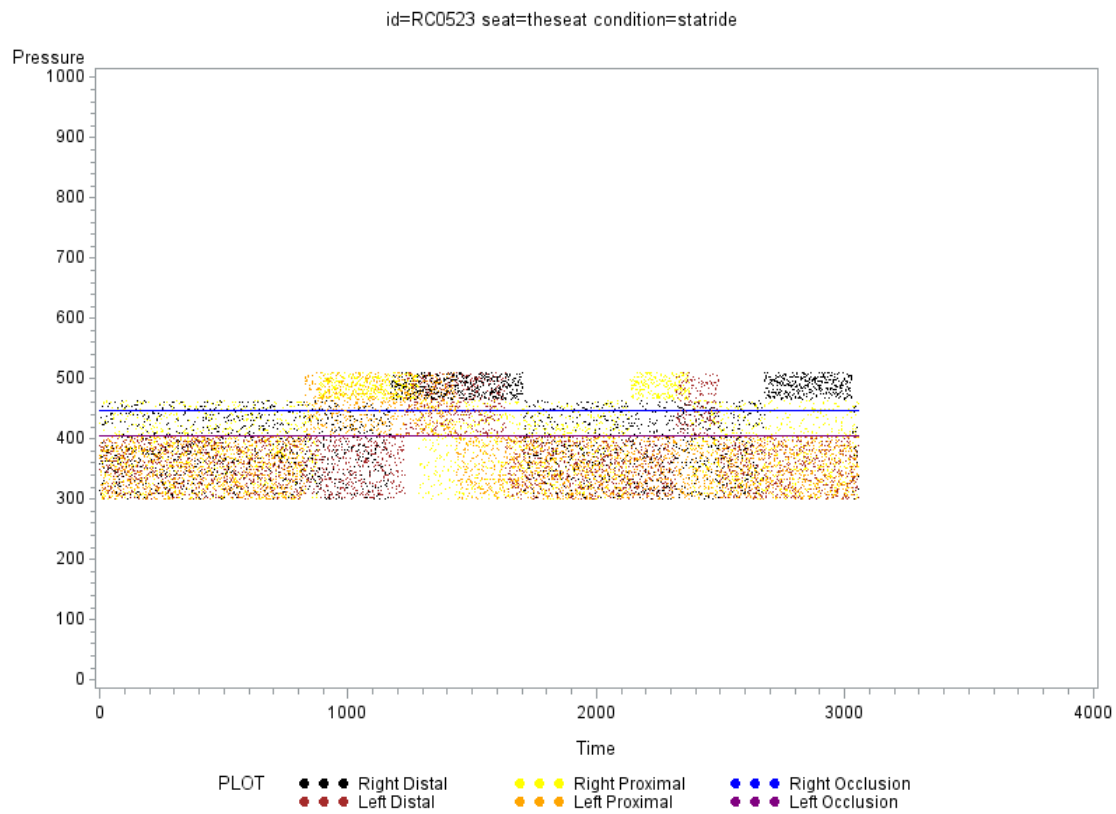

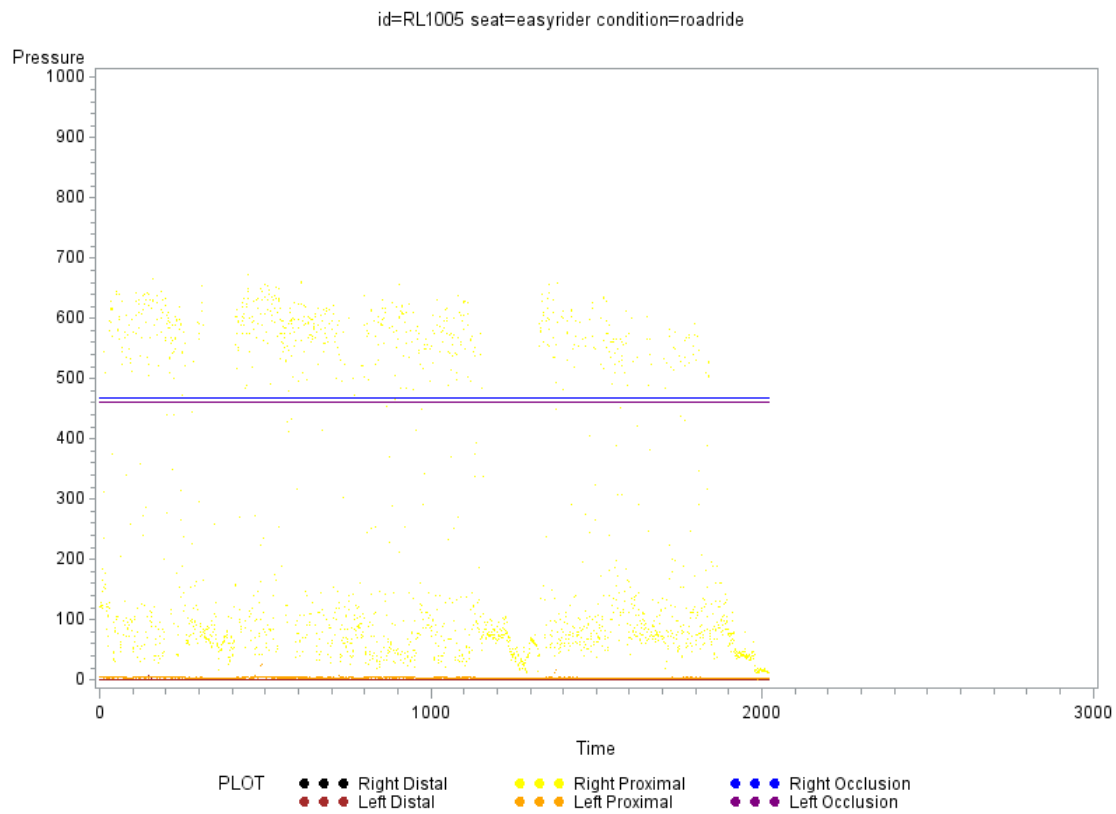

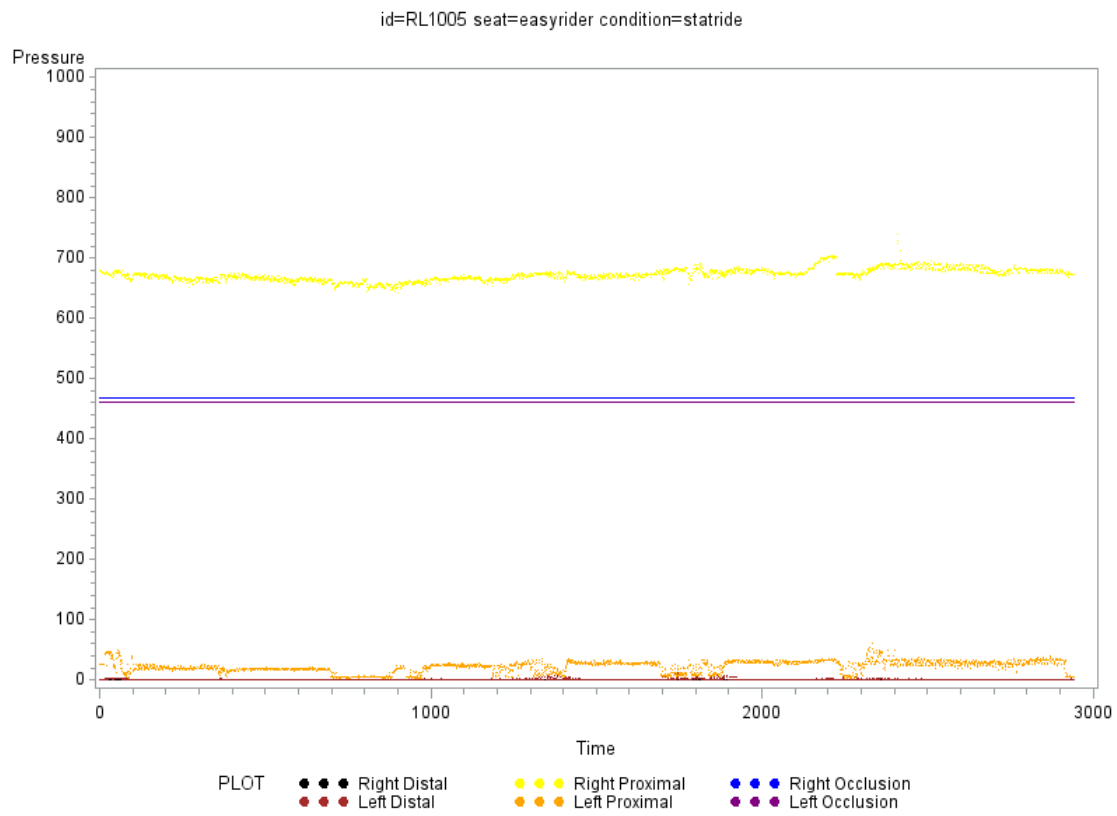

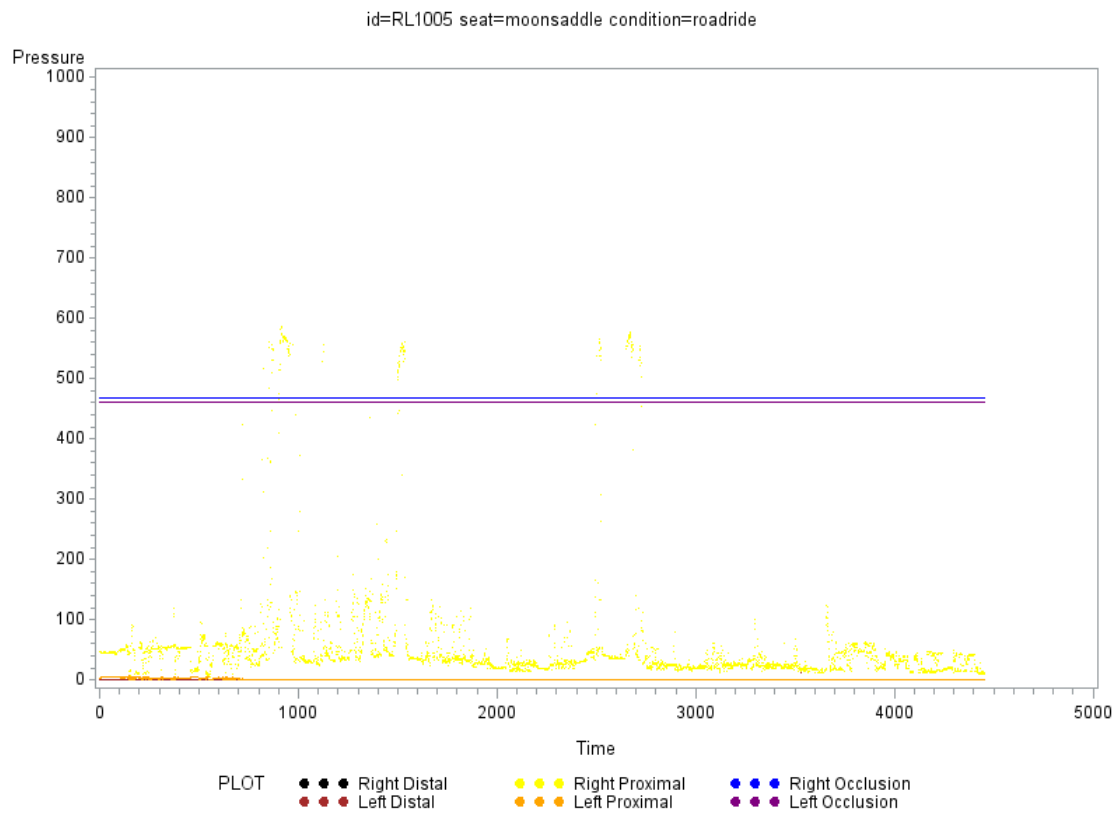

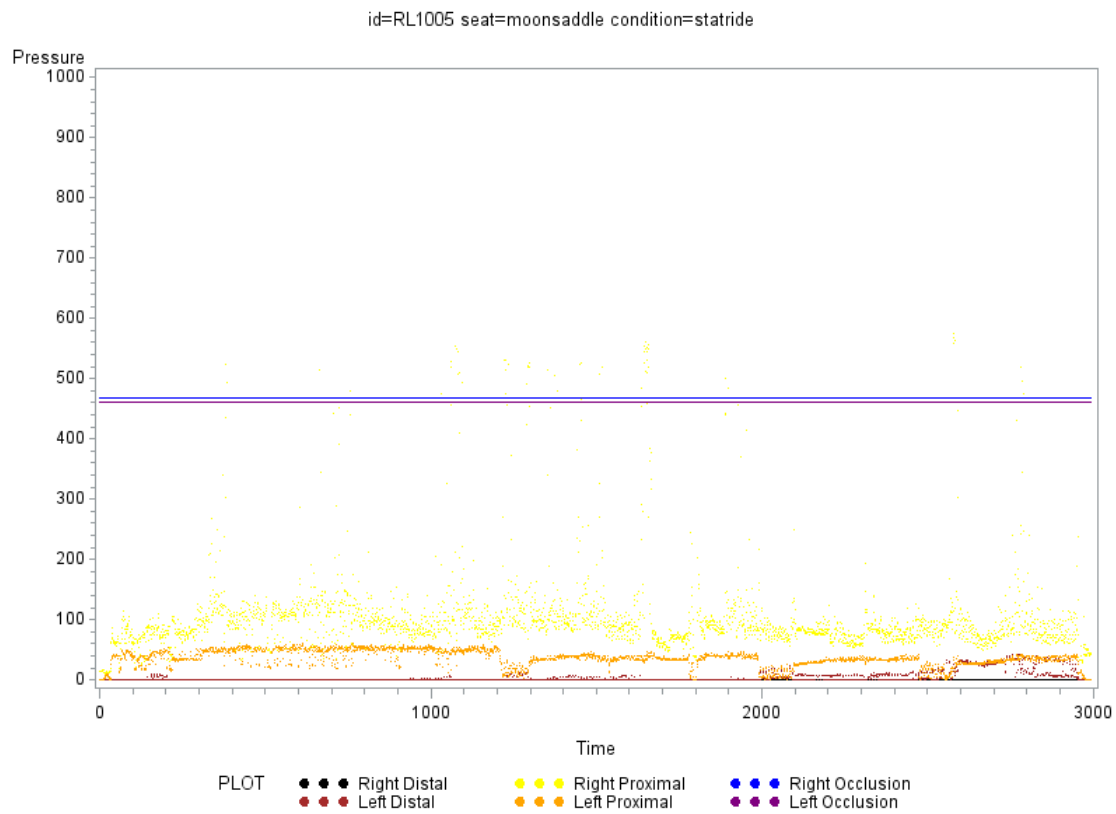

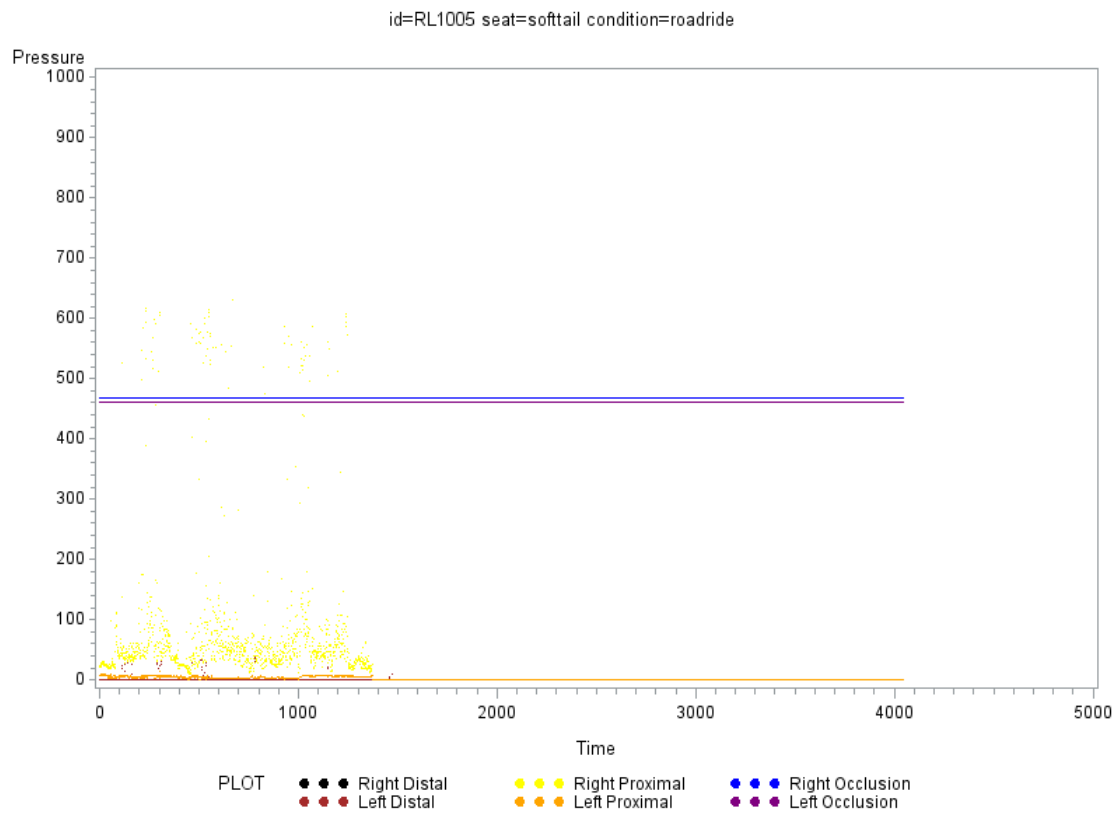

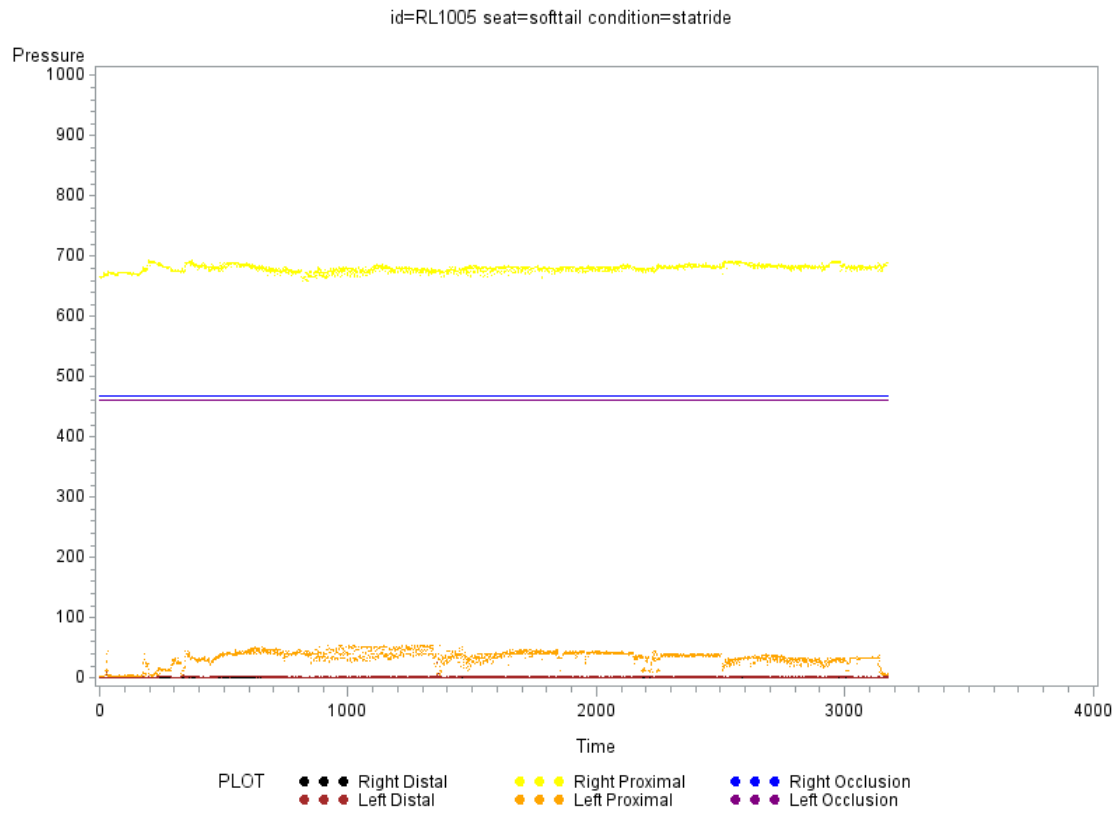

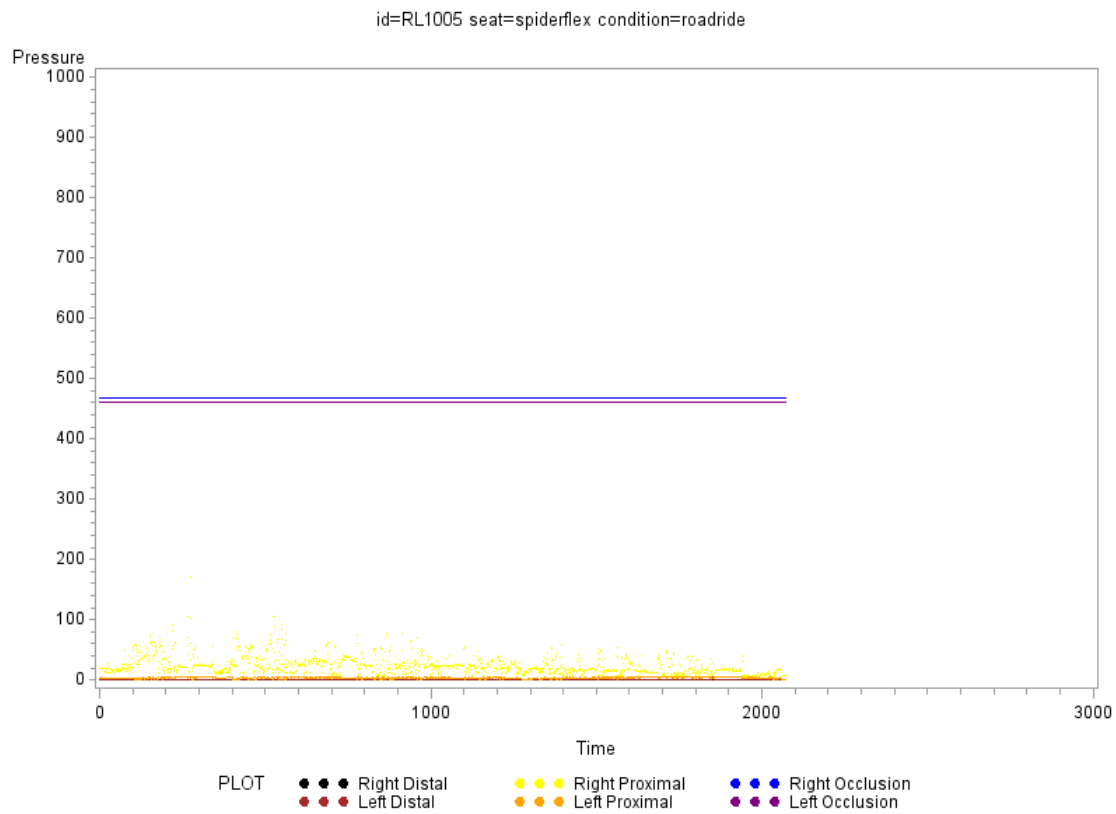

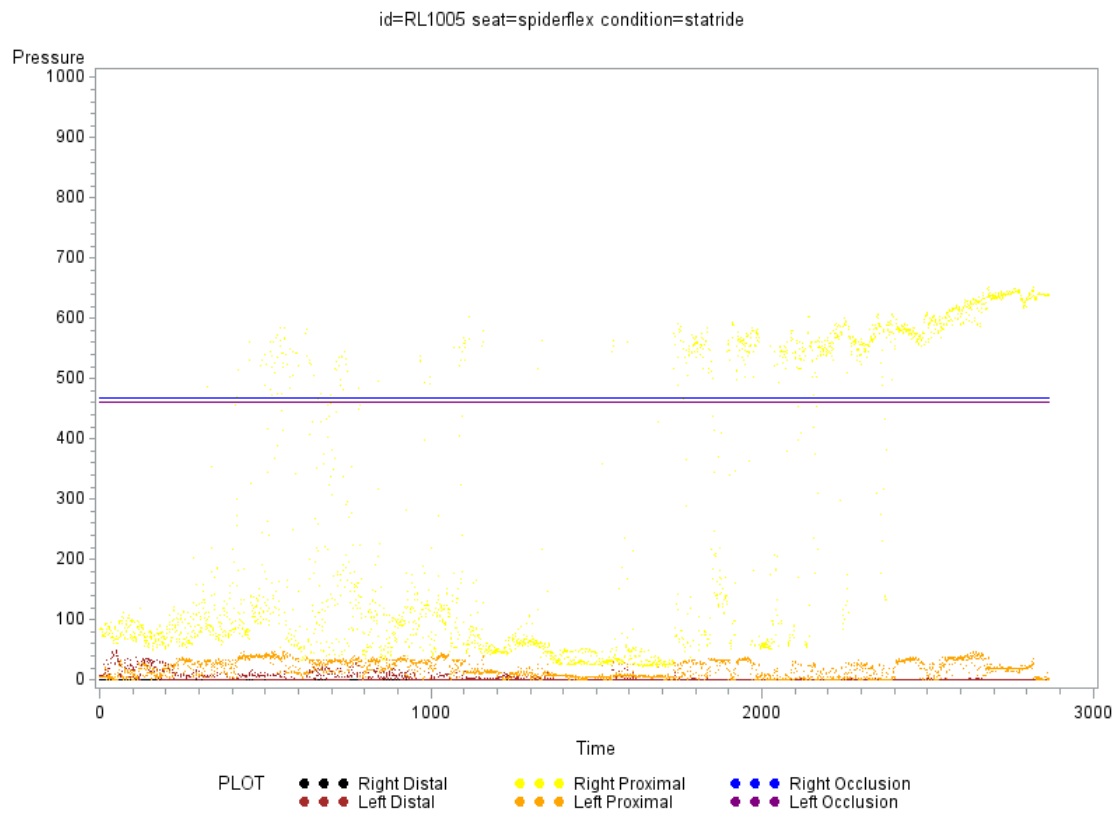

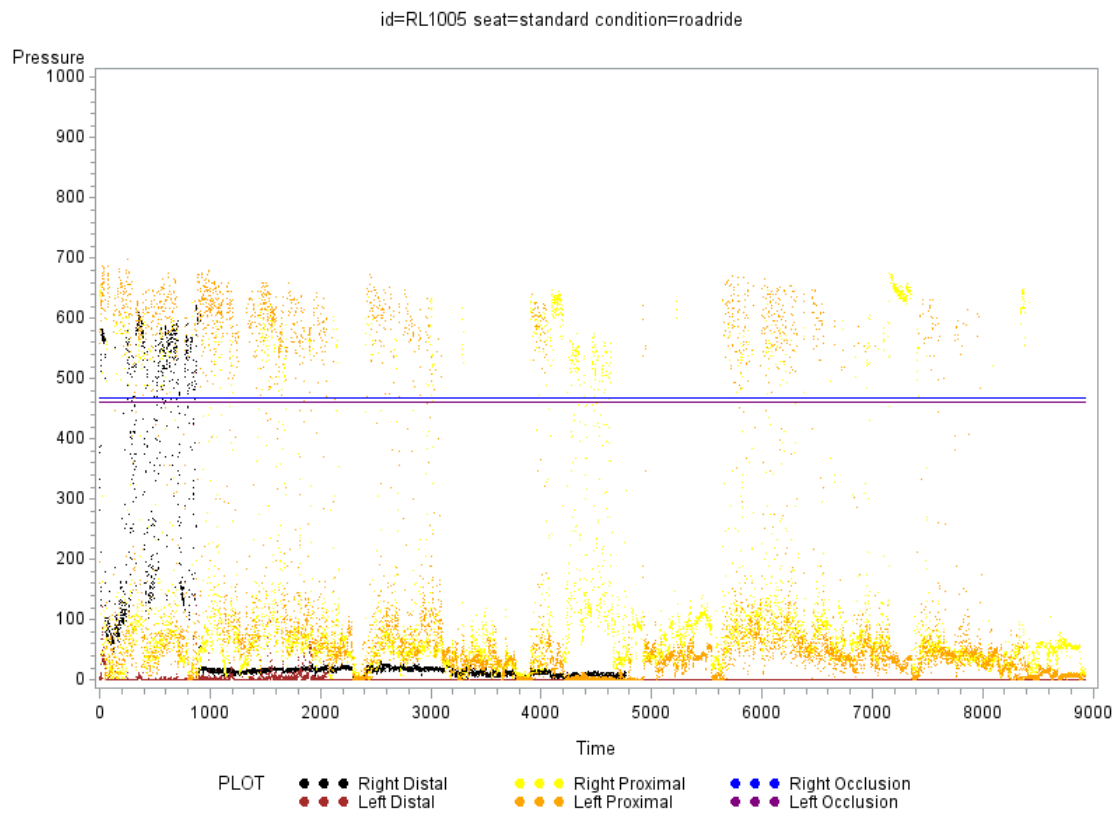

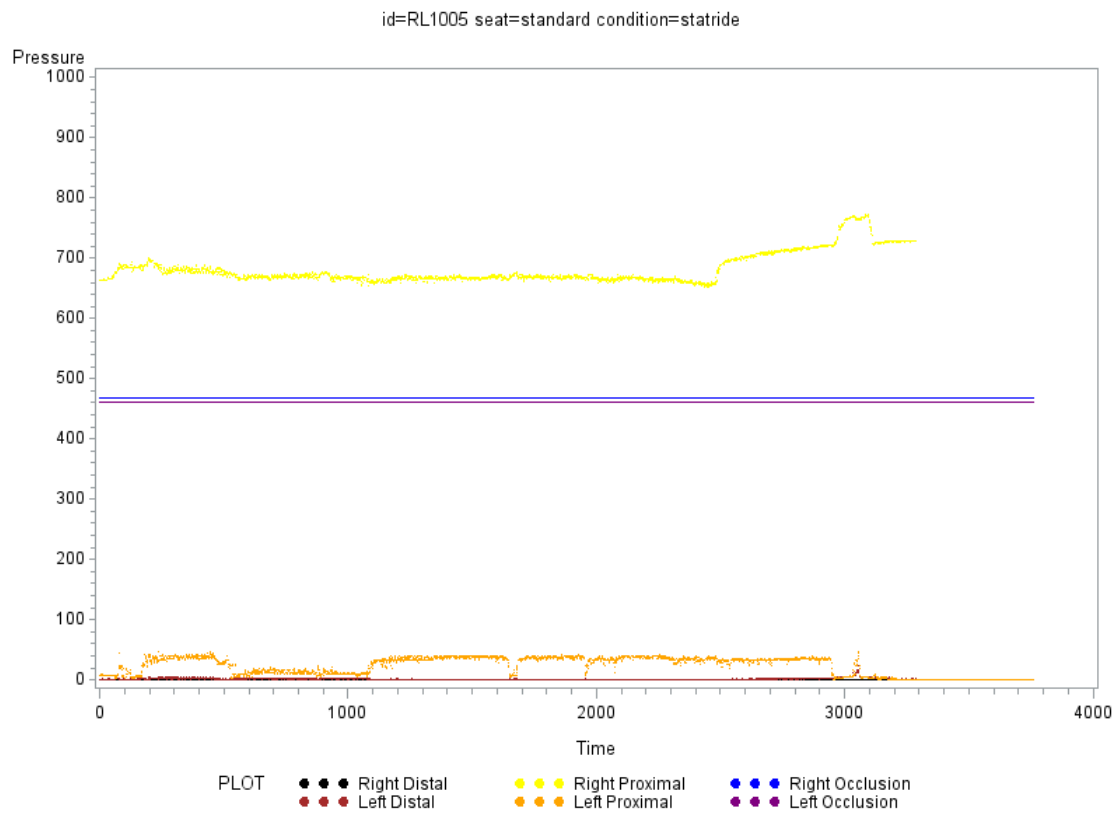

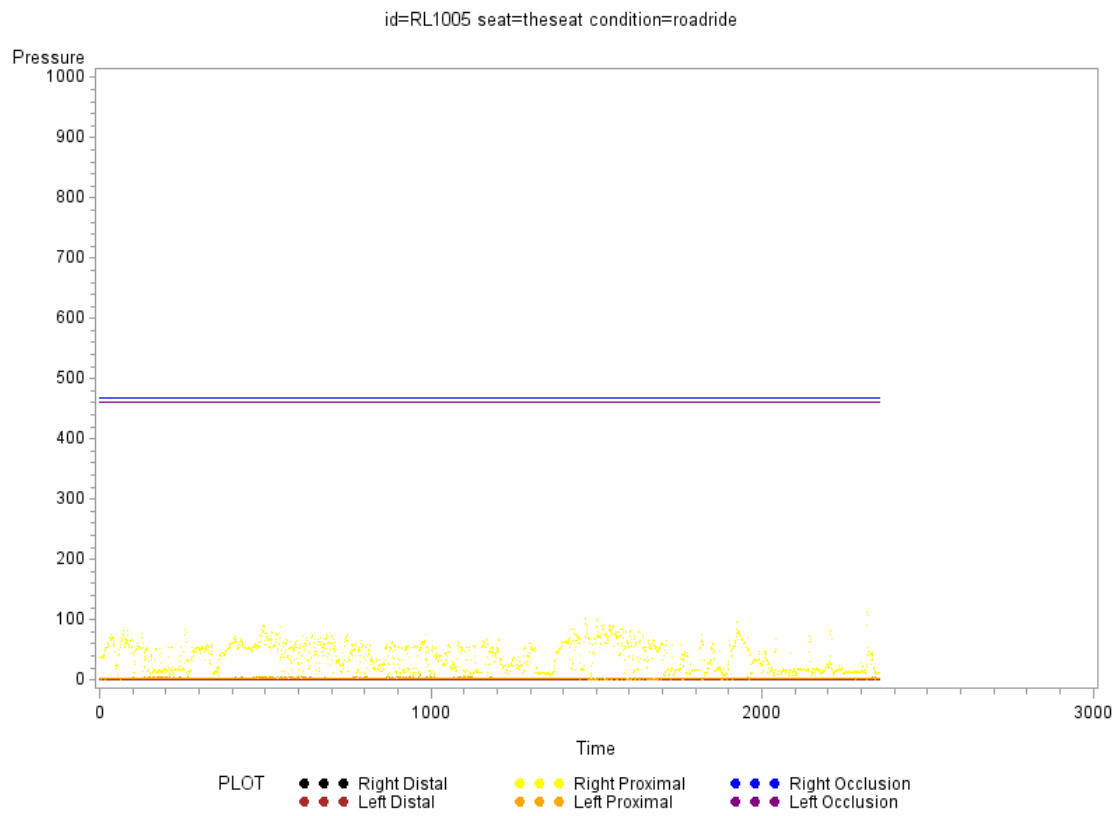

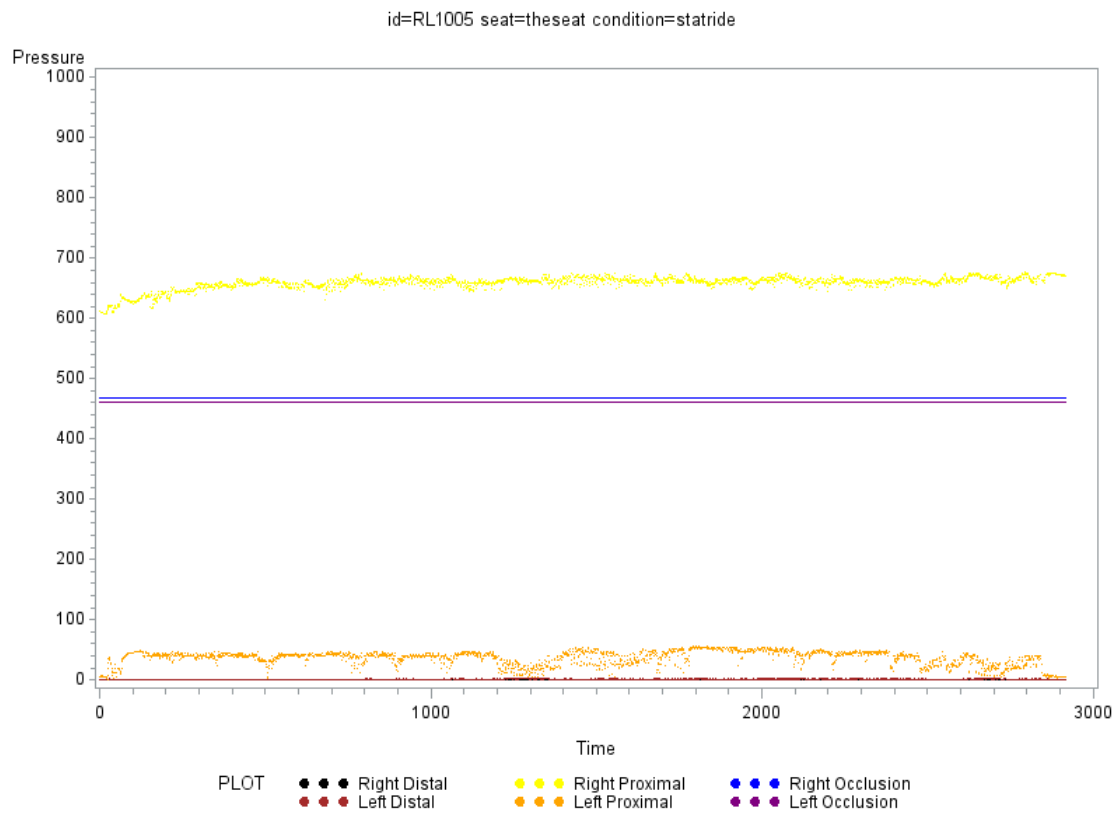

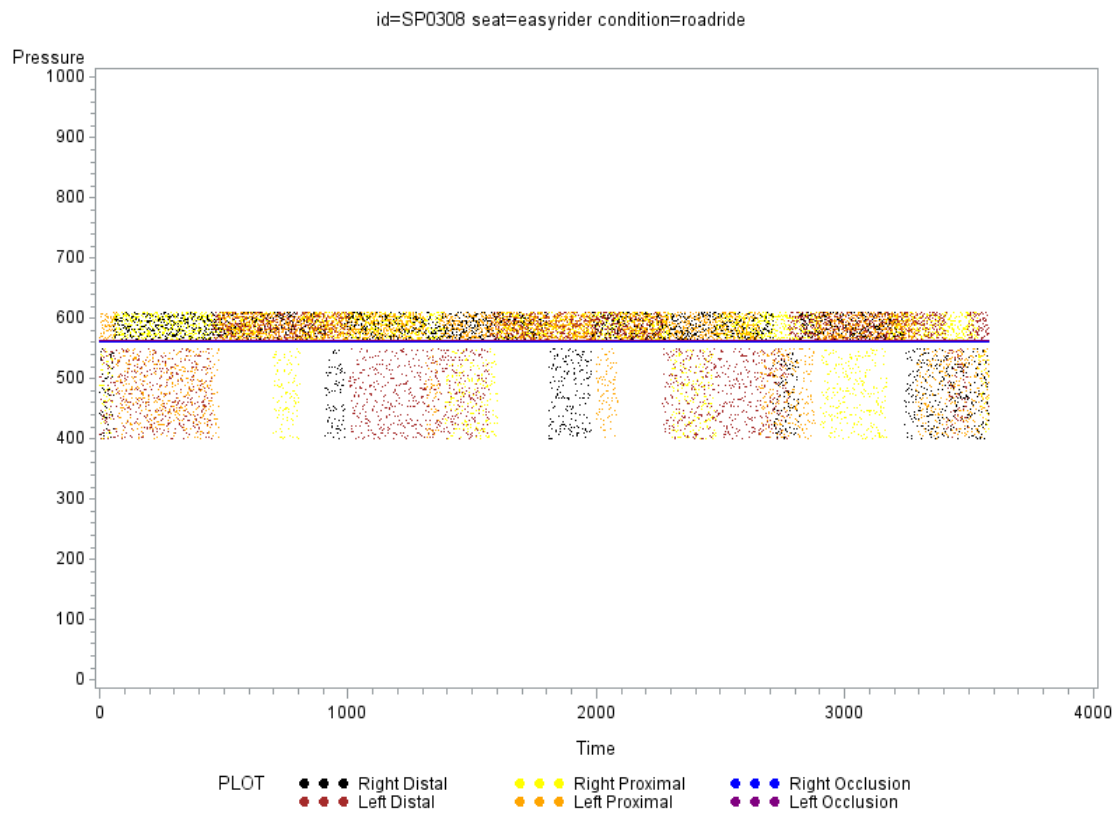

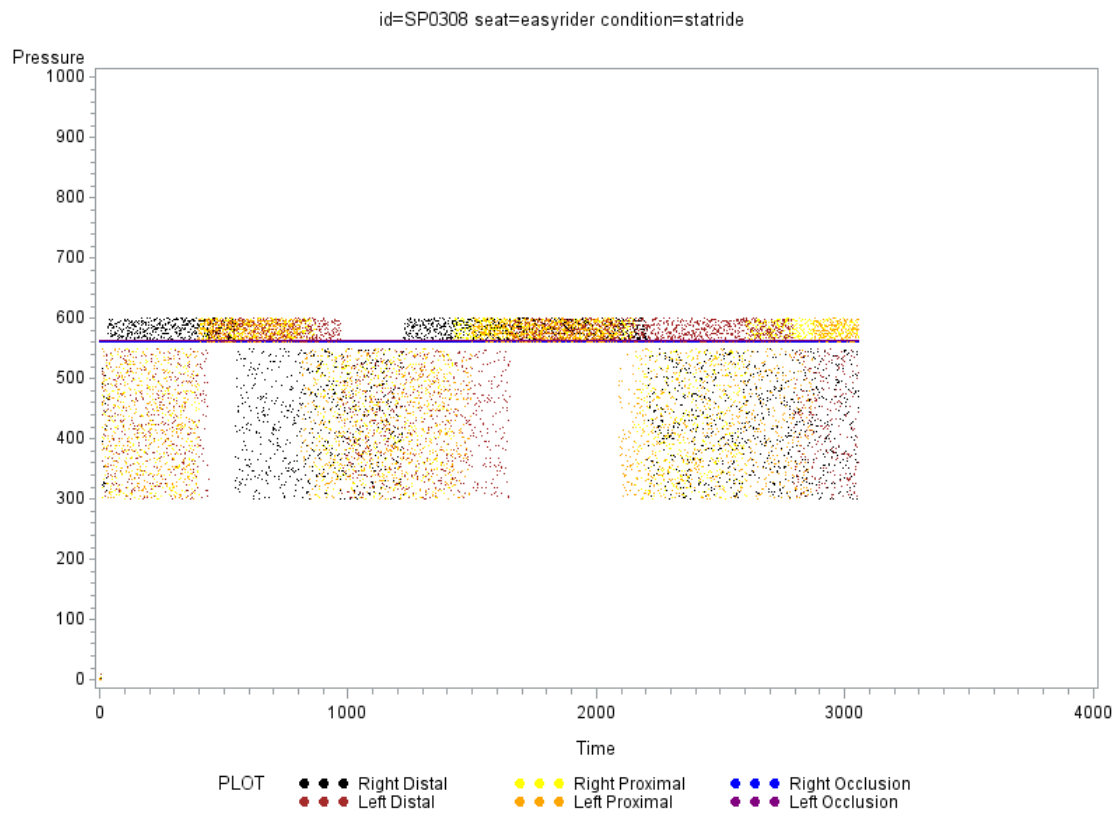

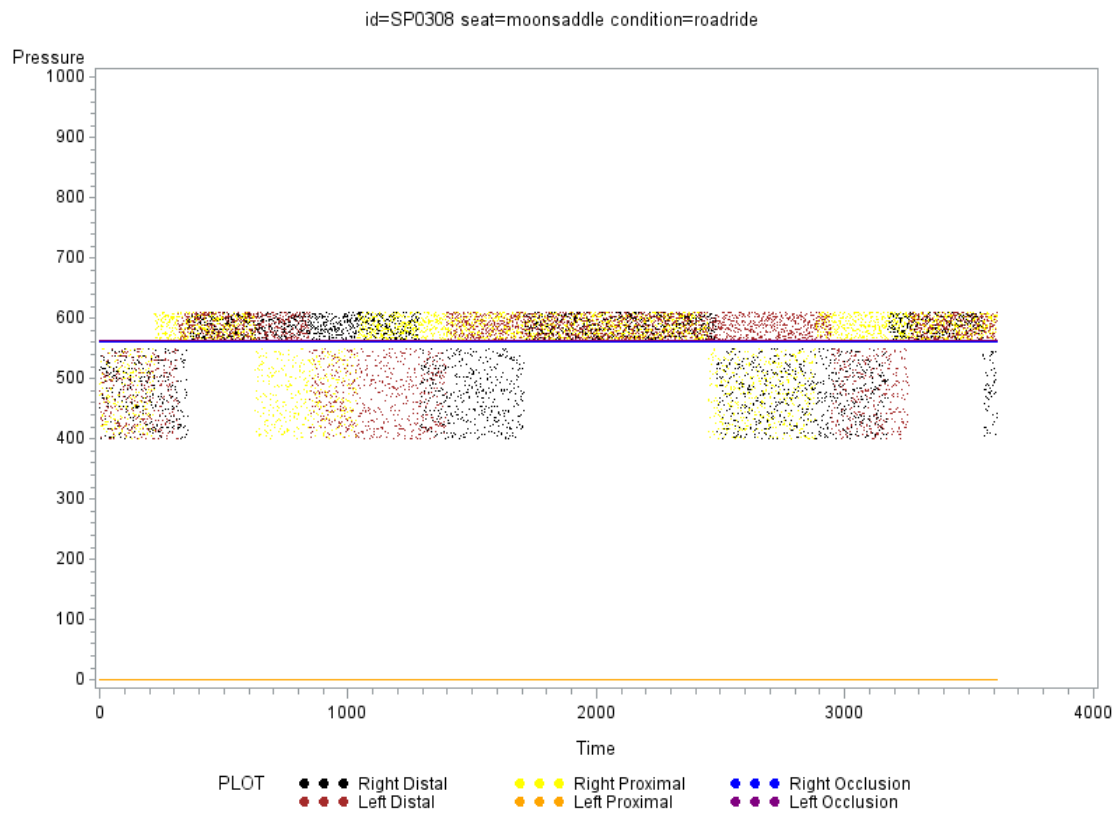

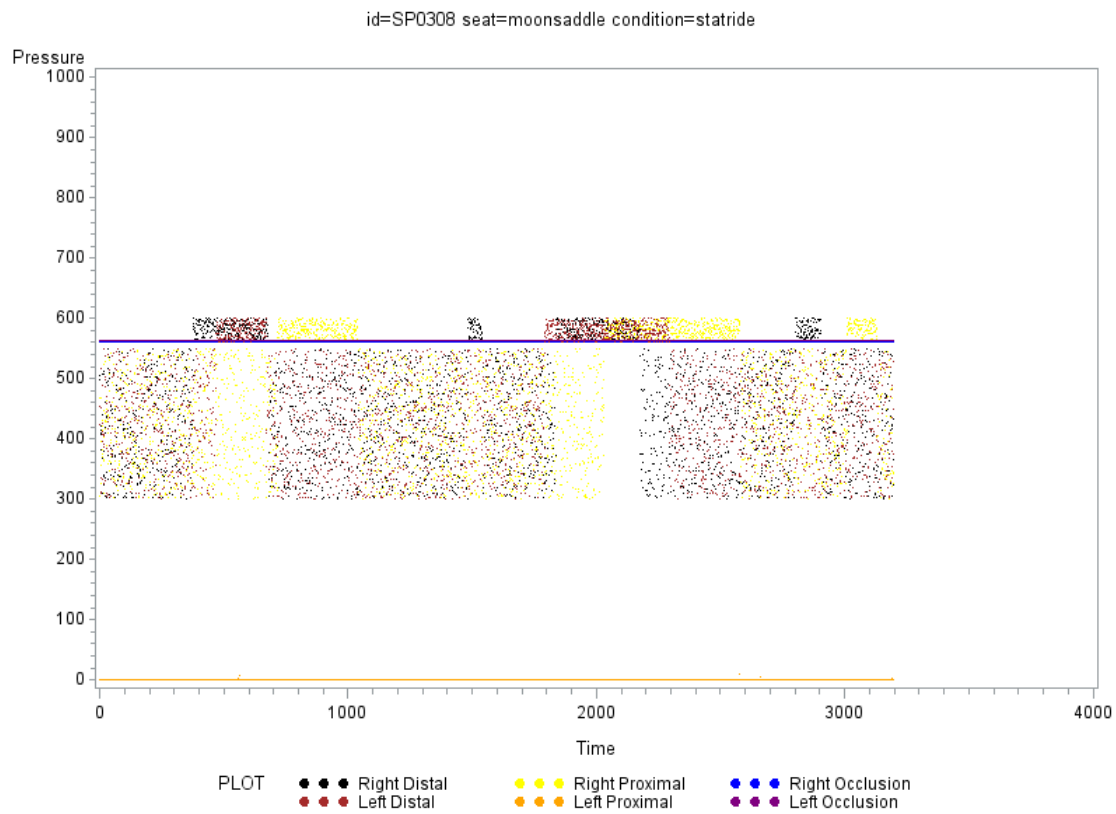

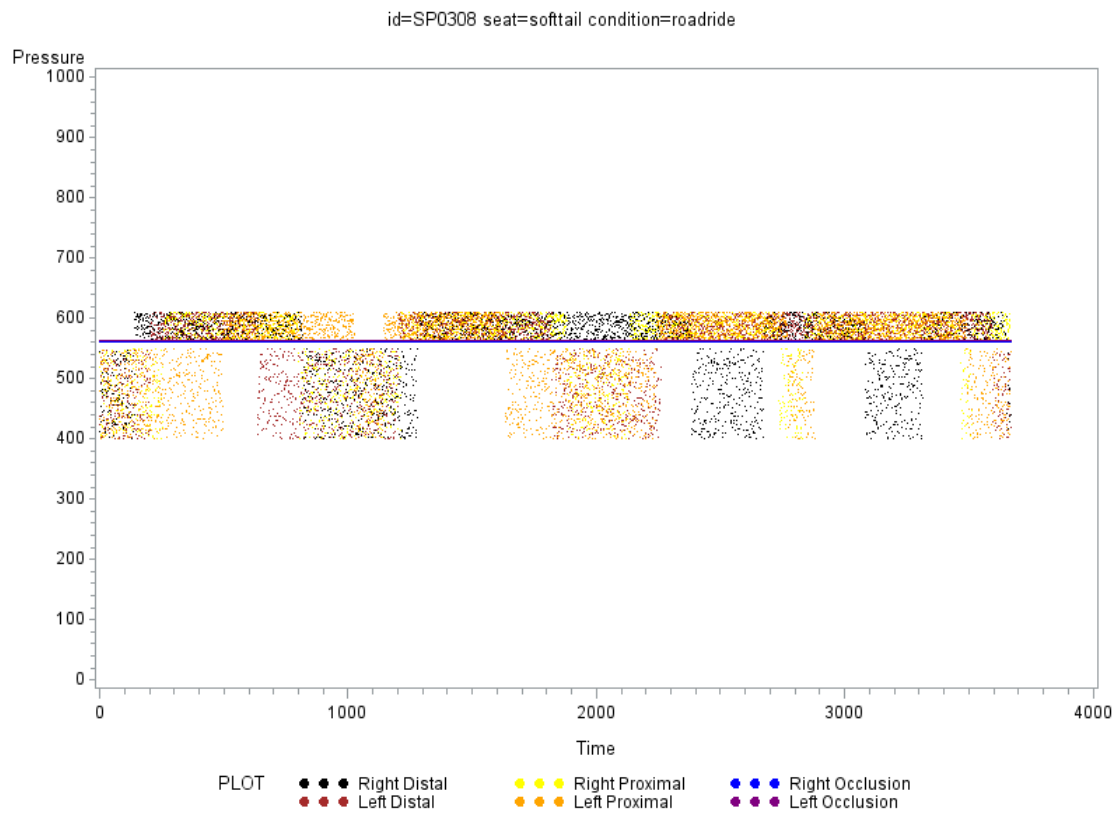

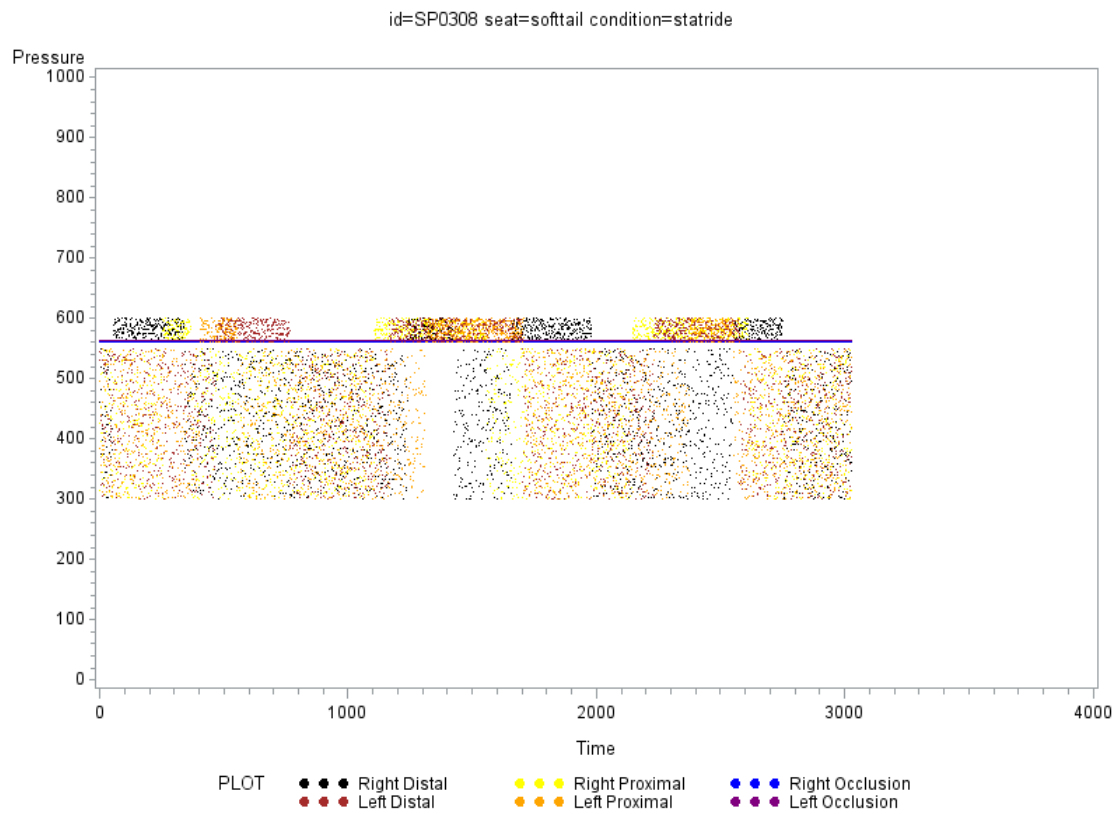

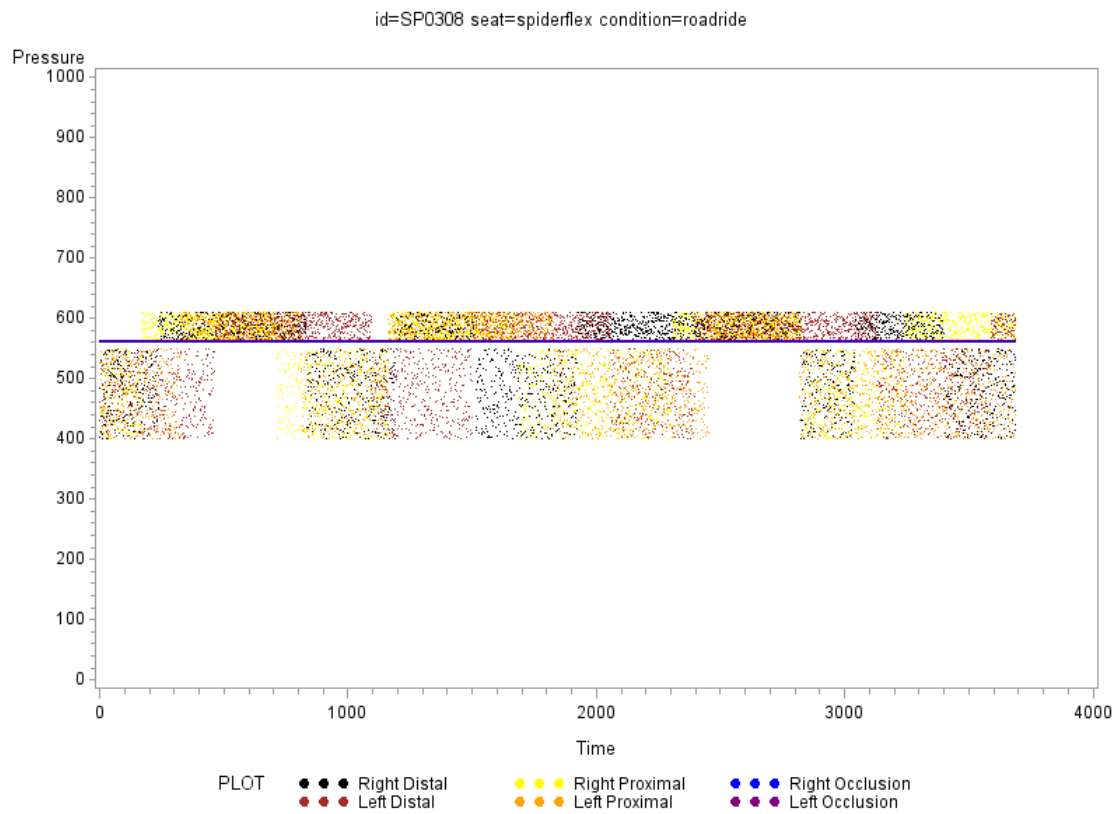

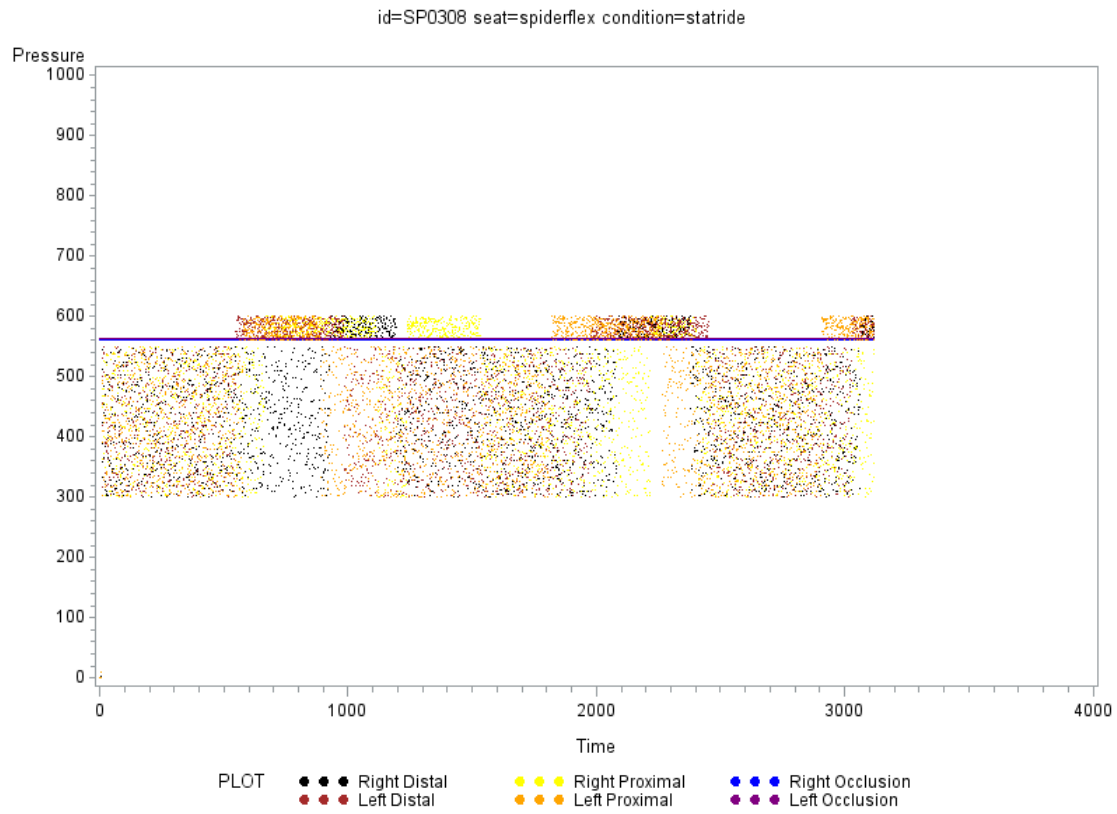

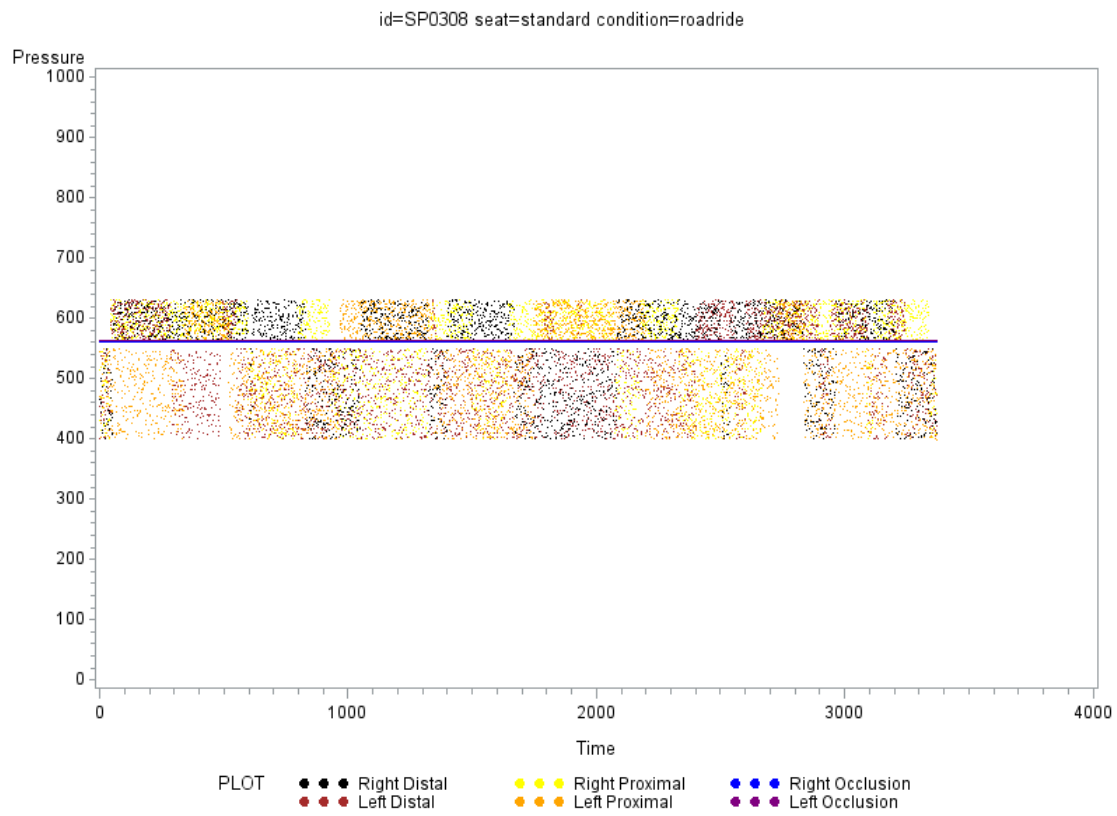

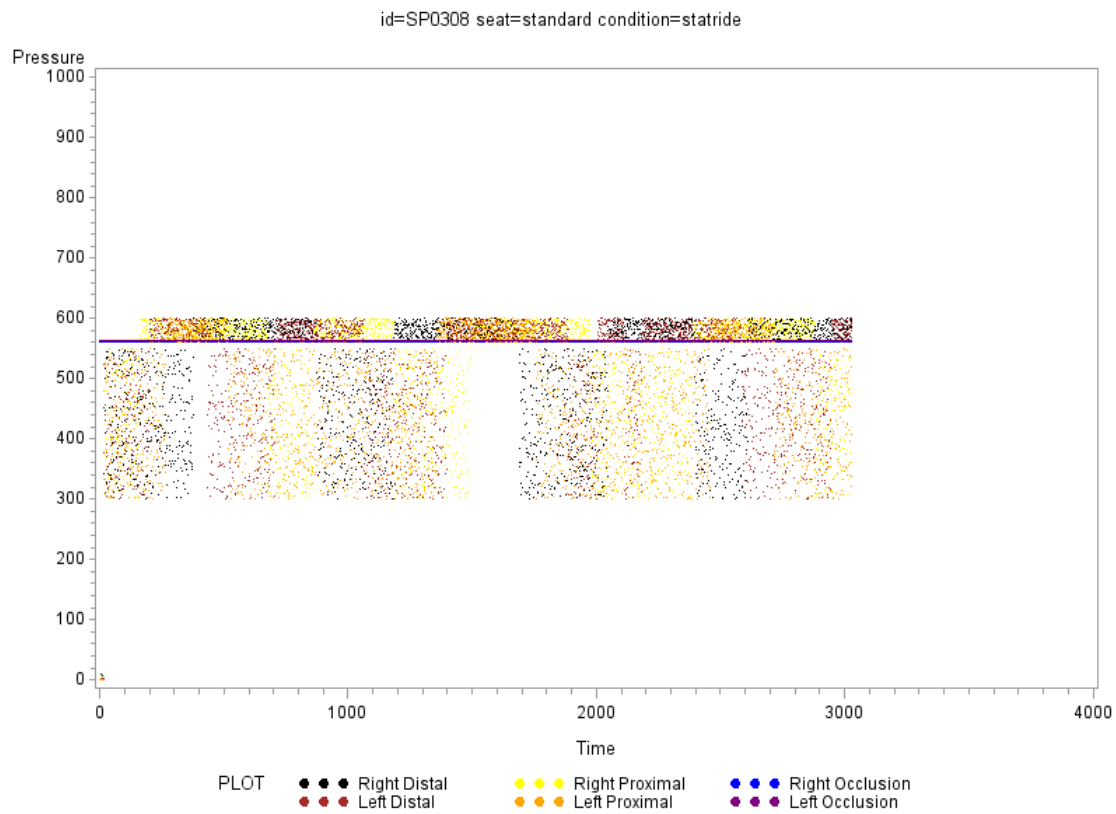

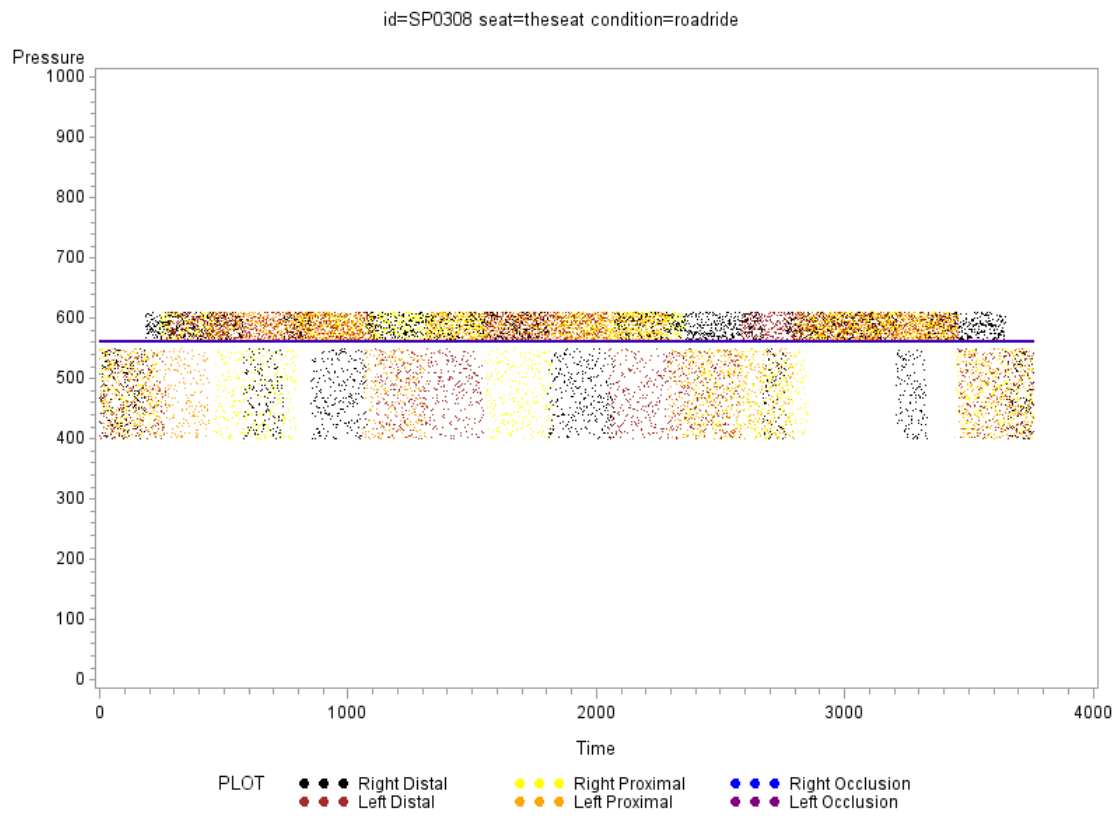

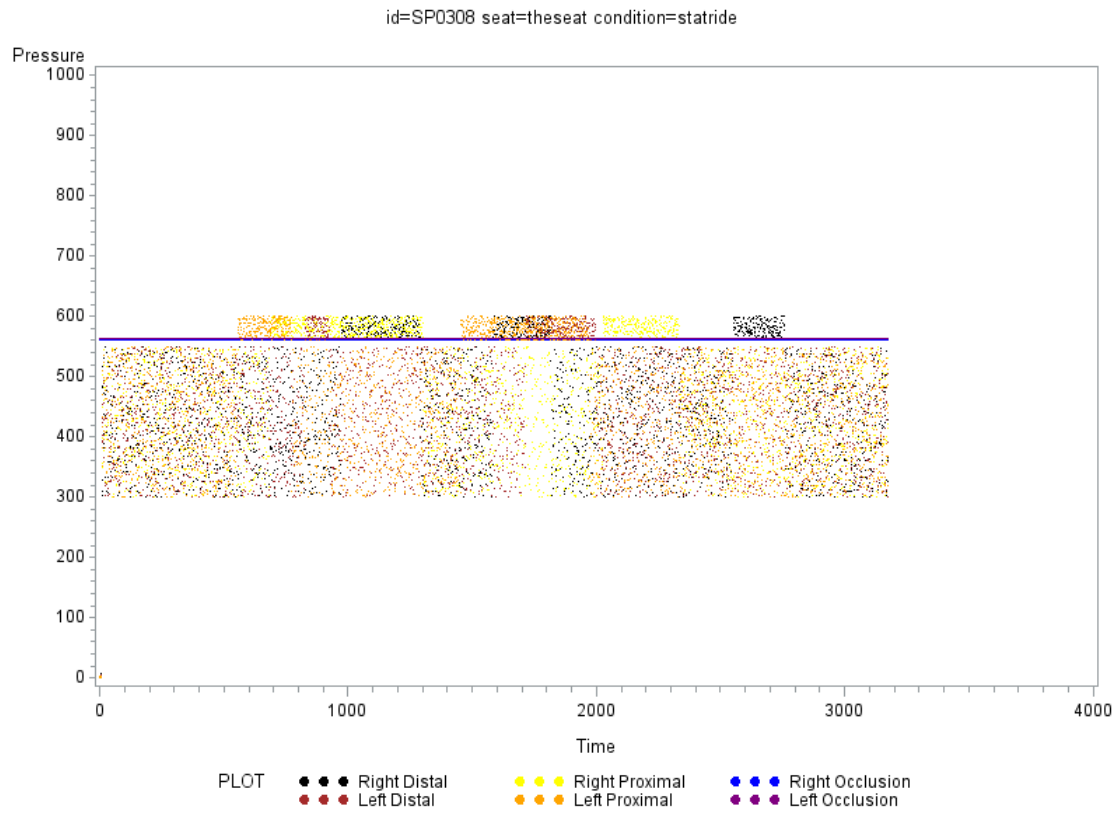

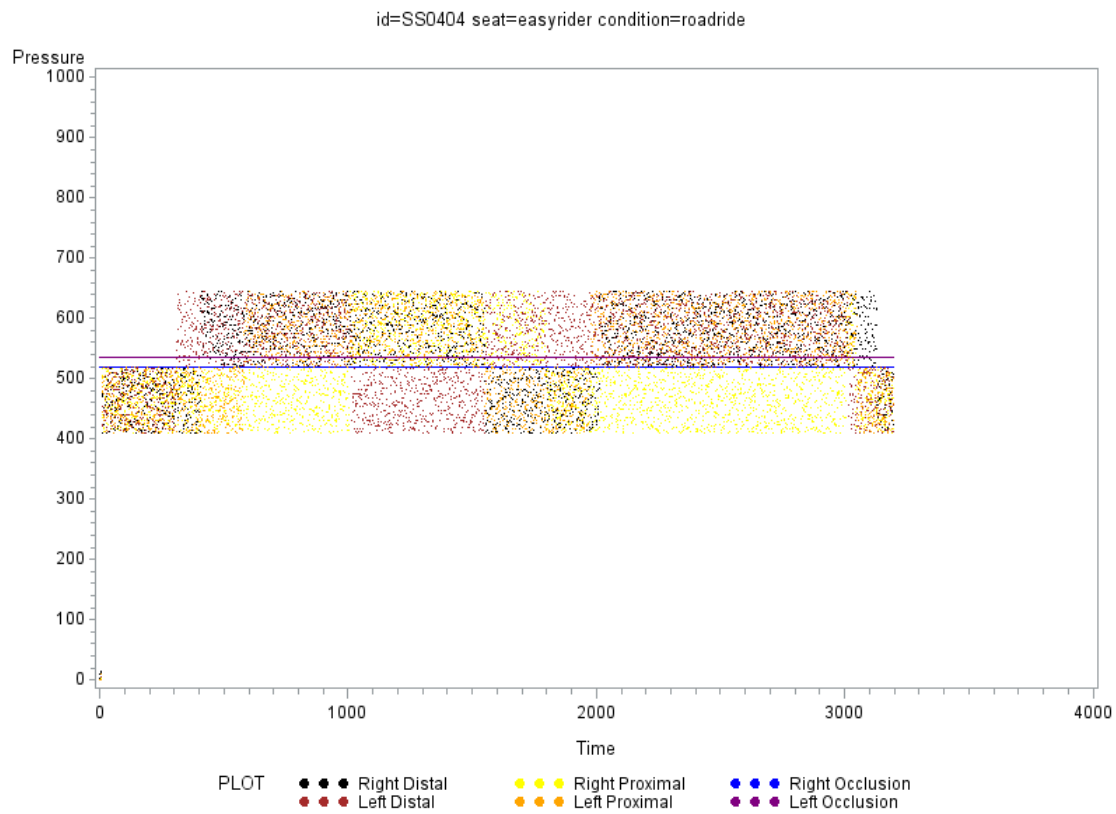

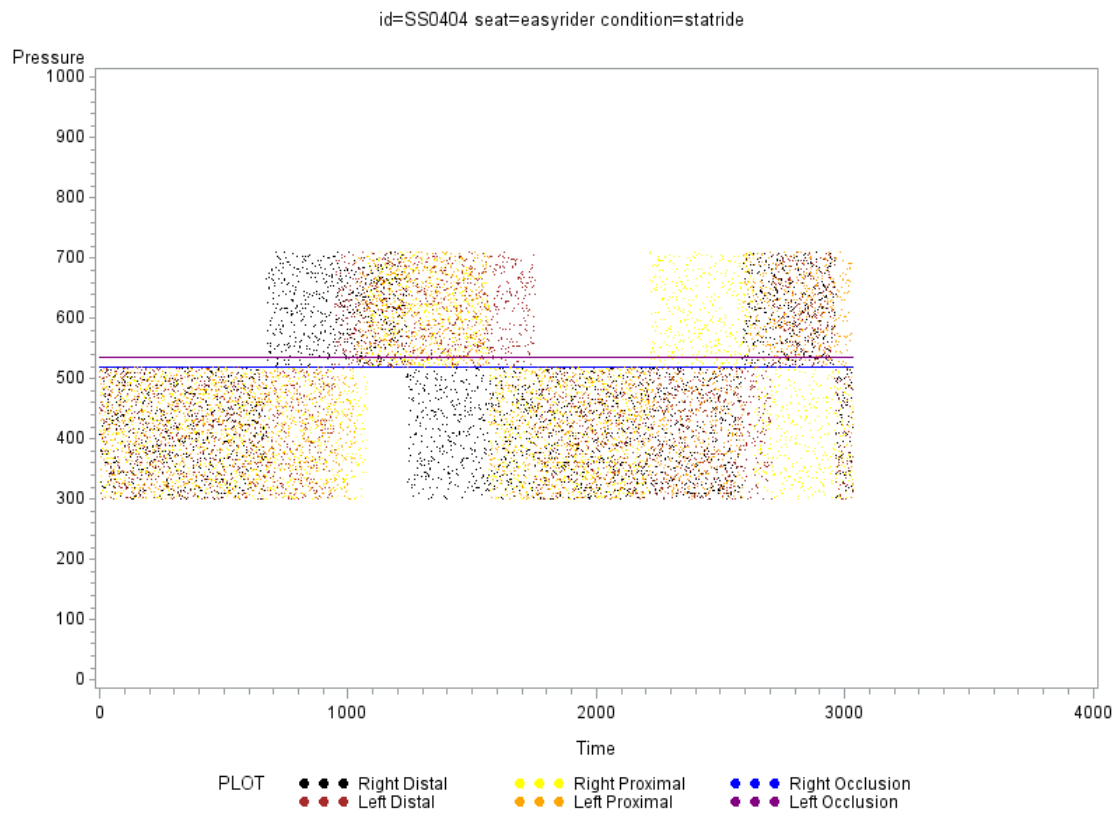

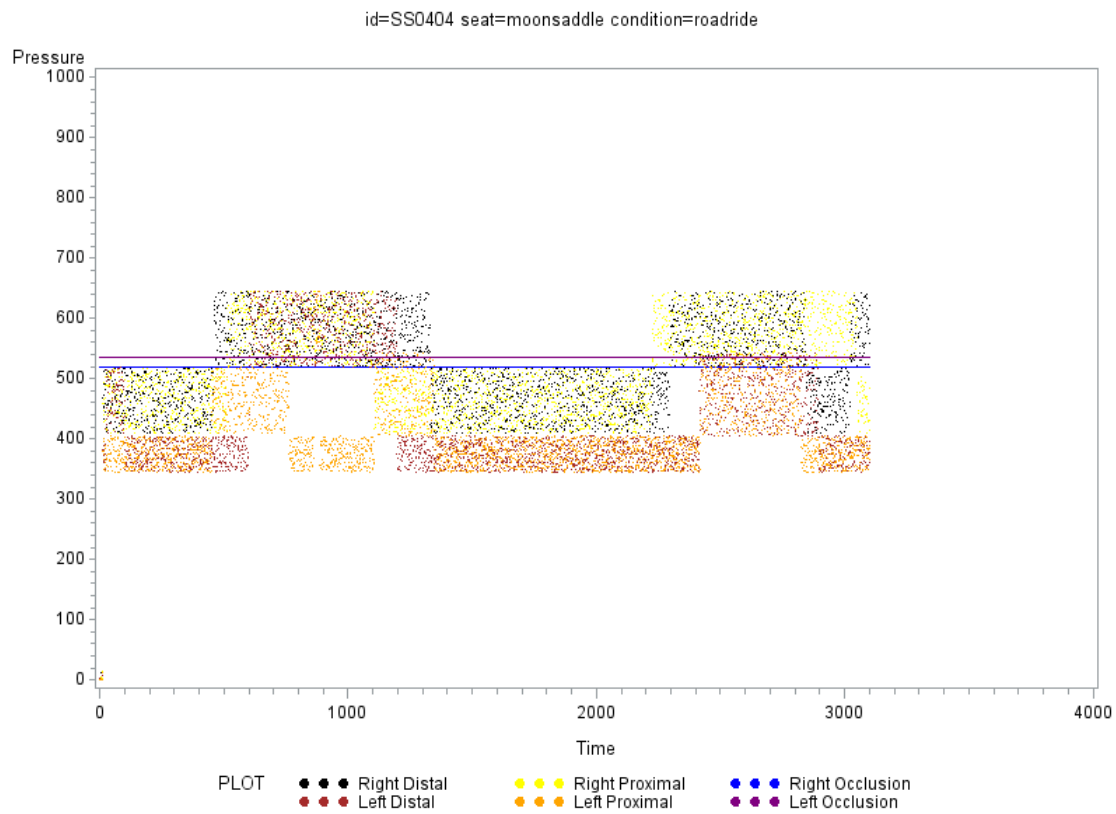

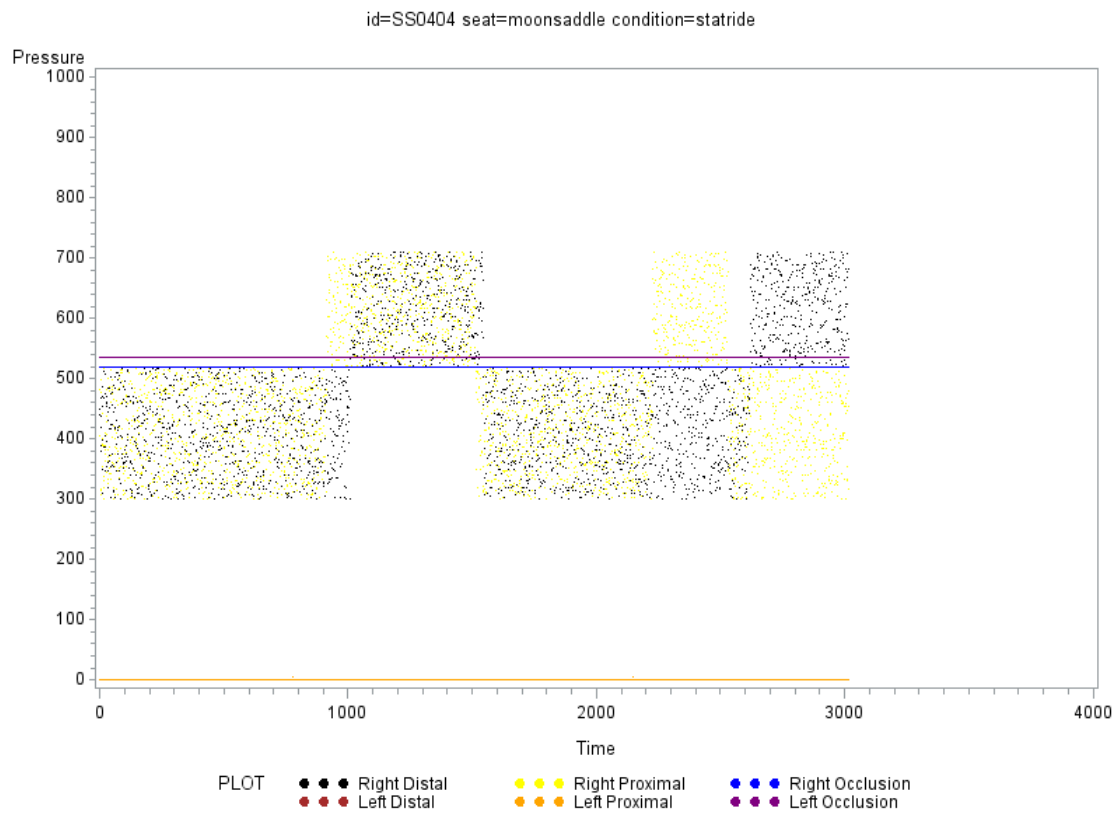

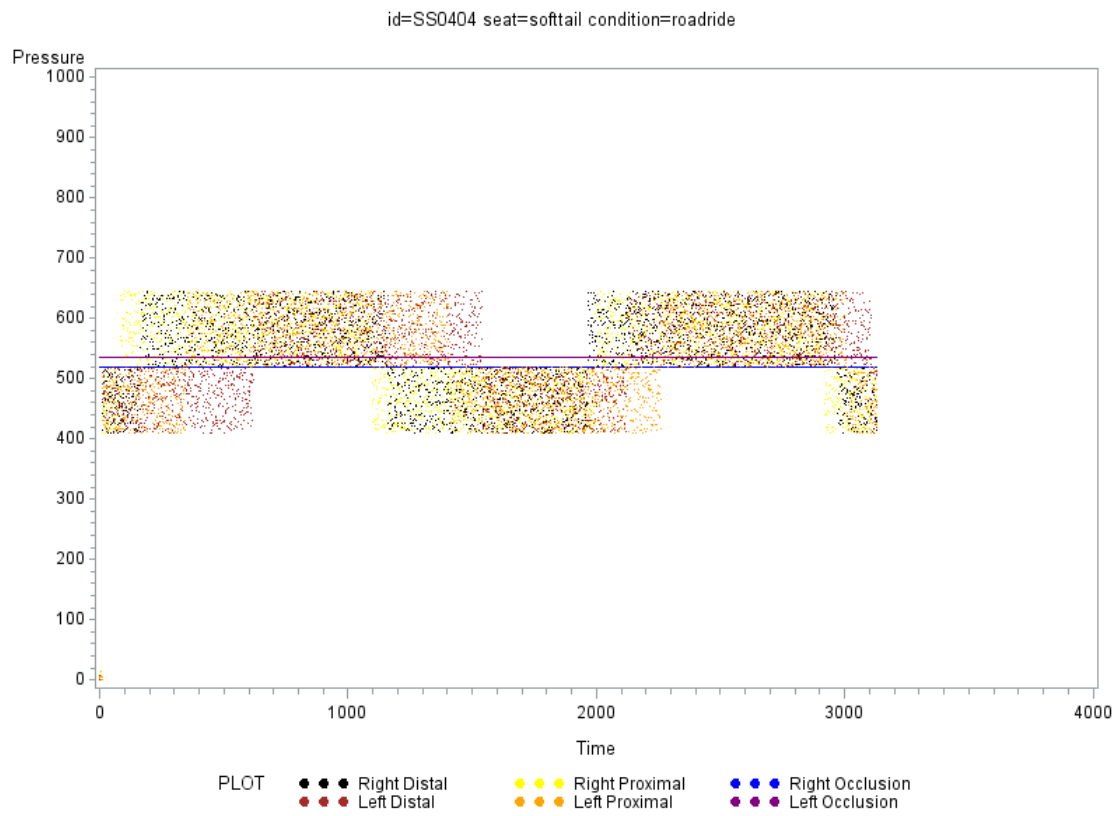

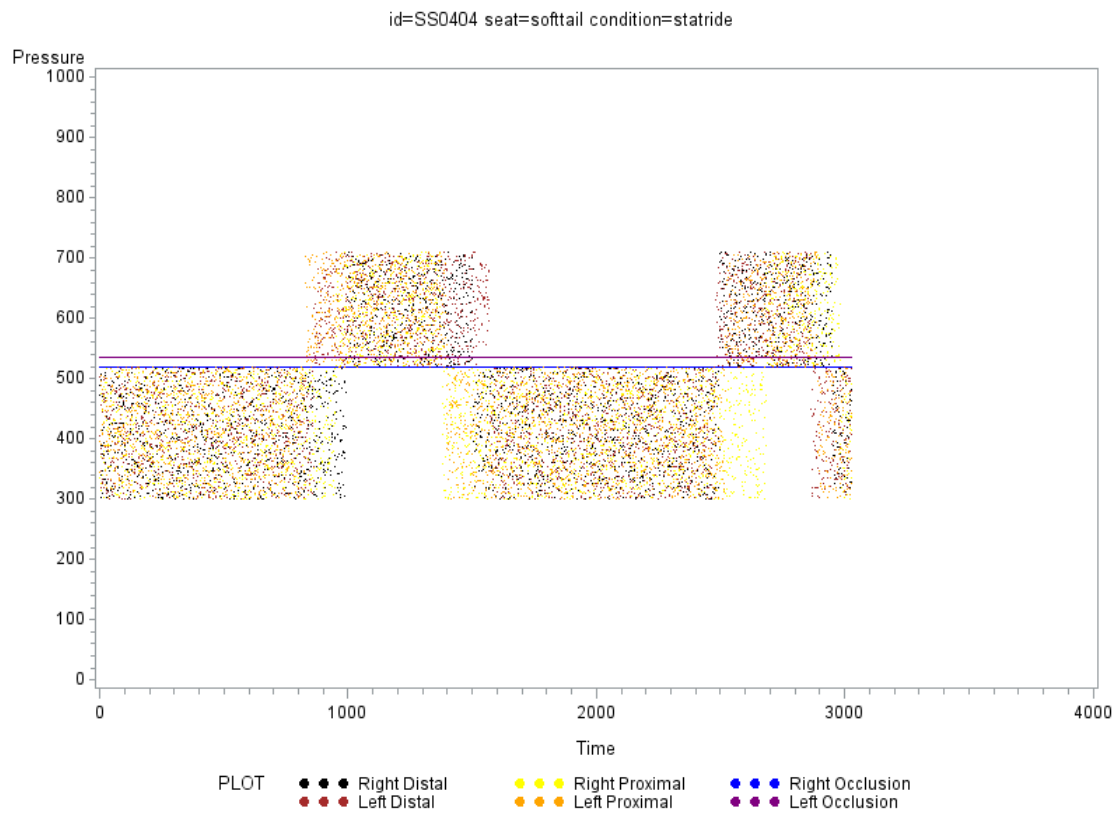

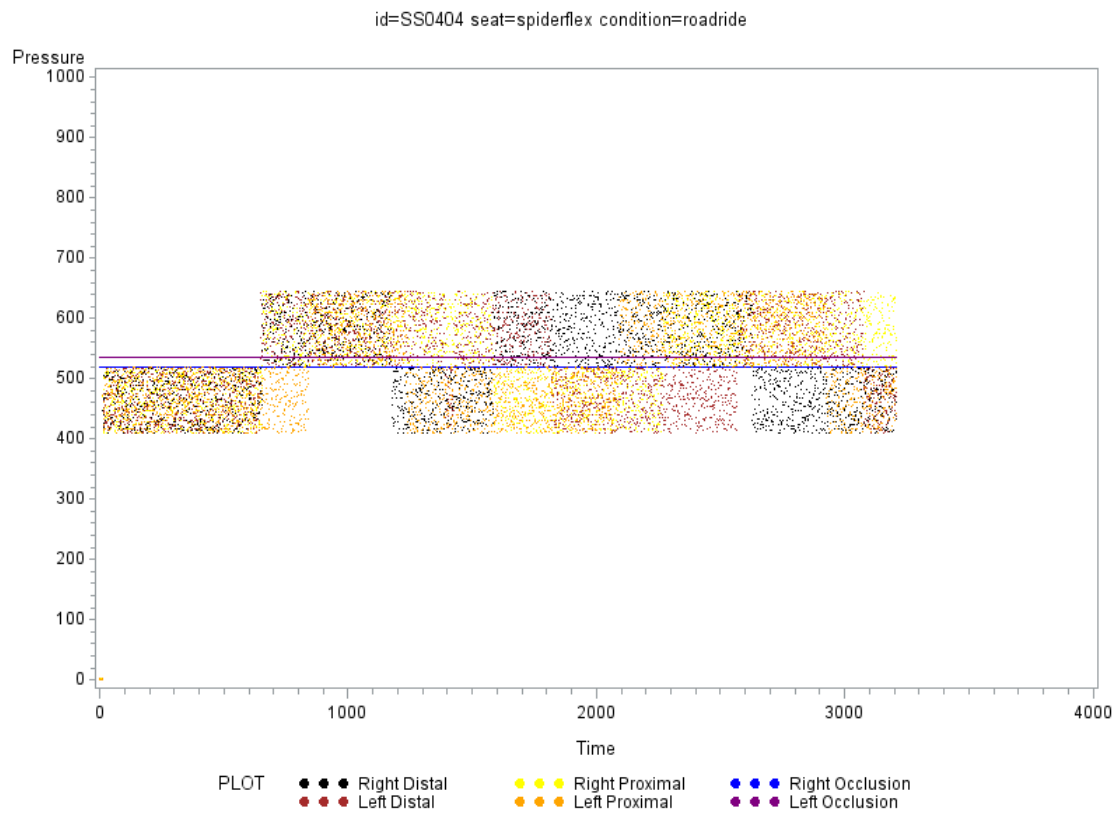

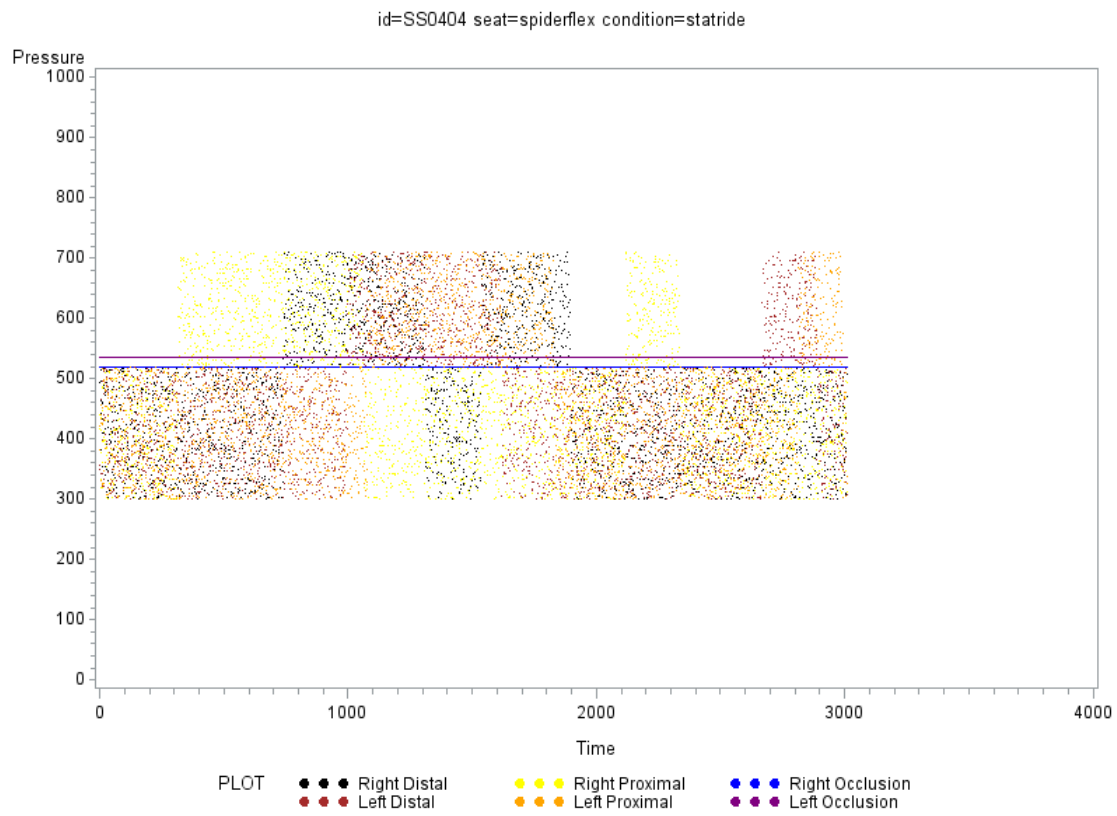

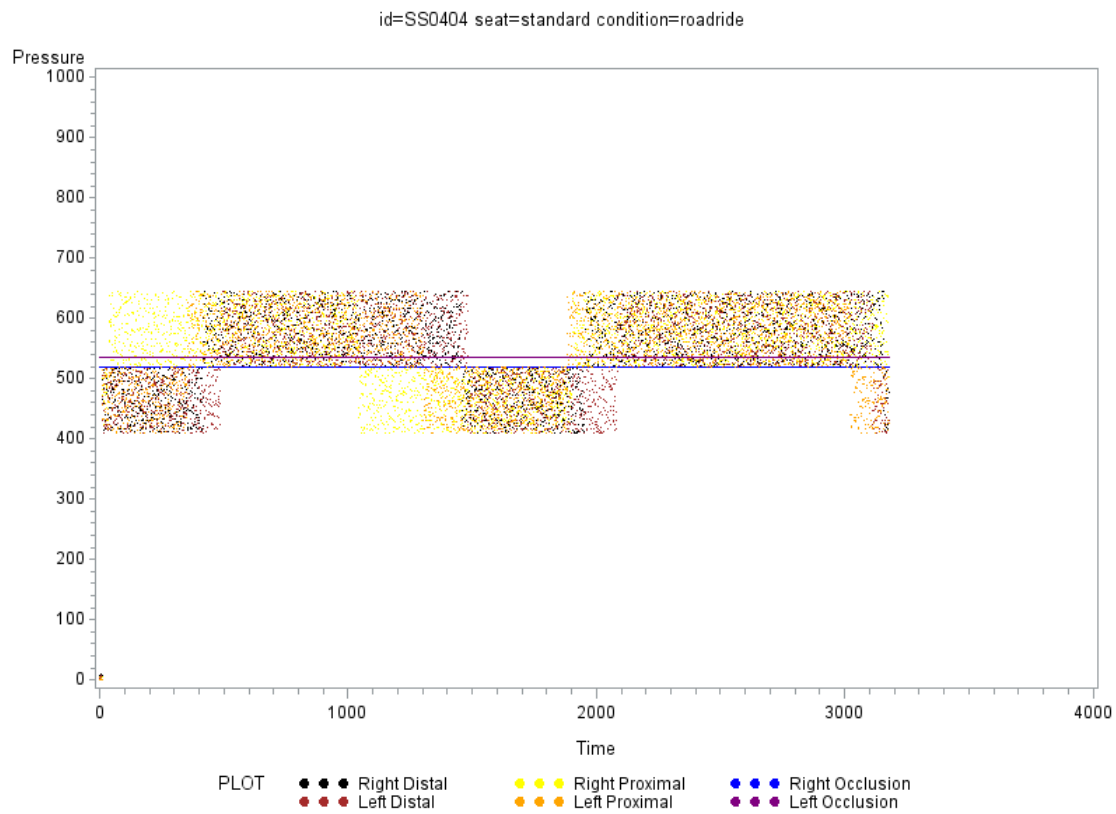

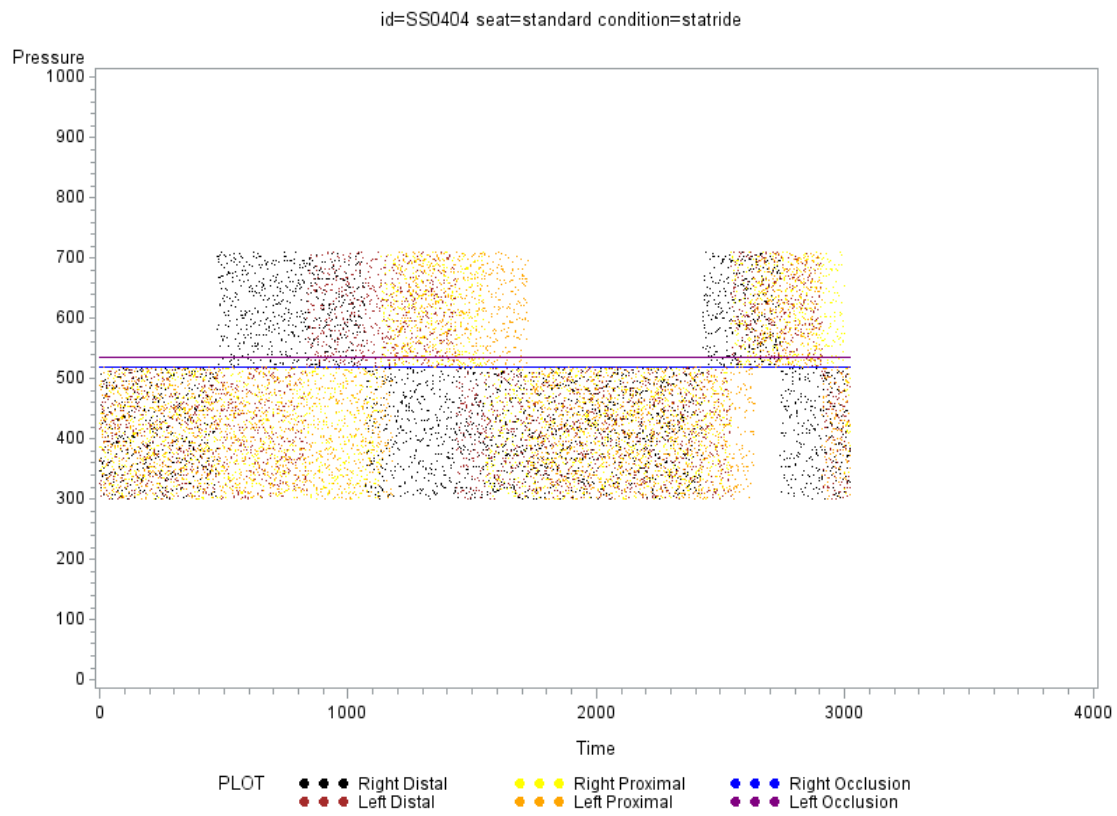

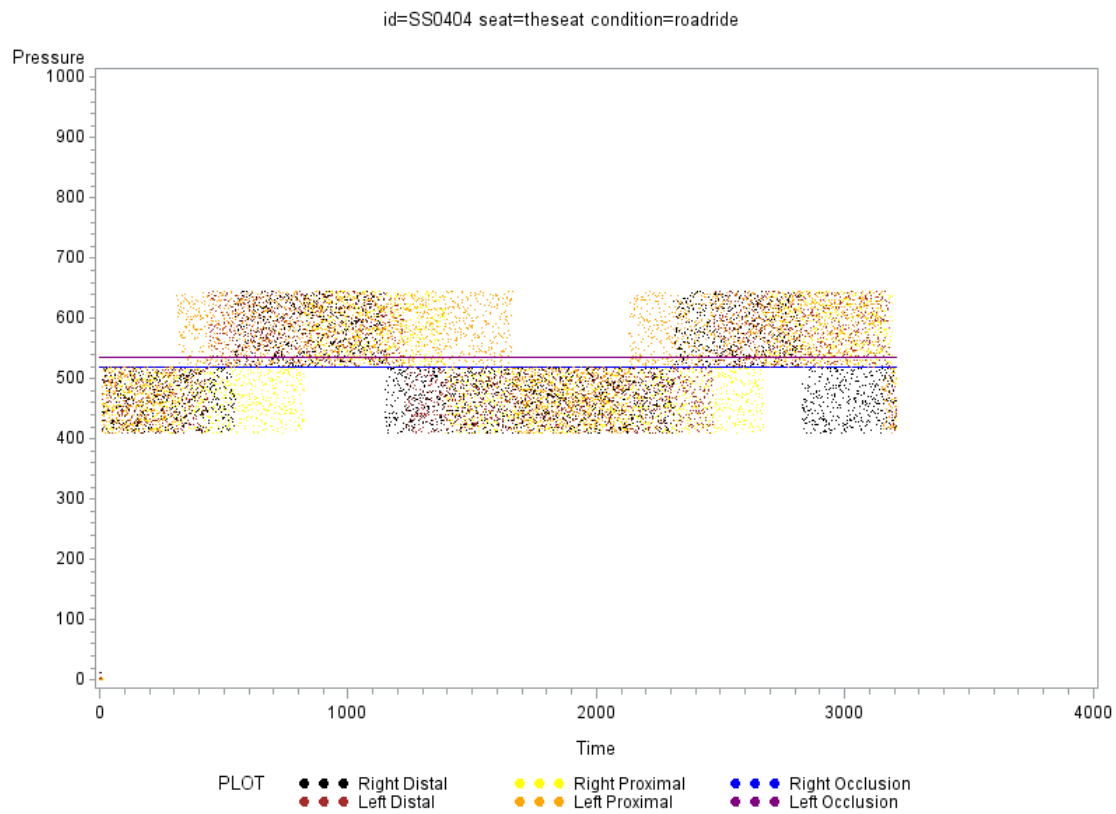

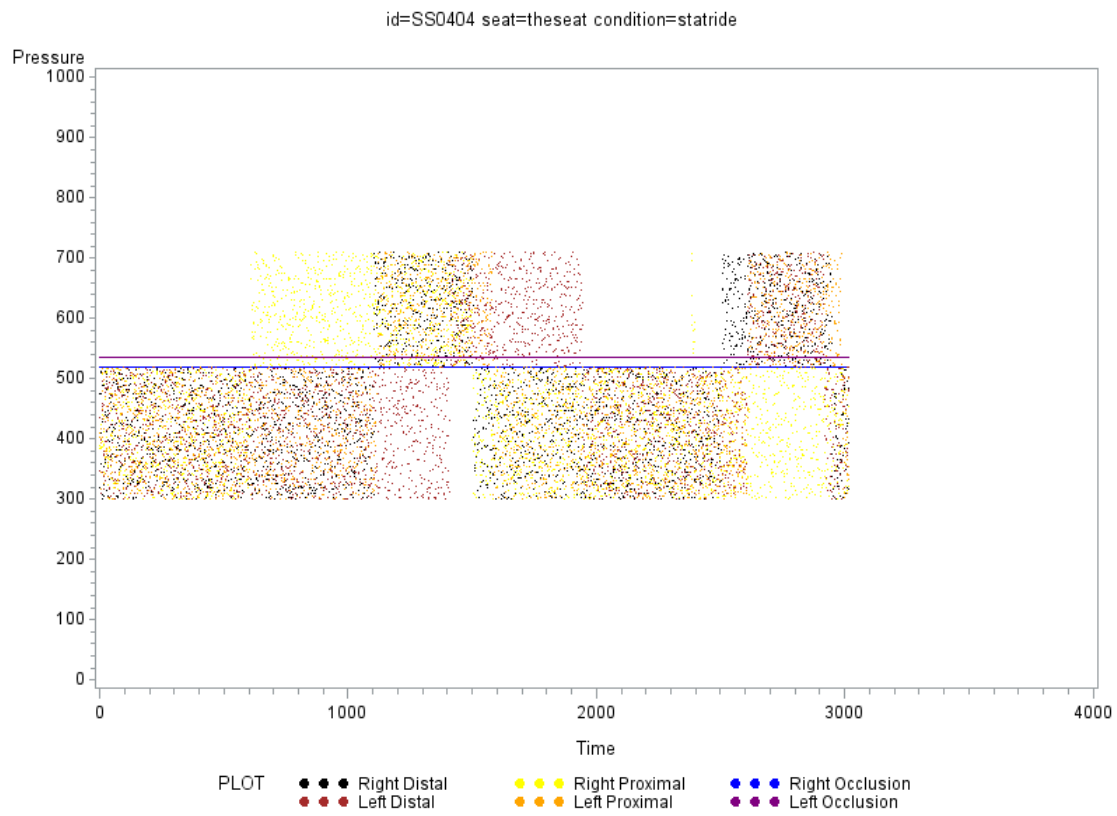

Supplement: Supplemental Information 1 — This pdf includes raw data from all the subjects. The values shown are actual sensor values and not converted into force in Newton. We had 20 subjects, each rode 6 different seats under 2 scenarios. So, we have in total 240 graphs representing each. [file peerj-03-1477-s001.pdf]
